# Supplementary material for: Impact of the COVID-19 pandemic on anxiety and depression symptoms of young people in the global south: evidence from a four-country cohort study
Source: BMJ Open. 2021 Apr 15;11(4):e049653. doi: 10.1136/bmjopen-2021-049653 (PMC8053815; doi:10.1136/bmjopen-2021-049653)
Supplement: Supplementary data [file bmjopen-2021-049653supp001.pdf]

Supplementary documents

Supplement to: Impact of the COVID-19 Pandemic on anxiety and depression symptoms of young people in the Global South: evidence from a four-country cohort study

- Survey questionnaire

Listening to Young Lives at Work: COVID-19 phone survey Second survey call

Data Handlers 2
Section 1. Introduction 7
Section 2. COVID-19: Behaviours and Risk Perceptions 10
Section 3. Socio-economic status 14
Section 4. Recent life history and Economic changes 18
4.1 Recent life history 18
4.2 Economic Shocks since the COVID-19 Outbreak 19
Section 5. Food Security, Prices and Health 22
5.1 Food Security 22
5.2 Food Prices 23
5.3 Individual Health 24
Section 6. Current Education 27
Section 7. Time Use 30
Section 8. Employment and Earnings 31
8.1 Labour Force participation 31
8.2 Main activity in the past 7 days 33
8.3 Main activity before COVID-19 38
8.4 Main activity during lockdown period 40
Section 9. Subjective wellbeing and Mental Health 42
9.1 Subjective wellbeing 42
9.2 Mental Health 43
Concluding remarks 46
Locating Information and contact info 48
Comments 49
Enumerator Statement 51
Codeboxes 52

Data Handlers

ETHIOPIA

CHILDID AND DATE

**0.1**	INSERT CHILD ID	**ET _ _ - 1 - _ _ _**	CHILDID
**0.2**	DATE OF INTERVIEW	_ _ / _ _ / 2 0 2 0<br>(day) (month) (year)	DINT
**0.3**	PHONE NUMBER USED FOR THE INTERVIEW		PHNUMB

DATA HANDLERS

Fieldworker that initiates questionnaire	**Name:**<br>✍___________________________	Code: [_ _ _]	FLWRIDPS
		**Date of check:**<br>_ _ / _ _ / 2 0 2 0<br>(day) (month) (year)	DINT
	ENUMERATOR ID number :<br>___________________		ID
	**Start time** (Use 24 hours):	[_ _ : _ _]	
Fieldworker that finishes questionnaire	**Name:**<br>✍___________________________	Code: [_ _ _]	ENDFLDPS
		**Date of check:**<br>_ _ / _ _ / 2 0 2 0<br>(day) (month) (year)	ENDDINT
	ENUMERATOR ID number 2:<br>___________________		ID2
	**End time** (Use 24 hours):	[_ _ : _ _]	

COMMENTS/OBSERVATIONS:

✍____

INDIA

CHILDID AND DATE

**0.1**	INSERT CHILD ID	**IN** __ __ __ __ __ __	CHILDID
**0.2**	DATE OF INTERVIEW	__ __ / __ __ / 2 0 2 0<br>(day) (month) (year)	DINT
**0.3**	PHONE NUMBER USED FOR THE INTERVIEW		PHNUMB

DATA HANDLERS

Fieldworker that initiates questionnaire	**Name:**<br>✍________________________________	Code: [__ __ __]	FLWRIDPS
		**Date of check:**<br>__ __ / __ __ / 2 0 2 0<br>(day) (month) (year)	DINT
	ID: ____________________		ID
	**Start time** (Use 24 hours):	[__ __ : __ __]	
Fieldworker that finishes questionnaire	**Name:**<br>✍________________________________	Code: [__ __ __]	ENDFLDPS
		**Date of check:**<br>__ __ / __ __ / 2 0 2 0<br>(day) (month) (year)	ENDDINT
	ID: ____________________		ID2
	**End time** (Use 24 hours):	[__ __ : __ __]	
**Supervisor**	**Name:**<br>✍________________________________	Code: [__ __ __]	SUPRIDPS
		**Date of check:**<br>__ __ / __ __ / 2 0 2 0<br>(day) (month) (year)	SUPCDATED

COMMENTS/OBSERVATIONS:

✍____

PERU

CHILDID AND DATE

**0.1**	INSERT CHILD ID	**PE __ __ - 1 - __ __ __**	CHILDID
**0.2**	DATE OF INTERVIEW	__ __ / __ __ / 2 0 2 0<br>(day) (month) (year)	DINT
**0.3**	PHONE NUMBER USED FOR THE INTERVIEW		PHNUMB

DATA HANDLERS

Fieldworker that initiates questionnaire	**Name:**<br>✍_______________________	Code: [__ __ __]	FLWRIDR6
		**Date of check:**<br>__ __ / __ __ / 2 0 2 0<br>(day) (month) (year)	DINT
	ID: ____________________		ID
	**Start time** (Use 24 hours):	[__ __ : __ __]	
Fieldworker that finishes questionnaire	**Name:**<br>✍_______________________	Code: [__ __ __]	ENDFLDPS
		**Date of check:**<br>__ __ / __ __ / 2 0 2 0<br>(day) (month) (year)	ENDDINT
	ID2: ____________________		ID2
	**End time** (Use 24 hours):	[__ __ : __ __]	

**Supervisor**	**Name:**<br>✍________________________________	Code: [__ __ __]	SUPRIDPS
		**Date of check:**<br>__ __/ __ __/ 2 0 2 0<br>(day) (month) (year)	SUPCDATED

COMMENTS/OBSERVATIONS:

✍________________________________

VIETNAM

CHILDID AND DATE

**0.1**	INSERT CHILD ID	**VN**__ __ __ __ __ __	CHILDID
**0.2**	DATE OF INTERVIEW	__ __/ __ __/ 2 0 2 0<br>(day) (month) (year)	DINT
**0.3**	PHONE NUMBER USED FOR THE INTERVIEW		PHNUMB

DATA HANDLERS

Fieldworker that initiates questionnaire	**Name:**<br>✍________________________________	Code: [__ __ __]	FLWRIDPS
		**Date of check:**<br>__ __/ __ __/ 2 0 2 0<br>(day) (month) (year)	DINT
	ID: ______________________		ID
	**Start time** (Use 24 hours):	[__ __ : __ __]	
Fieldworker that finishes questionnaire	**Name:**<br>✍________________________________	Code: [__ __ __]	ENDFLDPS

		**Date of check:**<br>__ __ / __ __ / 2 0 2 0<br>(day) (month) (year)	ENDDINT
	ID: ______________________________		ID2
	**End time** (Use 24 hours):	[__ __ : __ __]	
**Supervisor**	**Name:**<br>✍____	Code: [__ __ __]	SUPRIDPS
		**Date of check:**<br>__ __ / __ __ / 2 0 2 0<br>(day) (month) (year)	SUPCDATED

COMMENTS/OBSERVATIONS:

✍____

Section 1. Introduction

ENUMERATOR: Start the dialogue with YL Child as follows:

SAY: Hello, (are you) / is this [YL Child´s name] ? Hi, I am [first and last name of ENUMERATOR], of the Young Lives Study. The last time we talked to you over the phone was in [DATE OF CALL 1], how are you?

On that occasion we told you that we were going to contact you again to invite you to participate in a phone survey with questions related to the Covid-19 /Coronavirus, the quarantine, and its effects on your health, wellbeing, education, economy and work and your household. The information we are collecting from you and the other Young Lives Children in our country will help the authorities of the government and our health system to know how to better prevent and deal with these types of emergencies, for the benefit of the entire population.

The entire survey takes approximately 45 minutes. As always, the survey will be done with your consent and on the day and time that is most convenient for you. You know that everything you tell me is confidential.

Finally, as I told you the last time we talked, there is a reward in gratitude to the time that you have given us to answer all the questions.

[ET and VN] As for the first call, each participant will be given [ET: will be given Birr 100 or an equivalent Mobile card; VN: will be given VND 50,000 or a mobile card] which is the approximate equivalent to the value (cost) of the present we used to give to you when we visited you in person.
[PE and IN] This time, as it is not possible to visit the participants in their homes, we will not be able to personally carry and deliver any presents, so, only for this time all participants will be given [PE: S /. 50 soles, which is the approximate equivalent to the value (cost) of the present], [IN: Rs. 600, which is the approximate equivalent to the value (cost) of the present] as we anticipated to you in the previous call.

**Q.1**	**SAY**: [YL Child´s name], Do you agree? Will you give me your consent?<br>01=Yes -> Go to next section (Section 2. COVID-19)<br>00=No	O No O Yes
**Q.2**	**SAY**: Why don't you want to participate?<br><br>01= doesn´t have time/can/t do it, because he/she has to go to study… to work, etc.” -> Go to Q.3<br><br>02=he/she has doubts and/or don´t trust regarding the information they will give -> Go to Q.4<br><br>03: If the justification given by the person for Not Participating, (in this first call), is linked to a very delicate situation, a Health, Economic or other type of emergency (specifying whether it is personal or related to the family) -> Go to Farewell F3	[__ __]
**Q.3**	**ENUMERATOR:** Explain that you can call again the day and time that is more convenient for he/she…and also, mention again that it will be a conversation of no more than 45 minutes…<br>**SAY**: Would you like to participate? Do you give us your consent to go ahead?<br><br>01=Yes -> **ENUMERATOR**: Schedule Time for the call -> Go to Farewell F2<br>00=No -> **ENUMERATOR**: take note of the reason -> Go to Farewell F1	O No O Yes

	Q.4	**ENUMERATOR**: Explain again the Purpose of the conversation ..., the confidentiality of what they tell you and the importance that it has, (for the future of the Health system in our country), that they share their experience along with the experience of other young people of the Young Lives study during the “Coronavirus” the Covid-19 pandemic<br>**SAY**: What do you think? Would you like to participate?<br><br>01=Yes-> Go to next section (Section 2. COVID-19)<br>00=No -> Go to Farewell F1	O No O Yes
**F1**		**SAY**: YL Child´s name don´t worry, your decision is absolutely free and voluntary, we understand and respect it. It is necessary that you know that choosing not to participate in this survey doesn´t affect at all your link with Young Lives because you are still an important part of the study until you decide. That is why is important and necessary that you know that [between November and December, DATE OF PHONE SURVEY THIRD CALL], I will call you again to check how you and your family are doing. This is going to be a very short call, no more than 15 minutes. As always, the survey will be done with your consent and on the day and time that is most convenient for you according to your activities. I will give you a much broader and more detailed explanation so that you can freely decide whether or not you want to participate in the aforementioned survey.At the moment, I can tell you that it has been very good for me to meet and talk with you.Thank you very much for your attention and remember that in [between November and December, DATE OF PHONE SURVEY THIRD CALL], I am calling you again.	
**F2**	**F2a**	Is there a day of the week that usually works better for you?<br>**ENUMERATOR**: Mark all that apply	
		01 Monday	☐
		02 Tuesday	☐
		03 Wednesday	☐
		04 Thursday	☐
		05 Friday	☐
		06 Saturday	☐
		07 Sunday	☐
	**F2b**	When is it easier to reach you?<br>**ENUMERATOR**: Read options and mark all that apply	
		01=Mornings	☐
		02=Afternoons	☐
		03= Evening (after dinner)	☐
		Other comments: _______________	[____]

	**F2c**	**SAY**: You will remember that whenever we have visited you, at the end of the interview, in gratitude to the time that you have given us to answer all the questions, we have given you a [**PE**: a small present, e.g. backpack, watch, mini radio, etc.); **ET**: a small amount of money or transferred mobile card; **IN**: money purse, vanity bag, etc; **VN**: a small amount of money], right?<br><br>[ET and VN] As for this call and the previous one, each participant will be given [ET: will be given Birr 100 or an equivalent Mobile card; VN: will be given VND 50,000 or a mobile card] which is the approximate equivalent to the value (cost) of the present we used to give to you when we visited you in person and in gratitude to the time that you have given us.<br><br>[PE and IN] As it is not possible to visit the participants in their homes, we will not be able to personally carry and deliver any presents, so, only for this time, all participants will be given [PE: S /. 50 soles, which is the approximate equivalent to the value (cost) of the present], [IN: Rs. 600, which is the approximate equivalent to the value (cost) of the present] , as we anticipated to you in the previous call.<br><br>Ok, thanks a lot for now. I will call you again at the agreed time.	[_________]
**F3**		**ENUMERATOR**: collect and write down all the information you can, important details that exactly describe the problem/situation (… ask how they think it could be solved, if they have done any paperwork or management…, or if they are doing something to solve it…, what is their greatest difficulty, etc.)**SAY**:I will present your case, will consult with the supervisors and with the people in charge of the Young Lives study, to find out how or who could help them directly. * According to the case and according to the time, set the day/date on which you would be giving the answer.**ENUMERATOR**: Follow up on the case, and it is probable, (depending on the circumstances), that only when everything is "apparently solved" or in "the process of being solve", in a conversation with he/she you will be able to mention the compensation of [PE: S/. 50 in total; ET: 100 BIRR per survey call; IN: 600 INR in total; VN: 50,000 VND per survey call] as a way to show gratitude for their time, if they decide to participate in the CALL 2 survey and to inform them about the future survey that will take place [between November and December, DATE OF PHONE SURVEY THIRD CALL].If respondent sounds desperate/ suicidal please refer to the manual for further instructions on how to proceed.	[_________]

Section 2. COVID-19: Behaviours and Risk Perceptions

Behaviours and preventive measures adopted

SAY: We would like to ask you few questions about your personal behaviors since the outbreak of coronavirus/COVID-19.

**Q.1**	In the past 7 days, on how many days did you stay at home all day, without going out at all?<br><br>-79=NK<br><br>[**CAPI:** if 0-6 or -79 ask Q.2, if Q.1=7 Go to Q.3]<br><br>[**ENUMERATOR**: if the respondent say "Don't know" ask him/her for his/her best estimate	[__]
**Q.2**	Why did you leave the house during the past 7 days?<br><br>**ENUMERATOR**: please **READ** the alternatives and enter the corresponding responses<br><br>00=No<br>01=Yes<br>88=NA	
01	To buy food	O No O Yes O N/A
02	To buy medicine; visit doctor	O No O Yes O N/A
03	To to go the bank	O No O Yes O N/A
04	To exercise, walk the dog	O No O Yes O N/A
05	To take care of a household member	O No O Yes O N/A
06	To work or work related reasons, with permission	O No O Yes O N/A
07	To work or work related reasons, without permission	O No O Yes O N/A
08	To go to church/mosque/temple/funerals/weddings	O No O Yes O N/A
09	Recreation, meeting friends and family	O No O Yes O N/A
10	To allow my children to go out	O No O Yes O N/A
11	Other, specify [_________________________]	O No O Yes O N/A
**Q.3**	How often have you done any of these things to prevent the infection, in the past 7 days?<br><br>00=Never<br>01=Sometime<br>02=Always<br>79=RTA<br><br>**ENUMERATOR**: Please **read** each statement. Note that the YL Child should report about his/her own behaviors rather than what other family members did	
01	Wash your hands with soap more often than you used to	[__ __]
02	Avoid handshakes/ physical greetings	[__ __]

03	Avoid groups meeting such as family gatherings, parties; going to church/temples, funerals/marriages etc.	[__ __]
04	Wear a face mask when outside	[__ __]
05	Keep a distance of at least 1-2 meters from other people	[__ __]
06	Wear gloves when outside	[__ __]
07	Wear a facial shield/protector when outside	[__ __]
**Q.4**	Have you done anything else to prevent catching the infection or spreading it to others? For example changing your behaviour, eating certain food or drink or avoiding certain things?<br><br>**ENUMERATOR**: Please do <u>not</u> read, just mark with "x" all the ones mentioned. Please note that the YL child should report about his/her own behaviors rather than what other family members did	
01	Cancel travel plans	☐
02	Stock up on more food than normal	☐
03	Reduce the number of times you go to the market/grocery store	☐
04	Work from home	☐
05	Shower immediately upon entering the house	☐
06	Take off all clothes that were worn outside	☐
07	Drinking lemon	☐
08	Adding hot pepper to food	☐
09	Adding garlic/ginger/turmeric to food or eating garlic	☐
10	Hot bath when you come in from being out	☐
11	Going outside into the sun	☐
12	Any other herbal medicine/local prevention/homeopathic medicine	☐
13	Avoiding congested transport systems	☐
14	Using hand sanitizers more often	☐
15	Other, specify	☐

Risk perception and attitude to risk

**Q.5**	Do you think your chance of getting infected with Corona is low, medium, or high, or do you have no risk at all?<br><br>00=No risk<br>01=Low<br>02=Medium **-> Skip to Q.7**<br>03=High **-> Skip to Q.7**<br>77=NK **-> Skip to Q.7**<br><br>**ENUMERATOR:** Please, if the YL Child reported to have (or she/he belives to have) already got it, ask her/him to think about the chance to get it again.	[__ __]
**Q.6**	Why do you think you are at low/not at risk?<br><br>**ENUMERATOR**: Do **not** read the alternatives. Mark all that apply	
01	I'm young	☐
02	God protect me	☐
03	The hot/wet weather	☐
04	I haven't travelled	☐
05	COVID is a lie to try to get money	☐
06	I don't know anyone with COVID-19	☐
07	I'm not white or chinese	☐
08	COVID is not in my country region / continent/ community	☐
09	I already got it	☐
10	Other (specify)	[________________]

SAY: Now, I would like to understand what is your general attitude to risk

**Q.7**	Suppose I offered you a lottery ticket that wins 150 Birr/620 INR/155 Soles/600,000 VND with 50-50 chance. How much would you be willing to pay for such a ticket?<br><br>**ENUMERATOR**: if the [YL Child] seems not to understand please SAY "50-50 chance means that you have an equal chance to win the lottery or to not get anything and waste what you pay for the ticket". If the [YL Child] say s/he would not be willing to pay for a lottery ticket or is not interested in playing the lottery, please code as 0.<br>77=NK	[_____]

SAY: I am going to read you now a list of five statements that people like you have done or experienced during the lockdown period.

(CAPI: This questions apply ONLY to PE and IN): Sample is split into Group A and Group B. Group A gets Q8a only and Group B gets Q8b only.
Group A answer Q.8a and then Go to the next section; Group B answer Q.8b and then Go to the next section).

**Q.8a**	**SAY**: Now I'm going to read you 5 statements. Please listen to me very carefully and tell me how many of the following things you have done, or happened to you. You don't have to tell me about each one, only how many of the 5 statements are true for you.<br>An easy way to keep score is with the fingers of your hand. I mean, close your fist, and as I read, raise a finger every time you agree. Then at the end we can check how many fingers you have raised<br><br>**ENUMERATOR**: Make sure the participant has heard the list and repeat it if necessary.<br>**ENUMERATOR**: When you finish mentioning the statements, ask the young person many fingers s/he has raised. Record the number that the respondent tells you. [0-5]<br>1. During the lockdown I was able to spend more free time than previously doing exercise<br>2. I felt much closer to my family during the lockdown than I did before<br>3. During the lockdown I started drinking more alcohol than usual<br>4. During the lockdown there were more arguments in our household than there were before<br>5. I was physically hurt more often by someone in my household during the lockdown	[__ __]

**Q.8b**	**SAY**: Now I'm going to read you 4 statements. Please listen to me very carefully and tell me how many of the following things you have done, or happened to you. You don't have to tell me about each one, only how many of the 4 statements are true for you.<br>An easy way to keep score is with the fingers of your hand. I mean, close your fist, and as I read, raise a finger every time you agree. Then at the end we can check how many fingers you have raised<br><br>**ENUMERATOR**: Make sure the participant has heard the list and repeat it if necessary.<br>**ENUMERATOR**: When you finish mentioning the statements, ask the young person many fingers s/he has raised. Record the number that the respondent tells you. [0-4]<br>-<br>1. During the lockdown I was able to spend more free time than previously doing exercise<br>2. I felt much closer to my family during the lockdown than I did before<br>3. During the lockdown I started drinking more alcohol than usual<br>4. During the lockdown there were more arguments in our household than there were before	[__ __]

Section 3. Socio-economic status

SAY: Now I am going to ask some questions about the place where you live.

**Q.1**	How many people, including yourself, are currently living with you?<br><br>**ENUMERATOR**: Enter the number of people currently living in the same household as the YL Individual, YL individual included. Please remember that a household is defined as a group of individuals that meets three conditions: 1. live under the same 'roof' or within the same compound/homestead/stand; 2. share food from a common source at least once a day; 3. they contribute to or share in, a common resource pool, aside from food and shelter.	[__ __]
**Q.2**	**SAY**: How many rooms are there in the house (excluding kitchen, bathroom, garage) ?<br><br>**ENUMERATOR**: Do not include kitchen, bathrooms, passage, garage, warehouses or rooms divided with non-permanent items such as a curtain.<br><br>77=NK	[__ __]
**Q.3**	Do you have a separate kitchen/cooking area?<br><br>00=No<br>01=Yes<br>77=NK	O No O Yes O NK
**Q.4**	Do you have access to electricity?<br><br>00=No<br>01=Yes<br>77=NK	O No O Yes O NK
**Q.5**	Is your dwelling separated from the outside by walls on all sides?	O No O Yes O NK
**Q.6**	Is your dwelling completely covered by a roof?	O No O Yes O NK
**Q.7**	What is the main source of drinking water for members of your household?<br><br>Enter codes from **CODEBOX #1**	[__ __]<br>Specify__________

**Q.8**	What kind of toilet facility does your household use?<br><br>Enter codes from **CODEBOX #2**	[__ __]<br>Specify___________

**Q.9**	Do you always have access to the following when you need it:<br><br>**ENUMERATOR**: Read answer options aloud before reading each entry 01-03<br><br>01 = Yes<br>02 = No<br>77=NK	
01	Clean water for washing hands	○ No ○ Yes ○ NK
02	Soap for washing your hands	○ No ○ Yes ○ NK
03	Hand sanitizer	○ No ○ Yes ○ NK

SAY: I am now going to ask you whether the household owns specific items.
FIELDWORKER: Ask about each item.

ALL COUNTRIES

	**Household Items**	**Q.10**<br>Does anyone in the household own this item?<br><br>Tick in the box if anyone owns it.<br><br>If NO tick ► Skip to next row
1	Working television	☐
2	Working radio	☐
3	Working smartphone	☐
4	Access to internet at home (through computer)	☐

Ownership of Saving accounts (FOR OC)

SAY: If someone wants to keep money at a bank, he/she can open a savings account. The money in the savings account is yours. Sometimes, employers open savings account for their employees to deposit their payments.

**Q.11oc**<br><br>**(CAPI: ET, PE, IN)**	Do you or your partner currently own a bank account?<br><br>00 = No **-> Skip to Section 4. EconChanges**<br>01= Yes, I have a bank account<br>02= Yes, my partner has a bank account<br>03=Yes, we both have bank accounts<br>77=NK<br>79=RTA	[__ __]

**Q.11oc (CAPI: VN)**	Do you or someone else in your household own a bank account?<br><br>00= No **-> Skip to Section 4. EconChanges**<br>01= Yes, I have a bank account<br>02= Yes, someone else in my household has a bank account<br>03= Yes, myself and someone else in my household has a bank account<br>77=NK<br>79=RTA	[__ __]
**Q.12oc**	[CAPI: This question is for PE only]<br>In which institution do you or your partner keep your bank account(s)?<br>Allow for up to 3 answers.<br><br>01 = Banco de la Nación<br>02 = Commercial Bank<br>03 = Financial Firm<br>04 = Municipal savings banks<br>05 = Rural savings banks<br>06 = Edpymes<br>07 = Other (specify) ____________________<br>77=NK<br>88=NA<br>79=RTA	1. [__ __]<br>2. [__ __]<br>3. [__ __]

Ownership of Saving accounts (for YC)

SAY: If someone wants to keep money at a bank, he/she can open a savings account. The money in the savings account is yours. Sometimes, employers open savings account for their employees to deposit their payments.

**Q.11yc**	Do you or someone else in your household own a bank account?<br><br>00= No **-> Skip to Section 4. EconChanges**<br>01= Yes, I have a bank account<br>02= Yes, someone else in my household has a bank account<br>03= Yes, myself and someone else in my household has a bank account<br>77=NK<br>79=RTA	[__ __]

**Q.12yc**	[CAPI: This question is for PE only]<br>In which institution do you and/or someone else in your household keep your bank account(s)?<br>Allow for up to 3 answers.<br><br>01 = Banco de la Nación<br>02 = Commercial Bank<br>03 = Financial Firm<br>04 = Municipal savings banks<br>05 = Rural savings banks<br>06 = Edpymes<br>07 = Other (specify) ____________________<br>77=NK<br>88=NA<br>79=RTA	1. [__ __]<br>2. [__ __]<br>3. [__ __]

Section 4. Recent life history and Economic changes

4.1 Recent life history

SAY: I am now going to ask you to think about your current circumstances and how and why they might have changed over recent periods.

**Q.1**	How would you describe the household you were living in?<br><br>**FIELDWORKER:** Please read the alternatives<br><br>01=Very rich<br>02=Rich<br>03=Comfortable – manage to get by<br>04=Struggle – never have quite enough<br>05=Poor<br>06=Destitute	
01	Currently	[__ __]
02	Before [date of Outbreak], when the COVID crisis started	[__ __]

**Q.2**	Have the total household expenses been affected by the current COVID-19 pandemic?<br><br>00=No<br>01= Yes, they have gone up<br>02=Yes, they have gone down<br>77=NK	[__ __]
**Q.3**	During the past month, did the **total (monthly) income of your household** change compared to how it was before [date of outbreak], i.e. when the pandemic started?<br><br>00=No<br>01= Yes, it has gone up<br>02=Yes, it has gone down<br>77=NK	[__ __]

4.2 Economic Shocks since the COVID-19 Outbreak

SAY: Now I am going to ask you about the most important events and changes that have happened (that negatively affected the household economy) since [DATE OUTBREAK]

CAPI: Add a flag for those households that in Call 1 reported:
1. At least one household member (including the YL respondent) lost her/his job, was suspended without payment/had their salary cut or lost all or most of their income from their own business

EVENTS		Q.4 Has any of the following events affected your household since [DATE OUTBREAK]?00=No01=Yes77=NK**ENUMERATOR**: **read** the events one by one. Make sure to remind to the respondent that this question refer to shocks happened since the outbreak.
**1**	Job loss	○ No ○ Yes ○ NK
**2**	Some household members not received payment/full payment since [DATE OUTBREAK]	○ No ○ Yes ○ NK
**3**	Nonfarm business closure	○ No ○ Yes ○ NK
**4**	Less work, less or no clients in a nonfarm family business	○ No ○ Yes ○ NK
**5**	Disruption of farming, livestock, fishing activities, and supply chain disruption	○ No ○ Yes ○ NK
**6**	Increase in price of farming/business inputs	○ No ○ Yes ○ NK
**7**	Fall in the price of farming/business output	○ No ○ Yes ○ NK
**8**	Increase in price of major food items consumed	○ No ○ Yes ○ NK
**9**	Illness, injury, or death of income earning member of household	○ No ○ Yes ○ NK
**10**	Theft/looting of cash and other property	○ No ○ Yes ○ NK
**11**	New health expenses	○ No ○ Yes ○ NK
**12**	New expenses: for example expenses required by the employer to continue working: buying a laptop, hire internet, etc	○ No ○ Yes ○ NK
**13**	Locusts (**CAPI**: ET only)	○ No ○ Yes ○ NK
**14**	Burning down of property by riot/violence (Oromia) (CAPI: ET only)	○ No ○ Yes ○ NK
**15**	Violence/riot related injuries/loss of life (Oromia) (CAPI: ET only)	○ No ○ Yes ○ NK
16	Violence related arrest of family members (CAPI: ET ONLY)	○ No ○ Yes ○ NK
17	Flooding (CAPI: ET only)	○ No ○ Yes ○ NK

CAPI: Enable Q.5 if YL child answered Yes to at least one of the events mentioned in Q.4

	REACTIONS	**Q.5** How did your household cope with this/these events that negatively affected your household since [DATE OUTBREAK]? **ENUMERATOR**: Do **not read** the alternatives. Mark with an "x" all options that apply.
**1**	Sale of assets	☐
**2**	Engaged in additional income generating activity	☐
**3**	Received assistance from friends and family	☐
**4**	Took a loan from a financial institution	☐
**5**	Credited purchases	☐
**6**	Delayed payments	☐
**7**	Sold harvest in advance	☐
**8**	Reduced food consumption	☐
**9**	Relied on savings	☐
**10**	Received assistance from NGO/Faith based organization(CAPI: ET only)	☐
**11**	Took an advance payment from employer	☐
**12**	Received assistance from Government	☐
**13**	Was covered by insurance policy	☐
**14**	Did nothing	☐
**15**	Took any other type of informal loans	☐
**16**	Migrate	☐
**17**	Switched to another job	☐
**18**	Other, Specify	☐

CAPI: Q.5a and Q.5b applies to PE only

**Q.5a**	Since (Outbreak date), did anyone in your household withdraw money from their CTS ("Compensacion por tiempo de servicios") account?<br><br>01=Yes<br>00=No<br>77=NK	○ No ○ Yes ○ NK
**Q.5b**	Since (Outbreak date), did anyone in your household withdraw money from their pension fund ("AFP")?<br><br>01=Yes<br>00=No<br>77=NK	○ No ○ Yes ○ NK

SAY: I am going to read you now a list of five statements that people like you have done or experienced during the lockdown period.
(CAPI: This questions apply ONLY to PE and IN): Sample is split into Group A and Group B. Group A gets Q6a only and Group B gets Q6b only.

Group A answer Q.6a and then Go to the next section; Group B answer Q.6b and then Go to the next section).

**Q.6a**	**SAY**: Now I'm going to read you 4 statements. Please listen to me very carefully and tell me how many of the following things you have done it, or it happened to you. You don't have to tell me about each one, only how many of the 4 statements are true for you.<br>An easy way to keep score is with the fingers of your hand. I mean, close your fist, and as I read, raise a finger every time you agree. Then at the end we can check how many fingers you have raised<br><br>**ENUMERATOR**: Make sure the participant has heard the list and repeat it if necessary.<br>**ENUMERATOR**: When you finish mentioning the statements, ask the young person many fingers s/he has raised. Record the number that the respondent tells you. [0-4]<br>1. I had more caring responsibilities when the schools were closed during the lockdown<br>2. I spent much more time working to earn money during the lockdown than I normally would have<br>3. I talked on the phone to my friends much more than usual during the lockdown<br>4. During the lockdown I ran out of the money I needed for basic things more often than before	[__ __]

**Q.6b**	**SAY**: Now I'm going to read you 5 statements. Please listen to me very carefully and tell me how many of the following things you have done it, or it happened to you. You don't have to tell me about each one, only how many of the 5 statements are true for you.<br>An easy way to keep score is with the fingers of your hand. I mean, close your fist, and as I read, raise a finger every time you agree. Then at the end we can check how many fingers you have raised<br><br>**ENUMERATOR**: Make sure the participant has heard the list and repeat it if necessary.<br>**ENUMERATOR**: When you finish mentioning the statements, ask the young person many fingers s/he has raised. Record the number that the respondent tells you. [0-5]<br>1. I had more caring responsibilities when the schools were closed during the lockdown<br>2. I spent much more time working to earn money during the lockdown than I normally would have<br>3. I talked on the phone to my friends much more than usual during the lockdown<br>4. During the lockdown I ran out of the money I needed for basic things more often than before<br>5. I was physically hurt more often by someone in my household during the lockdown	[__ __]

Section 5. Food Security, Prices and Health

5.1 Food Security

**SAY**: Now I would like to ask you some questions about food. Since [Outbreak date], was there a time when:<br>00=No<br>01=Yes<br>77=NK		
**Q.1**	You or others in your household worried about not having enough food to eat because of a lack of money or other resources?	O No O Yes O NK
**Q.2**	Still thinking about the time since [Outbreak date], was there a time when you or others in your household were unable to eat healthy and nutritious food because of a lack of money or other resources?	O No O Yes O NK
**Q.3**	Was there a time since [Outbreak date] when you or others in your household ate only a few kinds of foods because of a lack of money or other resources?	O No O Yes O NK
**Q.4**	Was there a time since [Outbreak date] when you or others in your household had to skip a meal because there was not enough money or other resources to get food?	O No O Yes O NK
**Q.5**	Still thinking about the time since [Outbreak date], was there a time when you or others in your household ate less than you thought you should because of a lack of money or other resources?	O No O Yes O NK
**Q.6**	Was there a time since [Outbreak date] when your household ran out of food because of a lack of money or other resources?	O No O Yes O NK
**Q.7**	Since [Outbreak date], was there a time when you or others in your household were hungry but did not eat because there was not enough money or other resources for food?	O No O Yes O NK
**Q.8**	Was there a time since [Outbreak date] when you or others in your household went without eating for a whole day because of a lack of money or other resources?	O No O Yes O NK

5.2 Food Prices

Q.9	For each of these particular food items, can you please tell me if the price per unit has increased, decreased, or stayed in the same in comparison to before [DATE OF OUTBREAK]?<br><br>01=Increased<br>02=Decreased<br>03=Stayed the same<br>04=Item unavailable<br>-77=NK<br><br>**CAPI**: The list of food items varies across countries.	
ETHIOPIA		
01	Injera/bread (teff, maize, wheat, and/or barley)	[__ __]
02	Inset	[__ __]
03	Oil/fats/butter	[__ __]
04	Milk/cheese/yogurt	[__ __]
05	Vegetables (including onion, tomato, potato, cabbage, garlic)	[__ __]
06	Pulses (peas and beans)	[__ __]
07	Sugar	[__ __]
INDIA		
01	Rice and other cereals	[__ __]
02	Vegetables including onion	[__ __]
03	Oil, fat, ghee, butter	[__ __]
04	Milk and milk products	[__ __]
05	Meat and meat products including fish	[__ __]
06	Pulses	[__ __]
07	Any sugar, honey, sweets, sugary sweet drinks	[__ __]
08	Eggs	[__ __]
PERU		
01	Bread	[__ __]
02	Rice	[__ __]
03	Oil	[__ __]
04	Milk	[__ __]
05	Chicken	[__ __]
06	Pulses	[__ __]
07	Sugar	[__ __]
08	Fruits	[__ __]
VIETNAM		
01	Rice	[__ __]
02	Chicken	[__ __]
03	Fish	[__ __]
04	Milk	[__ __]
05	Vegetables	[__ __]
06	Fruits	[__ __]
07	Sugar	[__ __]

5.3 Individual Health

SAY: Now I would like to ask some questions about your health. But before doing it I would like to ask you about your family:

[**CAPI**: create a flag for those households were
1.One or more hh members were reported as infected by COVID in call 1
2. One of the hh members died from covid as reported in call 1]

**Q.1**	Since [FIST CALL DATE] when we last spoke to you, did anyone in your household (excluding you) get sick with COVID (either confirmed by a test, or you highly suspect it because of the symptoms display).<br><br>**SAY**: Typical symptom are high temperature, continuous cough, breathing problems, chest pain, loss of smell & taste<br><br>**ENUMERATOR**: Encourage the YL Child answer this question. If theYL Child answers "I don't know", emphasize that what he seeks to capture is his/her perceptions. Repeat symptoms if necessary. If, despite the above, YL Child repeat "I don't know" please mark it and add any relevant comment.<br><br>00= No<br>01= Yes, one person<br>02= Yes, more than one person<br>77=NK _Any comment? Specify {_______________}	[__ __]

SAY: Now I would like to ask some questions about your health.

**Q.2**	Since [FIRST CALL DATE], have you been tested for COVID? Which type of test did you take?<br>[**CAPI**: this question should be answer to everyone regardless of what they answer in Q.1]<br><br>00 = No test **->Skip to Q.5**<br>01 = Quick test (blood drop)<br>02 = Nose and throat sample<br>03 = Other, specify {____________________}<br>04=Both types of test<br>77 = NK **->Skip to Q.5**	[__ __]
**Q.3**	How many times have you been tested, since [FIRST CALL DATE]?	[__ __]
**Q.4**	Did any of those tests turn to be positive?<br>00=No<br>01=Yes ->Skip to Q.6<br>77=NK	○ No ○ Yes ○ NK

**Q.5**	Have you been infected or you think you might have been infected since [FIRST CALL DATE] when we talked last time?<br><br>**SAY**: Typical symptom are: high temperature, continuous cough, breathing problems, chest pain, loss of smell & taste<br><br>00=No -> Go to the next section<br>01=Yes<br>77=NK _Any comment? Specify {_______________}<br><br>**ENUMERATOR**: Encourage the YL Child to answer this question Yes or No. If the YL Child answers "I don't know", emphasize that what he seeks to capture is his/her perceptions. Repeat symptoms if necessary. If, despite the above, YL Child repeat "I don't know" please mark it and add any relevant comment.<br><br>**CAPI**: add a validation check. If Q.2=01,02,03,04 AND Q.4=01, then it should be Q.5=01; If Q.2=00, 77 then Q.5 can assume any value	O No O Yes O NK
**Q.6**	Did you receive treatment, since [FIST CALL DATE] ?<br><br>01 = Yes<br>00 = No -> **Skip to Q.8**<br>77=NK **-> Skip to Q.8**	O No O Yes O NK
**Q.7**	What type of treatment did you receive?<br><br>[**CAPI**] : Once answered go to Q.9]<br><br>**ENUMERATOR:** do not read the options. If the YL Child report more than one treatment please ask about the main one.<br><br>01 = Instructions by phone/WhatsApp<br>02 = Was under observation at home<br>03 = Hospitalized in private clinic<br>04 = Hospitalized in public hospital<br>05 = Hospitalized in health post (not relevant for IN)<br>06 = Was in Intensive Care Unit<br>07= Self-prescribed traditional medicine<br>08=Traditional medicine prescribed by local traditional doctors or healer<br>09=Self-prescribed modern medicine<br>10= Prescribed modern medicine by doctor<br>11= Other, specify	[__ __]

**Q.8**	Why didn't you receive treatment?<br><br>**ENUMERATOR:** do not read the options. If the YL Child report more than a reason please ask about the main one.<br><br>00= Not tested<br>01 = Rejected by the health center<br>02 = No health center where we live<br>03 = Told to stay at home<br>04 = Told that treatment was not required<br>06= I rejected seeking treatment/not interested<br>07= Family advise me not to take treatment<br>08= Fear of isolation from the community<br>09= Fear of being culprit of bringing the virus to the community<br>10= Belief illness can be cured at home.<br>11 = Other, specify	[__ __]
**Q.9**	How are you now?<br><br>01 = Fully recovered<br>02 = Recovered, but still have some problems<br>03 = Still ill	[__ __]

Section 6. Current Education

SAY: Now I would like to ask you some questions about your **current educational status**.

**Q.1**	PE: Are you currently enrolled in full-time education?<br>VN: Are you currently enrolled in full-time education/Are you planning to be enrolled in full-time education in September?<br>ET: Are you currently enrolled in full-time education/Are you planning to be enrolled in full-time education when the school/educational institutes reopen?<br>IN: Are you planning to be enroleld in full-time education in the next academic year (mid-August/September)?<br><br>ENUMERATOR (ET only): please note that if the interview happens before the beginning of the academic year in September, you should ask whether the [YL Child] plans to be enrolled in full-time education starting in September. If the interview happened later, and the academic year already started, then you should refer to the current enrolment.<br><br>**ENUMERATOR**: this includes online and blended sessions<br><br>00 = No ► Skip to Q.5<br>01 = Yes, attending regularly/ IN: Yes, I'm planning to attend regularly<br>02 = Yes, but attending irregularly/IN: Yes, even if I will be able to attend irregularly<br>03 = Never attended ► Skip to next section<br>04 = Registered for this school year but classes are suspended/ IN: Yes, I'm registered for the next school year but classes are suspended<br>77 = NK ► Skip to next section<br>79 = Refused to answer ► Skip to next section	[__ __]
**Q.2**	PE: What is the education level that you are currently studying (ET, VN:or you will be studying in September; IN: you will be studying in August/September) ?<br><br>**ENUMERATOR**: If the [YL Child] reports primary or secondary education level, ask him/her to specify what grade. Enter code from CODEBOX #3	[__ __]
**Q.3**	What type of school, institute or university (PE: are you currently attending) (ET/VN:or you will be attending in September; IN: will you be attending/ planning to attend in August/September) ?<br><br>**ENUMERATOR**: Enter code from CODEBOX #4<br><br>**CAPI**: Skip pattern varies across countries<br>**ET**: if Q.2 (CODEBOX#3)= 21;22;24; 25;32-40; 43-48; Ask Q.4 and ► Skip to Q.10; if Q.2=00-14;16-17;26-31; 41-42; 88, 77 ►Skip to Q.10<br>**IN YC:** if Q.2 (CODEBOX#3)= 13-15 Ask Q.4 and ► Skip to Q.10; if Q.2= 00-12;28,29,77,79►Skip to Q.10<br>**IN OC**: if Q.2 (CODEBOX#3)= 09-10 Ask Q.4 and ► Skip to Q.10; if Q.2= 00;01-0-6,08,77,79►Skip to Q.10<br>**PE:** if Q.2 (CODEBOX#3)= 13-16, 19, 21, 22 Ask Q.4 and ► Skip to Q.10; if Q.2=00-11; 17, 18, 20, 77, 88►Skip to Q.10<br>**VN:** if Q.2 (CODEBOX#3)= 13-16 Ask Q.4 and ► Skip to Q.10; if Q.2=00-12,17,28,29,50,77,79►Skip to Q.10	[__ __]

**Q.4a**	What major?<br>**ENUMERATOR**: Write the name of the major. Please look for the right code at the end of the call and add it in the line below.	[________________]
**Q.4b**	**ENUMERATOR**: Enter code from CODEBOX #5<br><br>-> ET, IN, VN Go to Q. 10<br>-> PE: Go to Q.4c and then go to Q.10	[__ __]
**Q.4c**	[CAPI : PE only]<br>Do you plan to continue your studies in the next 12 months?<br><br>00= No<br>01= Yes<br>77 = NK<br>88 = NA<br><br>-> Go to Q.10	[__ __]
**Q.5**	What is the (highest) education level that you completed?<br><br>**ENUMERATOR**: If the [YL Child] reports primary or secondary education level, ask him/her to specify what grade. Enter code from CODEBOX #3	[__ __]
**Q.6**	What type of school, institute or university was the last one you attended?<br>**ENUMERATOR**: Enter code from CODEBOX #4CAPI: Skip pattern varies across countries<br>**ET**: if Q.5 (CODEBOX#3)= 21;22;24; 25;32-40; 43-48; Go to Q.7 ; if Q.5= 00-14;16-17;26-31; 41-42; 88, 77 ►Skip to Q.8<br>**IN YC**: if Q.5 (CODEBOX#3)= 13-15 Go to Q.7 ; if Q.5= 00-12;28,29,77,79►Skip to Q.8<br>**IN OC**: if Q.5 (CODEBOX#3)= 09-10 Go to Q.7; if Q.5= 00;01-0-6,08,77,79►Skip to Q.8<br>**PE**: if Q.5 (CODEBOX#3)= 13-16, 19, 21, 22 Go to Q.7 ; if Q.5=00-11; 17, 18, 20, 77, 88►Skip to Q.8<br>**VN**: if Q.5 (CODEBOX#3)= 13-16 Go to Q.7; if Q.5=00-12,17,28,29,50,77,79►Skip to Q.8	[__ __]
**Q.7a**	What major?<br>**ENUMERATOR**: Write the name of the major. Please look for the right code at the end of the call and add it in the line below.	[________________]
**Q.7b**	**ENUMERATOR**: Enter code from CODEBOX #5	[__ __]
**Q.8**	When was the last time you attended full-time education?<br><br>**ENUMERATOR**: if the [YL Child] attended full-time education for the last time in the current year (2020) please ask and report in what month.<br><br>01-12= Jan-Dec 2020 ► Go to Q.9<br>13 = 2019 ► [ET,IN,VN skip to next section], PE: skip to Q.12<br>14 = Before 2019 ► [ET,IN,VN skip to next section], PE: skip to Q.12<br>77 = NK ► [ET,IN,VN skip to next section], PE: skip to Q.12<br>79 = Refused to answer ► [ET,IN,VN skip to next section], PE: skip to Q.12	[__ __]

**Q.9**	Why did you stop attending full-time education?<br>**ENUMERATOR**: do not read the options.<br><br>01 = Because of the quarantine, my school cancelled classes<br>02 = Because of the quarantine, my school offers virtual classess but I don't have the equipment<br>03 = Because of the quarantine, I couldn't pay the fees<br>04 = Because of the quarantine, I had to look for a job<br>05 = Because of the COVID, someone in the household became ill<br>06= The course has finished<br>07 = Other, specify (related to COVID or quarantine) : ______<br>08 = Other Specify: ______	[__ __]
**Q.10**	Since the lockdown/ Response to COVID (ET), have you engaged in any of these education or learning activities?<br>**ENUMERATOR**: read all options and mark all that apply	
	01 = Attend classes in person	☐
	02 = Virtual classes with lesson teacher (laptop/computer/smartphone)	☐
	03= Completed assignments provided by the teacher	☐
	04= Watched educational TV programs	☐
	05= Listened to educational programs on radio	☐
	06= Used learning apps	☐
	07 = Other Specify: ______	[____________]
**Q.11**	During the lockdown/ Response to COVID (ET), how were you in contact with your teachers?<br>**ENUMERATOR**: read all options and mark all that apply	
	00= I have never been in contact -> Go to next section	☐
	01 = SMS	☐
	02 = Online applications	☐
	03= Email	☐
	04= Telephone (audio)	☐
	05= WhatsApp/Telegram (ET)	☐
	06= Facebook	☐
	07 = Other Specify: ______	[____________]
**Q.12**	[CAPI=PE only]What do you think will be your **main activity** in the next 12 months?**ENUMERATOR**: Read all options and mark the alternative that applies the most	
	01= Studying	
	02= Looking for a job	
	03= Work in a family business	
	04= Start my own business	[__ __]
	05= Work as employees/wage worker	
	06=Help in home activities / taking care of family members	
	07=Others	

Section 7. Time Use

SAY: Now I want you to think about how you spent your time during a typical weekday when the country was in full lockdown in [MONTH_COUNTRY_SPECIFIC]/ ET: during the Response to COVID-19]

Q.1	**SAY**: Now I want you to think about how you spent your time during a typical weekday when the [ET, IN, PE: country was in full lockdown in] [VN: when the city/province is/was in social distancing] [IN, PE: MONTH_COUNTRY_SPECIFIC] / ET: when the Response to COVID-19 started]? [CAPI: do not specify any time for VN]<br><br>Do you agree, partially agree or disagree with the following statement:<br><br>01=Agree<br>02=Partially agree<br>03=Disagree<br>77=NK<br>88=NA	
01	I spent more time taking care of children than before	[__ __]
02	I spent more time on household chores than before	[__ __]
03	I spent more time working at home/from home than before	[__ __]
04	I spent more time working outside home than before	[__ __]
05	I spent more time studying/learning	[__ __]
06	I spent more time working in family business than before	[__ __]
07	I spent more time sleeping	[__ __]
08	I spent more time playing/doing nothing	[__ __]

Section 8. Employment and Earnings

8.1 Labour Force participation

SAY: Now I would like to ask you about your current and previous paid and unpaid work activities. This includes work done inside and outside the household, and EXCLUDES non-working activities (e.g. housewife, student, taking care of other household members, such as children, disabled, illed, elderly). Working as housemaid or childcare (not for relatives) should be considered as working activity.

**Q.1**	At any time during the last 12 months, did you work for at least 1 hour, in your own business, for a household member or for someone else?<br><br>00=No ► Skip to Q.6<br>01=Yes	○ No ○ Yes
**Q.2**	Thinking back to January-February (for PE)/December-February (for IN, VN)/ January-mid-March (for ET), just before the COVID crisis began, were you working for at least 1 hour, in your own business, for a household member or for someone else?<br><br>00=No<br>01=Yes	○ No ○ Yes
**Q.3**	During the **last week** (from Monday through Sunday), did you work for at least 1 hour, in your own business, for a household member or for someone else?<br><br>00=No<br>01=Yes ► Skip to "Section 8.2"	○ No ○ Yes
**Q.4**	Do you currently have a job even though you did NOT work **last week** (from Monday through Sunday)?<br><br>00=No ► Skip to Q.6<br>01=Yes	○ No ○ Yes
**Q.5**	Why were you not able to work as usual?<br>**FIELDWORKER**: Do not read the options, mark all that applies<br><br>[**CAPI**: Once this question is answered ► Skip to "Section 8.2"]	
01	Temporarily laid off for reduction of staff/less business due COVID-19	☐
02	Workplace closed and cannot work from home	☐
03	Reduction in working hours due to less business	☐
04	Temporarily absent	☐
05	Sick or not feeling well	☐
06	Need to care for ill relative	☐
07	Has to care of children because school is closed	☐
08	Has to help children with homework because school is closed	☐
09	More domestic work because of the outbreak	☐
10	More time needed to buy food and cook	☐
11	Abandoned the job to protect myself and the household from COVID-19	☐
12	Others	☐

**Q.6**	Did you look for work **last week** (from Monday through Sunday)?<br><br>00=No<br>01=Yes ► If Q.1=00, skip to Subjective Wellbeing and Mental Health section;<br>Q.1=01; Q.2=01; Q.3=00; Q.4=00 -> Go to Section 8.3 and Section 8.4<br>Q.1=01; Q.2=00; Q.3=00; Q.4=00 -> Go to Section 8.4	○ No ○ Yes
**Q.7**	What is the main reason you did NOT look for work **last week**?<br><br>**ENUMERATOR**: do not read the option. Prompt th respondent to report the MAIN reason<br><br>01=housewife/childcare<br>02=student<br>03=disabled<br>04=illness<br>05=waiting for response from potential employer<br>06=waiting for recall by previous employer<br>07=waiting for busy season<br>08=lockdown<br>09=other, specify<br><br>Once this question is answered ► If Q.1=00, skip to Subjective Wellbeing and Mental Health section;<br>Q.1=01; Q.2=01; Q.3=00; Q.4=00 -> Go to Section 8.3 and Section 8.4<br>Q.1=01; Q.2=00; Q.3=00; Q.4=00 -> Go to Section 8.4	[__ __]

CAPI: these are the 10 potential ending with instructions on which one of the next sections on labor market should be administered:

01	Q.1=00 -> Q.6==00 -> Q.7 > Section 9.1 Subjective wellbeing and mental health
02	Q.1=00 -> Q.6==01 -> Section 9.1 "Subjective wellbeing and mental health"
03	Q.1=01 -> Q.2==00 -> Q.3==00 -> Q.4==00 -> Q.6==01 -> Go to Section 8.4
04	Q.1=01 -> Q.2==00 -> Q.3==00 -> Q.4==00 -> Q.6==00 -> Q.7 -> Go to Section 8.4
05	Q.1=01 -> Q.2==00 -> Q.3==00 -> Q.4==01 -> Q.5 -> Section 8.2 and Section 8.4
06	Q.1=01 -> Q.2==00 -> Q.3==01 -> Section 8.2 and Section 8.4
07	Q.1=01 -> Q.2==01 -> Q.3==00 -> Q.4==00 -> Q.6==01-> Go to Section 8.3 and Section 8.4
08	Q.1=01 -> Q.2==01 -> Q.3==00 -> Q.4==00 -> Q.6==00 -> Q.7 -> Go to Section 8.3 and Section 8.4
09	Q.1=01 -> Q.2==01 -> Q.3==00 -> Q.4==01-> Q.5 -> Section 8.2 and Section 8.3 and Section 8.4
10	Q.1=01 -> Q.2==01 -> Q.3==01 -> Section 8.2 and Section 8.3 and Section 8.4

8.2 Main activity in the past 7 days

CAPI: This sub-section will be asked only if [YL Child] reported having worked **at least one hour in the last 7 days or has a job but couldn't work last week** (Q.3=01 **or** Q.4=01 in the "Section 8.1 Labour force participation").

SAY: Now I would like to ask you about your **most important paid or unpaid work activitities during the last 7 days**. This includes work done inside and outside the household, and EXCLUDES non-working activities (e.g. housewife, student, taking care of other household members, such as children, disabled, illed, elderly). Working as housemaid or childcare (not for relatives) should be considered as working activity. If you has a job but you couldn't work last week please still refer to the job you have to answer the following questions.

FIELDWORKER: If the YL Child report more than one activity, record the **most important** paid or unpaid work activity. The most important activity is the activity on which the [YL Child] spent more time on in the last 7 days.

**Q.1**	Description of work activity<br><br>**ENUMERATOR:** Describe the work activity.	[_____]
**Q.2**	Type of work activity<br><br>**ENUMERATOR:** Record the most important paid or unpaid work activity.<br><br>Enter code from **CODEBOX #6**	[__ __]<br><br>Specify _________
**Q.3**	Economic sector code of the job/occupation<br><br>Enter code from **CODEBOX #7**	[__ __]
**Q.4**	Who do you do this activity for?<br><br>01=Private company/enterprise or cooperative<br>02=For a household member<br>03=Other private individual/household (excluding own household)<br>04=Public sector/government<br>05=A rural public works program<br>06=Own account/self-employed (own business or farm)<br>07= Other, specify<br><br>**ENUMERATOR:** In case of more than one type of employer in the same activity, refer to the most important employer in terms of time spent.	[__ __]<br><br>Specify _________

CAPI: Q.5-Q.6 are different for IN/PE/ET and for VN

ET, IN,PE version

**Q.5**	**ENUMERATOR:** Specify the average number of weeks worked in the month.<br>Enter 01-04<br>-79=NK	[__ __]
**Q.6**	**ENUMERATOR:** Specify the average number of days per week Days per weekEnter 01-07-79=NK	[__ __]
**VN version**		
**Q.5**	**ENUMERATOR:** Specify the average number of months worked in the year.<br>Enter 01-12<br>-79=NK	[__ __]
**Q.6**	**ENUMERATOR:** Specify the average number of days worked in the month<br>Enter 0-31<br><br>-79=NK	[__ __]
**Q.7**	**ENUMERATOR:** Record the average number of hours per day.<br><br>Hours per day<br>Enter 01-24<br>-79=NK	[__ __]
**Q.8**	For how many months have you performed this activity in the last 12 months?<br><br>Months per year<br>Enter 01-12<br>-79=NK	[__ __]
**Q.9**	Which form of payment was received or is expected from this activity?<br>00=None ► Skip to Q.14<br>01= Cash<br>02= In-kind (non-cash payment) ► Skip to Q.13<br>03= Both in cash and in-kind<br>04= Debt relief<br>05=Pocket money<br>06=Other, specify<br><br>77=NK ► Skip to Q.14<br>79=Refused to answer ► Skip to Q.14	[__ __]

**Q.10**	What period of time does this payment usually cover?<br><br>**ENUMERATOR:** If payment is reported in different periods, please convert to the most convenient measure.<br><br>01= Per hour ► Skip to Q.12<br>02= Per day ► Skip to Q.12<br>03= Per week ► Skip to Q.12<br>04= Per month ► Skip to Q.12<br>05= Per year ►Skip to Q.12 [ENUMERATOR: prompt the respondents to use a different period; report yearly earning only if absolutely needed]<br>06= Per piece<br>07= Fortnightly ► Skip to Q.12<br>08= Other, specify ►Skip to Q.12<br><br>77=NK ► Skip to Q.16<br>79=Refused to answer ► Skip to Q.16	[__ __]

**Q.11**	How many pieces are produced by day?Enter number of pieces77=NK79=Refused to answer	[__ __]	
**Q.12-Q.13**	What are the net earnings per *[prefill from Q10]* from this activity?<br><br>**ENUMERATOR**: This asks for NET earnings (the sum of all wages/salaries, tips, gratuities, bonuses and the value of any in-kind payment after deducting taxes and any other work-related payments). If own business, make sure to capture profits/self-determined wage assigned to respondent from this activity, net of production costs. If in-cash or in-kind payment is non-existent, assign a zero to the corresponding payment.	**Q.12**<br><br>In cash<br>Enter in currency unit<br><br>-8888=NA<br><br>[__________]	**Q.13**<br><br>In-kind<br>Enter value in currency unit<br><br>-8888=NA<br><br>[__________]

**Q.14**	Apart from this activity, did you do another activity in the last week that was paid or is expected to be paid?<br><br>00= No ► Skip to Q.16<br>01= Yes	○ No ○ Yes

**Q.15**	We would like to ask you about your total earnings from all paid activities, including the main working activity.<br><br>What period of time is better suited to calculate your total earnings?<br><br>01= Per hour<br>02= Per day<br>03= Per week<br>04= Per month<br>05= Per year [**ENUMERATOR**: prompt the respondents to use a different period; report yearly earning only if absolutely needed]<br>07= Fortnightly<br>08= Other, specify<br><br>77=NK ► Skip to Q.17<br>79=Refused to answer ► Skip to Q.17	[__ __]
**Q.16**	What are the total earnings per [Period of time, Q.15] from all paid activities, including the main working activity?<br><br>77=NK ► Skip to Q.17<br>79=Refused to answer ► Skip to Q.17<br><br>**ENUMERATOR**: As for the main activity earnings this refer to the NET earnings (the sum of all wages/salaries, tips, gratuities, bonuses and the value of any in-kind payment after deducting taxes and any other work-related payments).	Enter value in currency unit<br><br>-8888=NA

WORKING ARRANGEMENT- ALL WORKERS

**Q.17**	Do you have health insurance paid by the employer?<br><br>00=No<br>01=Yes<br>77=NK	○ No ○ Yes ○ NK
**Q.18**	Do you have a written contract for this [MAIN ACTIVITY]?<br>00=No ► Skip to next section<br>01=Yes	○ No ○ Yes

Q.19	What is the type of contract for this [MAIN ACTIVITY]?<br>CAPI: the options are country specific	
	ETHIOPIA<br>01 = Permanent contract ► Skip to next section<br>02= Fixed term/Temporary contract<br>03= Trial period<br>04= Youth training agreements / pre-professional practices<br>05=Other, specify	[__ __]
	**INDIA**<br>01 = Permanent contract ► Skip to next section<br>02= Fixed term/Temporary contract<br>03= Probation period<br>04= Apprenticeship<br>05=Internship<br>06=Other, specify	[__ __]
	**PERU**<br>01 = Permanent contract ► Skip to next section<br>02= Fixed term contract<br>03= Trial period<br>04= Youth training agreements / pre-professional practices<br>05= Independent contract (RPH)<br>06= Special regime for the public sector (CAS)<br>07=Other, specify	[__ __]
	**VIETNAM**<br>01=Permanent contract, go to next section; ► Skip to next section<br>02=Fixed term contract;<br>03=Contract for specific work ► Skip to next section<br>04=Other, specify	[__ __]
**Q.20**	What is the duration of the contract that you have?<br>01=3 months or less<br>02=More than 3 months to 6 months<br>03=More than 6 months to 1 year<br>04=More than 1 year	[__ __]

8.3 Main activity before COVID-19

FIELDWORKER: This sub-section will be asked only if [YL Child] reported having worked **at least one hour during December-February, just before the COVID crisis began (as reported in the Labour Force Participation Section]**

[**CAPI**: Section 8.3 should be enabled only if Q.2=01 in the "Labour force participation" sub-section. If Q.2=0, skip to Section 8.4]

SAY: Now I would like to ask you about your most important paid or unpaid work activities during January-February (for PE)/December-February (for IN, VN)/ January-mid-March (for ET), just before the COVID crisis began. This includes work done inside and outside the household, and **EXCLUDES** non-working activities (e.g. housewife, student, taking care of other household members, such as children, disabled, illed, elderly). Working as housemaid or childcare (not for relatives) should be considered as working activity.

FIELDWORKER: Record the **most important** paid or unpaid work activity. The most important activity is the activity on which the [YL Child] spent more time on in the week before [OUTBREAK_DATE]

**Q.1**	Was this the same activity you did in the last 7 days? Is it with the same employer?<br><br>**ENUMERATOR**: for those who didn't work in the past 7 days but has a job please ask the respondent to refer to that job in answering this question<br><br>00=Different activity and different employer -> Go to Q.2 and continue<br>01= Same employer but different activity -> Go to Q.2 and continue (do not ask Q.5)<br>02= Same activity but different employer > Go to Q.5 and continue<br>03= Same activity same employer-> Go to Q.6	[__ __]
**Q.2**	Description of work activity<br><br>**ENUMERATOR:** Describe the work activity.	[________]
**Q.3**	Type of work activity<br><br>**ENUMERATOR:** Record the most important paid or unpaid work activity.<br><br>Enter code from **CODEBOX #6**	[__ __]<br><br>Specify<br>________
**Q.4**	Economic sector code of the job/occupation<br>Enter code from **CODEBOX #7**	[__ __]

**Q.5**	Who do you do this activity for?<br><br>01=Private company/enterprise or cooperative<br>02=For a household member<br>03=Other private individual/household (excluding own household)<br>04=Public sector/government<br>05=A rural public works program<br>06=Own account/self-employed (own business or farm)<br>07= Other, specify<br><br>**ENUMERATOR:** In case of more than one type of employer in the same activity, **refer to the most important employer in terms of time spent.**	[__ __]
**Q.6**	Which form of payment was received or is expected from this activity?<br><br>00=None ► Skip to Q.11<br>01= Cash (including pocket money)<br>02= In-kind (non-cash payment) ► Skip to Q.10<br>03= Both in cash and in-kind<br>04= Debt relief<br>05=Pocket money<br>06=Other, specify<br><br>77=NK ► Skip to Q.11<br>79=Refused to answer ► Skip to Q.11	[__ __]<br><br>Specify<br>__________
**Q.7**	What period of time does this payment usually cover?<br><br>**ENUMERATOR:** If payment is reported in different periods, please convert to the most convenient measure.<br><br>01= Per hour ► Skip to Q.9<br>02= Per day ► Skip to Q.9<br>03= Per week ► Skip to Q.9<br>04= Per month ► Skip to Q.9<br>05= Per year ► Skip to Q.9<br>06= Per piece<br>07= Fortnightly ► Skip to Q.9<br>08= Other, specify ► Skip to Q.9<br><br>77=NK<br>79=Refused to answer	[__ __]

**Q.8**	How many pieces are produced by day?<br><br>Enter number of pieces<br><br>77=NK<br>79=Refused to answer	[__ __]

**Q.9-Q.10**	What are the net earnings per [prefill from Q.7] from this activity?<br><br>**FIELDWORKER**: This asks for NET earnings (the sum of all wages/salaries, tips, gratuities, bonuses and the value of any in-kind payment after deducting taxes and any other work-related payments). If own business, make sure to capture profits/self-determined wage assigned to respondent from this activity, net of production costs. If in-cash or in-kind payment is non-existent, assign a zero to the corresponding payment.	**Q.9**<br>In cash<br><br>Enter in currency unit<br><br>-8888=NA<br><br>[__________]	**Q.10**<br>In-kind<br><br>Enter value in currency unit<br><br>-8888=NA<br><br>[__________]

**Q.11**	After the [OUTBREAK_DATE] did you move away from the village/town where your household is located?<br><br>01=Yes<br>00=No -> Skip to Section 8.4	○ No ○ Yes
**Q.12**	Why did you move?<br><br>**ENUMERATOR**: do not read the options. Mark only one. If the YL Child replies with two or more reasons aks about the main reason<br><br>01 Looking for work<br>02 Moved in with relatives<br>03 Going back to my original town<br>04 To access health services<br>05 Others, Specify _______________	[__ __]

8.4 Main activity during lockdown period

CAPI: Section 8.4 should be enabled if Q.1=01 in the "Section 8.1 Labour force participation". If Q.1=0, skip to next section
FIELDWORKER: This sub-section will be asked only if [YL Child] reported having worked **at least one hour in the past 12 months** in the Labor Force Participation Section

**Q.1**	During the lockdown/ET: COVID-19 Response, were you able to work either at your place of work or remotely? 01= Yes, working from work place -> Go to Q.302= Yes, working remotely -> Go to Q.303= Yes, combining remote work and working from workplace -> Go to Q.304= No -> Go to Q.2 and then go to Next section05= I did not have a job when lockdown/ET: COVID-19 Response started -> Go to Next Section	[__ __]

**Q.2**	Why not?<br><br>**FIELDWORKER**: Do not read the options, mark all that apply	
01	Temporarily laid off for reduction of staff/less business due covid-19	☐
02	Workplace closed and cannot work from home	☐
03	Reduction in working hours due to less business	☐
04	Temporarily absent	☐
05	Sick or not feeling well	☐
06	Need to care for ill relative	☐
07	Has to take care of children because school is closed	☐
08	Has to help children with homework because school is closed	☐
09	More domestic work because of the outbreak	☐
10	More time needed to buy food and cook	☐
11	Abandoned the job to protect myself and the household from COVID-19	☐
12	Others	☐

**Q.3**	Were/are you able to work as usual? Same number of working hours/amount of work?<br><br>**FIELDWORKER**: Do not read the options, mark all that apply	
01	Yes, same amount/hours of work	☐
02	No, reduction in hours/amount of work due to less business	☐
03	No, reduction in hours/amount of work to care for ill relative	☐
04	No, reduction in hours/amount of work to take care of children as school is closed	☐
05	No, reduction in hours/amount of work to help children with homework because school is closed	☐
06	No, reduction in hours/amount of work as more domestic work because of the outbreak	☐
07	No, reduction in hours/amount of work as more time needed to buy food and cook	☐
08	ETHIOPIA ONLY: No, reduction in hours/days/amount of work due to employers/government action to reduce the spread of covid-19 between workers	☐
09	Others	☐

Section 9. Subjective wellbeing and Mental Health

9.1 Subjective wellbeing

SAY: Now, I'm going to ask you some questions about your wellbeing and I would like to make sure that you feel comfortable enough to answers those questions.

**Q.1**	Are you alone in the room? Or could you find a quiet space?<br><br>00=No -> **SAY**: Ok, don't worry. Just make sure that you are not on speakers, so the conversation is between the two of us only. Let me ask few questions and please remember that you can decide not to answer if you wish.<br><br>01=Yes -> **SAY**: Great. Just make sure that you are not on speakers, so the conversation is between the two of us only. Let me ask few questions then, and remember that you can decide not to answer if you wish	○ No ○ Yes

SAY: Now, I want you to think of a ladder with nine steps. Suppose the ninth step, **at the very top**, represents the best possible life for you, and first step**, at the bottom** represents the worst possible life for you. Having in mind that scale…

**Q.2**	Where on the ladder do you feel you personally stand at the present time?<br><br>Record step number: 01-09<br><br>77=NK<br>79=Refused to answer	[__ __]

9.2 Mental Health

GAD-7 (Anxiety)

SAY: I am going to read you some questions and I want you to tell me whether these situations have occurred to you or not in tl weeks. If this has happened to you, I will also ask you how often this happened

			**Q.4** How often the situation occurred in the last two weeks?
**Q.3-Q.4**	**FIELDWORKER:** read the table line by line.<br><br>**Q.3. SAY:** In the last two weeks, have you been…?<br><br>00=No, not at all<br>01=Yes, even if a little bit<br><br>**CAPI:** Enable Q.4, for those items where Q.3 =01. If the answer is 00=No, move to the next line<br><br>**ENUMERATOR:** please make sure that the [YL Child] understand that No means never not even for a moment or a day in the past two week.		01= Less than half the days<br>02=More than half the days<br>03=Nearly everyday<br>77=NK<br>79=RTA<br>88=NA
**01**	Feeling nervous, anxious or on edge	○ No, not at all<br>○ Yes, even if a little bit	[__]
**02**	Not being able to stop or control worrying	○ No, not at all<br>○ Yes, even if a little bit	[__]
**03**	Worrying too much about different things	○ No, not at all<br>○ Yes, even if a little bit	[__]
**04**	Trouble relaxing/ Can't relax	○ No, not at all<br>○ Yes, even if a little bit	[__]
**05**	Being so restless that it's hard to sit still	○ No, not at all<br>○ Yes, even if a little bit	[__]
**06**	Becoming easily annoyed or irritable	○ No, not at all<br>○ Yes, even if a little bit	[__]
**07**	Feeling afraid as if something awful might happen	○ No, not at all<br>○ Yes, even if a little bit	[__]

PHQ-8

Say: I am going to read you some questions and I want you to tell me whether these situations have ocurred to you or not. If thi happened to you, I also want to know how often have occurred in the last two weeks

			**Q.6** How often the situation occurred in the last two weeks?
**Q.5-Q.6**	**FIELDWORKER:** read the table line by line.<br><br>**Q.5. SAY:** In the last two weeks, have you been bothered by any of the following problems…?<br><br>00=No, not at all<br>01=Yes, even if a little bit<br><br>**CAPI:** Enable Q.6, for those items where Q.5 is 01=Yes. If the answer is 00=No, move to the next line<br><br>**ENUMERATOR**: please make sure that the [YL Child] understand that No means never not even for a moment or a day in the past two week.		01= Less than half the days<br>02=More than half the days<br>03=Nearly everyday<br>77=NK<br>79=RTA<br>88=NA
**01**	Little interest or pleasure in doing things	O No, not at all<br>O Yes, even if a little bit	[__]
**02**	Feeling down, depressed or hopeless	O No, not at all<br>O Yes, even if a little bit	[__]
**03**	Trouble falling or staying asleep, or sleeping too much	O No, not at all<br>O Yes, even if a little bit	[__]
**04**	Feeling tired or having little energy	O No, not at all<br>O Yes, even if a little bit	[__]
**05**	Poor appetite or overeating	O No, not at all<br>O Yes, even if a little bit	[__]
**06**	Feeling bad about yourself - or that you are a failure or have let yourself or your family down	O No, not at all<br>O Yes, even if a little bit	[__]
**07**	Trouble concentrating on things, such as reading the newspaper or watching television	O No, not at all<br>O Yes, even if a little bit	[__]
**08**	Moving or speaking so slowly that other people could have noticed. Or the opposite - being so fidgety or restless that you have been moving around a lot more than usual.	O No, not at all<br>O Yes, even if a little bit	[__]

SAY: Now, I would like to ask you

**Q.7**	[CAPI: PE only] Since the beginning of the pandemic, in general, how do you think your relationship **with your father and mother** has changed?01= It has deteriorated a lot02= It has deteriorated a little03= It remains the same04= It has improved05= It has improved a lot79 = RTA 88 = NA	[__ __]
**Q.8**	[CAPI: PE only]<br><br>Since the beginning of the pandemic, in general, how do you think your relationship **with the rest of household members** has changed?<br><br>01= It has deteriorated a lot<br>02= It has deteriorated a little<br>03= It remains the same<br>04= It has improved<br>05= It has improved a lot<br>79 = RTA<br>88 = NA	[__ __]

**Q.9**	What are you <u>most</u> looking forward to do after COVID-19 is over?<br><br>**FIELDWORKER**: Do not read the options. If the respondent mention more than one please ask about the most desired one.	
01	Going back to education	[__ __]
02	Going back to work/workplace	
03	Visit family	
04	Visit friends	
05	Go to the city center	
06	Go to the park	
07	Go out for dinner	
08	Play a team sport	
09	To go to church/mosque/temple/ceremonies/weddings	
10	None	
11	Other (specify)	[____________]

Concluding remarks

**Q.1**	Is there anything else you would like to tell us?	Specify [__________]

SAY: YL Child´s name It has been very good for me to talk to you, and as I have said, in these circumstances/in these moments your information is very interesting and important.

In [November/ December, DATE OF PHONE SURVEY THIRD CALL] I will call you again to find out how you and your family are and ask you few questions to understand better how do you feel, how is your family. The information that you and the other Young Lives participants will provide us with will be extremely valuable to understand how things are changing and what policies can be effective in improve the situation. The final survey takes approximately 15 minutes.

**Q.2**	Is there a day of the week that usually works better for you?<br>**ENUMERATOR**: Mark all that apply	
01	Monday	☐
02	Tuesday	☐
03	Wednesday	☐
04	Thursday	☐
05	Friday	☐
06	Saturday	☐
07	Sunday	☐
**Q.3**	Is it easier to reach you in the mornings or afternoons?<br>**ENUMERATOR**: Read options and mark all that apply	
	01=Mornings	☐
	02=Afternoons	☐
	03= Evening (after dinner)	☐

Other comments	Specify [______________________________]

**SAY**: You will remember that whenever we have visited you, at the end of the interview, in gratitude to the time that you have given us to answer all the questions, we have given you a [PE: a small present, e.g. backpack, watch, mini radio, etc.); ET: a small amount of money or transferred mobile card; IN: money purse, vanity bag, etc; VN: a small amount of money], right?<br><br>[PE and IN] As I mentioned at the beginning, this time we can't visit the participants in their homes, so, only for this time, each participant will be given [PE: S /. 50 soles, which is the approximate equivalent to the value (cost) of the present], [IN: Rs. 600, which is the approximate equivalent to the value (cost) of the present] , as we anticipated to you in the previous call. Would you prefer to receive the 50 soles now or in November, at the end of the survey?”	[_____________]
[ET and VN] As I mentioned at the beginning, this time we can't visit the participants in their homes, so, only for this time, each participant will be given [ET: Birr 100 or Mobile card which is the approximate equivalent to the value of the money] [VN: VND 50,000 or a mobile card which is he approximate equivalent to the value of the money][The delivery of the money would be made by means of a "Money Order" by : [PE: Banco de la Nación; ET: Commercial Bank of Ethiopia; VN: Commercial Bank of Viet Nam] or any other bank that the participant indicates, so that they can collect it from there. Or if you don't have access to a Bank [PE: you can receive the reward from any BCP or Banco de la Nacion Agent located near your living place; ET: we may send you mobile card equivalent to the money. Or if you prefer to receive the money, we can send you the amount through our local guide. Please let us know your preference; VN: we may send you mobile card equivalent to the money. Please let us know your preference.] [IN: The amount will be sent through any nationalised banks by RTGS or we don't want to give us your bank account or you don't have one, we will send the amount by "Money Order" and it will take 4 or 5 days to reach them through Post office. In both cases we will send a photocopy of the receipt].Now, before I say goodbye, I would like to verify your address and your contact numbers. -> **Go to next section (Locating Information)**	[_____________]

Locating Information and contact info
[No publicly archived]

SAY: That's all for now, again: Thank you very much for your help and remember that in [between November and December, DATE OF PHONE SURVEY THIRD CALL] I am calling again.

Thank you very much and stay safe!

Comments

CAPI: This section should be enabled for all respondents regardless of whether they decide to participate or not
FIELDWORKER: Please use this section to take notes whether the YL child asked for further information or if you noticed anything that gave you cause for concern in relation to this young person/family. On this second aspect, please make sure to refer those cases to the field coordinator as soon as possible.

CAPI: enable the following comment fields C.1 and C.2 for all respondents, including those not going ahead with the survey (farewell F1 and farewell F2)

ENUMERATOR please provide with the following information:

**C.1**	Has the [YL Child] asked for information?<br><br>[CAPI] : If No --> Skip to C.3	○ No(0) ○ Yes(1)
**C.2**	Related to what?<br>ENUMERATOR: Do not read. Mark all that apply	
01	related to COVID-19 (symptoms, treatments, how it is transmitted etc..)	☐
02	related to health insurance	☐
03	related to economic support offered by the government	☐
04	related to jobs	☐
05	related to education	☐
06	related to scholarship	☐
07	relate to present offered to YL survey participations	☐
08	related to violence	☐
09	related to mental health	☐
10	Others, Specify	☐
11	Specify	[______________]
Any other comments?		

**C.3**	Was there anything that gave you cause for concern in relation to this young person/family?	○ No(0) ○ Yes(1)
**C.4**	Related to what?<br>ENUMERATOR: Mark all that apply	
01	Poor health	☐
02	Struggle to get something to eat	☐
03	Anxious, distressed about not having a job	☐
04	Related to personal violence	☐

05	Related to family violence	☐
06	Related to mental health	☐
07	Others, Specify	☐
08	Specify	[______________]
Any other comments?		____

Enumerator Statement

PERU

I declare that I have complied with the consent process, informing the Young Lives participant everything related to the survey, reading the consent text, and answering all of his/her doubts, questions and/or queries. Also, I regulate my conduct in accordance with the requirements of the Young Lives Code of conduct.

If the above statement is true, please fill in:

Your full name in capital letters [**CAPI**: pre-fill using Data Handlers information]	[____]
Your 8-digit ID national number	[_ _ _ _ _ _ _ _]
The full name of the YL participant [**CAPI**: pre-fill using Data Handlers information]	[____]
The 8-digit ID national number of the YL participant	[_ _ _ _ _ _ _ _]

ETHIOPIA

I declare that I have complied with the consent process, informing the Young Lives participant everything related to the survey, reading the consent text, and answering all of his/her doubts, questions and/or queries.
Also, I regulate my conduct in accordance with the requirements of the Young Lives Code of conduct.

If the above statement is true, please fill in:

Your full name in capital letters [**CAPI**: pre-fill using Data Handlers information]	[____]
Your Kebele ID number [**CAPI**: pre-fill using Data Handlers information]	[____]
The full name of the YL participant	[____]

INDIA

I declare that I have complied with the consent process, informing the Young Lives participant everything related to the survey, reading the consent text, and answering all of his/her doubts, questions and/or queries. Also, I regulate my conduct in accordance with the requirements of the Young Lives Code of conduct.

If the above statement is true, please fill in:

Your full name in capital letters [**CAPI**: pre-fill using Data Handlers information]	[____]
The full name of the YL participant [**CAPI**: pre-fill using Data Handlers information]	[____]

VIETNAM

I declare that I have complied with the consent process, informing the Young Lives participant everything related to the survey, reading the consent text, and answering all of his/her doubts, questions and/or queries. Also, I regulate my conduct in accordance with the requirements of the Young Lives Code of conduct.

If the above statement is true, please fill in:

Your full name in capital letters [**CAPI**: pre-fill using Data Handlers information]	[____]
The full name of the YL participant [**CAPI**: pre-fill using Data Handlers information]	[____]

Codeboxes

CODEBOX #1
Source of drinking water

ET

01=Bore well in dwelling/yard/plot
02=Bore well in another location
03=Bought water (delivery or bottled)
04= Piped into dwelling/yard/plot
05=Piped into neighbours dwelling/yard/plot
06=Piped into relatives' dwelling/yard/plot
07=Piped (communal)
08=Protected spring or well in dwelling/yard/plot
09=Protected spring or well in another location
10=Public standpipe/tubewell
11=Rain water (during rainy season)
12=Tubewell in dwelling/yard/plot
13=Unprotected well/spring/pond/river/stream/ canal/lake
14=Water tank in dwelling/yard/plot
15=Water tank in another location
16=Other source in dwelling/yard/plot, specify ________
17=Other source in another location, specify ________

PE

01= Piped water to the house/plot (public network)
02 = Piped water to the house/plot (non-public network)
03= Well, tube well with hand pump in house/plot
04= Well, tube well with hand pump in another location
05= Common tap, water fountain, public well
06= Lake, river,/ ditch / stream/ natural spring/unprotected canal
07= Water truck delivery
08= Unprotected Well in another location
09= Unprotected Well in house/plot
10 = Piped water from neighbors/relatives
11 = Untreated water from neighbors/ relatives
12=Other source in house/plot, specify ________
13=Other source in another location, specify ________
77 = NK
88 = NA

IN, VN

01=Bore well in the dwelling/yard/plot
02=Bore well in another location
03=Bought water (delivery or bottled)
04= Piped into dwelling/yard/plot
05=Piped into neighbours dwelling/yard/plot
06=Piped into relatives' dwelling/yard/plot
07=Protected spring or well in dwelling/yard/plot
08=Protected spring or well in another location
09=Public standpipe/tubewell
10=Rain water
11=Tubewell in dwelling/yard/plot
12=Unprotected well/spring/pond/river/stream/ canal
13=Unprotected water tank in dwelling/yard/plot
14=Unprotected water tank in another location
15=Water tank (community/protected)
16=Other source in dwelling/yard/plot, specify ________
17=Other source in another location, specify ________

CODEBOX #2

Toilet facility

ET

01=Flush toilet/septic tank (private)
02=Flush toilet/septic tank (shared)
02=Forest/field/open place
05=Pit latrine (communal)
06=Pit latrine (household's)
07=Bucket
10=Others (household only) [Specify____________]
11=Others (communal) [Specify____________]

IN

01=Flush toilet/septic tank
02=Forest/field/open place
03=Neighbours toilet
05=Pit latrine (communal)
06=Pit latrine (household's)
07=Relatives toilet
09=Toilet in health post
10=Others (household only) [Specify____________]
11=Others (communal) [Specify____________]

PE

01= Flush toilet/septic tank
02=Forest/field/open place
03=Neighbours toilet

04= None (river, stream, irrigation ditch)
05= Pit latrine (communal)
06= Pit latrine (household's)
07=Relatives toilet
09=Toilet in health facility / market
10=Others (household only) [Specify___________]
11=Others (communal) [Specify___________]

VN

01= Flush toilet/septic tank
02=Forest/field/open place
03=Neighbours toilet
05= Pit latrine (communal)
06= Pit latrine (household's)
10=Others (household only) [Specify___________]
11=Others (communal) [Specify___________]

CODEBOX #3
Education

ET

00 = None
01-08 = Grade 1-8 (Primary)
09-10= Grade 9-10 (Secondary First Cycle)
11-12 = Grade 11-12 (Secondary Second Cycle Preparatory Programme)
13 = First cycle of primary teaching certificate (grade 1-4)/1st year
14 = First cycle of primary teaching certificate (grade 1-4)/2nd year
17 = Second cycle of primary teaching certificate (grades 5-8)/1st year
16 = Second cycle of primary teaching certificate (grades 5-8)/2nd year
21 = TVET/1st year/level (include diplomas such as accounting diploma)
22 = TVET/2nd year/level (include diplomas such as accounting diploma)
26 = TVET/3rd or 4th year/level (include diplomas such as accounting diploma)
27=Secondary education, teacher (diploma holder)/1st or 2nd year
30 = Secondary education, teacher (bachelor's degree holder and above)/1st or 2nd or 3rd year
31=Preschool teacher certificate (6 months to one year)
32 = Undergraduate degree (1st year, regular (R))
33 = Undergraduate degree (2nd year, regular (R))
34 = Undergraduate degree (3rd year, regular (R))
35 = Undergraduate degree (4th year, regular (R))
36 = Undergraduate degree (1st year or equivalent, non-regular (NR), summer/distant/ evening/weekend student)
37 = Undergraduate degree (2nd year or equivalent, non-regular (NR), summer/distant/ evening/weekend student)

38 = Undergraduate degree (3rd year or equivalent, non-regular (NR), summer/distant/ evening/weekend student)
39 = Undergraduate degree (4th year or equivalent, non-regular (NR), summer/distant/ evening/weekend student)
43= Undergraduate degree (5rd year, regular (R))
44 = Undergraduate degree (6th year, regular (R))
45= Undergraduate degree (6th year, regular (R))
46= Undergraduate degree (5th year or equivalent, non-regular (NR), summer/distant/ evening/weekend student)
47 = Undergraduate degree (6th year or equivalent, non-regular (NR), summer/distant/ evening/weekend student)
48 = Undergraduate degree (7th year or equivalent, non-regular (NR), summer/distant/ evening/weekend student)
40=Masters or doctoral at university
28=Adult literacy
29=Religious education
88= NA
77=NK
41 =Other, (specify)________

IN

YC

00 = None
01-12= Grade
13=Post-secondary technological institute
14=Vocational
15=University degree (graduate)
28=Adult literacy
29=Religious education
77=NK
79=Refused to answer

OC

00=No certificate (or Pre-school)
01=Primary (Class 5)
02=Upper primary (Class 8)
03=Matriculation certificate (Class 10)
04=Senior Secondary school certificate / Intermediate certificate (Class 12)
05=ITI certificate
06=Diploma in technical education
08=Other, specify
09=University degree (graduate)

10=Post-graduate university degree (completed)
77=NK
79=Refused to answer

PE

00 = None
01-11 = Grade
13 = Incomplete technical or pedagogical institute (incl. "Escuela de Sub Officiales")
14 = Complete technical or pedagogical institute (incl. "Escuela de Sub Oficiales")
15 = Incomplete university (incl. "Escuela de Oficiales")
16 = Complete university (incl. "Escuela de Oficiales")
17 = Adult literacy program
19 = Masters or doctoral at university
18 = Other (Specify)
20 = Some form of formal or informal preschool
21 = Cent. Técnico Productivo CETPRO/Cent. Edu. Ocupacional CEO Incomplete
22 = Cent. Técnico Productivo CETPRO/ Cent. Edu. Ocupacional CEO Complete
77 = NK
88 = NA

VN

00 = None
01-12= Grade
13=Post-secondary technological institute
14=Vocational
15=Degree (graduate)
16=Post-graduate degree (e.g. Masters, PhD.)
17=Other, specify
28=Adult literacy
29=Religious education
50=Any pre-primary
77=NK
79=Refused to answer

CODEBOX #4
Type of educational institution

ET

01 = Private/domestic
02= Public (part student fees, part government funded);
03= Mission (NGO/Charity/Religious) with fees
04= Mission (NGO/Charity/Religious) free of charge
05= Government funded
06=Others, specify
07=Community school
08=Private-foreign like UNISA, SRI-SAI
09=domestic-foreign joint programme
77=NK
88=NA

IN

01=Private (unaided)
02=NGO/Charity/not-for-profit
03=Public (Government)
04=Informal or non-formal Community (e.g. mothers' cooperative)
06=Charitable trust
07=Bridge school
08=Mix of public and private (private aided)
11=Other, specify
77=NK
79=Refused to answer

PE

01 = Private, for profit
02 = NGO / Charity / Church (not for profit),
03 = Public, local municipality
04 = Public, of the national government,
06 = Informal,
07 = Half public / Half Private
05 = Other (specify)
08 = Center of public alternative basic education
09 = Center of private alternative basic education

10 = Public, Colegio de Alto Rendimiento (COAR)
11 = Public, Jornada Escolar Completa (JEC)
77 = NK,
79=RTA,
88 = NA

VN

01 =Private
02=NGO/Charity/ Religious (not for profit)
03=Public
06=Informal
07=Half public/Half Private
08=Centre for continuing education
**05=Others, specify**
**77=NK**
**88=NA**

Codebox #5:
Major classification

ET

	**Engineering and Technology**
1	Chemical Engineering
2	Civil Engineering / construction
3	Electrical Engineering
4	Mechanical Engineering
5	Information Technology/ Computer science
	**Natural and Computational Sciences**
10	Biology, Physics, or Chemistry
11	Mathematics or statistics
12	Geology or earth science
	**Medicine and Health Sciences**
20	Medicine
21	Health officer
22	Laboratory/radiology/ Midwifery/Nursing
23	Pharmacy
24	Animal Science/veterinary
	**Agriculture and Life Sciences**
30	Animal Production
31	Plant science/Crop Production
32	Natural resource management
33	development agent
	**Business and Economics**
40	Accounting / Management
41	Economics
42	Public Administration
	**Education and journalism**
50	Local languages and literature
51	Foreign language and literature
52	Education Development and Planning Management
53	Curriculum
54	Journalism & Communication
	**Social Science and Humanities**
60	Geography or history
61	Psychology
62	Law / political science
63	Humanities
64	Social work and/ Social Anthropology
70	Others (please mention)

IN

CODES
1=Teacher training
2=Fine Arts
3=Humanities and Social Sciences
4=Sciences
5=Journalism and Communication
6=Business and management
7=Law and Legal Services
8=Mathematics and Statistics
9=Computer and Information Technology
10=Food processing
11=Other technologies (Bio-technology etc)
12=Engineering
13=Agriculture, Forestry and Aquaculture
14=Veterinary
15=Medical, Pharmacy and Health Sciences
16=Tourism, Hotels, Recreation and Sports
17=Armed force and Police
18=Others Specify

PE

CODES
11=Preschool and Primary school
12=Secondary school
13=Physical Education
14=Special Education
15=Artistic Education
16=Technical Education
19=Other educational majors
21=Humanities
22=Arts
31=Social Sciences and Sciences of behaviour
32=Communication Sciences
33=Administration and Commercial Sciences
34=Economic and Accounting Sciences
35=Law, Political and Legal Sciences
41=Life Sciences
42=Physical and Chemical Sciences
43=Mathematics and Statistics
44=Computing
51=Systems and Telecommunications Engineering
52=Industrial Engineering and Production
53=Construction, Sanitary and Architecture Engineering

59=Other Engineering
61=Agriculture, Forestry and Aquaculture
62=Veterinary
71=Health Sciences
01=Armed Forces
02=Police Forces

VN

CODES
1=Education and teacher training
2=Art
3=Humanities
4=Social Sciences and Sciences of behaviour
5=Journalism and information
6=Business and management
7=Law and Legal Services
8=Life Sciences
9=Natural sciences
10=Mathematics and Statistics
11=Computer and Communications
12=Food processing
13=Other technologies
14=Construction and Architecture Engineering
15=Agriculture, Forestry and Aquaculture
16=Veterinary
17=Health Sciences
18=Social services
19=Tourism, Hotels, Recreation and Sports
20=Transports
21=Environmental Protection
22=Armed force and Police

Codebox #6:
Work activities

AGRICULTURE & ALLIED AGRICULTURE
01=Self Employed<br>(Food crops)
02=Self Employed<br>(Non-food, including horticulture, sericulture and floriculture)
03 = Self Employed (Aquaculture)
04 = Self Employed<br>(Livestock)

05=Wage Employment (Agriculture)
06=Annual Farm Servant
07=Other (allied) agriculture, specify
**NON-AGRICULTURE**
08=Self Employed (Manufacturing)
09 = Self Employed (Services)
10= Self Employed (Business)
11 = Self Employed (Other non-Agriculture)
12=Wage Employment (Unsalaried/ irregular; Non-agriculture)
13=Regular Salaried Employment
15 = Begging
19=Other non-agriculture, specify

Codebox #7:
Economic sector

01=Agriculture, forestry and fishing
02=Mining and quarrying
03=Manufacturing
04=Electricity, gas, steam and air conditioning supply
05=Water supply; sewerage, waste management and remediation activities
06=Construction
07=Wholesale and retail trade; repair of motor vehicles and motorcycles
08=Transportation and storage
09=Accommodation and food service activities
10=Information and communication
11=Financial and insurance activities
12=Real estate activities
13=Professional, scientific and technical activities
14=Administrative and support service activities
15=Public administration and defence; compulsory social security
16=Education
17=Human health and social work activities
18=Arts, entertainment and recreation
19=Other service activities
20=Activities of households as employers; undifferentiated goods- and services-producing activities of households for own use
21=Activities of extraterritorial organizations and bodies
77=NK
88=NA
79=RTA
