# Supplementary material for: Impact of the COVID-19 pandemic on anxiety and depression symptoms of young people in the global south: evidence from a four-country cohort study
Source: BMJ Open. 2021 Apr 15;11(4):e049653. doi: 10.1136/bmjopen-2021-049653 (PMC8053815; doi:10.1136/bmjopen-2021-049653)
Supplement: Supplementary data [file bmjopen-2021-049653supp002.pdf]

Supplementary tables

Supplement to: Impact of the COVID-19 Pandemic on anxiety and depression symptoms of young people in the Global South: evidence from a four-country cohort study

Supplementary Table 1. Sample size 3
Supplementary Table 2. Attrition rates (%) 3
Supplementary Table 3. Non-response rate to mental health questions (%) 4
Supplementary Table 4. Measures of reliability and internal consistency 4
Supplementary Table 5. Sample description 5
Supplementary Table 6. Logistic regression results: Symptoms of at least mild anxiety (Younger Cohort only) 7
Supplementary Table 7. Logistic regression results: Symptoms of at least mild depression (Younger Cohort only) 8
Supplementary Table 8. Logistic regression results: Symptoms of at least mild anxiety (Older Cohort only) 10
Supplementary Table 9. Logistic regression results: Symptoms of at least mild depression (Older Cohort only) 11
Supplementary Table 10. Ethiopia: Means of continuous control variables by at least mild anxiety/depression 14
Supplementary Table 11. India: Means of continuous control variables by at least mild anxiety/depression 15
Supplementary Table 12. Peru: Means of continuous control variables by at least mild anxiety/depression 16
Supplementary Table 13. Vietnam: Means of continuous control variables by at least mild anxiety/depression 17
Supplementary Table 14. Mental health outcomes by control variables (Ethiopia & India) 18
Supplementary Table 15. Mental health outcomes by control variables (Peru & Vietnam) 19
Supplementary Table 16. Logistic regression results: Symptoms of at least mild anxiety/depression by gender (Ethiopia) 21
Supplementary Table 17. Logistic regression results: Symptoms of at least mild anxiety/depression by gender (India) 22
Supplementary Table 18. Logistic regression results: Symptoms of at least mild anxiety/depression by gender (Peru) 24
Supplementary Table 19. Logistic regression results: Symptoms of at least mild anxiety/depression by gender (Vietnam) 25
Supplementary Table 20. Logistic regression results: Symptoms of at least mild anxiety/depression by gender for the Younger Cohort only (Ethiopia) 28
Supplementary Table 21. Logistic regression results: Symptoms of at least mild anxiety/depression by gender for the Younger Cohort only (India) 29
Supplementary Table 22. Logistic regression results: Symptoms of at least mild anxiety/depression by gender for the Younger Cohort only (Peru) 31

Supplementary Table 23. Logistic regression results: Symptoms of at least mild anxiety/depression by gender for the Younger Cohort only (Vietnam) 32
Supplementary Table 24. Ethiopia: Mean score and rates of general anxiety disorder 34
Supplementary Table 25. Ethiopia: Mean score and rates of depression................. 35
Supplementary Table 26.India: Mean score and rates of general anxiety disorder .. 36
Supplementary Table 27. India: Mean score and rates of depression...................... 37
Supplementary Table 28. Peru: Mean score and rates of general anxiety disorder . 38
Supplementary Table 29. Peru: Mean score and rates of depression 39
Supplementary Table 30. Vietnam: Mean score and rates of general anxiety disorder 40
Supplementary Table 31. Vietnam: Mean score and rates of depression................. 41
Supplementary Table 32. Correlation between GAD-7 and PHQ-8 scores 42
Supplementary Table 33. Joint rates 42
Supplementary Table 34. Correlation between subjective well-being and GAD-7/PHQ-8 scores 43
Supplementary Table 35. Subjective well-being score Round 5 (2016) / phone survey (2020) by anxiety/depression (Ethiopia & India) 44
Supplementary Table 36. Subjective well-being score Round 5 (2016) / phone survey (2020) by anxiety/depression (Peru & Vietnam) 45

Supplementary Table 1. Sample size

	Ethiopia			India			Peru			Vietnam			Total		
	Full sample	YC	OC	Full sample	YC	OC	Full sample	YC	OC	Full sample	YC	OC	Full sample	YC	OC
Round 5, 2016	2626	1812	814	2822	1900	922	2468	1860	608	2848	1938	910	10764	7510	3254
Tracking samples, 2019-2020	2634	1803	831	2815	1902	913	2215	1701	514	2832	1922	910	10496	7328	3168
Phone survey, 2020	2439	1665	774	2754	1868	886	2018	1561	457	2519	1691	828	9730	6785	2945
Mental health measures available	2433	1659	774	2753	1867	886	1977	1535	442	2517	1690	827	9680	6751	2929
Included in our analysis	2183	1533	650	2622	1786	836	1887	1496	391	2296	1596	700	8988	6411	2577

Notes: YC stands for Younger Cohort, OC for Older Cohort.

Supplementary Table 2. Attrition rates (%)

	Ethiopia			India			Peru			Vietnam			Total		
	Full sample	YC	OC	Full sample	YC	OC	Full sample	YC	OC	Full sample	YC	OC	Full sample	YC	OC
Tracking sample to phone survey	7.4	7.7	6.9	2.2	1.8	3.0	8.9	8.2	11.1	11.1	12.0	9.0	7.3	7.4	7.0

Supplementary Table 3. Non-response rate to mental health questions (%)

Ethiopia			India			Peru			Vietnam			Total		
Full sample	YC	OC	Full sample	YC	OC	Full sample	YC	OC	Full sample	YC	OC	Full sample	YC	OC
0.2	0.4	0.0	0.0	0.1	0.0	2.0	1.7	3.3	0.1	0.1	0.1	0.5	0.5	0.5

Supplementary Table 4. Measures of reliability and internal consistency

		Cronbach's Alpha	Inter-item correlation	Kaiser–Meyer–Olkin measure of sampling adequacy
**Ethiopia**	GAD-7	0.728	0.277	0.792
	PHQ-8	0.652	0.190	0.744
**India**	GAD-7	0.691	0.242	0.777
	PHQ-8	0.637	0.180	0.744
**Peru**	GAD-7	0.804	0.369	0.872
	PHQ-8	0.773	0.299	0.839
**Vietnam**	GAD-7	0.749	0.299	0.814
	PHQ-8	0.748	0.271	0.807

Note: Results are for the combined Younger Cohort/ Older Cohort sample.

Supplementary Table 5. Sample description

	Ethiopia		India		Peru		Vietnam	
	Mean	sd	Mean	sd	Mean	sd	Mean	sd
COVID-19 related stressors								
Risk perception: believe they are at medium/high risk	69.31	0.461	46.38	0.499	54.27	0.498	21.39	0.410
Left house for any reason in the past 7 days	94.50	0.228	79.25	0.406	80.45	0.397	87.67	0.329
Difference in subjective well-being 2016-2020	0.96	2.068	0.49	1.949	0.52	2.066	-0.39	1.876
Change in responsibilities:								
Spend more time taking care of children	29.00	0.454	18.31	0.387	34.23	0.475	22.91	0.420
Spend more time on household chores	46.82	0.499	50.95	0.500	77.53	0.417	51.44	0.500
Spend more time working in the family business	12.96	0.336	4.12	0.199	15.00	0.357	10.89	0.312
Economic adversities								
Faced with new health expenses	10.12	0.302	55.95	0.497	29.57	0.456	6.53	
Did not experience adversity	20.71	0.405	1.72	0.130	2.60	0.159	42.25	0.494
Experienced adversity but did not reduce food consumption	65.83	0.474	92.68	0.261	90.94	0.287	41.94	0.494
Reduced food consumption as response to experienced adversity	13.47	0.341	5.61	0.230	6.47	0.246	15.81	0.365
Changes in employment status								
Did not work at all in the past 12 month OR worked during the pandemic but not before the pandemic (and is not working now)	46.17	0.499	41.38	0.493	24.06	0.428	23.48	0.424
Did not work before the pandemic, but is working now	6.83	0.252	16.40	0.370	12.35	0.329	6.75	0.251
Worked before the pandemic and is working now/has a job	38.71	0.487	37.57	0.484	46.74	0.499	56.36	0.496
Worked before the pandemic and is not working now/does not have a job	8.29	0.276	4.65	0.211	16.85	0.374	13.41	0.341
Educational disruption								
Never attended school or not enrolled in full-time education/not planning to enrol[a]	32.68	0.469	33.87	0.473	58.96	0.492	47.49	0.500
Enrolled in/Planning to enrol in full time education and not participating in learning activities[a]	58.71	0.493	36.28	0.481	1.87	0.136	12.72	0.333
Enrolled in/Planning to enrol in full time education and participating in learning activities[a]	8.61	0.281	29.84	0.458	39.17	0.488	39.79	0.490
Background characteristics								
Female	46.27	0.499	47.79	0.500	49.28	0.500	51.61	0.500
Urban	45.99	0.499	28.60	0.452	81.13	0.391	45.73	0.498
Child has long-term health problem, 2016 (Round 5)	8.80	0.283	11.48	0.319	12.67	0.333	14.20	0.349
Total parent-child relationship score, 2012/2016 (Round 4/5)	26.26	2.848	27.63	2.916	24.86	3.610	25.74	3.204

	Ethiopia		India		Peru		Vietnam	
	Mean	sd	Mean	sd	Mean	sd	Mean	sd
Total peer-child relationship score, 2016 (Round 5)	24.13	2.866	25.02	2.631	23.41	3.204	22.57	
Middle/Top wealth tercile R5, 2016 (Round 5)	67.11	0.470	66.44	0.472	69.00	0.463	66.03	2.257
Proxy baseline information								
Emotional problem scale (EPS) score, 2009 (Round 3)[b]	2.78	2.473	3.57	2.348	4.27	2.323	3.68	2.143
Subjective well-being, 2009 (Round 3)[b]	4.86	1.719	4.73	1.815	6.12	1.616	5.43	1.604
N	2183		2622		1887		2296	

Note: Mean values are reported. Self-reported risk belief coded the question "chance of getting infected with Coronavirus" into no/low risk, or medium/high risk. All time-variant variables are measured in 2020 unless otherwise specified. [a] refers to Younger Cohort (18-19) only, [b] refers to Older Cohort (25-26) only. Around 30% of the sample in Ethiopia, India and Vietnam are part of the Older Cohort. In Peru they make up 20% of the sample. The average age of the Younger Cohort in Ethiopia, India, Peru, Vietnam in years (months) is 18 (226), 18 (227), 18 (227), and 18 (227). The average age of the Older Cohort in Ethiopia, India, Peru, Vietnam in years (months) is 25 (309), 25 (310), 25 (311), and 25 (311).

Supplementary Table 6. Logistic regression results: Symptoms of at least mild anxiety (Younger Cohort only)

	Ethiopia		India		Peru		Vietnam	
	**Odds Ratio**	**95% CI**	**Odds Ratio**	**95% CI**	**Odds Ratio**	**95% CI**	**Odds Ratio**	**95% CI**
COVID-19 related changes/behaviours								
Risk perception: believe they are at medium/high risk	0.679** (0.12)	[0.487,0.947]	1.244 (0.21)	[0.898,1.724]	1.432*** (0.16)	[1.149,1.784]	1.031 (0.22)	[0.673,1.579]
Left house for any reason in the past 7 days	0.885 (0.27)	[0.482,1.627]	1.576* (0.41)	[0.948,2.620]	1.023 (0.14)	[0.784,1.335]	1.304 (0.34)	[0.783,2.171]
Difference in subjective well-being between Round 5 (2016) and phone survey (2020)	1.043 (0.04)	[0.969,1.123]	1.103** (0.05)	[1.007,1.207]	1.102*** (0.03)	[1.046,1.161]	1.195*** (0.06)	[1.084,1.316]
Change in responsibilities:								
Spend more time taking care of children	1.012 (0.20)	[0.692,1.479]	2.176*** (0.52)	[1.363,3.475]	1.509*** (0.18)	[1.194,1.906]	1.400 (0.31)	[0.913,2.147]
Spend more time on household chores	0.983 (0.17)	[0.694,1.392]	0.816 (0.15)	[0.570,1.169]	1.180 (0.17)	[0.895,1.557]	1.013 (0.18)	[0.718,1.430]
Spend more time working in the family business	0.703 (0.21)	[0.394,1.253]	2.117** (0.67)	[1.136,3.943]	1.235 (0.19)	[0.917,1.663]	1.640** (0.38)	[1.037,2.593]
Economic shocks								
Faced with new health expenses	0.668 (0.21)	[0.360,1.240]	0.881 (0.16)	[0.624,1.245]	1.741*** (0.21)	[1.371,2.209]	1.309 (0.42)	[0.699,2.455]
Experienced adversity but did not reduce food consumption	3.636*** (0.99)	[2.128,6.213]	0.702 (0.38)	[0.245,2.011]	2.503** (1.08)	[1.074,5.834]	1.493** (0.29)	[1.021,2.184]
Reduced food consumption as response to experienced adversity	8.158*** (2.58)	[4.393,15.149]	1.173 (0.73)	[0.345,3.985]	2.154 (1.03)	[0.844,5.498]	1.680** (0.42)	[1.027,2.748]
Educational disruption								
Enrolled in/Planning to enrol in full time education and not participating in learning activities	1.588** (0.33)	[1.052,2.396]	1.043 (0.21)	[0.707,1.538]	0.600 (0.27)	[0.245,1.471]	0.828 (0.25)	[0.458,1.495]
Enrolled in/Planning to enrol in full time education and participating in learning activities	1.110 (0.38)	[0.565,2.182]	0.710 (0.17)	[0.450,1.120]	1.026 (0.12)	[0.816,1.290]	0.699* (0.15)	[0.465,1.051]
Background characteristics								
Age in months	1.000 (0.02)	[0.960,1.042]	0.987 (0.02)	[0.946,1.030]	0.991 (0.01)	[0.963,1.020]	1.020 (0.03)	[0.967,1.077]
Female	1.216 (0.21)	[0.874,1.692]	1.420* (0.30)	[0.940,2.143]	1.758*** (0.20)	[1.402,2.205]	1.453** (0.25)	[1.034,2.041]
Urban	1.565** (0.31)	[1.059,2.312]	1.198 (0.24)	[0.806,1.781]	1.362* (0.22)	[0.986,1.882]	1.322 (0.26)	[0.901,1.940]
Participant has long-term health problem, 2016 (Round 5)	1.298 (0.36)	[0.755,2.233]	1.226 (0.30)	[0.754,1.995]	1.624*** (0.28)	[1.163,2.266]	1.030 (0.29)	[0.591,1.793]
Total parent-child relationship score, 2012/2016 (Round 4/5)	0.959 (0.03)	[0.902,1.019]	0.912*** (0.03)	[0.861,0.967]	0.975 (0.02)	[0.944,1.007]	1.030 (0.03)	[0.976,1.088]

	Ethiopia		India		Peru		Vietnam	
	Odds Ratio	95% CI	Odds Ratio	95% CI	Odds Ratio	95% CI	Odds Ratio	95% CI
Total peer-child relationship score, 2016 (Round 5)	0.951 (0.03)	[0.895,1.011]	1.015 (0.04)	[0.948,1.088]	0.999 (0.02)	[0.964,1.035]	0.968 (0.04)	[0.896,1.047]
Middle/Top wealth tercile R5, 2016 (Round 5)	0.908 (0.19)	[0.608,1.356]	0.712* (0.13)	[0.495,1.023]	1.105 (0.15)	[0.845,1.444]	0.677** (0.13)	[0.467,0.981]
N	1533		1786		1496		1596	

*Note: Odds ratios are unadjusted odds ratios. Robust standard errors in parenthesis, *** significant at 1%, ** significant at 5%, * significant at 10%. Base categories are as follows: Believe they are at no/low risk, Did not leave the house at all during the past 7 days, Did not spend more time taking care of children, Did not spend more time on household chores, Did not spend more time working in the family business, Did not face new health expenses, Did not suffer a shock, Never attended school or not enrolled in full-time education/not planning to enrol, Male, Rural, Does not have long-term health condition, Lowest wealth tercile. All time-variant variables are measured in 2020 unless otherwise specified.*

Supplementary Table 7. Logistic regression results: Symptoms of at least mild depression (Younger Cohort only)

	Ethiopia		India		Peru		Vietnam	
	Odds Ratio	95% CI	Odds Ratio	95% CI	Odds Ratio	95% CI	Odds Ratio	95% CI
COVID-19 related changes/behaviours								
Risk perception: believe they are at medium/high risk	0.498*** (0.08)	[0.361,0.687]	0.966 (0.16)	[0.693,1.346]	1.548*** (0.18)	[1.230,1.949]	1.025 (0.22)	[0.674,1.559]
Left house for any reason in the past 7 days	0.940 (0.28)	[0.520,1.702]	2.537*** (0.77)	[1.403,4.589]	0.928 (0.13)	[0.704,1.223]	1.163 (0.28)	[0.724,1.867]
Difference in subjective well-being between Round 5 (2016) and phone survey (2020)	1.049 (0.04)	[0.973,1.130]	1.102** (0.05)	[1.011,1.201]	1.079*** (0.03)	[1.020,1.142]	1.169*** (0.06)	[1.065,1.284]
Change in responsibilities:								
Spend more time taking care of children	1.061 (0.20)	[0.736,1.527]	2.170*** (0.51)	[1.370,3.438]	1.268* (0.16)	[0.992,1.621]	1.616** (0.33)	[1.087,2.403]
Spend more time on household chores	1.082 (0.19)	[0.763,1.533]	0.857 (0.16)	[0.592,1.241]	1.142 (0.17)	[0.858,1.520]	1.840*** (0.34)	[1.287,2.631]
Spend more time working in the family business	1.076 (0.29)	[0.636,1.823]	0.803 (0.34)	[0.354,1.823]	1.160 (0.18)	[0.850,1.582]	1.724** (0.38)	[1.121,2.652]
Economic shocks								
Faced with new health expenses	0.222*** (0.10)	[0.093,0.530]	1.062 (0.19)	[0.747,1.510]	1.851*** (0.23)	[1.450,2.363]	0.805 (0.28)	[0.410,1.580]
Experienced adversity but did not reduce food consumption	4.913*** (1.49)	[2.712,8.902]	0.651 (0.36)	[0.218,1.944]	2.541* (1.21)	[0.997,6.476]	1.763*** (0.36)	[1.181,2.631]
Reduced food consumption as response to experienced	12.771*** (4.42)	[6.484,25.156]	0.913 (0.61)	[0.248,3.357]	3.620** (1.89)	[1.300,10.079]	2.292*** (0.57)	[1.410,3.726]

	Ethiopia		India		Peru		Vietnam	
	**Odds Ratio**	**95% CI**	**Odds Ratio**	**95% CI**	**Odds Ratio**	**95% CI**	**Odds Ratio**	**95% CI**
adversity								
Educational disruption								
Enrolled in/Planning to enrol in full time education and not participating in learning activities	1.728*** (0.36)	[1.153,2.592]	1.103 (0.22)	[0.743,1.638]	0.743 (0.36)	[0.287,1.928]	0.944 (0.28)	[0.529,1.685]
Enrolled in/Planning to enrol in full time education and participating in learning activities	1.174 (0.41)	[0.591,2.333]	0.749 (0.18)	[0.469,1.196]	1.042 (0.13)	[0.816,1.329]	1.172 (0.25)	[0.768,1.788]
Background characteristics								
Age in months	0.999 (0.02)	[0.959,1.041]	0.997 (0.02)	[0.954,1.041]	0.974* (0.01)	[0.945,1.003]	1.028 (0.03)	[0.975,1.084]
Female	1.318 (0.23)	[0.940,1.848]	1.225 (0.25)	[0.821,1.828]	1.282** (0.16)	[1.009,1.630]	1.438** (0.24)	[1.032,2.004]
Urban	1.533** (0.29)	[1.051,2.235]	0.969 (0.20)	[0.644,1.457]	1.175 (0.20)	[0.840,1.643]	1.093 (0.22)	[0.736,1.623]
Participant has long-term health problem, 2016 (Round 5)	0.540* (0.20)	[0.265,1.098]	1.461 (0.35)	[0.909,2.346]	1.502** (0.26)	[1.077,2.095]	1.466 (0.37)	[0.889,2.415]
Total parent-child relationship score, 2012/2016 (Round 4/5)	1.011 (0.03)	[0.954,1.072]	0.890*** (0.03)	[0.838,0.945]	0.924*** (0.02)	[0.892,0.957]	0.971 (0.03)	[0.918,1.026]
Total peer-child relationship score, 2016 (Round 5)	0.902*** (0.03)	[0.848,0.960]	1.000 (0.04)	[0.930,1.076]	1.025 (0.02)	[0.987,1.065]	0.987 (0.04)	[0.910,1.071]
Middle/Top wealth tercile R5, 2016 (Round 5)	0.763 (0.15)	[0.518,1.122]	1.275 (0.24)	[0.879,1.851]	1.166 (0.17)	[0.878,1.550]	1.189 (0.24)	[0.797,1.773]
N	1533		1786		1496		1596	

*Note: Odds ratios are unadjusted odds ratios. Robust standard errors in parenthesis, *** significant at 1%, ** significant at 5%, * significant at 10%. Base categories are as follows: Believe they are at no/low risk, Did not leave the house at all during the past 7 days, Did not spend more time taking care of children, Did not spend more time on household chores, Did not spend more time working in the family business, Did not face new health expenses, Did not suffer a shock, Never attended school or not enrolled in full-time education/not planning to enrol, Male, Rural, Does not have long-term health condition, Lowest wealth tercile. All time-variant variables are measured in 2020 unless otherwise specified.*

Supplementary Table 8. Logistic regression results: Symptoms of at least mild anxiety (Older Cohort only)

	Ethiopia		India		Peru		Vietnam	
	Odds Ratio	95% CI	Odds Ratio	95% CI	Odds Ratio	95% CI	Odds Ratio	95% CI
COVID-19 related changes/behaviours								
Risk perception: believe they are at medium/high risk	1.466 (0.35)	[0.915,2.346]	1.383 (0.30)	[0.908,2.108]	1.693** (0.41)	[1.051,2.728]	1.060 (0.37)	[0.536,2.098]
Left house for any reason in the past 7 days	0.693 (0.33)	[0.274,1.751]	1.257 (0.38)	[0.697,2.267]	0.850 (0.31)	[0.414,1.746]	0.432* 0.21)	[0.169,1.109]
Difference in subjective well-being between Round 5 (2016) and phone survey (2020)	1.089* (0.05)	[0.987,1.201]	1.121* (0.07)	[0.983,1.278]	1.132* (0.08)	[0.990,1.294]	1.105 (0.12)	[0.901,1.356]
Change in responsibilities:								
Spend more time taking care of children	0.735 (0.21)	[0.420,1.285]	2.081***(0.52)	[1.271,3.405]	0.971 (0.25)	[0.590,1.599]	1.230 (0.46)	[0.595,2.544]
Spend more time on household chores	1.313 (0.38)	[0.742,2.325]	0.639* (0.16)	[0.394,1.034]	1.667 (0.53)	[0.893,3.114]	2.240**(0.87)	[1.043,4.808]
Spend more time working in the family business	1.275 (0.41)	[0.682,2.383]	0.498 (0.41)	[0.100,2.478]	1.599 (0.54)	[0.829,3.085]	2.126* (0.96)	[0.879,5.139]
Economic shocks								
Faced with new health expenses	1.523 (0.45)	[0.854,2.716]	1.435 (0.34)	[0.901,2.286]	1.572* (0.38)	[0.984,2.513]	0.808 (0.41)	[0.302,2.164]
Experienced adversity but did not reduce food consumption	0.908 (0.31)	[0.466,1.767]	0.560 (0.49)	[0.102,3.068]	1.312 (1.77)	[0.093,18.595]	1.988* (0.78)	[0.922,4.284]
Reduced food consumption as response to experienced adversity	4.323***(1.81)	[1.900,9.835]	0.567 (0.55)	[0.086,3.751]	2.691 (3.82)	[0.167,43.423]	1.986 (0.96)	[0.772,5.113]
Changes in employment status								
Did not work before the pandemic, but is working now	2.044 (0.97)	[0.809,5.165]	0.951 (0.47)	[0.358,2.525]	1.095 (0.62)	[0.363,3.303]	0.868 (0.78)	[0.149,5.071]
Worked before the pandemic and is working now/has a job	1.566* (0.39)	[0.956,2.567]	1.306 (0.36)	[0.756,2.255]	1.401 (0.56)	[0.638,3.075]	0.591 (0.34)	[0.194,1.796]
Worked before the pandemic and is not working now/does not have a job	2.787***(0.85)	[1.534,5.063]	2.312** (0.95)	[1.037,5.156]	5.434***(2.55)	[2.167,13.626]	1.693 (1.03)	[0.513,5.582]
Background characteristics								
Age in months	1.008 (0.03)	[0.956,1.063]	1.052* (0.03)	[0.999,1.108]	0.977 (0.03)	[0.923,1.034]	0.960 (0.04)	[0.879,1.047]
Female	0.754 (0.21)	[0.432,1.319]	1.954** (0.58)	[1.087,3.511]	1.125 (0.30)	[0.670,1.888]	0.790 (0.27)	[0.404,1.545]

		Ethiopia		India		Peru		Vietnam	
		Odds Ratio	95% CI	Odds Ratio	95% CI	Odds Ratio	95% CI	Odds Ratio	95% CI
	Urban	1.087 (0.23)	[0.713,1.658]	0.561** (0.15)	[0.334,0.942]	1.825 (0.75)	[0.819,4.065]	0.961 (0.31)	[0.516,1.790]
	Participant has long-term health problem, 2016 (Round 5)	1.134 (0.33)	[0.637,2.019]	1.583* (0.40)	[0.960,2.612]	2.591***(0.91)	[1.298,5.171]	1.647 (0.54)	[0.862,3.150]
	Total parent-child relationship score, 2012/2016 (Round 4/5)	0.957 (0.03)	[0.899,1.018]	0.939* (0.03)	[0.875,1.007]	0.997 (0.03)	[0.940,1.058]	1.118* (0.07)	[0.989,1.265]
	Total peer-child relationship score, 2016 (Round 5)	0.948 (0.03)	[0.887,1.014]	1.041 (0.04)	[0.963,1.124]	1.011 (0.04)	[0.938,1.089]	0.858* (0.08)	[0.720,1.023]
	Middle/Top wealth tercile R5, 2016 (Round 5)	1.033 (0.23)	[0.670,1.594]	0.871 (0.20)	[0.551,1.376]	0.635 (0.20)	[0.342,1.180]	0.488** 0.16)	[0.260,0.914]
Proxy baseline information									
	Emotional problem scale (EPS) score, 2009 (Round 3)	1.074* (0.04)	[0.994,1.161]	1.007 (0.05)	[0.910,1.114]	1.222*** 0.07)	[1.093,1.366]	1.072 (0.07)	[0.940,1.221]
	Subjective well-being, 2009 (Round 3)	0.979 (0.06)	[0.872,1.100]	1.131** (0.07)	[1.010,1.267]	0.924 (0.07)	[0.800,1.068]	0.975 (0.08)	[0.827,1.148]
	N	650		836		391		700	

*Note: Odds ratios are unadjusted odds ratios. Robust standard errors in parenthesis, *** significant at 1%, ** significant at 5%, * significant at 10%. Base categories are as follows: Believe they are at no/low risk, Did not leave the house at all during the past 7 days, Did not spend more time taking care of children, Did not spend more time on household chores, Did not spend more time working in the family business, Did not face new health expenses, Did not suffer a shock, Did not work at all in the past 12 month OR worked during the pandemic but not before and is not working now, Male, Rural, Does not have long-term health condition, Lowest wealth tercile. All time-variant variables are measured in 2020 unless otherwise specified.*

Supplementary Table 9. Logistic regression results: Symptoms of at least mild depression (Older Cohort only)

Depression	OC	Ethiopia		India		Peru		Vietnam	
		Odds Ratio	95% CI	Odds Ratio	95% CI	Odds Ratio	95% CI	Odds Ratio	95% CI
COVID-19 related changes/behaviours									
	Risk perception: believe they are at medium/high risk	1.595* (0.43)	[0.942,2.703]	0.956 (0.22)	[0.607,1.505]	1.610* 0.41)	[0.980,2.646]	1.672 (0.58)	[0.851,3.287]
	Left house for any reason in the past 7 days	1.216 (0.69)	[0.402,3.672]	1.780 (0.63)	[0.890,3.559]	1.778 (0.78)	[0.753,4.195]	0.287***(0.14)	[0.113,0.731]
	Difference subjective well-being between Round 5 (2016) and	1.221***(0.08)	[1.082,1.377]	1.175***(0.07)	[1.043,1.324]	1.076 (0.08)	[0.926,1.250]	1.129 (0.12)	[0.915,1.393]

Depression	OC	Ethiopia		India		Peru		Vietnam	
		Odds Ratio	95% CI	Odds Ratio	95% CI	Odds Ratio	95% CI	Odds Ratio	95% CI
	phone survey (2020)								
Change in responsibilities:									
	Spend more time taking care of children	0.517** (0.16)	[0.279,0.959]	1.135 (0.33)	[0.637,2.023]	0.707 (0.20)	[0.401,1.247]	1.252 (0.50)	[0.571,2.746]
	Spend more time on household chores	1.457 (0.54)	[0.708,3.002]	0.858 (0.24)	[0.499,1.476]	1.026 (0.33)	[0.543,1.940]	0.912 (0.33)	[0.448,1.858]
	Spend more time working in the family business	1.215 (0.42)	[0.614,2.407]	1.258 (0.73)	[0.402,3.938]	1.534 (0.53)	[0.779,3.022]	1.888 (0.87)	[0.763,4.673]
Economic shocks									
	Faced with new health expenses	1.278 (0.43)	[0.657,2.487]	1.821** (0.48)	[1.089,3.045]	1.226 (0.31)	[0.745,2.017]	0.681 (0.39)	[0.223,2.079]
	Experienced adversity but did not reduce food consumption	1.566 (0.69)	[0.661,3.713]	1.027 (1.15)	[0.114,9.237]	0.463 (0.57)	[0.042,5.082]	1.513 (0.55)	[0.744,3.074]
	Reduced food consumption as response to experienced adversity	4.926***(2.50)	[1.825,13.296]	0.520 (0.66)	[0.042,6.360]	0.363 (0.48)	[0.027,4.887]	1.005 (0.51)	[0.374,2.699]
Changes in employment status									
	Did not work before the pandemic, but is working now[3]	3.429***(1.59)	[1.382,8.507]	0.270* (0.20)	[0.061,1.185]	1.044 (0.69)	[0.285,3.829]	1.000 (.)	[1.000,1.000]
	Worked before the pandemic and is working now/has a job	1.588 (0.45)	[0.915,2.758]	0.740 (0.21)	[0.422,1.297]	0.943 (0.43)	[0.387,2.299]	0.634 (0.37)	[0.203,1.978]
	Worked before the pandemic and is not working now/does not have a job	2.360** (0.87)	[1.145,4.865]	1.267 (0.55)	[0.541,2.968]	1.986 (1.00)	[0.741,5.323]	1.747 (1.07)	[0.523,5.832]
Background characteristics									
	Age in months	1.018 (0.03)	[0.962,1.077]	1.065**(0.03)	[1.006,1.127]	0.984 (0.03)	[0.927,1.044]	0.985 (0.04)	[0.915,1.060]
	Female	0.834 (0.30)	[0.413,1.683]	1.301 (0.41)	[0.705,2.403]	1.266 (0.35)	[0.734,2.185]	1.093 (0.37)	[0.567,2.107]
	Urban	1.395 (0.36)	[0.846,2.298]	0.549**(0.16)	[0.313,0.961]	1.162 (0.50)	[0.496,2.722]	0.512**(0.17)	[0.266,0.988]
	Participant has long-term health problem, 2016 (Round 5)	1.129 (0.40)	[0.562,2.268]	1.636* (0.46)	[0.941,2.844]	2.246**(0.74)	[1.176,4.291]	1.036 (0.35)	[0.530,2.025]
	Total parent-child relationship score, 2012/2016 (Round 4/5)	1.003 (0.04)	[0.934,1.078]	0.953 (0.04)	[0.877,1.035]	1.010 (0.03)	[0.944,1.080]	1.059 (0.06)	[0.950,1.180]

Depression	OC	Ethiopia		India		Peru		Vietnam	
		Odds Ratio	95% CI	Odds Ratio	95% CI	Odds Ratio	95% CI	Odds Ratio	95% CI
	Total peer-child relationship score, 2016 (Round 5)	1.049 (0.04)	[0.971,1.133]	1.041 (0.04)	[0.963,1.125]	0.943 (0.04)	[0.868,1.026]	1.001 (0.08)	[0.852,1.177]
	Middle/Top wealth tercile R5, 2016 (Round 5)	0.732 (0.19)	[0.445,1.205]	0.867 (0.22)	[0.522,1.440]	2.247**(0.77)	[1.144,4.414]	0.840 (0.27)	[0.445,1.585]
Proxy baseline information									
	Emotional problem scale (EPS) score, 2009 (Round 3)	1.090* (0.05)	[0.995,1.195]	1.101* (0.06)	[0.991,1.223]	1.129**(0.07)	[1.003,1.270]	1.114 (0.08)	[0.973,1.276]
	Subjective well-being, 2009 (Round 3)	0.894 (0.06)	[0.782,1.023]	1.077 (0.07)	[0.952,1.219]	0.869* (0.07)	[0.736,1.026]	0.952 (0.08)	[0.805,1.126]
	N	650		836		391		683	

*Note: Odds ratios are unadjusted odds ratios. Robust standard errors in parenthesis, *** significant at 1%, ** significant at 5%, * significant at 10%. Base categories are as follows: Believe they are at no/low risk, Did not leave the house at all during the past 7 days, Did not spend more time taking care of children, Did not spend more time on household chores, Did not spend more time working in the family business, Did not face new health expenses, Did not suffer a shock, Did not work at all in the past 12 month OR worked during the pandemic but not before and is not working now, Male, Rural, Does not have long-term health condition, Lowest wealth tercile. All time-variant variables are measured in 2020 unless otherwise specified. [3]In Vietnam only 17 people did not work before the pandemic but are working now. None showed depressive symptoms. As it predicts failure perfectly it drops out of the regressions.*

Supplementary Table 10. Ethiopia: Means of continuous control variables by at least mild anxiety/depression

		All	at least mild anxiety	no anxiety	p-value	at least mild depression	no depression	p-value
COVID-19 related changes/behaviours								
	Difference in subjective well-being between Round 5 (2016) and phone survey (2020)	0.96	1.15	0.92	0.052	1.26	0.91	0.005
Background characteristics								
	Age in months	250.86	264.14	247.97	0.000	255.55	250.00	0.014
	Total parent-child relationship score, 2012/2016 (Round 4/5)	26.26	25.98	26.32	0.036	26.20	26.27	0.686
	Total peer-child relationship score, 2016 (Round 5)	24.13	23.51	24.26	0.000	23.58	24.23	0.000
Proxy baseline information								
	Emotional problem scale (EPS) score, 2009 (Round 3)²	2.78	3.00	2.70	0.173	3.13	2.71	0.096
	Subjective well-being, 2009 (Round 3)²	5.22	5.18	5.23	0.649	5.24	5.22	0.896

Note: *Results are for the combined Younger Cohort/ Older Cohort sample unless indicated otherwise, ² refers to Older Cohort (25-26) only.*

Supplementary Table 11. India: Means of continuous control variables by at least mild anxiety/depression

		All	at least mild anxiety	no anxiety	p-value	at least mild depression	no depression	p-value
COVID-19 related changes/behaviours								
	Difference in subjective well-being between Round 5 (2016) and phone survey (2020)	0.49	0.77	0.45	0.009	0.86	0.45	0.001
Background characteristics								
	Age in months	253.30	261.16	252.32	0.000	258.12	252.77	0.037
	Total parent-child relationship score, 2012/2016 (Round 4/5)	27.63	26.94	27.72	0.000	26.84	27.72	0.000
	Total peer-child relationship score, 2016 (Round 5)	25.02	24.97	25.03	0.743	24.91	25.03	0.468
Proxy baseline information								
	Emotional problem scale (EPS) score, 2009 (Round 3)²	3.57	3.93	3.51	0.067	4.14	3.49	0.010
	Subjective well-being, 2009 (Round 3)²	5.00	5.29	4.96	0.013	4.97	5.00	0.850

Note: *Results are for the combined Younger Cohort/ Older Cohort sample unless indicated otherwise, ² refers to Older Cohort (25-26) only.*

Supplementary Table 12. Peru: Means of continuous control variables by at least mild anxiety/depression

		All	at least mild anxiety	no anxiety	p-value	at least mild depression	no depression	p-value
COVID-19 related changes/behaviours								
	Difference in subjective well-being in Round 5 (2016) and phone survey (2020)	0.52	0.74	0.38	0.000	0.70	0.44	0.013
Background characteristics								
	Age in months	244.23	245.19	243.57	0.315	242.56	245.00	0.151
	Total parent-child relationship score, 2012/2016 (Round 4/5)	24.86	24.60	25.04	0.009	24.28	25.12	0.000
	Total peer-child relationship score, 2016 (Round 5)	23.41	23.40	23.42	0.862	23.41	23.42	0.954
Proxy baseline information								
	Emotional problem scale (EPS) score, 2009 (Round 3)²	4.27	4.87	3.81	0.000	4.85	4.03	0.002
	Subjective well-being, 2009 (Round 3)²	6.82	6.72	6.89	0.073	6.72	6.87	0.135

Note: Results are for the combined Younger Cohort/ Older Cohort sample unless indicated otherwise, ² refers to Older Cohort (25-26) only.

Supplementary Table 13. Vietnam: Means of continuous control variables by at least mild anxiety/depression

		All	at least mild anxiety	no anxiety	p-value	at least mild depression	no depression	p-value
COVID-19 related changes/behaviours								
	Difference in subjective well-being in Round 5 (2016) and phone survey (2020)	-0.39	0.14	-0.45	0.000	0.11	-0.45	0.000
Background characteristics								
	Age in months	252.95	248.28	253.43	0.067	246.49	253.63	0.010
	Total parent-child relationship score, 2012/2016 (Round 4/5)	25.74	26.11	25.70	0.073	25.72	25.74	0.930
	Total peer-child relationship score, 2016 (Round 5)	22.57	22.40	22.59	0.230	22.52	22.58	0.704
Proxy baseline information								
	Emotional problem scale (EPS) score, 2009 (Round 3)²	3.68	3.96	3.65	0.314	4.18	3.64	0.086
	Subjective well-being, 2009 (Round 3)²	5.84	5.73	5.86	0.383	5.85	5.84	0.943

Note: *Results are for the combined Younger Cohort/ Older Cohort sample unless indicated otherwise, ² refers to Older Cohort (25-26) only.*

Supplementary Table 14. Mental health outcomes by control variables (Ethiopia & India)

	Ethiopia				India			
	% at least mild Anxiety	p-value	% at least mild Depression	p-value	% at least mild Anxiety	p-value	% at least mild Depression	p-value
COVID-19 related changes/behaviours								
Risk perception: believe they are at no/low risk	20.30	0.048	20.75	0.000	10.10	0.092	10.10	0.735
Risk perception: believe they are at medium/high risk	16.79		13.09		12.17		9.70	
Did not leave house for any reason in the past 7 days	22.50	0.173	18.33	0.367	8.46	0.030	5.51	0.000
Left house for any reason in the past 7 days	17.60		15.27		11.74		11.07	
Change in responsibilities:								
Did not spend more time taking care of children	18.06	0.704	15.55	0.823	9.01	0.000	8.82	0.000
Spend more time taking care of children	17.38		15.17		20.21		14.79	
Did not spend more time on household chores	17.83	0.963	14.64	0.273	10.58	0.438	9.72	0.742
Spend more time on household chores	17.91		16.34		11.53		10.10	
Did not spend more time working in the family business	18.26	0.209	15.63	0.516	10.90	0.204	9.90	0.924
Spend more time working in the family business	15.19		14.13		14.81		10.19	
Economic shocks								
Was not faced with new health expenses	17.43	0.115	15.75	0.230	10.91	0.827	8.57	0.041
Faced with new health expenses	21.72		12.67		11.18		10.97	
Did not experience adversity	6.86	0.000	4.65	0.000	13.33	0.381	11.11	0.956
Experienced adversity but did not reduce food consumption	17.54		15.59		10.82		9.88	
Reduced food consumption as response to experienced adversity	36.39		31.29		14.29		10.20	
Changes in employment status								
Did not work at all in the past 12 month OR worked during the pandemic but not before the pandemic (and is not working now)	13.79	0.000	13.10	0.000	9.22	0.000	9.03	0.059
Did not work before the pandemic, but is working now	26.17		25.50		9.07		8.37	
Worked before the pandemic and is working now/has a job	18.34		14.91		12.89		10.86	
Worked before the pandemic and is not working now/does not have a job	31.49		22.65		19.67		15.57	
Educational disruption								
Never attended school or not enrolled in full-time education/not planning to enrol in learning activities[1]	18.25	0.212	13.45	0.056	13.01	0.000	11.00	0.012
Enrolled in/Planning to enrol in full time education and	16.58		17.41		10.69		10.40	

not participating in learning activities[1]								
Enrolled in/Planning to enrol in full time education and participating in learning activities[1]	21.13		15.47		6.57		6.57	
Background characteristics								
Participant does not have a long-term health problem, 2016 (Round 5)	17.13	0.004	15.42	0.940	10.47	0.007	9.31	0.004
Participant has long-term health problem, 2016 (Round 5)	25.52		15.63		15.61		14.62	

Notes: If any missing answers to questions then the whole score is set to missing. p-value indicates significance of t-tests between dummy variables and o F-tests in case of categorical variables. Results are for the combined Younger Cohort/ Older Cohort sample unless indicated otherwise, [1] refers to Younger Cohort (18-19) only.

Supplementary Table 15. Mental health outcomes by control variables (Peru & Vietnam)

	Peru				Vietnam			
	**% at least mild Anxiety**	p-value	**% at least mild Depression**	p-value	**% at least mild Anxiety**	p-value	**% at least mild Depression**	p-value
COVID-19 related changes/behaviours								
Risk perception: believe they are at no/low risk	35.34	0.000	25.84	0.000	9.31	0.967	9.31	0.557
Risk perception: believe they are at medium/high risk	45.51		36.43		9.37		10.18	
Did not leave house for any reason in the past 7 days	42.82	0.393	33.33	0.421	9.54	0.892	11.66	0.184
Left house for any reason in the past 7 days	40.38		31.16		9.29		9.19	
Change in responsibilities:								
Did not spend more time taking care of children	36.83	0.000	29.57	0.009	8.19	0.001	8.19	0.000
Spend more time taking care of children	48.61		35.45		13.12		13.88	
Did not spend more time on household chores	33.96	0.001	28.77	0.157	7.44	0.003	6.46	0.000
Spend more time on household chores	42.86		32.40		11.09		12.36	
Did not spend more time working in the family business	40.09	0.105	30.92	0.141	8.46	0.000	8.60	0.000
Spend more time working in the family business	45.23		35.34		16.40		16.80	
Economic shocks								
Was not faced with new health expenses	36.12	0.000	27.24	0.000	8.99	0.041	9.27	0.171
Faced with new health expenses	52.15		41.94		14.00		12.67	

	Peru				Vietnam			
	**% at least mild Anxiety**	p-value	**% at least mild Depression**	p-value	**% at least mild Anxiety**	p-value	**% at least mild Depression**	p-value
Did not experience adversity	16.33	0.002	14.29	0.004	6.49	0.000	6.29	0.000
Experienced adversity but did not reduce food consumption	41.32		31.47		10.80		11.11	
Reduced food consumption as response to experienced adversity	44.26		40.16		12.95		13.77	
Changes in employment status								
Did not work at all in the past 12 month OR worked during the pandemic but not before the pandemic (and is not working now)	41.41	0.176	33.26	0.003	8.53	0.269	9.83	0.002
Did not work before the pandemic, but is working now	37.77		32.19		10.97		8.39	
Worked before the pandemic and is working now/has a job	39.57		27.89		8.81		8.11	
Worked before the pandemic and is not working now/does not have a job	45.91		38.99		12.01		15.26	
Educational disruption								
Never attended school or not enrolled in full-time education/not planning to enrol in learning activities[1]	40.74	0.611	30.74	0.490	9.95	0.374	8.91	0.387
Enrolled in/Planning to enrol in full time education and not participating in learning activities[1]	32.14		28.57		8.96		9.43	
Enrolled in/Planning to enrol in full time education and participating in learning activities[1]	41.47		33.33		8.04		10.82	
Background characteristics								
Participant does not have a long-term health problem, 2016 (Round 5)	38.71	0.000	29.85	0.000	9.04	0.248	9.14	0.151
Participant has long-term health problem, 2016 (Round 5)	55.65		43.51		11.04		11.66	

Notes: If any missing answers to questions then the whole score is set to missing. p-value indicates significance of t-tests between dummy variables and o F-tests in case of categorical variables. Results are for the combined Younger Cohort/ Older Cohort sample unless indicated otherwise, [1] refers to Younger Cohort (18-19) only.

Supplementary Table 16. Logistic regression results: Symptoms of at least mild anxiety/depression by gender (Ethiopia)

	At least mild anxiety				At least mild depression			
	Male		Female		Male		Female	
	Odds Ratio	95% CI	Odds Ratio	95% CI	Odds Ratio	95% CI	Odds Ratio	95% CI
COVID-19 related changes/behaviours								
Risk perception: believe they are at medium/high risk	0.807 (0.14)	[0.569,1.146]	0.825 (0.16)	[0.562,1.211]	0.576*** (0.10)	[0.403,0.824]	0.659** (0.13)	[0.452,0.961]
Left house for any reason in the past 7 days	0.593 (0.29)	[0.229,1.533]	0.847 (0.25)	[0.480,1.494]	0.556 (0.25)	[0.233,1.327]	1.126 (0.35)	[0.614,2.064]
Difference in subjective well-being between Round 5 (2016) and phone survey (2020)	1.097** (0.04)	[1.016,1.185]	1.052 (0.05)	[0.967,1.146]	1.084* (0.05)	[0.999,1.177]	1.114** (0.05)	[1.019,1.218]
Change in responsibilities:								
Spend more time taking care of children	1.262 (0.34)	[0.747,2.132]	0.749 (0.14)	[0.513,1.093]	0.791 (0.21)	[0.468,1.339]	0.957 (0.20)	[0.640,1.433]
Spend more time on household chores	0.869 (0.20)	[0.558,1.353]	1.447 (0.33)	[0.929,2.252]	1.181 (0.25)	[0.776,1.797]	1.162 (0.27)	[0.731,1.845]
Spend more time working in the family business	0.750 (0.20)	[0.448,1.256]	0.713 (0.24)	[0.367,1.384]	0.643 (0.19)	[0.361,1.144]	1.498 (0.47)	[0.814,2.755]
Economic shocks								
Faced with new health expenses	0.733 (0.21)	[0.419,1.281]	1.115 (0.30)	[0.660,1.886]	0.438*** (0.13)	[0.241,0.797]	0.592* (0.19)	[0.319,1.096]
Experienced adversity but did not reduce food consumption	1.889** (0.53)	[1.091,3.270]	3.063*** (1.00)	[1.619,5.793]	3.658*** (1.35)	[1.776,7.537]	3.739*** (1.28)	[1.908,7.326]
Reduced food consumption as response to experienced adversity	5.891*** (1.92)	[3.107,11.172]	8.642*** (3.16)	[4.222,17.690]	12.987*** (5.25)	[5.884,28.663]	8.585*** (3.32)	[4.026,18.308]
Changes in employment status								
Did not work before the pandemic, but is working now	2.623*** (0.88)	[1.356,5.075]	2.853*** (0.97)	[1.470,5.538]	2.426*** (0.79)	[1.283,4.591]	2.754*** (0.99)	[1.357,5.590]
Worked before the pandemic and is working now/has a job	1.335 (0.30)	[0.859,2.074]	1.981*** (0.46)	[1.260,3.115]	1.057 (0.23)	[0.684,1.634]	1.744** (0.41)	[1.096,2.776]
Worked before the pandemic and is not working now/does not have a job	2.196*** (0.61)	[1.268,3.801]	2.239*** (0.64)	[1.279,3.918]	1.020 (0.33)	[0.540,1.927]	2.647*** (0.79)	[1.476,4.746]
Background characteristics								
Age in months	1.012*** (0.00)	[1.007,1.016]	1.003 (0.00)	[0.999,1.008]	1.007*** (0.00)	[1.002,1.012]	0.996* (0.00)	[0.991,1.001]

	At least mild anxiety				At least mild depression			
	Male		Female		Male		Female	
	Odds Ratio	95% CI	Odds Ratio	95% CI	Odds Ratio	95% CI	Odds Ratio	95% CI
Urban	1.155 (0.22)	[0.799,1.670]	1.523** (0.32)	[1.003,2.312]	1.307 (0.26)	[0.880,1.942]	1.440* (0.31)	[0.938,2.210]
Participant has long-term health problem, 2016 (Round 5)	1.848** (0.53)	[1.054,3.241]	1.204 (0.29)	[0.745,1.944]	0.919 (0.33)	[0.450,1.875]	0.998 (0.28)	[0.580,1.716]
Total parent-child relationship score, 2012/2016 (Round 4/5)	0.968 (0.03)	[0.914,1.026]	0.962 (0.03)	[0.902,1.026]	1.027 (0.03)	[0.965,1.092]	0.972 (0.03)	[0.908,1.039]
Total peer-child relationship score, 2016 (Round 5)	0.935** (0.03)	[0.877,0.998]	0.944* (0.03)	[0.890,1.002]	0.948 (0.03)	[0.884,1.017]	0.932** (0.03)	[0.875,0.993]
Middle/Top wealth tercile R5, 2016 (Round 5)	1.199 (0.24)	[0.811,1.773]	0.985 (0.21)	[0.643,1.511]	0.882 (0.18)	[0.587,1.327]	0.844 (0.19)	[0.539,1.321]
N	1173		1010		1173		1010	

*Note: Odds ratios are unadjusted odds ratios. Robust standard errors in parenthesis, *** significant at 1%, ** significant at 5%, * significant at 10%. Base categories are as follows: Believe they are at no/low risk, Did not leave the house at all during the past 7 days, Did not spend more time taking care of children, Did not spend more time on household chores, Did not spend more time working in the family business, Did not face new health expenses, Did not suffer a shock, Did not work at all in the past 12 month OR worked during the pandemic but not before and is not working now, Rural, Does not have long-term health condition, Lowest wealth tercile. All time-variant variables are measured in 2020 unless otherwise specified. Results are for the combined Younger Cohort/ Older Cohort sample.*

Supplementary Table 17. Logistic regression results: Symptoms of at least mild anxiety/depression by gender (India)

	At least mild anxiety				At least mild depression			
	Male		Female		Male		Female	
	Odds Ratio	95% CI	Odds Ratio	95% CI	Odds Ratio	95% CI	Odds Ratio	95% CI
COVID-19 related changes/behaviours								
Risk perception: believe they are at medium/high risk	1.520** (0.30)	[1.038,2.224]	1.149 (0.22)	[0.795,1.661]	1.048 (0.20)	[0.722,1.522]	0.927 (0.19)	[0.616,1.395]
Left house for any reason in the past 7 days	0.744 (0.28)	[0.356,1.555]	1.606** (0.36)	[1.040,2.482]	1.063 (0.43)	[0.482,2.342]	2.810*** (0.77)	[1.642,4.810]
Difference in subjective well-being between Round 5 (2016) and phone survey (2020)	0.999 (0.05)	[0.904,1.105]	1.245*** (0.07)	[1.121,1.383]	1.041 (0.05)	[0.950,1.141]	1.248*** (0.07)	[1.117,1.395]
Change in responsibilities:								
Spend more time taking care of children	1.708* (0.50)	[0.967,3.015]	2.455*** (0.54)	[1.595,3.780]	1.327 (0.41)	[0.721,2.443]	1.668** (0.43)	[1.007,2.761]
Spend more time on household chores	1.356	[0.923,1.993]	0.440***	[0.297,0.651]	1.337	[0.915,1.955]	0.576**	[0.376,0.883]

	At least mild anxiety				At least mild depression			
	Male		Female		Male		Female	
	Odds Ratio	95% CI	Odds Ratio	95% CI	Odds Ratio	95% CI	Odds Ratio	95% CI
	(0.27)		(0.09)		(0.26)		(0.13)	
Spend more time working in the family business	1.944*	[0.953,3.967]	1.210	[0.520,2.816]	1.100	[0.493,2.457]	1.043	[0.348,3.122]
	(0.71)		(0.52)		(0.45)		(0.58)	
Economic shocks								
Faced with new health expenses	0.786	[0.532,1.161]	1.349	[0.924,1.968]	1.203	[0.799,1.810]	1.477*	[0.969,2.250]
	(0.16)		(0.26)		(0.25)		(0.32)	
Experienced adversity but did not reduce food consumption	0.894	[0.169,4.723]	0.500	[0.155,1.609]	1.749	[0.207,14.799]	0.384*	[0.130,1.137]
	(0.76)		(0.30)		(1.91)		(0.21)	
Reduced food consumption as response to experienced adversity	1.272	[0.209,7.752]	0.691	[0.177,2.697]	1.651	[0.169,16.089]	0.398	[0.099,1.608]
	(1.17)		(0.48)		(1.92)		(0.28)	
Changes in employment status								
Did not work before the pandemic, but is working now	0.629	[0.326,1.213]	1.267	[0.687,2.334]	0.555*	[0.294,1.051]	0.887	[0.472,1.669]
	(0.21)		(0.40)		(0.18)		(0.29)	
Worked before the pandemic and is working now/has a job	0.982	[0.562,1.718]	1.504*	[0.978,2.314]	0.980	[0.591,1.626]	0.802	[0.485,1.328]
	(0.28)		(0.33)		(0.25)		(0.21)	
Worked before the pandemic and is not working now/does not have a job	2.812**	[1.266,6.245]	2.286**	[1.121,4.660]	2.908***	[1.367,6.188]	0.836	[0.325,2.148]
	(1.14)		(0.83)		(1.12)		(0.40)	
Background characteristics								
Age in months	0.999	[0.994,1.004]	1.003	[0.998,1.008]	0.998	[0.993,1.003]	1.002	[0.996,1.008]
	(0.00)		(0.00)		(0.00)		(0.00)	
Urban	0.428***	[0.252,0.728]	1.564**	[1.012,2.417]	0.502***	[0.311,0.808]	1.113	[0.679,1.824]
	(0.12)		(0.35)		(0.12)		(0.28)	
Participant has long-term health problem, 2016 (Round 5)	1.310	[0.746,2.299]	1.466	[0.924,2.326]	1.353	[0.773,2.366]	1.785**	[1.105,2.885]
	(0.38)		(0.35)		(0.39)		(0.44)	
Total parent-child relationship score, 2012/2016 (Round 4/5)	0.908***	[0.845,0.977]	0.934**	[0.880,0.991]	0.908***	[0.846,0.973]	0.909***	[0.848,0.974]
	(0.03)		(0.03)		(0.03)		(0.03)	
Total peer-child relationship score, 2016 (Round 5)	1.039	[0.965,1.119]	1.014	[0.944,1.089]	1.040	[0.961,1.125]	0.994	[0.924,1.069]
	(0.04)		(0.04)		(0.04)		(0.04)	
Middle/Top wealth tercile R5, 2016 (Round 5)	0.804	[0.534,1.210]	0.764	[0.504,1.159]	1.324	[0.881,1.989]	0.877	[0.559,1.375]
	(0.17)		(0.16)		(0.27)		(0.20)	
N	1369		1253		1369		1253	

*Note: Odds ratios are unadjusted odds ratios. Robust standard errors in parenthesis, *** significant at 1%, ** significant at 5%, * significant at 10%. Base categories are as follows: Believe they are at no/low risk, Did not leave the house at all during the past 7 days, Did not spend more time taking care of children, Did not spend more time on household chores, Did not spend more time working in the family business, Did not face new health expenses, Did not suffer a shock, Did not work at all in the past 12 month OR worked during the pandemic but not before and is not working now, Rural, Does not have long-term health condition, Lowest wealth tercile. All time-variant variables are measured in 2020 unless otherwise specified. Results are for the combined Younger Cohort/ Older Cohort sample.*

Supplementary Table 18. Logistic regression results: Symptoms of at least mild anxiety/depression by gender (Peru)

	At least mild anxiety				At least mild depression			
	Male		Female		Male		Female	
	Odds Ratio	95% CI	Odds Ratio	95% CI	Odds Ratio	95% CI	Odds Ratio	95% CI
COVID-19 related changes/behaviours								
Risk perception: believe they are at medium/high risk	1.326* (0.19)	[0.994,1.767]	1.585*** (0.22)	[1.204,2.086]	1.513*** (0.23)	[1.117,2.051]	1.657*** (0.24)	[1.241,2.212]
Left house for any reason in the past 7 days	1.324 (0.28)	[0.878,1.996]	0.848 (0.14)	[0.609,1.181]	1.052 (0.22)	[0.697,1.588]	1.067 (0.19)	[0.756,1.505]
Difference in subjective well-being between Round 5 (2016) and phone survey (2020)	1.182*** (0.05)	[1.097,1.274]	1.064* (0.03)	[0.998,1.134]	1.131*** (0.05)	[1.043,1.227]	1.047 (0.04)	[0.978,1.121]
Change in responsibilities:								
Spend more time taking care of children	1.567*** (0.26)	[1.134,2.165]	1.285* (0.18)	[0.973,1.698]	1.529** (0.26)	[1.097,2.131]	0.932 (0.14)	[0.697,1.246]
Spend more time on household chores	1.220 (0.21)	[0.873,1.704]	1.293 (0.25)	[0.882,1.895]	1.039 (0.18)	[0.734,1.471]	1.105 (0.22)	[0.742,1.644]
Spend more time working in the family business	1.299 (0.25)	[0.896,1.885]	1.335 (0.29)	[0.871,2.046]	1.258 (0.25)	[0.848,1.865]	1.362 (0.30)	[0.883,2.101]
Economic shocks								
Faced with new health expenses	1.983*** (0.32)	[1.451,2.710]	1.532*** (0.23)	[1.148,2.044]	1.735*** (0.29)	[1.257,2.394]	1.757*** (0.26)	[1.308,2.361]
Experienced adversity but did not reduce food consumption	5.087** (3.94)	[1.114,23.224]	1.609 (0.88)	[0.548,4.728]	4.172* (3.08)	[0.983,17.705]	1.056 (0.63)	[0.330,3.378]
Reduced food consumption as response to experienced adversity	5.843** (4.83)	[1.157,29.524]	1.288 (0.77)	[0.397,4.178]	5.041** (4.02)	[1.057,24.045]	1.354 (0.87)	[0.383,4.782]
Changes in employment status								
Did not work before the pandemic, but is working now	0.684 (0.21)	[0.379,1.235]	0.945 (0.23)	[0.591,1.510]	1.065 (0.32)	[0.593,1.910]	0.790 (0.19)	[0.491,1.271]
Worked before the pandemic and is working now/has a job	0.958 (0.22)	[0.608,1.509]	1.049 (0.19)	[0.737,1.493]	0.899 (0.22)	[0.563,1.437]	0.799 (0.15)	[0.551,1.158]
Worked before the pandemic and is not working now/does not have a job	1.029 (0.27)	[0.616,1.719]	1.326 (0.26)	[0.900,1.952]	1.385 (0.37)	[0.824,2.329]	1.193 (0.25)	[0.797,1.786]
Background characteristics								
Age in months	1.001 (0.00)	[0.997,1.005]	0.998 (0.00)	[0.994,1.002]	0.996* (0.00)	[0.991,1.001]	0.996* (0.00)	[0.992,1.000]
Urban	1.245 (0.27)	[0.817,1.898]	1.610** (0.34)	[1.070,2.423]	0.978 (0.21)	[0.639,1.498]	1.222 (0.27)	[0.790,1.889]
Participant has long-term health problem, 2016 (Round 5)	1.794** (0.41)	[1.142,2.818]	1.937*** (0.38)	[1.318,2.848]	1.662** (0.39)	[1.047,2.640]	1.564** (0.29)	[1.081,2.262]
Total parent-child relationship score, 2012/2016	0.974	[0.931,1.020]	0.982	[0.947,1.019]	0.944**	[0.898,0.992]	0.936***	[0.900,0.973]

	At least mild anxiety				At least mild depression			
	Male		Female		Male		Female	
	Odds Ratio	95% CI	Odds Ratio	95% CI	Odds Ratio	95% CI	Odds Ratio	95% CI
(Round 4/5)	(0.02)		(0.02)		(0.02)		(0.02)	
Total peer-child relationship score, 2016 (Round 5)	1.010 (0.03)	[0.958,1.065]	0.990 (0.02)	[0.951,1.031]	1.002 (0.03)	[0.946,1.063]	1.011 (0.02)	[0.968,1.055]
Middle/Top wealth tercile R5, 2016 (Round 5)	0.777 (0.14)	[0.548,1.104]	1.257 (0.22)	[0.890,1.774]	1.137 (0.21)	[0.795,1.627]	1.294 (0.24)	[0.894,1.873]
N	957		930		957		930	

*Note: Odds ratios are unadjusted odds ratios. Robust standard errors in parenthesis, *** significant at 1%, ** significant at 5%, * significant at 10%. Base categories are as follows: Believe they are at no/low risk, Did not leave the house at all during the past 7 days, Did not spend more time taking care of children, Did not spend more time on household chores, Did not spend more time working in the family business, Did not face new health expenses, Did not suffer a shock, Did not work at all in the past 12 month OR worked during the pandemic but not before and is not working now, Rural, Does not have long-term health condition, Lowest wealth tercile. All time-variant variables are measured in 2020 unless otherwise specified. Results are for the combined Younger Cohort/ Older Cohort sample.*

Supplementary Table 19. Logistic regression results: Symptoms of at least mild anxiety/depression by gender (Vietnam)

	At least mild anxiety				At least mild depression			
	Male		Female		Male		Female	
	Odds Ratio	95% CI	Odds Ratio	95% CI	Odds Ratio	95% CI	Odds Ratio	95% CI
COVID-19 related changes/behaviours								
Risk perception: believe they are at medium/high risk	1.548* (0.41)	[0.921,2.603]	0.816 (0.21)	[0.498,1.337]	2.094** (0.61)	[1.180,3.715]	0.828 (0.19)	[0.525,1.304]
Left house for any reason in the past 7 days	0.666 (0.22)	[0.353,1.254]	1.535 (0.51)	[0.802,2.936]	0.748 (0.25)	[0.388,1.439]	1.225 (0.36)	[0.694,2.164]
Difference in subjective well-being between Round 5 (2016) and phone survey (2020)	1.296*** (0.10)	[1.116,1.506]	1.111* (0.06)	[0.994,1.241]	1.471*** (0.11)	[1.269,1.706]	1.016 (0.05)	[0.914,1.129]
Change in responsibilities:								
Spend more time taking care of children	1.222 (0.37)	[0.673,2.220]	1.520* (0.35)	[0.971,2.381]	1.221 (0.39)	[0.648,2.300]	1.506* (0.34)	[0.970,2.339]
Spend more time on	1.191 (0.27)	[0.757,1.872]	1.191 (0.26)	[0.777,1.825]	1.076 (0.27)	[0.655,1.770]	1.996*** (0.46)	[1.277,3.122]

	At least mild anxiety				At least mild depression			
	Male		Female		Male		Female	
	**Odds Ratio**	**95% CI**	**Odds Ratio**	**95% CI**	**Odds Ratio**	**95% CI**	**Odds Ratio**	**95% CI**
household chores								
Spend more time working in the family business	2.394*** (0.75)	[1.292,4.434]	1.624* (0.44)	[0.950,2.777]	2.373*** (0.76)	[1.261,4.463]	1.875** (0.48)	[1.132,3.106]
Economic shocks								
Faced with new health expenses	0.544 (0.29)	[0.191,1.547]	1.757* (0.57)	[0.933,3.309]	1.093 (0.49)	[0.453,2.636]	0.721 (0.27)	[0.345,1.508]
Experienced adversity but did not reduce food consumption	2.494*** (0.70)	[1.440,4.320]	1.230 (0.28)	[0.787,1.925]	2.430*** (0.71)	[1.370,4.309]	1.416 (0.33)	[0.902,2.224]
Reduced food consumption as response to experienced adversity	2.367** (0.86)	[1.159,4.833]	1.357 (0.38)	[0.785,2.346]	1.883* (0.72)	[0.889,3.987]	1.946** (0.51)	[1.162,3.259]
Changes in employment status								
Did not work before the pandemic, but is working now	1.605 (0.84)	[0.574,4.486]	0.814 (0.34)	[0.357,1.857]	0.254 (0.25)	[0.036,1.782]	0.913 (0.34)	[0.436,1.910]
Worked before the pandemic and is working now/has a job	1.127 (0.42)	[0.545,2.331]	0.948 (0.24)	[0.575,1.565]	1.157 (0.41)	[0.576,2.324]	0.708 (0.17)	[0.441,1.136]
Worked before the pandemic and is not working now/does not have a job	1.798 (0.72)	[0.817,3.958]	1.178 (0.37)	[0.635,2.187]	1.921* (0.75)	[0.898,4.108]	1.456 (0.40)	[0.848,2.502]
Background characteristics								
Age in months	0.996 (0.00)	[0.990,1.002]	0.994** (0.00)	[0.988,1.000]	0.992** (0.00)	[0.985,0.999]	0.994** (0.00)	[0.988,1.000]
Urban	0.960 (0.22)	[0.608,1.517]	1.358 (0.27)	[0.916,2.014]	0.837 (0.22)	[0.500,1.402]	1.091 (0.22)	[0.740,1.607]
Participant has long-term health problem, 2016 (Round 5)	1.944** (0.58)	[1.088,3.474]	1.055 (0.29)	[0.612,1.819]	2.231** (0.74)	[1.167,4.266]	1.063 (0.28)	[0.631,1.789]
Total parent-child relationship score, 2012/2016 (Round 4/5)	1.015 (0.04)	[0.940,1.096]	1.056 (0.04)	[0.989,1.127]	0.983 (0.04)	[0.904,1.071]	0.982 (0.03)	[0.925,1.043]
Total peer-child relationship score, 2016 (Round 5)	1.014 (0.06)	[0.907,1.133]	0.897** (0.04)	[0.814,0.989]	1.055 (0.07)	[0.928,1.199]	0.961 (0.04)	[0.877,1.052]

	At least mild anxiety				At least mild depression			
	Male		Female		Male		Female	
	**Odds Ratio**	**95% CI**	**Odds Ratio**	**95% CI**	**Odds Ratio**	**95% CI**	**Odds Ratio**	**95% CI**
Middle/Top wealth tercile R5, 2016 (Round 5)	0.551** (0.14)	[0.336,0.903]	0.566*** (0.12)	[0.379,0.844]	1.015 (0.28)	[0.588,1.754]	1.130 (0.24)	[0.745,1.713]
N	1111		1185		1111		1185	

*Note: Odds ratios are unadjusted odds ratios. Robust standard errors in parenthesis, *** significant at 1%, ** significant at 5%, * significant at 10%. Base categories are as follows: Believe they are at no/low risk, Did not leave the house at all during the past 7 days, Did not spend more time taking care of children, Did not spend more time on household chores, Did not spend more time working in the family business, Did not face new health expenses, Did not suffer a shock, Did not work at all in the past 12 month OR worked during the pandemic but not before and is not working now, Rural, Does not have long-term health condition, Lowest wealth tercile. All time-variant variables are measured in 2020 unless otherwise specified. Results are for the combined Younger Cohort/ Older Cohort sample.*

Supplementary Table 20. Logistic regression results: Symptoms of at least mild anxiety/depression by gender for the Younger Cohort only (Ethiopia)

	At least mild anxiety				At least mild depression			
	Male		Female		Male		Female	
	Odds Ratio	95% CI	Odds Ratio	95% CI	Odds Ratio	95% CI	Odds Ratio	95% CI
COVID-19 related changes/behaviours								
Risk perception: believe they are at medium/high risk	0.550** (0.13)	[0.348,0.869]	0.875 (0.22)	[0.540,1.420]	0.461*** (0.11)	[0.290,0.733]	0.512*** (0.12)	[0.324,0.810]
Left house for any reason in the past 7 days	0.620 (0.29)	[0.249,1.545]	1.140 (0.47)	[0.504,2.579]	0.702 (0.34)	[0.269,1.832]	1.129 (0.42)	[0.546,2.336]
Difference in subjective well-being between Round 5 (2016) and phone survey (2020)	1.076 (0.05)	[0.973,1.189]	1.001 (0.06)	[0.897,1.117]	1.015 (0.06)	[0.910,1.131]	1.083 (0.06)	[0.975,1.204]
Change in responsibilities:								
Spend more time taking care of children	1.481 (0.44)	[0.823,2.666]	0.754 (0.19)	[0.460,1.236]	0.839 (0.25)	[0.463,1.522]	1.224 (0.30)	[0.757,1.979]
Spend more time on household chores	0.780 (0.19)	[0.479,1.271]	1.401 (0.41)	[0.793,2.475]	1.083 (0.26)	[0.671,1.749]	1.132 (0.32)	[0.647,1.979]
Spend more time working in the family business	0.542 (0.23)	[0.239,1.233]	0.842 (0.39)	[0.344,2.064]	0.475 (0.22)	[0.189,1.195]	2.336** (0.88)	[1.120,4.872]
Economic shocks								
Faced with new health expenses	0.673 (0.31)	[0.272,1.666]	0.645 (0.28)	[0.273,1.524]	0.278** (0.17)	[0.083,0.931]	0.199** (0.13)	[0.057,0.695]
Experienced adversity but did not reduce food consumption	3.346*** (1.34)	[1.530,7.320]	3.633*** (1.42)	[1.692,7.801]	6.595*** (3.49)	[2.338,18.604]	3.859*** (1.52)	[1.785,8.340]
Reduced food consumption as response to experienced adversity	6.188*** (2.97)	[2.418,15.835]	9.701*** (4.34)	[4.035,23.323]	19.255*** (11.13)	[6.202,59.780]	8.538*** (3.98)	[3.427,21.271]
Educational disruption								
Enrolled in/Planning to enrol in full time education and not participating in learning activities	1.188 (0.33)	[0.693,2.039]	2.556*** (0.87)	[1.313,4.972]	1.520 (0.45)	[0.845,2.732]	2.033** (0.62)	[1.122,3.683]
Enrolled in/Planning to enrol in full time education and participating in learning activities	0.810 (0.46)	[0.269,2.436]	1.600 (0.79)	[0.608,4.207]	0.735 (0.48)	[0.207,2.613]	1.524 (0.68)	[0.635,3.656]
Background characteristics								
Age in months	1.011 (0.03)	[0.952,1.074]	0.990 (0.03)	[0.937,1.047]	1.005 (0.03)	[0.946,1.068]	0.997 (0.03)	[0.944,1.054]
Urban	1.421 (0.39)	[0.831,2.430]	1.712* (0.51)	[0.953,3.075]	1.341 (0.37)	[0.783,2.296]	1.634* (0.45)	[0.956,2.792]
Participant has long-term health problem, 2016 (Round 5)	1.185 (0.57)	[0.464,3.028]	1.291 (0.47)	[0.630,2.646]	0.358 (0.26)	[0.086,1.494]	0.621 (0.28)	[0.260,1.483]

	At least mild anxiety				At least mild depression			
	Male		Female		Male		Female	
	Odds Ratio	95% CI	Odds Ratio	95% CI	Odds Ratio	95% CI	Odds Ratio	95% CI
Total parent-child relationship score, 2012/2016 (Round 4/5)	0.971 (0.04)	[0.890,1.059]	0.943 (0.04)	[0.858,1.035]	1.056 (0.04)	[0.974,1.145]	0.970 (0.04)	[0.889,1.059]
Total peer-child relationship score, 2016 (Round 5)	0.934 (0.05)	[0.848,1.028]	0.970 (0.04)	[0.893,1.054]	0.892** (0.05)	[0.808,0.985]	0.915** (0.04)	[0.842,0.994]
Middle/Top wealth tercile R5, 2016 (Round 5)	0.922 (0.26)	[0.528,1.611]	0.923 (0.28)	[0.507,1.682]	0.792 (0.23)	[0.453,1.383]	0.756 (0.21)	[0.434,1.315]
N	825		708		825		708	

*Note: Odds ratios are unadjusted odds ratios. Robust standard errors in parenthesis, *** significant at 1%, ** significant at 5%, * significant at 10%. Base categories are as follows: Believe they are at no/low risk, Did not leave the house at all during the past 7 days, Did not spend more time taking care of children, Did not spend more time on household chores, Did not spend more time working in the family business, Did not face new health expenses, Did not suffer a shock, Never attended school or not enrolled in full-time education/not planning to enrol, Rural, Does not have long-term health condition, Lowest wealth tercile. All time-variant variables are measured in 2020 unless otherwise specified.*

Supplementary Table 21. Logistic regression results: Symptoms of at least mild anxiety/depression by gender for the Younger Cohort only (India)

	At least mild anxiety				At least mild depression			
	Male		Female		Male		Female	
	Odds Ratio	95% CI	Odds Ratio	95% CI	Odds Ratio	95% CI	Odds Ratio	95% CI
<u>*COVID-19 related changes/behaviours*</u>								
Risk perception: believe they are at medium/high risk	1.455 (0.34)	[0.914,2.315]	0.995 (0.25)	[0.605,1.639]	0.969 (0.22)	[0.622,1.509]	0.989 (0.26)	[0.586,1.669]
Left house for any reason in the past 7 days	0.517* (0.21)	[0.238,1.125]	2.471*** (0.77)	[1.337,4.565]	0.913 (0.39)	[0.392,2.124]	3.923*** (1.55)	[1.811,8.496]
Difference in subjective well-being between Round 5 (2016) and phone survey (2020)	1.012 (0.06)	[0.898,1.141]	1.243*** (0.09)	[1.083,1.427]	1.027 (0.06)	[0.919,1.148]	1.215*** (0.09)	[1.058,1.395]
Change in responsibilities:								
Spend more time taking care of children	2.137* (0.90)	[0.935,4.884]	2.311** (0.76)	[1.213,4.403]	1.118 (0.58)	[0.407,3.074]	3.427*** (1.13)	[1.798,6.534]
Spend more time on household chores	1.370 (0.35)	[0.826,2.274]	0.517*** (0.13)	[0.317,0.843]	1.207 (0.30)	[0.738,1.973]	0.673 (0.19)	[0.390,1.162]
Spend more time working in the family business	2.020* (0.86)	[0.879,4.640]	2.424** (1.09)	[1.005,5.849]	0.750 (0.38)	[0.278,2.021]	0.832 (0.58)	[0.212,3.262]
Economic shocks								

	At least mild anxiety				At least mild depression			
	Male		Female		Male		Female	
	Odds Ratio	95% CI	Odds Ratio	95% CI	Odds Ratio	95% CI	Odds Ratio	95% CI
Faced with new health expenses	0.670 (0.17)	[0.413,1.088]	1.165 (0.30)	[0.699,1.944]	1.037 (0.25)	[0.645,1.667]	1.171 (0.36)	[0.646,2.121]
Experienced adversity but did not reduce food consumption	0.627 (0.52)	[0.125,3.157]	0.691 (0.53)	[0.152,3.137]	1.449 (1.56)	[0.177,11.888]	0.313* (0.21)	[0.085,1.153]
Reduced food consumption as response to experienced adversity	1.181 (1.09)	[0.193,7.208]	0.882 (0.78)	[0.155,5.013]	1.943 (2.25)	[0.201,18.787]	0.481 (0.42)	[0.087,2.668]
Educational disruption								
Enrolled in/Planning to enrol in full time education and not participating in learning activities	0.934 (0.27)	[0.530,1.644]	1.134 (0.34)	[0.628,2.049]	0.914 (0.26)	[0.520,1.607]	1.456 (0.47)	[0.770,2.753]
Enrolled in/Planning to enrol in full time education and participating in learning activities	0.684 (0.24)	[0.342,1.369]	0.659 (0.23)	[0.328,1.324]	0.540* (0.19)	[0.276,1.057]	1.122 (0.42)	[0.540,2.332]
Background characteristics								
Age in months	1.007 (0.03)	[0.947,1.069]	0.971 (0.03)	[0.911,1.034]	1.013 (0.03)	[0.955,1.075]	0.968 (0.03)	[0.908,1.031]
Urban	0.570* (0.18)	[0.303,1.070]	2.637*** (0.74)	[1.517,4.585]	0.769 (0.22)	[0.439,1.349]	1.367 (0.45)	[0.720,2.594]
Participant has long-term health problem, 2016 (Round 5)	1.336 (0.46)	[0.676,2.640]	1.199 (0.46)	[0.568,2.530]	1.447 (0.49)	[0.748,2.802]	1.429 (0.53)	[0.692,2.951]
Total parent-child relationship score, 2012/2016 (Round 4/5)	0.918* (0.04)	[0.838,1.007]	0.922* (0.04)	[0.846,1.005]	0.921* (0.04)	[0.847,1.001]	0.864*** (0.04)	[0.787,0.948]
Total peer-child relationship score, 2016 (Round 5)	0.998 (0.05)	[0.908,1.096]	1.011 (0.05)	[0.910,1.123]	0.973 (0.05)	[0.877,1.080]	1.040 (0.06)	[0.934,1.157]
Middle/Top wealth tercile R5, 2016 (Round 5)	0.877 (0.23)	[0.524,1.468]	0.586* (0.17)	[0.333,1.033]	1.494 (0.41)	[0.877,2.546]	1.129 (0.34)	[0.627,2.033]
N	958		828		958		828	

*Note: Odds ratios are unadjusted odds ratios. Robust standard errors in parenthesis, *** significant at 1%, ** significant at 5%, * significant at 10%. Base categories are as follows: Believe they are at no/low risk, Did not leave the house at all during the past 7 days, Did not spend more time taking care of children, Did not spend more time on household chores, Did not spend more time working in the family business, Did not face new health expenses, Did not suffer a shock, Never attended school or not enrolled in full-time education/not planning to enrol, Rural, Does not have long-term health condition, Lowest wealth tercile. All time-variant variables are measured in 2020 unless otherwise specified.*

Supplementary Table 22. Logistic regression results: Symptoms of at least mild anxiety/depression by gender for the Younger Cohort only (Peru)

	At least mild anxiety				At least mild depression			
	Male		Female		Male		Female	
	Odds Ratio	95% CI	Odds Ratio	95% CI	Odds Ratio	95% CI	Odds Ratio	95% CI
COVID-19 related changes/behaviours								
Risk perception: believe they are at medium/high risk	1.375* (0.23)	[0.988,1.913]	1.488*** (0.23)	[1.100,2.014]	1.500** (0.26)	[1.064,2.114]	1.621*** (0.26)	[1.180,2.226]
Left house for any reason in the past 7 days	1.228 (0.27)	[0.804,1.876]	0.931 (0.17)	[0.650,1.332]	0.998 (0.22)	[0.645,1.546]	0.888 (0.17)	[0.614,1.284]
Difference in subjective well-being between Round 5 (2016) and phone survey (2020)	1.190*** (0.05)	[1.097,1.291]	1.060* (0.04)	[0.989,1.135]	1.137*** (0.05)	[1.036,1.247]	1.045 (0.04)	[0.970,1.126]
Change in responsibilities:								
Spend more time taking care of children	1.784*** (0.33)	[1.238,2.571]	1.384** (0.22)	[1.013,1.890]	1.823*** (0.35)	[1.247,2.665]	0.965 (0.16)	[0.699,1.331]
Spend more time on household chores	1.233 (0.25)	[0.832,1.826]	1.176 (0.25)	[0.771,1.792]	1.118 (0.23)	[0.748,1.671]	1.121 (0.24)	[0.731,1.720]
Spend more time working in the family business	1.094 (0.24)	[0.712,1.681]	1.426 (0.33)	[0.911,2.230]	1.038 (0.24)	[0.664,1.622]	1.238 (0.29)	[0.787,1.950]
Economic shocks								
Faced with new health expenses	2.093*** (0.38)	[1.460,3.000]	1.530*** (0.25)	[1.109,2.110]	2.089*** (0.40)	[1.435,3.041]	1.701*** (0.28)	[1.226,2.360]
Experienced adversity but did not reduce food consumption	4.608** (3.59)	[1.003,21.173]	1.475 (0.93)	[0.427,5.092]	3.693* (2.66)	[0.899,15.172]	1.615 (1.06)	[0.445,5.863]
Reduced food consumption as response to experienced adversity	3.800 (3.22)	[0.721,20.038]	1.249 (0.85)	[0.327,4.769]	4.074* (3.27)	[0.845,19.648]	2.556 (1.83)	[0.631,10.360]
Educational disruption								
Enrolled in/Planning to enrol in full time education and not participating in learning activities	0.559 (0.44)	[0.120,2.602]	0.552 (0.33)	[0.173,1.763]	1.190 (0.94)	[0.253,5.599]	0.407 (0.28)	[0.107,1.546]
Enrolled in/Planning to enrol in full time education and participating in learning activities	0.894 (0.16)	[0.625,1.278]	1.127 (0.18)	[0.825,1.538]	1.215 (0.23)	[0.841,1.754]	0.898 (0.15)	[0.646,1.248]
Background characteristics								
Age in months	0.981 (0.02)	[0.939,1.024]	1.004 (0.02)	[0.965,1.045]	0.953** (0.02)	[0.910,0.997]	0.991 (0.02)	[0.952,1.033]
Urban	1.090 (0.26)	[0.684,1.736]	1.658** (0.37)	[1.070,2.569]	1.043 (0.25)	[0.651,1.670]	1.268 (0.30)	[0.793,2.029]
Participant has long-term health problem, 2016 (Round 5)	2.008*** (0.54)	[1.186,3.398]	1.493* (0.33)	[0.964,2.312]	1.910** (0.53)	[1.108,3.293]	1.269 (0.28)	[0.827,1.947]
Total parent-child relationship score, 2012/2016 (Round 4/5)	0.973 (0.03)	[0.922,1.028]	0.977 (0.02)	[0.938,1.019]	0.907*** (0.03)	[0.856,0.961]	0.931*** (0.02)	[0.891,0.973]

	At least mild anxiety				At least mild depression			
	Male		Female		Male		Female	
	Odds Ratio	95% CI	Odds Ratio	95% CI	Odds Ratio	95% CI	Odds Ratio	95% CI
Total peer-child relationship score, 2016 (Round 5)	1.014 (0.03)	[0.954,1.078]	0.989 (0.02)	[0.947,1.034]	1.034 (0.03)	[0.968,1.104]	1.023 (0.02)	[0.975,1.072]
Middle/Top wealth tercile R5, 2016 (Round 5)	0.892 (0.18)	[0.600,1.325]	1.247 (0.24)	[0.857,1.816]	1.034 (0.21)	[0.695,1.537]	1.239 (0.26)	[0.821,1.871]
N	745		751		745		751	

*Note: Odds ratios are unadjusted odds ratios. Robust standard errors in parenthesis, *** significant at 1%, ** significant at 5%, * significant at 10%. Base categories are as follows: Believe they are at no/low risk, Did not leave the house at all during the past 7 days, Did not spend more time taking care of children, Did not spend more time on household chores, Did not spend more time working in the family business, Did not face new health expenses, Did not suffer a shock, Never attended school or not enrolled in full-time education/not planning to enrol, Rural, Does not have long-term health condition, Lowest wealth tercile. All time-variant variables are measured in 2020 unless otherwise specified.*

Supplementary Table 23. Logistic regression results: Symptoms of at least mild anxiety/depression by gender for the Younger Cohort only (Vietnam)

	At least mild anxiety				At least mild depression			
	Male		Female		Male		Female	
	Odds Ratio	95% CI	Odds Ratio	95% CI	Odds Ratio	95% CI	Odds Ratio	95% CI
COVID-19 related changes/behaviours								
Risk perception: believe they are at medium/high risk	1.553 (0.49)	[0.834,2.890]	0.830 (0.25)	[0.464,1.482]	1.711 (0.61)	[0.851,3.439]	0.775 (0.22)	[0.446,1.347]
Left house for any reason in the past 7 days	0.862 (0.33)	[0.409,1.814]	1.783 (0.64)	[0.879,3.617]	0.944 (0.37)	[0.441,2.022]	1.480 (0.47)	[0.793,2.759]
Difference in subjective well-being between Round 5 (2016) and phone survey (2020)	1.247*** (0.10)	[1.063,1.462]	1.154** (0.07)	[1.021,1.305]	1.444*** (0.12)	[1.221,1.708]	1.008 (0.06)	[0.896,1.133]
Change in responsibilities:								
Spend more time taking care of children	1.005 (0.41)	[0.452,2.238]	1.720** (0.46)	[1.019,2.903]	1.425 (0.55)	[0.667,3.045]	1.796** (0.44)	[1.111,2.902]
Spend more time on household chores	0.948 (0.25)	[0.570,1.575]	1.057 (0.26)	[0.654,1.706]	1.079 (0.31)	[0.619,1.880]	2.902*** (0.80)	[1.694,4.972]
Spend more time working in the family business	2.053** (0.73)	[1.018,4.140]	1.509 (0.46)	[0.830,2.745]	2.214** (0.79)	[1.099,4.459]	1.750* (0.50)	[0.998,3.068]
Economic shocks								
Faced with new health expenses	0.930 (0.50)	[0.323,2.684]	1.523 (0.62)	[0.688,3.368]	1.294 (0.63)	[0.498,3.365]	0.476 (0.24)	[0.181,1.255]

	At least mild anxiety				At least mild depression			
	Male		Female		Male		Female	
	Odds Ratio	95% CI	Odds Ratio	95% CI	Odds Ratio	95% CI	Odds Ratio	95% CI
Experienced adversity but did not reduce food consumption	2.415*** (0.77)	[1.295,4.502]	1.091 (0.29)	[0.652,1.826]	2.411*** (0.81)	[1.245,4.668]	1.502 (0.40)	[0.888,2.542]
Reduced food consumption as response to experienced adversity	2.963*** (1.22)	[1.320,6.652]	1.166 (0.37)	[0.628,2.165]	2.427** (1.04)	[1.050,5.613]	2.449*** (0.74)	[1.350,4.441]
Educational disruption								
Enrolled in/Planning to enrol in full time education and not participating in learning activities	0.954 (0.44)	[0.390,2.333]	0.817 (0.34)	[0.358,1.862]	1.153 (0.58)	[0.431,3.085]	0.967 (0.35)	[0.476,1.966]
Enrolled in/Planning to enrol in full time education and participating in learning activities	0.678 (0.23)	[0.350,1.315]	0.714 (0.19)	[0.420,1.216]	1.250 (0.43)	[0.639,2.445]	1.058 (0.29)	[0.622,1.799]
Background characteristics								
Age in months	1.009 (0.04)	[0.933,1.091]	1.027 (0.04)	[0.954,1.106]	1.051 (0.04)	[0.976,1.131]	1.014 (0.04)	[0.938,1.095]
Urban	1.026 (0.30)	[0.576,1.826]	1.550* (0.41)	[0.921,2.609]	0.735 (0.24)	[0.386,1.399]	1.562* (0.40)	[0.942,2.590]
Participant has long-term health problem, 2016 (Round 5)	1.190 (0.58)	[0.458,3.088]	1.036 (0.37)	[0.517,2.074]	2.696** (1.17)	[1.151,6.317]	1.204 (0.40)	[0.630,2.300]
Total parent-child relationship score, 2012/2016 (Round 4/5)	1.004 (0.05)	[0.918,1.098]	1.055 (0.04)	[0.981,1.135]	0.925 (0.05)	[0.833,1.026]	0.999 (0.04)	[0.931,1.072]
Total peer-child relationship score, 2016 (Round 5)	1.058 (0.06)	[0.950,1.178]	0.916 (0.05)	[0.821,1.022]	1.063 (0.07)	[0.926,1.220]	0.948 (0.05)	[0.852,1.054]
Middle/Top wealth tercile R5, 2016 (Round 5)	0.583* (0.18)	[0.323,1.055]	0.741 (0.18)	[0.454,1.208]	1.314 (0.45)	[0.672,2.566]	1.164 (0.31)	[0.693,1.955]
N	790		806		790		806	

*Note: Odds ratios are unadjusted odds ratios. Robust standard errors in parenthesis, *** significant at 1%, ** significant at 5%, * significant at 10%. Base categories are as follows: Believe they are at no/low risk, Did not leave the house at all during the past 7 days, Did not spend more time taking care of children, Did not spend more time on household chores, Did not spend more time working in the family business, Did not face new health expenses, Did not suffer a shock, Never attended school or not enrolled in full-time education/not planning to enrol, Rural, Does not have long-term health condition, Lowest wealth tercile. All time-variant variables are measured in 2020 unless otherwise specified.*

Supplementary Table 24. Ethiopia: Mean score and rates of general anxiety disorder

	Mean GAD-7 score	CI95%	p-value	Minimal (0-4)	CI95%	p-value	Mild anxiety (5-9)	CI95%	p-value	Moderate anxiety (10-14)	CI95%	p-value	Severe anxiety (>=15)	CI95%	p-value
**Total**	**2.06**	**1.94; 2.19**		**82.13**	**80.46; 83.72**		**15.21**	**13.73; 16.78**		**2.34**	**1.74; 3.06**		**0.32**	**0.13; 0.66**	
Male	2.03	1.86; 2.20	0.603	82.52	80.35; 84.70	0.610	14.66	12.64; 16.69	0.445	2.47	1.58; 3.36	0.650	0.34	0.01; 0.67	0.856
Female	2.10	1.92; 2.28		81.68	79.30; 84.07		15.84	13.59; 18.10		2.18	1.28; 3.08		0.30	-0.04; 0.63	
Rural	1.78	1.62; 1.93	0.000	85.33	83.30; 87.35	0.000	12.55	10.66; 14.45	0.000	2.12	1.30; 2.94	0.470	0.00	0.00; 0.00	0.004
Urban	2.40	2.20; 2.60		78.39	75.84; 80.94		18.33	15.93; 20.72		2.59	1.61; 3.57		0.70	0.18; 1.21	
Poorest tercile	1.89	1.68; 2.10	0.056	85.10	82.49; 87.71	0.011	11.98	9.60; 14.36	0.003	2.65	1.47; 3.82	0.502	0.28	-0.11; 0.66	0.808
Middle/Richest terciles	2.15	1.99; 2.30		80.68	78.66; 82.71		16.79	14.88; 18.71		2.18	1.44; 2.93		0.34	0.04; 0.64	
No internet	1.90	1.74; 2.07	0.010	84.03	81.89; 86.18	0.017	13.20	11.22; 15.19	0.007	2.50	1.58; 3.41	0.608	0.27	-0.04; 0.57	0.653
With internet	2.23	2.05; 2.41		80.13	77.73; 82.53		17.33	15.05; 19.60		2.17	1.29; 3.04		0.38	0.01; 0.75	
N	2183														

Notes: If any missing answers to questions then the whole score is set to missing. p-values represent significance of t-test of equality between groups (Male-Female; Rural-Urban; Bottom-Top/Middle wealth tercile; Internet access through home computer/working smartphone (No-Yes)). Poorest and middle/richest terciles refer to the household's position in the 2016 (round 5) wealth distribution.
Results are for the combined Younger Cohort/Older Cohort sample.

Supplementary Table 25. Ethiopia: Mean score and rates of depression

	Mean PHQ-8 score	CI95%	p-val	PHQ (0-4)	CI95%	p-val	PHQ (5-9)	CI95%	p-val	PHQ (10-14)	CI95%	p-val	PHQ (15-19)	CI95%	p-val	PHQ (>=20)	CI95%	p-val
**Total**	**1.89**	**1.78; 2.01**		**84.56**	**82.98; 86.05**		**13.33**	**11.93; 14.83**		**1.97**	**1.43; 2.64**		**0.09**	**0.01; 0.33**		**0.05**	**0.00; 0.25**	
Male	1.86	1.70; 2.02	0.569	85.08	83.04; 87.12	0.470	12.79	10.87; 14.70	0.422	1.88	1.10; 2.65	0.733	0.17	-0.07; 0.41	0.189	0.09	-0.08; 0.25	0.354
Female	1.93	1.76; 2.10		83.96	81.69; 86.23		13.96	11.82; 16.10		2.08	1.20; 2.96		0.00	0.00; 0.00		0.00	0.00; 0.00	
Rural	1.66	1.52; 1.81	0.000	87.11	85.19; 89.02	0.000	11.54	9.71; 13.36	0.007	1.27	0.63; 1.91	0.011	0.08	-0.08; 0.25	0.909	0.00	0.00; 0.00	0.279
Urban	2.16	1.97; 2.34		81.57	79.17; 83.97		15.44	13.20; 17.68		2.79	1.77; 3.81		0.10	-0.10; 0.29		0.10	-0.10; 0.29	
Poorest tercile	1.75	1.55; 1.95	0.086	85.65	83.09; 88.22	0.323	12.53	10.11; 14.96	0.444	1.53	0.63; 2.43	0.303	0.28	-0.11; 0.66	0.043	0.00	0.00; 0.00	0.484
Middle/Richest terciles	1.96	1.82; 2.10		84.03	82.15; 85.90		13.72	11.96; 15.48		2.18	1.44; 2.93		0.00	0.00; 0.00		0.07	-0.07; 0.20	
No internet	1.69	1.54; 1.85	0.001	85.91	83.87; 87.94	0.074	12.49	10.55; 14.43	0.235	1.52	0.80; 2.23	0.118	0.09	-0.09; 0.26	0.970	0.00	0.00; 0.00	0.304
With internet	2.10	1.93; 2.28		83.15	80.89; 85.40		14.22	12.12; 16.32		2.45	1.52; 3.38		0.09	-0.09; 0.28		0.09	-0.09; 0.28	
N		2183																

Notes: If any missing answers to questions then the whole score is set to missing. p-values represent significance of t-test of equality between groups (Male-Female; Rural-Urban; Bottom-Top/Middle wealth tercile; Internet access through home computer/working smartphone (No-Yes)). Poorest and middle/richest terciles refer to the household's position in the 2016 (round 5) wealth distribution.
Results are for the combined Younger Cohort/ Older Cohort sample.

Supplementary Table 26.India: Mean score and rates of general anxiety disorder

	Mean GAD-7 score	CI95%	p-value	Minimal (0-4)	CI95%	p-value	Mild anxiety (5-9)	CI95%	p-value	Moderate anxiety (10-14)	CI95%	p-value	Severe anxiety (>=15)	CI95%	p-value
**Total**	**1.69**	**1.59; 1.78**		**88.94**	**87.68; 90.12**		**9.15**	**8.08; 10.32**		**1.64**	**1.19; 2.20**		**0.27**	**0.11; 0.55**	
Male	1.55	1.43; 1.67	0.003	90.72	89.19; 92.26	0.002	8.04	6.59; 9.48	0.038	1.10	0.54; 1.65	0.022	0.15	-0.06; 0.35	0.210
Female	1.84	1.69; 1.99		86.99	85.13; 88.86		10.38	8.69; 12.06		2.23	1.42; 3.05		0.40	0.05; 0.75	
Rural	1.76	1.65; 1.88	0.014	88.19	86.73; 89.66	0.055	9.56	8.23; 10.90	0.252	1.92	1.30; 2.55	0.071	0.32	0.06; 0.58	0.401
Urban	1.50	1.34; 1.66		90.80	88.73; 92.87		8.13	6.17; 10.09		0.93	0.24; 1.62		0.13	-0.13; 0.39	
Poorest tercile	1.83	1.66; 2.00	0.042	86.48	84.22; 88.74	0.004	11.02	8.95; 13.09	0.018	2.16	1.20; 3.12	0.137	0.34	-0.04; 0.73	0.602
Middle/Richest terciles	1.62	1.50; 1.73		90.18	88.79; 91.58		8.21	6.92; 9.50		1.38	0.83; 1.93		0.23	0.00; 0.45	
No internet	1.80	1.52; 2.08	0.410	87.84	84.11; 91.57	0.521	10.47	6.98; 13.97	0.403	1.69	0.22; 3.16	0.944	0.00	0.00; 0.00	0.345
With internet	1.67	1.57; 1.78		89.08	87.81; 90.35		8.99	7.82; 10.15		1.63	1.12; 2.15		0.30	0.08; 0.52	
N	2622														

Notes: If any missing answers to questions then the whole score is set to missing. p-values represent significance of t-test of equality between groups (Male-Female; Rural-Urban; Bottom-Top/Middle wealth tercile; Internet access through home computer/working smartphone (No-Yes)). Poorest and middle/richest terciles refer to the household's position in the 2016 (round 5) wealth distribution.
Results are for the combined Younger Cohort/ Older Cohort sample.

Supplementary Table 27. India: Mean score and rates of depression

	Mean PHQ-8 score	CI95%	p-value	No significant depressive symptoms(0-4)	CI95%	p-value	Mild depression (5-9)	CI95%	p-value	Moderate depressive symptoms(10-14)	CI95%	p-value	Moderately severe depressive symptoms(15-19)	CI95%	p-value
**Total**	**1.41**	**1.33; 1.5**		**90.08**	**88.88; 91.2**		**8.73**	**7.68; 9.88**		**1.03**	**0.68; 1.49**		**0.15**	**0.04; 0.39**	
Male	1.34	1.22; 1.45	0.067	90.36	88.79; 91.92	0.624	8.84	7.33; 10.34	0.843	0.73	0.28; 1.18	0.113	0.07	-0.07; 0.22	0.276
Female	1.50	1.37; 1.63		89.78	88.11; 91.46		8.62	7.06; 10.17		1.36	0.72; 2.00		0.24	-0.03; 0.51	
Rural	1.47	1.36; 1.58	0.039	89.16	87.75; 90.57	0.012	9.40	8.08; 10.72	0.056	1.28	0.77; 1.79	0.043	0.16	-0.02; 0.34	0.873
Urban	1.27	1.12; 1.42		92.40	90.50; 94.30		7.07	5.23; 8.90		0.40	-0.05; 0.85		0.13	-0.13; 0.39	
Poorest tercile	1.49	1.33; 1.65	0.220	89.20	87.15; 91.26	0.284	9.55	7.60; 11.49	0.296	1.02	0.36; 1.69	0.980	0.23	-0.09; 0.54	0.486
Middle/Richest terciles	1.37	1.27; 1.48		90.53	89.15; 91.90		8.32	7.03; 9.620		1.03	0.56; 1.51		0.11	-0.04; 0.27	
No internet	1.42	1.17; 1.67	0.941	89.86	86.42; 93.31	0.894	8.78	5.55; 12.02	0.974	1.35	0.03; 2.67	0.561	0.00	0.00; 0.00	0.475
With internet	1.41	1.32; 1.50		90.11	88.90; 91.33		8.73	7.58; 9.88		0.99	0.59; 1.39		0.17	0.00; 0.34	
N	2622														

Notes: If any missing answers to questions then the whole score is set to missing. p-values represent significance of t-test of equality between groups (Male-Female; Rural-Urban; Bottom-Top/Middle wealth tercile; Internet access through home computer/working smartphone (No-Yes)). Poorest and middle/richest terciles refer to the household's position in the 2016 (round 5) wealth distribution.
Results are for the combined Younger Cohort/ Older Cohort sample. No severe depressive symptoms were reported.

Supplementary Table 28. Peru: Mean score and rates of general anxiety disorder

	Mean GAD-7 score	CI95%	p-value	Minimal (0-4)	CI95%	p-value	Mild anxiety (5-9)	CI95%	p-value	Moderate anxiety (10-14)	CI95%	p-value	Severe anxiety (>=15)	CI95%	p-value
**Total**	**4.53**	**4.34; 4.72**		**59.14**	**56.88; 61.37**		**27.34**	**25.34; 29.42**		**10.55**	**9.20; 12.02**		**2.97**	**2.25; 3.84**	
Male	3.80	3.55; 4.04	0.000	66.35	63.36; 69.35	0.000	23.72	21.02; 26.42	0.000	7.94	6.23; 9.66	0.000	1.99	1.10; 2.87	0.011
Female	5.29	5.01; 5.57		51.72	48.51; 54.94		31.08	28.10; 34.05		13.23	11.05; 15.41		3.98	2.72; 5.24	
Rural	3.87	3.46; 4.27	0.001	66.29	61.37; 71.21	0.002	25.56	21.02; 30.10	0.402	5.62	3.22; 8.01	0.001	2.53	0.89; 4.16	0.588
Urban	4.69	4.47; 4.90		57.48	55.00; 59.96		27.76	25.51; 30.00		11.69	10.08; 13.30		3.07	2.20; 3.93	
Poorest tercile	4.05	3.74; 4.35	0.001	63.08	59.16; 66.99	0.020	27.01	23.41; 30.61	0.826	8.21	5.98; 10.43	0.026	1.71	0.66; 2.76	0.031
Middle/Richest terciles	4.75	4.51; 4.99		57.37	54.68; 60.06		27.50	25.07; 29.92		11.60	9.86; 13.34		3.53	2.53; 4.54	
No internet	4.96	4.14; 5.78	0.367	48.68	37.37; 60.00	0.058	36.84	25.92; 47.77	0.058	14.47	6.51; 22.44	0.255	0.00	0.00; 0.00	0.120
With internet	4.51	4.32; 4.71		59.58	57.32; 61.84		26.95	24.90; 28.99		10.38	8.97; 11.79		3.09	2.29; 3.89	
N	1887														

Notes: If any missing answers to questions then the whole score is set to missing. p-values represent significance of t-test of equality between groups (Male-Female; Rural-Urban; Bottom-Top/Middle wealth tercile; Internet access through home computer/working smartphone (No-Yes)). Poorest and middle/richest terciles refer to the household's position in the 2016 (round 5) wealth distribution.
Results are for the combined Younger Cohort/ Older Cohort sample.

Supplementary Table 29. Peru: Mean score and rates of depression

	Mean PHQ-8 score	CI 95%	p-val	PHQ (0-4)	CI 95%	p-val	PHQ (5-9)	CI 95%	p-val	PHQ (10-14)	CI 95%	p-val	PHQ (15-19)	CI 95%	p-val	PHQ (>=20)	CI 95%	p-val
**Total**	**3.61**	**3.43; 3.79**		**68.42**	**66.26; 70.51**		**21.94**	**20.09; 23.88**		**7.79**	**6.62; 9.09**		**1.48**	**0.99; 2.14**		**0.37**	**0.15; 0.76**	
Male	3.08	2.85; 3.32	0.000	72.62	69.79; 75.45	0.000	19.75	17.22; 22.27	0.020	6.58	5.01; 8.16	0.047	0.73	0.19; 1.27	0.006	0.31	-0.04; 0.67	0.677
Female	4.15	3.89; 4.41		64.09	61.00; 67.17		24.19	21.44; 26.95		9.03	7.19; 10.88		2.26	1.30; 3.21		0.43	0.01; 0.85	
Rural	3.11	2.73; 3.49	0.008	73.60	69.01; 78.18	0.020	19.38	15.27; 23.50	0.196	6.18	3.67; 8.69	0.208	0.84	-0.11; 1.79	0.267	0.00	0.00; 0.00	0.201
Urban	3.73	3.53; 3.92		67.21	64.86; 69.56		22.53	20.44; 24.63		8.16	6.79; 9.54		1.63	1.00; 2.27		0.46	0.12; 0.80	
Poorest tercile	3.07	2.79; 3.36	0.000	73.85	70.28; 77.41	0.001	19.66	16.43; 22.88	0.109	5.47	3.62; 7.32	0.012	0.68	0.01; 1.35	0.054	0.34	-0.13; 0.82	0.889
Middle/ Richest terciles	3.85	3.63; 4.07		65.98	63.40; 68.55		22.96	20.68; 25.25		8.83	7.29; 10.38		1.84	1.11; 2.57		0.38	0.05; 0.72	
No internet	3.34	2.62; 4.07	0.545	68.42	57.89; 78.95	0.999	25.00	15.19; 34.81	0.511	6.58	0.96; 12.19	0.688	0.00	0.00; 0.00	0.275	0.00	0.00; 0.00	0.587
With internet	3.62	3.44; 3.80		68.42	66.27; 70.56		21.81	19.91; 23.71		7.84	6.60; 9.08		1.55	0.98; 2.11		0.39	0.10; 0.67	
N	1887																	

Notes: If any missing answers to questions then the whole score is set to missing. p-values represent significance of t-test of equality between groups (Male-Female; Rural-Urban; Bottom-Top/Middle wealth tercile; Internet access through home computer/working smartphone (No-Yes)). Poorest and middle/richest terciles refer to the household's position in the 2016 (round 5) wealth distribution.
Results are for the combined Younger Cohort/ Older Cohort sample.

Supplementary Table 30. Vietnam: Mean score and rates of general anxiety disorder

	Mean GAD-7 score	CI95%	p-value	Minimal (0-4)	CI95%	p-value	Mild anxiety (5-9)	CI95%	p-value	Moderate anxiety (10-14)	CI95%	p-value	Severe anxiety (>=15)	CI95%	p-value
**Total**	**1.47**	**1.37; 1.56**		**90.68**	**89.42; 91.84**		**7.93**	**6.85; 9.11**		**1.09**	**0.71; 1.60**		**0.30**	**0.12; 0.63**	
Male	1.31	1.17; 1.44	0.001	92.08	90.49; 93.67	0.025	6.48	5.03; 7.93	0.013	1.17	0.54; 1.80	0.717	0.27	-0.04; 0.58	0.769
Female	1.62	1.48; 1.76		89.37	87.61; 91.12		9.28	7.63; 10.94		1.01	0.44; 1.58		0.34	0.01; 0.67	
Rural	1.47	1.34; 1.60	0.925	90.93	89.33; 92.53	0.652	7.62	6.15; 9.10	0.559	1.04	0.48; 1.61	0.819	0.40	0.05; 0.75	0.362
Urban	1.46	1.32; 1.60		90.38	88.60; 92.17		8.29	6.62; 9.95		1.14	0.50; 1.79		0.19	-0.07; 0.45	
Poorest tercile	1.60	1.42; 1.78	0.045	88.33	86.08; 90.59	0.006	10.00	7.89; 12.11	0.008	1.15	0.40; 1.90	0.830	0.51	0.01; 1.01	0.195
Middle/Richest terciles	1.40	1.29; 1.51		91.89	90.51; 93.26		6.86	5.59; 8.13		1.06	0.54; 1.57		0.20	-0.03; 0.42	
No internet	2.40	1.24; 3.56	0.017	77.14	63.02; 91.26	0.005	17.14	4.47; 29.82	0.042	2.86	-2.75; 8.46	0.310	2.86	-2.75; 8.46	0.006
With internet	1.45	1.36; 1.55		90.89	89.70; 92.08		7.78	6.68; 8.89		1.06	0.64; 1.48		0.27	0.05; 0.48	
N	2296														

Notes: If any missing answers to questions then the whole score is set to missing. p-values represent significance of t-test of equality between groups (Male-Female; Rural-Urban; Bottom-Top/Middle wealth tercile; Internet access through home computer/working smartphone (No-Yes)). Poorest and middle/richest terciles refer to the household's position in the 2016 (round 5) wealth distribution.
Results are for the combined Younger Cohort/ Older Cohort sample.

Supplementary Table 31. Vietnam: Mean score and rates of depression

	Mean PHQ-8 score	CI95%	p-value	No significant depressive symptoms(0-4)	CI95%	p-value	Mild depression (5-9)	CI95%	p-value	Moderate depr-essive symptoms (10-14)	CI95%	p-value	Moderately severe depressive symptoms(15-19)	CI95%	p-value
**Total**	**1.42**	**1.32; 1.52**		**90.51**	**89.23; 91.67**		**7.67**	**6.61; 8.83**		**1.39**	**0.96; 1.96**		**0.44**	**0.21; 0.80**	
Male	1.24	1.11; 1.37	0.000	92.35	90.78; 93.91	0.004	6.12	4.71; 7.53	0.007	1.17	0.54; 1.80	0.376	0.36	0.01; 0.71	0.595
Female	1.59	1.45; 1.74		88.78	86.98; 90.58		9.11	7.47; 10.75		1.60	0.89; 2.32		0.51	0.10; 0.91	
Rural	1.43	1.30; 1.56	0.852	90.61	88.99; 92.23	0.852	7.70	6.22; 9.19	0.939	1.20	0.6; 1.81	0.398	0.48	0.10; 0.87	0.716
Urban	1.41	1.26; 1.56		90.38	88.60; 92.17		7.62	6.01; 9.23		1.62	0.85; 2.38		0.38	0.01; 0.75	
Poorest tercile	1.40	1.23; 1.57	0.757	91.54	89.58; 93.49	0.226	6.28	4.58; 7.99	0.074	1.54	0.67; 2.40	0.671	0.64	0.08; 1.20	0.284
Middle/Richest terciles	1.43	1.31; 1.55		89.97	88.46; 91.49		8.38	6.98; 9.77		1.32	0.74; 1.89		0.33	0.04; 0.62	
No internet	2.20	1.05; 3.35	0.053	85.71	73.95; 97.48	0.330	8.57	-0.84; 17.99	0.839	2.86	-2.75; 8.46	0.457	2.86	-2.75; 8.46	0.028
With internet	1.41	1.31; 1.51		90.58	89.37; 91.78		7.65	6.55; 8.75		1.37	0.89; 1.85		0.40	0.14; 0.66	
N	2296														

Notes: If any missing answers to questions then the whole score is set to missing. p-values represent significance of t-test of equality between groups (Male-Female; Rural-Urban; Bottom-Top/Middle wealth tercile; Internet access through home computer/working smartphone (No-Yes)). Poorest and middle/richest terciles refer to the household's position in the 2016 (round 5) wealth distribution.
Results are for the combined Younger Cohort/ Older Cohort sample. No severe depressive symptoms were reported.

Supplementary Table 32. Correlation between GAD-7 and PHQ-8 scores

	Ethiopia	India	Peru	Vietnam
Correlation coefficient	0.629***	0.610***	0.700***	0.636***

*Note: Bonferroni corrected p-value *** significant at 1%, ** significant at 5%, * significant at 10% for the unconditional correlation between GAD-7 and PHQ-8. Results are for the combined Younger Cohort/ Older Cohort sample.*

Supplementary Table 33. Joint rates

	Ethiopia		India		Peru		Vietnam	
	% of sample	95% CI	% of sample	95% CI	% of sample	95% CI	% of sample	95% CI
Display neither symptoms of anxiety nor depression	75.45	73.58; 77.24	83.87	82.40; 85.26	52.36	50.08; 54.63	85.50	83.99; 86.91
Display symptoms of at least mild anxiety only	9.12	7.94; 10.4	6.22	5.32; 7.21	16.06	14.43; 17.79	5.01	4.15; 5.98
Display symptoms of at least mild depression only	6.69	7.94; 10.4	5.07	5.32; 7.21	6.78	14.43; 17.79	5.18	4.15; 5.98
Display symptoms of at least mild anxiety and at least mild depression	8.75	7.60; 10.01	4.84	4.05; 5.74	24.80	22.87; 26.81	4.31	3.52; 5.22

Note: Results are for the combined Younger Cohort/ Older Cohort sample.

Supplementary Table 34. Correlation between subjective well-being and GAD-7/PHQ-8 scores

	Ethiopia				India				Peru				Vietnam			
	Subjective well-being (phone survey, 2020)		Subjective well-being (difference Round 5 (2016) and phone survey (2020))		Subjective well-being (phone survey, 2020)		Subjective well-being (difference Round 5 (2016) and phone survey (2020))		Subjective well-being (phone survey, 2020)		Subjective well-being (difference Round 5 (2016) and phone survey (2020))		Subjective well-being (phone survey, 2020)		Subjective well-being (difference Round 5 (2016) and phone survey (2020))	
	r	p-value	r	p-value	r	p-value	r	p-value	r	p-value	r	p-value	r	p-value	r	p-value
Mean GAD-7 score	0.140	0.000	0.041	0.054	0.107	0.000	0.064	0.001	0.199	0.000	0.096	0.000	0.253	0.000	0.118	0.000
Mean PHQ-8 score	0.091	0.000	0.024	0.263	0.097	0.000	0.055	0.005	0.186	0.000	0.067	0.004	0.207	0.000	0.119	0.000

Note: Results are for the combined Younger Cohort/ Older Cohort sample. Bonferroni corrected p-value.

Supplementary Table 35. Subjective well-being score Round 5 (2016) / phone survey (2020) by anxiety/depression (Ethiopia & India)

	Ethiopia							India						
	**All**	**at least mild anxiety**	**no anxiety**	**p-value**	**at least mild depression**	**no depression**	**p-value**	**All**	**at least mild anxiety**	**no anxiety**	**p-value**	**at least mild depression**	**no depression**	**p-value**
(1) Subjective well-being, 2016 (Round 5)	5.69	5.43	5.75	0.000	5.61	5.71	0.303	5.05	4.94	5.06	0.178	4.98	5.06	0.381
(2) Subjective well-being, 2020 (Phone survey)	4.73	4.28	4.83	0.000	4.36	4.80	0.000	4.56	4.18	4.61	0.000	4.12	4.61	0.000
Difference in subjective well-being (1)-(2)	0.96	1.15	0.92	0.052	1.26	0.91	0.005	0.49	0.77	0.45	0.009	0.86	0.45	0.001

Note: Results are for the combined Younger Cohort/ Older Cohort sample.

Supplementary Table 36. Subjective well-being score Round 5 (2016) / phone survey (2020) by anxiety/depression (Peru & Vietnam)

	Peru							Vietnam						
	All	at least mild anxiety	no anxiety	p-value	at least mild depression	no depression	p-value	All	at least mild anxiety	no anxiety	p-value	at least mild depression	no depression	p-value
(1) Subjective well-being, 2016 (Round 5)	6.33	6.20	6.42	0.001	6.14	6.42	0.000	5.87	5.46	5.91	0.000	5.65	5.89	0.024
(2) Subjective well-being, 2020 (Phone survey)	5.81	5.47	6.05	0.000	5.44	5.98	0.000	6.26	5.33	6.36	0.000	5.54	6.34	0.000
Difference in subjective well-being (1)-(2)	0.52	0.74	0.38	0.000	0.70	0.44	0.013	-0.39	0.14	-0.45	0.000	0.11	-0.45	0.000

Note: Results are for the combined Younger Cohort/ Older Cohort sample.
