# Supplementary material for: Impact of the COVID-19 pandemic on anxiety and depression symptoms of young people in the global south: evidence from a four-country cohort study
Source: BMJ Open. 2021 Apr 15;11(4):e049653. doi: 10.1136/bmjopen-2021-049653 (PMC8053815; doi:10.1136/bmjopen-2021-049653)
Supplement: Supplementary data [file bmjopen-2021-049653supp003.pdf]

Supplementary figures

Supplement to: Impact of the COVID-19 Pandemic on anxiety and depression symptoms of young people in the Global South: evidence from a four-country cohort study

- Supplementary Figure 1

Supplementary Figure 1. Variables used in the analysis

Variables used in the analysis. Gender refers to male/female. Location refers to rural/urban.
