# Supplementary figures and images for: Impact of the COVID-19 pandemic on anxiety and depression symptoms of young people in the global south: evidence from a four-country cohort study
Source: BMJ Open. 2021 Apr 15;11(4):e049653. doi: 10.1136/bmjopen-2021-049653 (PMC8053815; doi:10.1136/bmjopen-2021-049653)

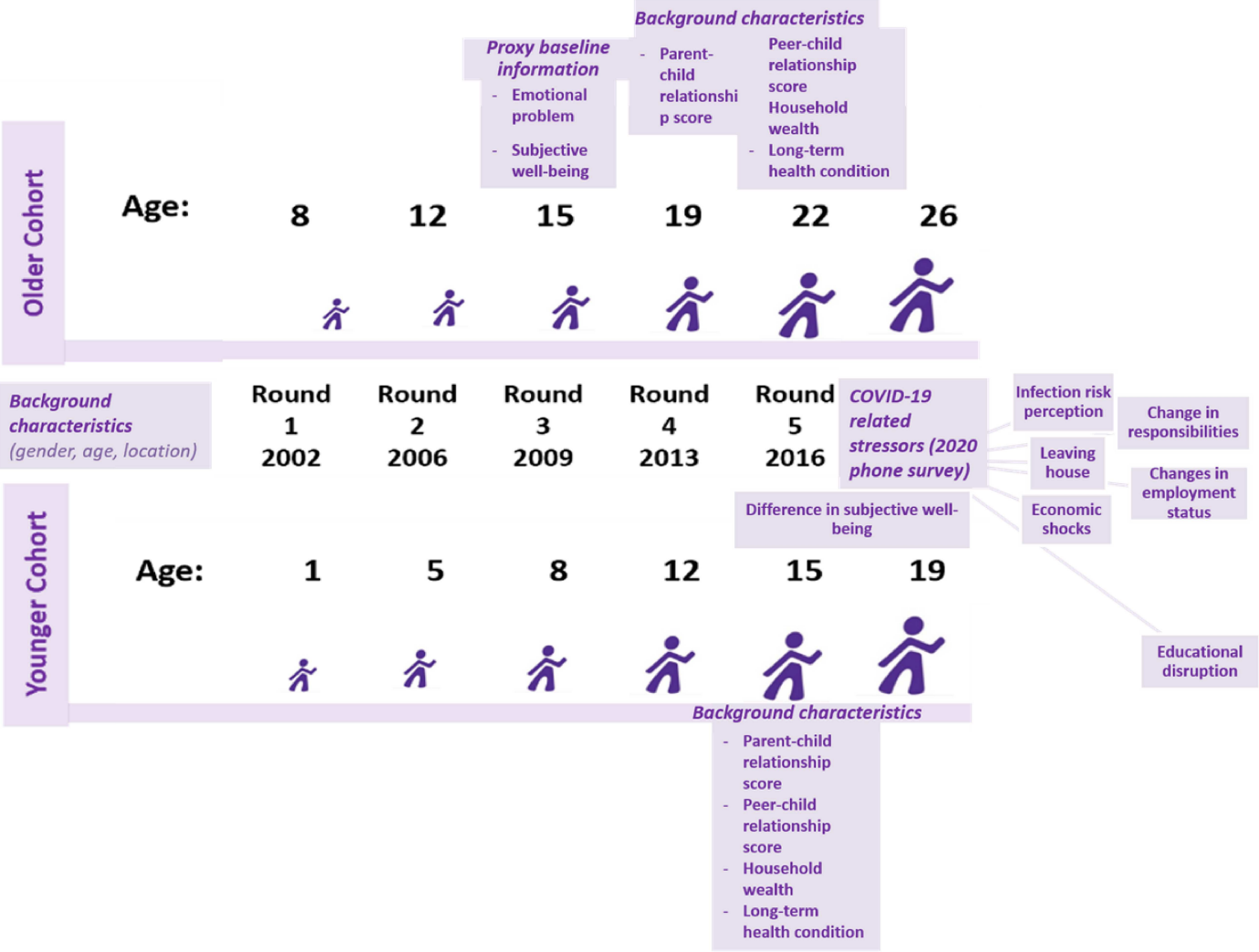

Supplement: Supplementary data [file bmjopen-2021-049653supp004.pdf]
