# Supplementary material for: Impact of the COVID-19 pandemic on anxiety and depression symptoms of young people in the global south: evidence from a four-country cohort study
Source: BMJ Open. 2021 Apr 15;11(4):e049653. doi: 10.1136/bmjopen-2021-049653 (PMC8053815; doi:10.1136/bmjopen-2021-049653)
Supplement: Author's manuscript [file bmjopen-2021-049653.draft_revisions.pdf]

BMJ Open is committed to open peer review. As part of this commitment we make the peer review history of every article we publish publicly available.

When an article is published we post the peer reviewers' comments and the authors' responses online. We also post the versions of the paper that were used during peer review. These are the versions that the peer review comments apply to.

The versions of the paper that follow are the versions that were submitted during the peer review process. They are not the versions of record or the final published versions. They should not be cited or distributed as the published version of this manuscript.

BMJ Open is an open access journal and the full, final, typeset and author-corrected version of record of the manuscript is available on our site with no access controls, subscription charges or pay-per-view fees (http://bmjopen.bmj.com).

If you have any questions on BMJ Open's open peer review process please email info.bmjopen@bmj.com

BMJ Open

Impact of the COVID-19 Pandemic on anxiety and depression symptoms of young people in the Global South: evidence from a four-country cohort study

Journal:	*BMJ Open*
Manuscript ID	bmjopen-2021-049653
Article Type:	Original research
Date Submitted by the Author:	01-Feb-2021
Complete List of Authors:	Porter, Catherine; Lancaster University, Management School<br>Favara, Marta; University of Oxford, Oxford Department of International Development<br>Hittmeyer, Annina; University of Oxford Department of International Development<br>Scott, Douglas; University of Oxford, Oxford Department of International Development<br>Sánchez Jiménez, Alan<br>Ellanki, Revathi; Centre for Economic and Social Studies<br>Woldehanna, T; Addis Ababa University, Department of Economics<br>Duc, Le Thuc; Vietnam Academy of Social Sciences<br>Craske, Michelle; University of California Los Angeles, UCLA Department of Psychology<br>Stein, Alan; University of Oxford; University of the Witwatersrand
Keywords:	COVID-19, Anxiety disorders < PSYCHIATRY, Depression & mood disorders < PSYCHIATRY

SCHOLARONE™
Manuscripts

I, the Submitting Author has the right to grant and does grant on behalf of all authors of the Work (as defined in the below author licence), an exclusive licence and/or a non-exclusive licence for contributions from authors who are: i) UK Crown employees; ii) where BMJ has agreed a CC-BY licence shall apply, and/or iii) in accordance with the terms applicable for US Federal Government officers or employees acting as part of their official duties; on a worldwide, perpetual, irrevocable, royalty-free basis to BMJ Publishing Group Ltd ("BMJ") its licensees and where the relevant Journal is co-owned by BMJ to the co-owners of the Journal, to publish the Work in this journal and any other BMJ products and to exploit all rights, as set out in our licence.

The Submitting Author accepts and understands that any supply made under these terms is made by BMJ to the Submitting Author unless you are acting as an employee on behalf of your employer or a postgraduate student of an affiliated institution which is paying any applicable article publishing charge ("APC") for Open Access articles. Where the Submitting Author wishes to make the Work available on an Open Access basis (and intends to pay the relevant APC), the terms of reuse of such Open Access shall be governed by a Creative Commons licence – details of these licences and which Creative Commons licence will apply to this Work are set out in our licence referred to above.

Other than as permitted in any relevant BMJ Author's Self Archiving Policies, I confirm this Work has not been accepted for publication elsewhere, is not being considered for publication elsewhere and does not duplicate material already published. I confirm all authors consent to publication of this Work and authorise the granting of this licence.

For peer review only

Impact of the COVID-19 Pandemic on anxiety and depression symptoms of young people in the Global South: evidence from a four-country cohort study

Catherine Porter[1], Marta Favara[2], Annina Hittmeyer[2], Douglas Scott[2], Alan Sánchez Jiménez[3], Revathi Ellanki[4], Tassew Woldehanna[5], Le Thuc Duc[6], Michelle G. Craske[7], Alan Stein[8,9,10]

28th January 2021

[1] Lancaster University Management School, Bailrigg, Lancaster LA1 4YX, United Kingdom
[2] Oxford Department of International Development, University of Oxford, 3 Mansfield Rd, Oxford OX1 3TB, United Kingdom
[3] Grupo de Análisis para el Desarrollo (GRADE), Av. Grau 915, Barranco, Lima 15063, Peru
[4] Centre for Economic and Social Studies (CESS), Nizamia Observatory Campus, Begumpet, Hyderabad 500016, Telangana, India
[5] Department of Economics, Addis Ababa University (AAU), P.O. Box 150175, Addis Ababa, Ethiopia
[6] Centre for Analysis and Forecasting, Vietnam Academy of Social Sciences, No.1, Lieu Giai Street, Hanoi, Vietnam
[7] Distinguished Professor Joanne and George Miller and Family Endowed Term Chair (BRI), Director, Anxiety and Depression Research Center (ADRC), Director, Innovative Treatment Network, UCLA Depression Grand Challenge, Associate Director, Staglin Family Music Center for Behavioral & Brain Health, Editor-in-Chief, Behaviour Research and Therapy, UCLA Department of Psychology and Department of Psychiatry & Biobehavioral Sciences, , University of California, Franz Hall – Box 951563, Los Angeles, CA 90094-1563, United States
[8] Department of Psychiatry, University of Oxford, Warneford Hospital, Oxford OX3 7JX, United Kingdom
[9] Africa Health Research Institute (AHRI), KwaZulu-Natal, South Africa
[10] MRC/Wits Rural Public Health and Health Transitions Research Unit (Agincourt), School of Public Health, Faculty of Health Sciences, University of the Witwatersrand, Johannesburg, South Africa

Correspondence to:
Dr Catherine Porter, Lancaster University Management School, Bailrigg, Lancaster LA1 4YX, United Kingdom, **catherine.porter@lancaster.ac.uk,** +44 7905 760608

Wordcount: 4241 words

For peer review only

For peer review only - http://bmjopen.bmj.com/site/about/guidelines.xhtml

ABSTRACT (300 words)

Objective To provide evidence on the effect of the COVID-19 pandemic on the mental health of adolescents and young adults who grew up in poverty in Low and Middle Income Countries (LMICs).

Design A phone survey implemented August-October 2020 to participants of a population-based cohort study since 2002 comprising two cohorts born in 1994-5 and 2001-2 in Ethiopia, India (Andhra Pradesh and Telangana), Peru and Vietnam. We examined associations between mental health and pandemic-related stressors, as well as structural factors (gender, location, wealth); and lifelong protective/risk factors (parent and peer relationship, past household wealth, long-term health problems, past emotional problems and subjective well-being) measured at younger ages.

Setting A diverse, poverty focused sample, reaching those without mobile phones or internet access.

Participants 10,496 individuals were approached, 9,730 participated. Overall, 8,988 individuals were included in this study, 4,610 (51%) male and 4,378 (49%) female. Non-inclusion was due to non-location or missing data.

Main outcome measures At least mild anxiety and depression were measured by Generalized Anxiety Disorder-7 (GAD-7, ≥5) and Patient Health Questionnaire-8 (PHQ-8, ≥5).

Results Rates of symptoms of at least mild anxiety (depression) were highest in Peru at 41% (32%) [95% CI, 38.63-43.12; (29.49-33.74)], and lowest in Vietnam at 9% (9%) [95% CI, 8.16- 10.58; (8.33- 10.77)], mirroring COVID-19 mortality rates. Females were most affected in all countries but Ethiopia. In all countries, pandemic-related stressors were associated with increased rates of anxiety and depression, though with varying levels of importance across countries. Prior parent and peer relationships were protective factors for mental health while having a long-term health problem or prior emotional problems were risk factors.

Conclusion The COVID-19 pandemic presents significant risks to the mental health of young people. Mental health support is limited in LMICs and young people have to date been lower priority for COVID-19 interventions.

Strengths and limitations of this study

- The study uses data from adolescents and young adults who grew up in poverty in four LMICs which were diversely affected by the COVID-19 pandemic, therefore investigating a globally vulnerable, but understudied group both in terms of age and wealth.
- This study reaches a broad sample of young people who grew up in poverty, including those without internet or mobile phone access.
- A key strength is combining a broad range of pandemic-related stressors from survey data on experiences of COVID-19 with previously measured information on longer-term risk and protective factors, therefore contributing to a more complete picture of COVID-19 effects.
- A limitation of the study is that it does not have a directly comparable pre-COVID baseline for depression/anxiety, however, proxy variables are used as a baseline and the explanatory variables capture dynamics that happened during the pandemic.
- A further limitation is possible underreporting due to stigma associated with mental health, despite piloting and validation, as well as possible bias in self-reported experiences of pandemic-related stressors due to feelings of anxiety or depression.

For peer review only - http://bmjopen.bmj.com/site/about/guidelines.xhtml

INTRODUCTION

The Coronavirus disease 2019 (COVID-19) is creating concerns about the mental health of young people around the globe. There has been a call for research funders and researchers to "deploy resources to understand the psychological effects"[1] of the COVID-19 pandemic and the ensuing "mental health crisis".[2] The crisis likely exacerbates previous risk factors of poverty and vulnerability. The Lancet Commission on Global Mental Health had already identified poverty as a key risk factor for the onset and persistence of mental disorders. [3] A recent study[4] found that those with the lowest income were much more likely to suffer from anxiety and depressive disorders than their wealthier counterparts and point to the bidirectional causal relationship between poverty and mental health.

Several studies have examined the mental health impacts of the pandemic, predominantly in high-income countries.[5,6,7] The few studies from Low-and-Middle-income countries (LMICs) have primarily relied on convenience samples, and internet-based surveys (e.g.[8-11]), unlikely reaching the rural poor, though one study[12] investigated the effect of immediate lockdown orders on (adult) women's mental health and experiences of intimate partner violence using a phone survey in rural Bangladesh.

Half of all mental health conditions develop by 14 years of age and 75% by early adulthood.[3] In developed countries young women aged 16-24 are the most likely to have experienced a deterioration in mental health during the pandemic.[12] Thus, understanding risk and protective factors during the pandemic at this age is critical to prevention, especially for the poorest. There is little research on the mental health of adolescents in LMICs, though they make up the bulk of the global adolescent population.[13]

This study examines the impact of the COVID-19 pandemic on the mental health of nearly 10,000 young people from a 20-year cohort study operating in four LMICs: Ethiopia, India (Andhra Pradesh and Telangana), Peru and Vietnam. When the cohorts were originally recruited, the objective was to ensure that families living in poverty were substantially represented. [14-17] Nowadays, these countries represent a diverse set of experiences during the pandemic, in terms of number and severity of cases as well as policy responses. Figure 1 shows that COVID-19 has had by far the most striking impact in Peru in terms of deaths per population, followed by India. In contrast, Vietnam has been hailed as a success story in controlling the spread of the virus.

Pressure on mental health is likely to be greater in countries which are more affected by the pandemic. Within each country, hypothesised stressors related to changes in circumstances/behaviours/wellbeing that occurred due to the pandemic (*COVID-19 related stressors*) include individuals' perceived infection risk, economic adversities, changes in employment status and increased household responsibilities, educational disruption, and changes in subjective well-being between 2016 and the pandemic. Regarding *structural factors* (age, gender, rural/urban place of residence) we expect that residents of urban areas may have difficulties social distancing in slum-like conditions and possibly more likely than residents in rural areas to develop mental health conditions. Similarly, depression and anxiety symptoms might be more frequent among females than males. Changes imposed by COVID-19 in time use, education and work may impact the genders differently. We exploited information collected in previous survey rounds to investigate past *protective/risk factors measured during childhood and adolescence*: positive social interactions (parent and peer relations), past household wealth, and long-term health

For peer review only - http://bmjopen.bmj.com/site/about/guidelines.xhtml

conditions. It also allows us to include *proxy baseline information for mental health* in the form of past emotional problems and subjective well-being measured 10 years ago at age 15.

METHODS

Study design and participants

A phone survey[18-21] was administered between August and October 2020 to participants of the Young Lives study, a longitudinal survey established in 2002 following two cohorts of children born in 1994-5 and 2000-1 in Ethiopia, India (Andhra Pradesh and Telangana), Peru and Vietnam. Respondents have been surveyed in person every three years, with five consecutive rounds completed by 2016. In 2020, the cohort members are aged 18-19 (younger cohort) and 25-26 years (older cohort). The original sample was selected to include a significant coverage of poorer areas.[14-17] 93% of the cohort (9,704) were tracked in 2019. The sample reduced to 8,988 individuals due to missing values for any question (Supplementary Table 1 and Supplementary Table 2) including between 0.1% (Vietnam) and 2% (Peru) who did not respond to the mental health questions (Supplementary Table 3).

The phone survey was administered over mobile telephone by up to 15 trained interviewers per country with access to hardware, software and internet access required for working from home. Responses were recorded in an electronic questionnaire using *Surveybe* Implementer software.

Symptoms of anxiety and depression were measured using the Generalized Anxiety Disorder-7 (GAD-7) scale and the Patient Health Questionnaire depression scale-8 (PHQ-8). The GAD-7 has been validated[22] and used in all four study countries.[23-28] The PHQ-9 was also validated[29-33] and used in several studies.[25-28 34-39] The scales were slightly adapted for administration in a phone survey. First, we asked participants whether they were alone in the room and if not, whether they could find a quiet space and/or make sure their phone speaker was off. Second, for each item in GAD-7 and PHQ-8 we asked whether the symptom had been observed (Yes/No) over the past 14 days, and if "Yes" we then asked about the frequency. The scales were administered as the last section of the survey.

GAD-7 scores between 5 and 9, 10 and 14 and above 15 represent mild, moderate, and severe anxiety, respectively.[40] A PHQ-8 score between 5 and 9, 10 and 14, 15 and 19 and above 19 were considered representative of mild, moderate, moderately severe, and severe depression, respectively.[41] Cronbach's Alpha[42] for both scales was close to or above 0.7.[43] Inter-item correlations fell within the recommended range (0·15 to 0·50[44 45]; Supplementary Table 4).

Statistical analysis

Logistic regressions were used to examine the relationship between a range of stressors on a binary variable indicating (at least) mild anxiety (GAD-7≥5) and (at least) mild depression (PHQ-8 ≥5), as reported in the Results section. We include four sets of stressors hypothesised to be associated with mental health: *changes in circumstances/behaviours/wellbeing that occurred due to the pandemic, past risk/protective factors, structural factors* and *proxy baseline information* (emotional problems and subjective wellbeing measured 10 years earlier). The characteristics of the sample population are shown in Supplementary Table 5. Figure 2 gives an overview of the variables used in the analysis and the respective ages when they were measured.

For peer review only - http://bmjopen.bmj.com/site/about/guidelines.xhtml

The first set of *COVID-19 related stressors* include perceived COVID-19 infection risk, the extent to which people practice self-isolation (having left the house in the past 7 days), increased household responsibilities (including spending more time caring for children, on household chores or working in a family business), suffering from any adverse economic events (including increases in the price of food, incurring increased health expenditures, fewer clients in a family business, and if so, whether the household reduced food consumption to cope with it) and changes in working status compared to before the pandemic. In further analysis for the 19-year-old cohort we replaced working status with engagement with education, given that more than half were still enrolled when the pandemic began (Supplementary tables 6 and 7). Finally, among the *COVID-19 related stressors*, we included the change in subjective well-being (SWB) between 2016 and the pandemic. SWB was measured in Round 5 (2016) at ages 15 and 22, and in the phone survey. Cantril's Ladder (1965)[46] asks respondents to visualise a ladder of nine steps; the bottom (top) step representing their worst (best) possible life. Respondents are asked to identify which step they presently stand on. Difference *in subjective well-being* is a continuous variable ranging from -8 to +8.

Past risk and protective factors include long-term health problems and the past household wealth[47], both measured in 2016, and Parent-child and Peer-child relationships measured using the total raw scores of the Marsh Self-Description Questionnaires II[48] and I[49]. Both scores range between 8 and 32, with higher scores being positive. Peer relationships were obtained at ages 15 (younger cohort) and 22 (older cohort) in 2016. Parent relationship was obtained at ages 15 (younger cohort, 2016) and 19 (older cohort, 2013).

GAD-7 and PHQ-8 were not measured in previous survey rounds. Therefore, we control for p*roxy baseline information* including emotional problems and SWB, both available for the 25-year-old cohort only in 2009 (round 3) at the age of 15 (Supplementary tables 8 and 9). The Emotional Problem Scale comes from the self-completed Strengths and Difficulties Questionnaire (SDQ). Total scores range from 0 to 10, a higher score indicates more severe emotional problems. We report extensive disaggregated rates of mental health issues (Supplementary table 10 to supplementary table 15).

Changes in responsibilities, the labour market and education environment may affect males and females differently. Therefore, we re-estimated the regressions separately by gender (Supplementary table 16 to supplementary table 23). We report odds ratios, robust standard errors and the 95% confidence intervals for all regressions.

Ethics statement

The survey was approved by the institutional research ethics committees at the University of Oxford (UK), the University of Addis Ababa (Ethiopia), the Centre for Economic and Social Studies in Hyderabad (India), the Instituto de Investigación Nutricional (Peru) and the Hanoi University of Public Health (Vietnam). Participants were asked for their verbal informed consent before the study commenced and were assured of confidentiality. A consultation guide was provided to all participants with resources for support in issues raised by the questionnaire, including mental health.

Patient and public involvement

No patients or the public were involved in the study design, setting the research questions, interpretation or writing up of results, or reporting of the research.

For peer review only - http://bmjopen.bmj.com/site/about/guidelines.xhtml

RESULTS

93% of the Young Lives sample located during the last tracking exercise in November 2019 across the four countries participated in the phone survey and only between 0·1% (Vietnam) and 2% (Peru) did not respond to the mental health questions. The results presented in this section refer to the 19-year olds and 25-year olds together, unless differently specified.

Both mild anxiety (41%, 95% CI 38·63-43·12) and mild depression (32%, 29·49-33·74) rates were highest in Peru, followed by Ethiopia (anxiety: 18%, 16·28-19·54; depression: 15%, 13·95- 17·02), see Table 1. The rates of moderate/severe anxiety and depression were highest in Peru, 13·5% (95% CI 12·00-15·14) and 9·6% (8·35-11·07), and below 3% in the other countries (Supplementary tables 24 to 31). Females had significantly higher rates of anxiety symptoms in all countries except Ethiopia and higher rates of depression in Peru and Vietnam. In Peru, almost half of all females had symptoms consistent with at least mild anxiety. Rates of anxiety and depression in rural areas were significantly lower than urban rates in Ethiopia and Peru, but significantly higher in India. The poorest wealth tercile had significantly lower rates of anxiety in Ethiopia and Peru, but higher rates in India and Vietnam. In Peru, the poorest wealth tercile also had significantly lower rates of depression. Those not having any access to the internet, although a minority, had significantly higher levels of anxiety (Vietnam p<0·01, Peru p<0·1).

We noted a high correlation between GAD-7 and PHQ-8 scores (minimum 0·610 (p <0·01) (India) and maximum 0·700 (p <0·01) (Peru), and the rate of having both (at least mild) anxiety and depression symptoms was quite high, with values of up to 24·8% (95% CI 22·87- 26·81) in Peru (Supplementary Table 32 and 33).

The significant risk and protective factors were similar. For brevity, the main results refer to associations with experiencing at least mild anxiety symptoms (see **Error! Reference source not found.**) and at the end we comment on differences with results on depression (see **Error! Reference source not found.**) for the sample pooling the two cohorts and females and males together, unless differently specified.

For peer review only - http://bmjopen.bmj.com/site/about/guidelines.xhtml

Table 1. Rates of at least mild depression and anxiety in Ethiopia, India, Peru and Vietnam

Rates of at least mild anxiety	Ethiopia		India		Peru		Vietnam	
	% at least mild Anxiety	CI95%	% at least mild Anxiety	CI95%	% at least mild Anxiety	CI95%	% at least mild Anxiety	CI95%
**Total**	**17·87**	**16·28; 19·54**	**11·06**	**9·88; 12·32**	**40·86**	**38·63; 43·12**	**9·32**	**8·16; 10·58**
Male	17·48	15·30; 19·65	9·28***	7·74; 10·81	33·65***	30·65; 36·64	7·92**	6·33; 9·51
Female	18·32	15·93; 20·70	13·01	11·14; 14·87	48·28	45·06; 51·49	10·63	8·88; 12·39
Rural	14·67***	12·65; 16·70	11·81*	10·34; 13·27	33·71***	28·79; 38·63	9·07	7·47; 10·67
Urban	21·61	19·06; 24·16	9·20	7·13; 11·27	42·52	40·04; 45	9·62	7·83; 11·40
Poorest tercile	14·90**	12·29; 17·51	13·52***	11·26; 15·78	36·92**	33·01; 40·84	11·67***	9·41; 13·92
Middle/Richest terciles	19·32	17·29; 21·34	9·82	8·42; 11·21	42·63	39·94; 45·32	8·11	6·74; 9·49
No internet	15·97**	13·82; 18·11	12·16	8·43; 15·89	51·32*	40; 62·63	22·86***	8·74; 36·98
With internet	19·87	17·47; 22·27	10·92	9·65; 12·19	40·42	38·16; 42·68	9·11	7·92; 10·30
**Rates of at least mild depression**	**% at least mild Depression**	**CI95%**	**% at least mild Depression**	**CI95%**	**% at least mild Depression**	**CI95%**	**% at least mild Depression**	**CI95%**
**Total**	**15·44**	**13·95; 17·02**	**9·92**	**8·80; 11·12**	**31·58**	**29·49; 33·74**	**9·49**	**8·33; 10·77**
Male	14·92	12·88; 16·96	9·64	8·08; 11·21	27·38***	24·55; 30·21	7·65***	6·09; 9·22
Female	16·04	13·77; 18·31	10·22	8·54; 11·89	35·91	32·83; 39·00	11·22	9·42; 13·02
Rural	12·89***	10·98; 14·81	10·84**	9·43; 12·25	26·40**	21·82; 30·99	9·39	7·77; 11·01
Urban	18·43	16·03; 20·83	7·60	5·70; 9·50	32·79	30·44; 35·14	9·62	7·83; 11·40
Poorest tercile	14·35	11·78; 16·91	10·80	8·74; 12·85	26·15***	22·59; 29·72	8·46	6·51; 10·42
Middle/Richest terciles	15·97	14·10; 17·85	9·47	8·10; 10·85	34·02	31·45; 36·60	10·03	8·51; 11·54
No internet	14·09*	12·06; 16·13	10·14	6·69; 13·58	31·58	21·05; 42·11	14·29	2·52; 26·05
With internet	16·85	14·60; 19·11	9·89	8·67; 11·10	31·58	29·44; 33·73	9·42	8·22; 10·63
N	2183		2622		1887		2296	

*Notes: If any missing answers to questions then the whole score is set to missing. *** p<0.01; ** p<0.05; *p<0.1. Stars represent significance of t-test of equality between groups (Male-Female; Rural-Urban; Bottom-Top/Middle wealth tercile;, Internet access through home computer/working smartphone (No-Yes)). Poorest and middle/richest terciles refer to the household's position in the 2016 (round 5) wealth distribution. Internet access refers to having internet access through a home computer and/or a working smart phone.*

For peer review only - http://bmjopen.bmj.com/site/about/guidelines.xhtml

Table 2. Logistic regression results: Symptoms of at least mild anxiety

	Ethiopia		India		Peru		Vietnam	
	Odds Ratio	95% CI	Odds Ratio	95% CI	Odds Ratio	95% CI	Odds Ratio	95% CI
Structural factors								
Age in months	1.008*** (0.00)	[1.005,1.011]	1.001 (0.00)	[0.998,1.005]	0.999 (0.00)	[0.997,1.002]	0.995** (0.00)	[0.991,0.999]
Female	1.074 (0.16)	[0.810,1.426]	1.601*** (0.27)	[1.149,2.231]	1.706*** (0.18)	[1.389,2.095]	1.295* (0.20)	[0.961,1.746]
Urban	1.276* (0.18)	[0.974,1.670]	0.903 (0.15)	[0.658,1.238]	1.441** (0.22)	[1.072,1.937]	1.206 (0.18)	[0.895,1.625]
COVID-19 related stressors								
Risk perception: believe they are at medium/high risk	0.813 (0.11)	[0.629,1.052]	1.265* (0.16)	[0.980,1.633]	1.460*** (0.15)	[1.200,1.777]	1.022 (0.19)	[0.717,1.459]
Left house for any reason in the past 7 days	0.817 (0.20)	[0.501,1.332]	1.401* (0.27)	[0.955,2.056]	0.994 (0.13)	[0.776,1.274]	1.054 (0.25)	[0.666,1.668]
Difference in subjective well-being R5-Call2	1.070** (0.03)	[1.012,1.133]	1.099** (0.04)	[1.020,1.184]	1.105*** (0.03)	[1.053,1.159]	1.181*** (0.05)	[1.081,1.291]
Change in responsibilities:								
Spend more time taking care of children	0.891 (0.14)	[0.652,1.219]	2.209*** (0.37)	[1.593,3.063]	1.354*** (0.14)	[1.100,1.667]	1.401* (0.25)	[0.986,1.991]
Spend more time on household chores	1.059 (0.16)	[0.794,1.414]	0.756* (0.11)	[0.565,1.011]	1.204 (0.15)	[0.943,1.537]	1.209 (0.19)	[0.888,1.645]
Spend more time working in the family business	0.772 (0.16)	[0.520,1.149]	1.605* (0.46)	[0.916,2.811]	1.288* (0.18)	[0.976,1.699]	1.790*** (0.37)	[1.196,2.679]
Economic adversities								
Faced with new health expenses	0.937 (0.18)	[0.646,1.359]	1.046 (0.14)	[0.798,1.370]	1.733*** (0.19)	[1.404,2.139]	1.240 (0.33)	[0.733,2.098]
Experienced adversity but did not reduce food consumption	2.362*** (0.49)	[1.567,3.561]	0.621 (0.28)	[0.253,1.524]	2.495** (1.00)	[1.140,5.461]	1.624*** (0.28)	[1.161,2.270]
Reduced food consumption as response to experienced adversity	7.187*** (1.71)	[4.510,11.454]	0.871 (0.46)	[0.310,2.446]	2.402** (1.06)	[1.009,5.718]	1.673** (0.37)	[1.089,2.570]
Changes in employment status								
Did not work before the pandemic, but is working now	2.667*** (0.64)	[1.671,4.259]	0.993 (0.22)	[0.643,1.533]	0.866 (0.16)	[0.609,1.232]	1.104 (0.35)	[0.594,2.053]
Worked before the pandemic and is working now/has a job	1.570*** (0.24)	[1.156,2.132]	1.288 (0.22)	[0.925,1.795]	1.068 (0.15)	[0.815,1.399]	0.978 (0.20)	[0.653,1.464]
Worked before the pandemic and is not working now/does not have a job	2.294*** (0.45)	[1.559,3.376]	2.504*** (0.65)	[1.501,4.175]	1.205 (0.18)	[0.893,1.628]	1.346 (0.33)	[0.834,2.171]
Educational disruption								
Enrolled in/Planning to enrol in full time education and not participating in learning activities[a]	1.588** (0.33)	[1.052,2.396]	1.043 (0.21)	[0.707,1.538]	0.600 (0.27)	[0.245,1.471]	0.828 (0.25)	[0.458,1.495]
Enrolled in/Planning to enrol in full time education and participating in	1.110 (0.38)	[0.565,2.182]	0.710 (0.17)	[0.450,1.120]	1.026 (0.12)	[0.816,1.290]	0.699* (0.15)	[0.465,1.051]

For peer review only

For peer review only - http://bmjopen.bmj.com/site/about/guidelines.xhtml

	Ethiopia		India		Peru		Vietnam	
	Odds Ratio	95% CI	Odds Ratio	95% CI	Odds Ratio	95% CI	Odds Ratio	95% CI
learning activities[a]								
Past protective/risk factors								
Participant has long-term health problem, 2016 (Round 5)	1.424* (0.27)	[0.984,2.060]	1.399* (0.25)	[0.989,1.981]	1.805*** (0.26)	[1.354,2.406]	1.337 (0.27)	[0.897,1.991]
Total parent-child relationship score, 2012/2016 (Round 4/5)	0.967 (0.02)	[0.926,1.009]	0.923*** (0.02)	[0.883,0.965]	0.976* (0.01)	[0.949,1.004]	1.041 (0.03)	[0.992,1.093]
Total peer-child relationship score, 2016 (Round 5)	0.941*** (0.02)	[0.901,0.982]	1.025 (0.03)	[0.975,1.078]	1.001 (0.02)	[0.970,1.033]	0.941* (0.03)	[0.876,1.011]
Middle/Top wealth tercile R5, 2016 (Round 5)	1.124 (0.16)	[0.846,1.495]	0.781* (0.11)	[0.587,1.038]	1.016 (0.13)	[0.797,1.295]	0.550*** (0.09)	[0.404,0.747]
Proxy baseline information								
Emotional problem scale (EPS) score, 2009 (Round 3)[b]	1.074* (0.04)	[0.994,1.161]	1.007 (0.05)	[0.910,1.114]	1.222*** (0.07)	[1.093,1.366]	1.072 (0.07)	[0.940,1.221]
Subjective well-being, 2009 (Round 3)[b]	0.979 (0.06)	[0.872,1.100]	1.131** (0.07)	[1.010,1.267]	0.924 (0.07)	[0.800,1.068]	0.975 (0.08)	[0.827,1.148]
N	2183		2622		1887		2296	

*Note: Odds ratios are unadjusted odds ratios. Robust standard errors in parenthesis, *** significant at 1%, ** significant at 5%, * significant at 10%. Base categories are as follows: Male, Rural, Believe they are at no/low risk, Did not leave the house at all during the past 7 days, Did not spend more time taking care of children, Did not spend more time on household chores, Did not spend more time working in the family business, Did not face new health expenses, Did not suffer a shock, Did not work at all in the past 12 month OR worked during the pandemic but not before and is not working now, Never attended school or not enrolled in full-time education/not planning to enrol, Does not have long-term health condition, Lowest wealth tercile. All time-variant variables are measured in 2020 unless otherwise specified. The regression was run on the joint Younger Cohort /Older Cohort sample except for the following results which were added from independent regression specifications run on the Younger and Older Cohort only:*

[a]results come from a separate regression where Educational disruption was substituted for Changes in employment status and which was run on the Younger Cohort (18-19) sample only (Supplementary Table 6).

[b] results come from a separate regression where we added proxy baseline information measured in 2009 at age 15 and which was run on the Older Cohort (25-26) sample only (Supplementary Table 8)

Table 3. Logistic regression results: Symptoms of at least mild depression

	Ethiopia		India		Peru		Vietnam	
	Odds Ratio	95% CI	Odds Ratio	95% CI	Odds Ratio	95% CI	Odds Ratio	95% CI
Structural factors								
Age in months	1.001 (0.00)	[0.998,1.005]	1.000 (0.00)	[0.997,1.004]	0.996** (0.00)	[0.993,0.999]	0.994*** (0.00)	[0.989,0.998]
Female	1.142 (0.17)	[0.847,1.541]	1.239 (0.21)	[0.890,1.725]	1.317** (0.15)	[1.059,1.637]	1.405** (0.21)	[1.048,1.883]
Urban	1.356** (0.20)	[1.019,1.805]	0.733* (0.12)	[0.526,1.022]	1.114 (0.17)	[0.820,1.513]	0.961 (0.15)	[0.706,1.308]
COVID-19 related stressors								

For peer review only - http://bmjopen.bmj.com/site/about/guidelines.xhtml

	Ethiopia		India		Peru		Vietnam	
	Odds Ratio	95% CI	Odds Ratio	95% CI	Odds Ratio	95% CI	Odds Ratio	95% CI
Risk perception: believe they are at medium/high risk	0.620*** (0.08)	[0.481,0.800]	0.962 (0.13)	[0.736,1.258]	1.595*** (0.17)	[1.297,1.963]	1.107 (0.20)	[0.778,1.575]
Left house for any reason in the past 7 days	0.995 (0.26)	[0.596,1.661]	2.285*** (0.53)	[1.454,3.590]	1.055 (0.14)	[0.813,1.368]	0.965 (0.21)	[0.629,1.480]
Difference in subjective well-being R5-Call2	1.089*** (0.03)	[1.026,1.155]	1.119*** (0.04)	[1.041,1.202]	1.080*** (0.03)	[1.026,1.137]	1.163*** (0.05)	[1.068,1.267]
Change in responsibilities:								
Spend more time taking care of children	0.852 (0.14)	[0.623,1.165]	1.611** (0.30)	[1.121,2.316]	1.145 (0.13)	[0.919,1.425]	1.455** (0.26)	[1.021,2.074]
Spend more time on household chores	1.128 (0.17)	[0.834,1.526]	0.877 (0.14)	[0.649,1.186]	1.061 (0.14)	[0.821,1.371]	1.550*** (0.25)	[1.133,2.119]
Spend more time working in the family business	0.948 (0.20)	[0.633,1.419]	1.053 (0.35)	[0.551,2.011]	1.302* (0.19)	[0.975,1.738]	1.804*** (0.35)	[1.228,2.650]
Economic adversities								
Faced with new health expenses	0.522*** (0.11)	[0.342,0.796]	1.289* (0.19)	[0.967,1.716]	1.739*** (0.19)	[1.401,2.159]	0.909 (0.26)	[0.523,1.580]
Experienced adversity but did not reduce food consumption	3.808*** (0.95)	[2.336,6.206]	0.646 (0.32)	[0.244,1.713]	2.049* (0.86)	[0.896,4.686]	1.695*** (0.30)	[1.203,2.390]
Reduced food consumption as response to experienced adversity	10.890*** (3.00)	[6.341,18.701]	0.641 (0.38)	[0.203,2.027]	2.531** (1.17)	[1.019,6.285]	1.907*** (0.41)	[1.249,2.912]
Changes in employment status								
Did not work before the pandemic, but is working now	2.657*** (0.64)	[1.655,4.265]	0.685* (0.16)	[0.438,1.072]	0.920 (0.17)	[0.643,1.316]	0.715 (0.24)	[0.375,1.363]
Worked before the pandemic and is working now/has a job	1.378** (0.22)	[1.010,1.881]	0.873 (0.15)	[0.620,1.229]	0.841 (0.12)	[0.634,1.114]	0.810 (0.16)	[0.552,1.190]
Worked before the pandemic and is not working now/does not have a job	1.679** (0.36)	[1.104,2.555]	1.597 (0.45)	[0.914,2.790]	1.283 (0.20)	[0.940,1.751]	1.509* (0.34)	[0.974,2.336]
Educational disruption								
Enrolled in/Planning to enrol in full time education and not participating in learning activities[a]	1.728*** (0.36)	[1.153,2.592]	1.103 (0.22)	[0.743,1.638]	0.743 (0.36)	[0.287,1.928]	0.944 (0.28)	[0.529,1.685]
Enrolled in/Planning to enrol in full time education and participating in learning activities[a]	1.174 (0.41)	[0.591,2.333]	0.749 (0.18)	[0.469,1.196]	1.042 (0.13)	[0.816,1.329]	1.172 (0.25)	[0.768,1.788]
<u>*Past protective/risk factors*</u>								
Participant has long-term health problem, 2016 (Round 5)	0.954 (0.21)	[0.620,1.470]	1.566** (0.28)	[1.101,2.226]	1.590*** (0.23)	[1.197,2.113]	1.328 (0.27)	[0.889,1.982]
Total parent-child relationship score, 2012/2016 (Round 4/5)	1.004 (0.02)	[0.960,1.050]	0.909*** (0.02)	[0.867,0.954]	0.939*** (0.01)	[0.911,0.968]	0.983 (0.02)	[0.938,1.031]
Total peer-child relationship score,	0.938*** (0.02)	[0.895,0.983]	1.015 (0.03)	[0.963,1.070]	1.007 (0.02)	[0.973,1.042]	0.988 (0.04)	[0.920,1.062]

For peer review only - http://bmjopen.bmj.com/site/about/guidelines.xhtml

	Ethiopia		India		Peru		Vietnam	
	Odds Ratio	95% CI	Odds Ratio	95% CI	Odds Ratio	95% CI	Odds Ratio	95% CI
2016 (Round 5)								
Middle/Top wealth tercile R5, 2016 (Round 5)	0.884 (0.13)	[0.656,1.192]	1.074 (0.16)	[0.802,1.437]	1.249* (0.16)	[0.968,1.613]	1.068 (0.18)	[0.770,1.482]
Proxy baseline information								
Emotional problem scale (EPS) score, 2009 (Round 3)[b]	1.090* (0.05)	[0.995,1.195]	1.101* (0.06)	[0.991,1.223]	1.129** (0.07)	[1.003,1.270]	1.114 (0.08)	[0.973,1.276]
Subjective well-being, 2009 (Round 3)[b]	0.894 (0.06)	[0.782,1.023]	1.077 (0.07)	[0.952,1.219]	0.869* (0.07)	[0.736,1.026]	0.952 (0.08)	[0.805,1.126]
N	2183		2622		1887		2296	

*Note: Odds ratios are unadjusted odds ratios. Robust standard errors in parenthesis, *** significant at 1%, ** significant at 5%, * significant at 10%. Base categories are as follows: Male, Rural, Believe they are at no/low risk, Did not leave the house at all during the past 7 days, Did not spend more time taking care of children, Did not spend more time on household chores, Did not spend more time working in the family business, Did not face new health expenses, Did not suffer a shock, Did not work at all in the past 12 month OR worked during the pandemic but not before and is not working now, Never attended school or not enrolled in full-time education/not planning to enrol, Does not have long-term health condition, Lowest wealth tercile. All time-variant variables are measured in 2020 unless otherwise specified. The regression was run on the joint Younger Cohort /Older Cohort sample except for the following results which were added from independent regression specifications run on the Younger and Older Cohort only:*

[a]results come from a separate regression where Educational disruption was substituted for Changes in employment status and which was run on the Younger Cohort (19-20) sample only (Supplementary Table 7).

[b] results come from a separate regression where we added proxy baseline information measured in 2009 at age 15 and which was run on the Older Cohort (25-26) sample only (Supplementary Table 9).

For peer review only

For peer review only - http://bmjopen.bmj.com/site/about/guidelines.xhtml

Structural factors: for females, the odds of at least mild anxiety were 1·30 (95% CI 0·96-1·75, p<0·1) (Vietnam), 1·60 (1·15-2·23, p<0·01) (India) and 1·70 (1·39-2·10, p<0·01) (Peru) times greater than the odds for males (n.s. in Ethiopia). Urban location increased odds significantly in Ethiopia and Peru. Age was not significant in India and Peru, protective in Vietnam, and a risk factor in Ethiopia.

COVID-related stressors

COVID-19 infection risk perception: The odds of those who believed that they were at medium/high risk of catching the virus were 1·27 (95% CI 0·98 - 1·63, p<0·1) (India) to 1·46 (1·20 - 1·78, p<0·01) (Peru) times higher than for those who believed themselves to have no/low risk. The former group had rates of at least mild anxiety of 12% (India) and 45% (Peru).

Leaving the house for at least one day a week: No significant effects, except in India where it increased the odds of anxiety by 1·40 (95% CI, 0·96-2·06, p<0·1).

Economic adversity: For those who suffered from economic adversity (e.g. fewer clients in the family business, food price increase) odds of anxiety were higher (p<0·01 (Ethiopia and Vietnam), (2·50, 95% CI 1·14-5·46, p<0·05) (2·40, 1·01-5·72, p<0·05) (Peru)) even if it did not cause reduced food consumption. Moreover, in Ethiopia and Vietnam, those who reduced food consumption as a coping strategy had 7·19 (95% CI 4·51-11·45, p<0·01) (Ethiopia), 1·67 (1·09-2·57, p<0·05) (Vietnam) higher odds than those who experienced an adverse event but did not need to reduce food consumption in response 2·36 (1·57-3·56, p<0·01) (Ethiopia) and 1·62 (1·16 - 2·27, p<0·01) (Vietnam) (both compared to those who did not experience any adverse event at all). In Ethiopia, 36% of those who reduced food consumption reported at least mild anxiety compared to 7% for those who did not experience an adverse event (p<0·0001). In Ethiopia, odds were higher among females than males, but in Peru and Vietnam significant for males only. Facing new health expenses significantly increased the odds of displaying at least mild symptoms of anxiety by 1·73 (95% CI 1·40 - 2·14) (p <0·01) in Peru. Over half (52%, p- <0.0001) of those who faced new health expenses report at least mild anxiety (although not significant in India or Ethiopia, significant risk factor for females in Vietnam (1·76, 95% CI 0·93-3·31, p<0·1)).

Increased responsibilities: Spending more time on childcare during the lockdown increased odds of anxiety by 2·21 (95% CI 1·59-3·06, p<0·01) in India, 1·35 (1·10-1·67, p<0·01) in Peru and 1·4 (0·99-1·99, p<0·1) in Vietnam. Rates of at least mild anxiety for those who spent more time taking care of children were 20% vs 9% (India), 49% vs 37% (Peru) and 13% vs 8% (Vietnam), (all p<0·001). Spending more time on household chores *lowered* odds in India for anxiety (females only). For those who spend more time working in the family business the odds of anxiety were 1·61 (95% CI 0·92-2·81, p<0·1) times higher in India, 1·29 (0·98-1·67, p<0·1, n.s. for females) in Peru and 1·80 (1·20-2·68, p<0·01) in Vietnam (higher odds among males). In Vietnam, those who spent more time working in the family business reported rates of at least mild anxiety at 16% (p<0·0001), the highest among the Vietnamese sample.

Change in subjective well-being (SWB): the large decrease in SWB between 2016 and the pandemic (registered in all countries but Vietnam) is correlated with GAD-7 and PHQ-8 mean scores in the expected direction (Supplementary Table 34). Furthermore, across countries those with at least mild anxiety (depression) reported lower SWB (2016 and 2020) than their counterparts. Moreover, those with at least mild anxiety (depression) reported larger drops in SWB than their non-anxious (non-

For peer review only - http://bmjopen.bmj.com/site/about/guidelines.xhtml

depressed) counterparts (Supplementary Table 35 and Supplementary Table 36). For each one-point decrease in SWB between 2016 and the pandemic, the odds of at least mild anxiety increased by a factor of 1·07 (95% CI 1·01-1·13, p<0·05) (Ethiopia), 1·10 (1·02-1·18, p<0·05) (India), 1·11 (1·05-1·16, p<0·01) (Peru) and 1·18 (1·08-1·29, p<0·01) (Vietnam).

Changes in employment status: In Ethiopia, those who participated in the labour market had higher odds of anxiety than those who did not (e.g. full-time students, stay-at-home parents). However, the odds of those who were pushed into the labour market (2·67, 95% CI 1·67-4·26, p<0·01) or lost their job (2·29, 1·56-3·38, p<0·01) were higher than those who simply participated (1·57, 1·16-2·13, p<0·01) (all in comparison to non-participants). In India losing a job increased risk of anxiety by 2·50 (95% CI 1·50-4·18, p<0·01). In Peru and Vietnam, there were no employment effects. Rates of at least mild anxiety among those who lost their jobs were among the highest in each country 31% (Ethiopia, p<0·001), 20% (India, p<0·001), 46% (Peru, n.s.), 12% (Vietnam, n.s.).

Educational disruption (19-year-old cohort only): Students who were enrolled in Ethiopia before the pandemic and were unable to access virtual classes, or complete homework had 1·59 (95% CI 1·05-2·40, p<0·05) times higher odds of anxiety than those who were not enrolled. In Vietnam, those who were enrolled and engaged in learning activities had lower odds of anxiety (0·70, 95% CI 0·47-1·05, p<0·1) than those who were not enrolled (base category). The full YC only regression results can be found in the Supplementary Tables 6 and 7; the education results split by gender (again YC only) are located in the Supplementary Tables 20-23.

<u>*Past protective/risk factors*</u>

Long-term health problems (measured in 2016): The odds of at least mild anxiety were 1·42 (95% CI 0·98-2·06, p<0·1) (Ethiopia), 1·40 (1·00-2·00, p<0·1) (India) and 1·80 (1·35-2·41, p<0·01) (Peru) times as large as the odds for those who did not (n.s. in Vietnam). In Peru, those reporting long-term health problems had the highest rates of at least mild anxiety, 56% (p<0·0001).

Parent-child relationship (measured at ages 15 for the younger cohort, in 2016 and measured at age 19 for the older cohort, in 2013) *and peer-child relationship* (at ages 15 for the younger cohort and at age 22 for the older cohort, in 2016): Strong parent-child relationships were a significant protective factor in India and Peru, while peer-child relationships were a significant protective factor in Ethiopia and Vietnam.

Past household wealth (measured in 2016): i.e. being in the middle/highest wealth tercile vs the lowest was a marginally significant protective factor in India (0·78, 95% CI 0·59-1·04, p<0·1) and significant in Vietnam (0·55, 0·40-0·75, p<0·01).

<u>*Proxy baseline information*</u>

Past emotional problems and well-being (25-year-old cohort only): For a one-point increase in previous emotional problems at age 15 (measured in 2009), the odds of at least mild anxiety increased by a factor of 1·22 (1·09 - 1·37, p <0·01) (Peru) and 1·07 (95% CI 0·99 - 1·16, p <0·1) (Ethiopia). Notably, the effect of the COVID-19 related stressors holds when controlling for past proxy baseline information. The full older cohort only regression results can be found in Supplementary Tables 8 and 9.

For peer review only - http://bmjopen.bmj.com/site/about/guidelines.xhtml

Significant differences between anxiety and depression regression results (See ***Error! Reference source not found.***). In Ethiopia, food insecurity had a higher impact on depression for males than females. In India, subjective high infection risk increased anxiety, but not depression, and those faced with new health expenses had higher odds of depression, but not anxiety. Females had higher rates of anxiety but not depression. In Peru, childcare was not a risk factor for depression, and past SWB was a protective factor. In Vietnam losing a job was a significant risk factor for depression, while good peer relations and education were not significant for depression.

DISCUSSION

We examined the impact of the COVID-19 pandemic on the mental health of young people in Ethiopia, India, Peru and Vietnam. The sample has broad coverage of the poorer population in each country, and we interviewed 93% of those located during the tracking prior the pandemic, including those without internet access and in Ethiopia also those without mobile phone, who would be excluded from an online survey. Internet access has had both positive and negative effects in the pandemic[50]. In our sample, those without access to internet have significantly higher rates of anxiety in Vietnam and Peru.

The four countries have had different experiences of the pandemic – Peru is the most affected country in terms of deaths per population, and Vietnam the least. India, Peru and Vietnam imposed strict lockdowns to control the spread of the virus, and Ethiopia restricted certain activities such as school closure but did not impose a strict lockdown, though faced other challenges (locust infestations, food price inflation and violence).

This study reveals a strong relationship between the severity of the pandemic and the rates of mental health conditions in our sample, both in terms of anxiety and depression symptoms. Rates of at least mild anxiety (depression) were four (three) times higher in Peru compared to Vietnam. Furthermore, the 2020 survey showed a significant fall in subjective wellbeing from 2016 in all countries except Vietnam. The fall in subjective wellbeing is highly correlated with anxiety and depression symptoms. A strong correlation between SWB and our mental health indicators is important as in absence of baseline measures of GAD-7 and PhQ-8, it suggests that SWB is a useful proxy baseline.

The economic impact of the pandemic has affected certain groups of young people in all study countries, even Vietnam, and Ethiopia where there was no full national lockdown. Overall, our findings confirmed that those experiencing COVID-19 related stressors had worse mental health, although the relative importance of stressors varied across countries: increased health expenses and believing they were at a medium/high infection risk was detrimental for young people in Peru, but increased food insecurity was much more important in Ethiopia, reflecting high rates of food price inflation in 2019 which continued into 2020. Moreover, peer relations in earlier years was a protective factor for anxiety and depression only in Ethiopia. In Peru and Vietnam there were no employment effects on anxiety, likely for very different reasons – in Peru health concerns were more important, and in Vietnam the labour market was relatively resilient.

Exploiting the longitudinal data allowed us to investigate past protective/risk factors. As expected, parent and peer relations measured during childhood and adolescence were protective, though in different ways across countries. Strong parental relationships were a significant protective factor in India and Peru, whereas peer relationships were more important in Ethiopia and Vietnam. Those reporting

For peer review only - http://bmjopen.bmj.com/site/about/guidelines.xhtml

long term health problems were twice as likely to display symptoms consistent with at least mild anxiety, this effect being particularly pronounced in Peru. Previous relative wealth was a significant protective factor only in India and Vietnam. Pre-pandemic emotional problems were risk factors, especially in Ethiopia and Peru. The associations with COVID-19 related stressors were robust to the inclusion of pre-pandemic emotional problems and past subjective well-being.

Other studies used longitudinal data to document the impact of the pandemic on mental health,[5 51] though none investigated a general population of young people of a similar age in our study countries that we can directly compare to, though results from UK studies have similar findings. The closest study to ours, a phone survey in a developing country finds a deterioration in maternal mental health in rural Bangladesh.[51] Our study shows lower rates of anxiety and depression in rural areas in Ethiopia and Peru, but significantly higher in India. We are able to disaggregate the effect of a range of COVID-19 related stressors, which we can relate individually to other studies. A study in Hubei province, China [11] showed the importance of income losses during the pandemic. Studies of college students in China[8] and Bangladesh[10] show that educational disruption significantly increased anxiety and depression, similar to our results in Vietnam and Ethiopia. Social support was negatively correlated with the level of anxiety[8], similar to our findings regarding parent/peer-child relationships. In Jordan[9] female healthcare professionals, female university students and university students with chronic disease were at higher risk of developing depression, similar to our results for long-term health problems.

Even controlling for other factors, we found females to be more vulnerable to anxiety in India, Peru and Vietnam and more vulnerable to depression in Peru and Vietnam. In Ethiopia we found no significant gender effects. While most studies[5 9 11] have similar findings regarding females, one[8] finds no gender differences among Chinese college students while male Bangladeshi students[10] had higher depressive symptoms than females.

Strengths and limitations

Other studies have used longitudinal data to document the impact of the pandemic on mental health.[5, 12] This study's strength combines survey data about experiences of COVID-19 with long term information from two cohorts of participants of a population-based cohort study in four LMICs. The study was able to cover the poorest, those without internet (or mobile phone), and examine a broad range of pandemic-related stressors as well as controlling for longer-term risk and protective factors. Our study has a number of limitations. We do not have a directly comparable pre-COVID baseline for depression/anxiety. However, we use proxy variables for baseline and our explanatory variables capture dynamics during the pandemic. Finally, as for other studies, there may be underreporting because of stigma associated with mental health, despite piloting and validation; however, note that our analysis identifies high risk groups *within* each country. Also self-reported variables may be biased due to feelings of anxiety or depression. The findings are not fully generalizable to the whole population of LMICs due to the pro poor design and age group, however they broadly represent poor young people in the study countries.

CONCLUSION

Adolescents and young people have been lower priority for COVID-19 interventions, given the lower rates of hospitalization and death for this age group. This research shows that the pandemic is having important effects on the mental health of certain groups of young people even in countries with fewer

For peer review only - http://bmjopen.bmj.com/site/about/guidelines.xhtml

cases. Mental health services are very limited in LMICs making it urgent to develop evidence-based and sustainable prevention programmes in response to the pandemic.

FIGURE CAPTIONS

Figure 1. Cumulative confirmed COVID-19 cases per million people in the four Young Lives countries

Source: Johns Hopkins University CSSE COVID-19 Data[52], accessed via our world in data[53]. Last updated 15 December 2020. Testing and challenges in the attribution of the cause of death means that the number of confirmed deaths may not be an accurate count of the true number of deaths from COVID-19.

Figure 2. Variables used in analysis

Variables used in the analysis. Structural factors refer to age, gender and rural/urban location.

For peer review only

For peer review only

For peer review only - http://bmjopen.bmj.com/site/about/guidelines.xhtml

Author affiliations

[1] Lancaster University Management School, Bailrigg, Lancaster LA1 4YX, United Kingdom
[2] Oxford Department of International Development, University of Oxford, 3 Mansfield Rd, Oxford OX1 3TB, United Kingdom
[3] Grupo de Análisis para el Desarrollo (GRADE), Av. Grau 915, Barranco, Lima 15063, Peru
[4] Centre for Economic and Social Studies (CESS), Nizamia Observatory Campus, Begumpet, Hyderabad 500016, Telangana, India
[5] Department of Economics, Addis Ababa University (AAU), P.O. Box 150175, Addis Ababa, Ethiopia
[6] Centre for Analysis and Forecasting, Vietnam Academy of Social Sciences, No.1, Lieu Giai Street, Hanoi, Vietnam
[7] Distinguished Professor Joanne and George Miller and Family Endowed Term Chair (BRI), Director, Anxiety and Depression Research Center (ADRC), Director, Innovative Treatment Network, UCLA Depression Grand Challenge, Associate Director, Staglin Family Music Center for Behavioral & Brain Health, Editor-in-Chief, Behaviour Research and Therapy, UCLA Department of Psychology and Department of Psychiatry & Biobehavioral Sciences, , University of California, Franz Hall – Box 951563, Los Angeles, CA 90094-1563, United States
[8] Department of Psychiatry, University of Oxford, Warneford Hospital, Oxford OX3 7JX, United Kingdom
[9] Africa Health Research Institute (AHRI), KwaZulu-Natal, South Africa
[10] MRC/Wits Rural Public Health and Health Transitions Research Unit (Agincourt), School of Public Health, Faculty of Health Sciences, University of the Witwatersrand, Johannesburg, South Africa

Acknowledgments We particularly wish to thank the Young Lives respondents and their families for generously giving us their time and cooperation during this difficult time as well as fieldworkers and field managers in the four study countries. We are gratefully indebted to Professor George Patton for his helpful comments.

Author Contributions CP and MF conceived the study. CP, MF, DS, and ASJ designed the study. MF, ASJ, RE, TW, LTD led data collection. CP and AH did the statistical analyses. CP, AH and MF wrote the first draft of the Article. AS and MK provided comments and input to the several drafts of the article. MF, DS, ASJ verified the underlying data. AS, MF, CP and AH assessed scale validation and methodology. AH prepared the supplementary files. All authors critically reviewed multiple versions of the manuscript and approved the final version. The authors alone are responsible for the views expressed in this article.

Funding statement Young Lives at Work is funded by UK aid from the Foreign, Commonwealth and Development Office (FCDO), under Department for International Development, UK Government grant number 200425. The funders of the study had no role in study design, data collection, data analysis, data interpretation, or writing of the report. The views expressed are those of the authors. They are not necessarily those of, or endorsed by, the University of Oxford, Young Lives, FCDO. All authors had full access to the anonymized data in the study and had final responsibility for the decision to submit for publication.

Competing interests CP, MF, AH, DS, ASJ, RE, TW and LTD report grants from the Foreign, Commonwealth and Development Office (FCDO), during the conduct of the study. MC and AS have nothing to disclose.

Patient consent for publication Not required.

For peer review only

Ethics approval The survey was approved by the institutional research ethics committees at the University of Oxford (UK), the University of Addis Ababa (Ethiopia), the Centre for Economic and Social Studies in Hyderabad (India), the Instituto de Investigación Nutricional (Peru) and the Hanoi University of Public Health (Vietnam). Participants were asked for their verbal informed consent before the study commenced and were assured of confidentiality. A consultation guide was provided to all participants with resources for support in issues raised by the questionnaire, including mental health.

Data sharing statement The entire individual participant data collected during the phone survey and previous in-person rounds, after de-identification, is available including data dictionaries. Furthermore the questionnaire, attrition reports and the field work manual are available at https://www.younglives.org.uk/ . The data will be available in January 2021, with no end date to anyone who wishes to access the data for any purpose, via the UK Data Archive (study number 8678, DOI: 10.5255/UKDA-SN-8678-1).

For peer review only

For peer review only - http://bmjopen.bmj.com/site/about/guidelines.xhtml

Reference List

1. Holmes EA, O'Connor RC, Perry VH, et al. Multidisciplinary research priorities for the COVID-19 pandemic: a call for action for mental health science. *The Lancet Psychiatry* 2020;7(6):547-60. doi: 10.1016/s2215-0366(20)30168-1
2. WHO. Substantial investment needed to avert mental health crisis. 2020. https://www.who.int/news/item/14-05-2020-substantial-investment-needed-to-avert-mental-health-crisis (accessed 14.05.2020).
3. Patel V, Saxena S, Lund C, et al. The Lancet Commission on global mental health and sustainable development. *The Lancet* 2018;392(10157):1553-98. doi: 10.1016/s0140-6736(18)31612-x
4. Ridley M, Rao G, Schilbach F, et al. Poverty, depression, and anxiety: Causal evidence and mechanisms. *Science* 2020;370(6522) doi: 10.1126/science.aay0214 [published Online First: 2020/12/12]
5. Pierce M, Hope H, Ford T, et al. Mental health before and during the COVID-19 pandemic: a longitudinal probability sample survey of the UK population. *The Lancet Psychiatry* 2020;7(10):883-92. doi: 10.1016/s2215-0366(20)30308-4
6. Pan K-Y, Kok AAL, Eikelenboom M, et al. The mental health impact of the COVID-19 pandemic on people with and without depressive, anxiety, or obsessive-compulsive disorders: a longitudinal study of three Dutch case-control cohorts. *The Lancet Psychiatry* 2020 doi: 10.1016/s2215-0366(20)30491-0
7. Czeisler MÉ, Lane RI, Petrosky E, et al. Mental Health, Substance Use, and Suicidal Ideation During the COVID-19 Pandemic — United States. *MMWR Morb Mortal Wkly Rep 2020* 2020;69:1049–57. doi: 10.15585/mmwr.mm6932a1external
8. Cao W, Fang Z, Hou G, et al. The psychological impact of the COVID-19 epidemic on college students in China. *Psychiatry Res* 2020;287:112934. doi: 10.1016/j.psychres.2020.112934 [published Online First: 2020/04/02]
9. Naser AY, Dahmash EZ, Al-Rousan R, et al. Mental health status of the general population, healthcare professionals, and university students during 2019 coronavirus disease outbreak in Jordan: A cross-sectional study. *Brain Behav* 2020;10(8):e01730. doi: 10.1002/brb3.1730 [published Online First: 2020/06/25]
10. Islam MA, Barna SD, Raihan H, et al. Depression and anxiety among university students during the COVID-19 pandemic in Bangladesh: A web-based cross-sectional survey. *PLoS One* 2020;15(8):e0238162. doi: 10.1371/journal.pone.0238162 [published Online First: 2020/08/28]
11. Li X, Lu P, Hu L, et al. Factors Associated with Mental Health Results among Workers with Income Losses Exposed to COVID-19 in China. *Int J Environ Res Public Health* 2020;17(15) doi: 10.3390/ijerph17155627 [published Online First: 2020/08/08]
12. Banks J, Xu X. The mental health effects of the first two months of lockdown and social distancing during the Covid-19 pandemic in the UK. *IFS Working Paper W20/16* 2020
13. Blum R, Boyden J. Understand young people in low-income countries. *Nature* 2018;544
14. Escobal J, Flores E. An Assessment of the Young Lives Sampling Approach in Peru. Technical Note. Oxford: Young Lives, 2008.
15. Kumra N. An Assessment of the Young Lives Sampling Approach in Andhra Pradesh, India. Technical Note. Oxford: Young Lives, 2008.
16. Nguyen NP. An Assessment of the Young Lives Sampling Approach in Vietnam. Technical Note. Oxford: Young Lives, 2008.
17. Outes-Leon I, Sanchez A. An Assessment of the Young Lives Sampling Approach in Ethiopia. Technical Note. Oxford: Young Lives, 2008.

For peer review only - http://bmjopen.bmj.com/site/about/guidelines.xhtml

18. Scott D, Thuc DL, Hittmeyer A, et al. Listening to Young Lives at Work in Vietnam: Second Call. *Listening to Young Lives at Work Phone Survey* 2020; 2.4. https://www.younglives.org.uk/sites/www.younglives.org.uk/files/YOL-Vietnam-Headlines-PhoneSurvey2-Nov20.pdf (accessed 24.11.2020).
19. Porter C, Woldehanna T, Freund R, et al. Listening to Young Lives at Work in Ethiopia: Second Call. *Listening to Young Lives at Work Phone Survey* 2020; 2.1. https://www.younglives.org.uk/sites/www.younglives.org.uk/files/YOL-Ethiopia-Headlines-PhoneSurvey2-Nov20.pdf (accessed 24.11.2020).
20. Favara M, Ellanki R, Molina MdlÁ, et al. Listening to Young Lives at Work in Andhra Pradesh and Telangana: Second Call. *Listening to Young Lives at Work Phone Survey* 2020; 2.2. https://www.younglives.org.uk/sites/www.younglives.org.uk/files/YOL-India-Headlines-PhoneSurvey2-Nov20.pdf (accessed 24.11.2020).
21. Sánchez A, Cueto S, Mary Penny JL, et al. Listening to Young Lives at Work in Peru: Second Call. *Listening to Young Lives at Work Phone Survey* 2020; 2.3. https://www.younglives.org.uk/sites/www.younglives.org.uk/files/YOL-Peru-Headlines-PhoneSurvey2-Nov20-2%2023rd%20.pdf (accessed 24.11.2020).
22. Zhong QY, Gelaye B, Zaslavsky AM, et al. Diagnostic Validity of the Generalized Anxiety Disorder - 7 (GAD-7) among Pregnant Women. *PLoS One* 2015;10(4):e0125096. doi: 10.1371/journal.pone.0125096 [published Online First: 2015/04/29]
23. Gezie LD, Yalew AW, Gete YK, et al. Socio-economic, trafficking exposures and mental health symptoms of human trafficking returnees in Ethiopia: using a generalized structural equation modelling. *Int J Ment Health Syst* 2018;12:62. doi: 10.1186/s13033-018-0241-z [published Online First: 2018/11/06]
24. Dadi AF, Dachew BA, Kisi T, et al. Anxiety and associated factors among prisoners in North West of Amhara Regional State, Ethiopia. *BMC Psychiatry* 2016;16:83. doi: 10.1186/s12888-016-0792-y [published Online First: 2016/04/03]
25. Hakim A, Tak H, Nagar S, et al. Assessment of prevalence of depression and anxiety and factors associated with them in undergraduate medical students of Dr. S. N. Medical College, Jodhpur. *International Journal Of Community Medicine And Public Health* 2017;4(9) doi: 10.18203/2394-6040.ijcmph20173826
26. Collier KM, Weiss B, Pollack A, et al. Explanatory variables for women's increased risk for mental health problems in Vietnam. *Soc Psychiatry Psychiatr Epidemiol* 2020;55(3):359-69. doi: 10.1007/s00127-019-01761-3 [published Online First: 2019/08/30]
27. Pham Tien N, Pham Thanh T, Nguyen Hanh D, et al. Utilization of mental health services among university students in Vietnam. *International Journal of Mental Health* 2020:1-23. doi: 10.1080/00207411.2020.1816114
28. Pollack AA, Weiss B, Trung LT. Mental health, life functioning and risk factors among people exposed to frequent natural disasters and chronic poverty in Vietnam. *BJPsych Open* 2016;2(3):221-32. doi: 10.1192/bjpo.bp.115.002170 [published Online First: 2016/07/28]
29. Carroll HA, Hook K, Perez OFR, et al. Establishing reliability and validity for mental health screening instruments in resource-constrained settings: Systematic review of the PHQ-9 and key recommendations. *Psychiatry Res* 2020;291:113236. doi: 10.1016/j.psychres.2020.113236 [published Online First: 2020/07/01]
30. Zhong QY, Gelaye B, Rondon MB, et al. Using the Patient Health Questionnaire (PHQ-9) and the Edinburgh Postnatal Depression Scale (EPDS) to assess suicidal ideation among pregnant women in Lima, Peru. *Arch Womens Ment Health* 2015;18(6):783-92. doi: 10.1007/s00737-014-0481-0 [published Online First: 2014/11/06]

For peer review only - http://bmjopen.bmj.com/site/about/guidelines.xhtml

31. Gelaye B, Williams MA, Lemma S, et al. Validity of the Patient Health Questionnaire-9 for depression screening and diagnosis in East Africa. *Psychiatry Res* 2013;210(2):653-61. doi: 10.1016/j.psychres.2013.07.015 [published Online First: 2013/08/27]
32. Woldetensay YK, Belachew T, Tesfaye M, et al. Validation of the Patient Health Questionnaire (PHQ-9) as a screening tool for depression in pregnant women: Afaan Oromo version. *PLoS One* 2018;13(2):e0191782. doi: 10.1371/journal.pone.0191782 [published Online First: 2018/02/07]
33. Nguyen TQ, Bandeen-Roche K, Bass JK, et al. A tool for sexual minority mental health research: The Patient Health Questionnaire (PHQ-9) as a depressive symptom severity measure for sexual minority women in Viet Nam. *J Gay Lesbian Ment Health* 2016;20(2):173-91. doi: 10.1080/19359705.2015.1080204 [published Online First: 2016/09/20]
34. Mokona H, Yohannes K, Ayano G. Youth unemployment and mental health: prevalence and associated factors of depression among unemployed young adults in Gedeo zone, Southern Ethiopia. *Int J Ment Health Syst* 2020;14:61. doi: 10.1186/s13033-020-00395-2 [published Online First: 2020/08/13]
35. Molla GL, Sebhat HM, Hussen ZN, et al. Depression among Ethiopian Adults: Cross-Sectional Study. *Psychiatry J* 2016;2016:1468120. doi: 10.1155/2016/1468120 [published Online First: 2016/06/02]
36. Gupta R, Taneja N, Anand T, et al. Internet Addiction, Sleep Quality and Depressive Symptoms Amongst Medical Students in Delhi, India. *Community Ment Health J* 2020 doi: 10.1007/s10597-020-00697-2 [published Online First: 2020/08/28]
37. Roberts T, Shidhaye R, Patel V, et al. Health care use and treatment-seeking for depression symptoms in rural India: an exploratory cross-sectional analysis. *BMC Health Serv Res* 2020;20(1):287. doi: 10.1186/s12913-020-05162-0 [published Online First: 2020/04/08]
38. Hernandez-Vasquez A, Rojas-Roque C, Vargas-Fernandez R, et al. Dynamics of depressive symptoms and within-country migration among Peruvian women. *Rural Remote Health* 2020;20(2):5692. doi: 10.22605/RRH5692 [published Online First: 2020/04/03]
39. Hernandez-Vasquez A, Vargas-Fernandez R, Bendezu-Quispe G, et al. Depression in the Peruvian population and its associated factors: analysis of a national health survey. *J Affect Disord* 2020;273:291-97. doi: 10.1016/j.jad.2020.03.100 [published Online First: 2020/05/19]
40. Spitzer RL, Kroenke K, Williams JB, et al. A brief measure for assessing generalized anxiety disorder: the GAD-7. *Arch Intern Med* 2006;166(10):1092-7. doi: 10.1001/archinte.166.10.1092 [published Online First: 2006/05/24]
41. Kroenke K, Strine TW, Spitzer RL, et al. The PHQ-8 as a measure of current depression in the general population. *J Affect Disord* 2009;114(1-3):163-73. doi: 10.1016/j.jad.2008.06.026 [published Online First: 2008/08/30]
42. Cronbach LJ. Coefficient alpha and the internal structure of tests. *Psychometrika* 1951;16(3):297-334. doi: 10.1007/bf02310555
43. Nunnally J, Bernstein I. Psychometric Theory. 3 ed. New York: McGraw-Hill 1994.
44. Briggs SR, Cheek JM. The role of factor analysis in the development and evaluation of personality scales. *Journal of Personality* 1986;54(1):106-48. doi: 10.1111/j.1467-6494.1986.tb00391.x
45. Clark LA, Watson D. Constructing validity: Basic issues in objective scale development. *Psychological Assessment* 1995;7(3):309-19. doi: 10.1037/1040-3590.7.3.309
46. Cantril H. The pattern of human concerns: New Brunswick, N. J. : Rutgers University Press, [1965] 1965.
47. Briones K. 'How Many Rooms Are There in Your House?'Constructing the Young Lives Wealth Index. 2017; 43. https://www.younglives.org.uk/sites/www.younglives.org.uk/files/YL-TN43_0.pdf.

For peer review only - http://bmjopen.bmj.com/site/about/guidelines.xhtml

48. Marsh HW. Self Description Questionnaire (SDQ) II: A theoretical and empirical basis for the measurement of multiple dimensions of adolescent self-concept. San Antonio, TX Psychological Corporation 1990.
49. Marsh HW. The Self Description Questionnaire (SDQ): A theoretical and empirical basis for the measurement of multiple dimensions of preadolescent self-concept: A test manual and research monograph. San Antonio, TX Psychological Corporation 1988.
50. Amsalem D, Dixon LB, Neria Y. The Coronavirus Disease 2019 (COVID-19) Outbreak and Mental Health: Current Risks and Recommended Actions. *JAMA Psychiatry* 2021;78(1):9-10. doi: 10.1001/jamapsychiatry.2020.1730 [published Online First: 2020/06/25]
51. Hamadani JD, Hasan MI, Baldi AJ, et al. Immediate impact of stay-at-home orders to control COVID-19 transmission on socioeconomic conditions, food insecurity, mental health, and intimate partner violence in Bangladeshi women and their families: an interrupted time series. *The Lancet Global Health* 2020;8(11):e1380-e89. doi: 10.1016/s2214-109x(20)30366-1
52. Dong E, Du H, Gardner L. An interactive web-based dashboard to track COVID-19 in real time. *The Lancet Infectious Diseases* 2020;20(5):533-34. doi: 10.1016/s1473-3099(20)30120-1
53. Ritchie H, Ortiz-Ospina E, Beltekian D, et al. Coronavirus Pandemic Data Explorer. In: OurWorldInData.org, ed., 2020.

For peer review only

For peer review only - http://bmjopen.bmj.com/site/about/guidelines.xhtml

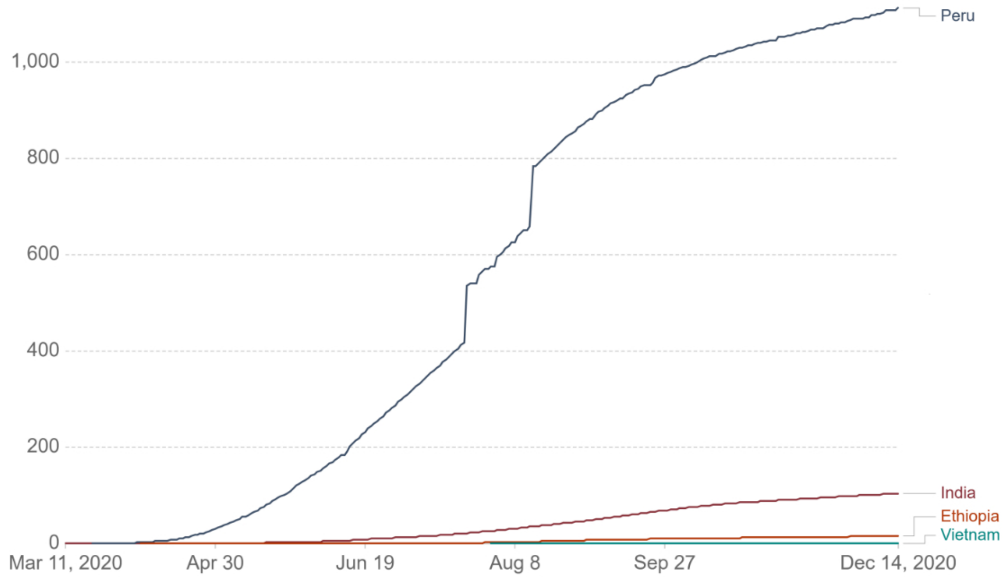

Figure 1. Cumulative confirmed COVID-19 cases per million people in the four Young Lives countries. Source: Johns Hopkins University CSSE COVID-19 Data52, accessed via our world in data. Last updated 15 December 2020. Testing and challenges in the attribution of the cause of death means that the number of confirmed deaths may not be an accurate count of the true number of deaths from COVID-19.

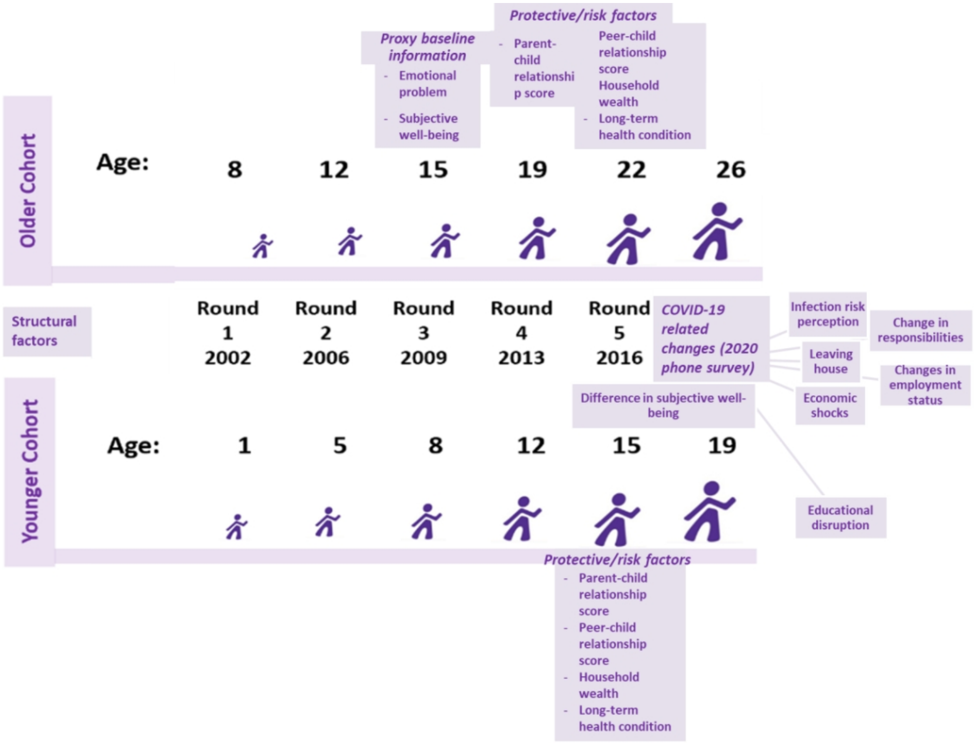

Figure 2. Variables used in the analysis. Structural factors refer to age, gender and rural/urban location.

Supplemental material

Supplement to: Impact of the COVID-19 Pandemic on anxiety and depression symptoms of young people in the Global South: evidence from a four-country cohort study

Supplementary Table 1. Sample size 3
Supplementary Table 2. Attrition rates (%) 3
Supplementary Table 3. Non-response rate to mental health questions (%)........ 4
Supplementary Table 4. Measures of reliability and internal consistency........ 4
Supplementary Table 5. Sample description 5
Supplementary Table 6. Logistic regression results: Symptoms of at least mild anxiety (Younger Cohort only) 7
Supplementary Table 7. Logistic regression results: Symptoms of at least mild depression (Younger Cohort only) 8
Supplementary Table 8. Logistic regression results: Symptoms of at least mild anxiety (Older Cohort only) 10
Supplementary Table 9. Logistic regression results: Symptoms of at least mild depression (Older Cohort only) 11
Supplementary Table 10. Ethiopia: Means of continuous control variables by at least mild anxiety/depression 14
Supplementary Table 11. India: Means of continuous control variables by at least mild anxiety/depression 15
Supplementary Table 12. Peru: Means of continuous control variables by at least mild anxiety/depression 16
Supplementary Table 13. Vietnam: Means of continuous control variables by at least mild anxiety/depression 17
Supplementary Table 14. Mental health outcomes by control variables (Ethiopia & India)........ 18
Supplementary Table 15. Mental health outcomes by control variables (Peru & Vietnam) 19
Supplementary Table 16. Logistic regression results: Symptoms of at least mild anxiety/depression by gender (Ethiopia)........ 21
Supplementary Table 17. Logistic regression results: Symptoms of at least mild anxiety/depression by gender (India)........ 22
Supplementary Table 18. Logistic regression results: Symptoms of at least mild anxiety/depression by gender (Peru) 24
Supplementary Table 19. Logistic regression results: Symptoms of at least mild anxiety/depression by gender (Vietnam) 25
Supplementary Table 20. Logistic regression results: Symptoms of at least mild anxiety/depression by gender for the Younger Cohort only (Ethiopia)........ 28
Supplementary Table 21. Logistic regression results: Symptoms of at least mild anxiety/depression by gender for the Younger Cohort only (India)........ 29
Supplementary Table 22. Logistic regression results: Symptoms of at least mild anxiety/depression by gender for the Younger Cohort only (Peru) 31

For peer review only

Supplementary Table 23. Logistic regression results: Symptoms of at least mild anxiety/depression by gender for the Younger Cohort only (Vietnam) 32
Supplementary Table 24. Ethiopia: Mean score and rates of general anxiety disorder 34
Supplementary Table 25. Ethiopia: Mean score and rates of depression................. 35
Supplementary Table 26.India: Mean score and rates of general anxiety disorder .. 36
Supplementary Table 27. India: Mean score and rates of depression...................... 37
Supplementary Table 28. Peru: Mean score and rates of general anxiety disorder . 38
Supplementary Table 29. Peru: Mean score and rates of depression 39
Supplementary Table 30. Vietnam: Mean score and rates of general anxiety disorder 40
Supplementary Table 31. Vietnam: Mean score and rates of depression................. 41
Supplementary Table 32. Correlation between GAD-7 and PHQ-8 scores 42
Supplementary Table 33. Joint rates 42
Supplementary Table 34. Correlation between subjective well-being and GAD-7/PHQ-8 scores 43
Supplementary Table 35. Subjective well-being score Round 5 (2016) / phone survey (2020) by anxiety/depression (Ethiopia & India) 44
Supplementary Table 36. Subjective well-being score Round 5 (2016) / phone survey (2020) by anxiety/depression (Peru & Vietnam) 45

For peer review only

Supplementary Table 1. Sample size

	Ethiopia			India			Peru			Vietnam			Total		
	Full sample	YC	OC	Full sample	YC	OC	Full sample	YC	OC	Full sample	YC	OC	Full sample	YC	OC
Round 5, 2016	2626	1812	814	2822	1900	922	2468	1860	608	2848	1938	910	10764	7510	3254
Tracking samples, 2019-2020	2634	1803	831	2815	1902	913	2215	1701	514	2832	1922	910	10496	7328	3168
Phone survey, 2020	2439	1665	774	2754	1868	886	2018	1561	457	2519	1691	828	9730	6785	2945
Mental health measures available	2433	1659	774	2753	1867	886	1977	1535	442	2517	1690	827	9680	6751	2929
Included in our analysis	2183	1533	650	2622	1786	836	1887	1496	391	2296	1596	700	8988	6411	2577

Notes: YC stands for Younger Cohort, OC for Older Cohort.

Supplementary Table 2. Attrition rates (%)

	Ethiopia			India			Peru			Vietnam			Total		
	Full sample	YC	OC	Full sample	YC	OC	Full sample	YC	OC	Full sample	YC	OC	Full sample	YC	OC
Tracking sample to phone survey	7.4	7.7	6.9	2.2	1.8	3.0	8.9	8.2	11.1	11.1	12.0	9.0	7.3	7.4	7.0

For peer review only

Supplementary Table 3. Non-response rate to mental health questions (%)

Ethiopia			India			Peru			Vietnam			Total		
Full sample	YC	OC	Full sample	YC	OC	Full sample	YC	OC	Full sample	YC	OC	Full sample	YC	OC
0.2	0.4	0.0	0.0	0.1	0.0	2.0	1.7	3.3	0.1	0.1	0.1	0.5	0.5	0.5

Supplementary Table 4. Measures of reliability and internal consistency

		Cronbach's Alpha	Inter-item correlation	Kaiser–Meyer–Olkin measure of sampling adequacy
**Ethiopia**	GAD-7	0.728	0.277	0.792
	PHQ-8	0.652	0.190	0.744
**India**	GAD-7	0.691	0.242	0.777
	PHQ-8	0.637	0.180	0.744
**Peru**	GAD-7	0.804	0.369	0.872
	PHQ-8	0.773	0.299	0.839
**Vietnam**	GAD-7	0.749	0.299	0.814
	PHQ-8	0.748	0.271	0.807

Note: Results are for the combined Younger Cohort/ Older Cohort sample.

For peer review only

Supplementary Table 5. Sample description

	Ethiopia		India		Peru		Vietnam	
	Mean	sd	Mean	sd	Mean	sd	Mean	sd
Structural factors								
Female	46.27	0.499	47.79	0.500	49.28	0.500	51.61	0.500
Urban	45.99	0.499	28.60	0.452	81.13	0.391	45.73	0.498
COVID-19 related stressors								
Risk perception: believe they are at medium/high risk	69.31	0.461	46.38	0.499	54.27	0.498	21.39	0.410
Left house for any reason in the past 7 days	94.50	0.228	79.25	0.406	80.45	0.397	87.67	0.329
Difference in subjective well-being 2016-2020	0.96	2.068	0.49	1.949	0.52	2.066	-0.39	1.876
Change in responsibilities:								
Spend more time taking care of children	29.00	0.454	18.31	0.387	34.23	0.475	22.91	0.420
Spend more time on household chores	46.82	0.499	50.95	0.500	77.53	0.417	51.44	0.500
Spend more time working in the family business	12.96	0.336	4.12	0.199	15.00	0.357	10.89	0.312
Economic adversities								
Faced with new health expenses	10.12	0.302	55.95	0.497	29.57	0.456	6.53	
Did not experience adversity	20.71	0.405	1.72	0.130	2.60	0.159	42.25	0.494
Experienced adversity but did not reduce food consumption	65.83	0.474	92.68	0.261	90.94	0.287	41.94	0.494
Reduced food consumption as response to experienced adversity	13.47	0.341	5.61	0.230	6.47	0.246	15.81	0.365
Changes in employment status								
Did not work at all in the past 12 month OR worked during the pandemic but not before the pandemic (and is not working now)	46.17	0.499	41.38	0.493	24.06	0.428	23.48	0.424
Did not work before the pandemic, but is working now	6.83	0.252	16.40	0.370	12.35	0.329	6.75	0.251
Worked before the pandemic and is working now/has a job	38.71	0.487	37.57	0.484	46.74	0.499	56.36	0.496
Worked before the pandemic and is not working now/does not have a job	8.29	0.276	4.65	0.211	16.85	0.374	13.41	0.341
Educational disruption								
Never attended school or not enrolled in full-time education/not planning to enrol[a]	32.68	0.469	33.87	0.473	58.96	0.492	47.49	0.500
Enrolled in/Planning to enrol in full time education and not participating in learning activities[a]	58.71	0.493	36.28	0.481	1.87	0.136	12.72	0.333
Enrolled in/Planning to enrol in full time education and participating in learning activities[a]	8.61	0.281	29.84	0.458	39.17	0.488	39.79	0.490

For peer review only

	Ethiopia		India		Peru		Vietnam	
	Mean	sd	Mean	sd	Mean	sd	Mean	sd
<u>Past protective/risk factors</u>								
Child has long-term health problem, 2016 (Round 5)	8.80	0.283	11.48	0.319	12.67	0.333	14.20	0.349
Total parent-child relationship score, 2012/2016 (Round 4/5)	26.26	2.848	27.63	2.916	24.86	3.610	25.74	3.204
Total peer-child relationship score, 2016 (Round 5)	24.13	2.866	25.02	2.631	23.41	3.204	22.57	
Middle/Top wealth tercile R5, 2016 (Round 5)	67.11	0.470	66.44	0.472	69.00	0.463	66.03	2.257
<u>Proxy baseline information</u>								
Emotional problem scale (EPS) score, 2009 (Round 3)[b]	2.78	2.473	3.57	2.348	4.27	2.323	3.68	2.143
Subjective well-being, 2009 (Round 3)[b]	4.86	1.719	4.73	1.815	6.12	1.616	5.43	1.604
N	2183		2622		1887		2296	

Note: Mean values are reported. Self-reported risk belief coded the question "chance of getting infected with Coronavirus" into no/low risk, or medium/high risk. All time-variant variables are measured in 2020 unless otherwise specified. [a] refers to Younger Cohort (18-19) only, [b] refers to Older Cohort (25-26) only. Around 30% of the sample in Ethiopia, India and Vietnam are part of the Older Cohort. In Peru they make up 20% of the sample. The average age of the Younger Cohort in Ethiopia, India, Peru, Vietnam in years (months) is 18 (226), 18 (227), 18 (227), and 18 (227). The average age of the Older Cohort in Ethiopia, India, Peru, Vietnam in years (months) is 25 (309), 25 (310), 25 (311), and 25 (311).

For peer review only

Supplementary Table 6. Logistic regression results: Symptoms of at least mild anxiety (Younger Cohort only)

	Ethiopia		India		Peru		Vietnam	
	**Odds Ratio**	**95% CI**	**Odds Ratio**	**95% CI**	**Odds Ratio**	**95% CI**	**Odds Ratio**	**95% CI**
Structural factors								
Age in months	1.000 (0.02)	[0.960,1.042]	0.987 (0.02)	[0.946,1.030]	0.991 (0.01)	[0.963,1.020]	1.020 (0.03)	[0.967,1.077]
Female	1.216 (0.21)	[0.874,1.692]	1.420* (0.30)	[0.940,2.143]	1.758*** (0.20)	[1.402,2.205]	1.453** (0.25)	[1.034,2.041]
Urban	1.565** (0.31)	[1.059,2.312]	1.198 (0.24)	[0.806,1.781]	1.362* (0.22)	[0.986,1.882]	1.322 (0.26)	[0.901,1.940]
COVID-19 related changes/behaviours								
Risk perception: believe they are at medium/high risk	0.679** (0.12)	[0.487,0.947]	1.244 (0.21)	[0.898,1.724]	1.432*** (0.16)	[1.149,1.784]	1.031 (0.22)	[0.673,1.579]
Left house for any reason in the past 7 days	0.885 (0.27)	[0.482,1.627]	1.576* (0.41)	[0.948,2.620]	1.023 (0.14)	[0.784,1.335]	1.304 (0.34)	[0.783,2.171]
Difference in subjective well-being between Round 5 (2016) and phone survey (2020)	1.043 (0.04)	[0.969,1.123]	1.103** (0.05)	[1.007,1.207]	1.102*** (0.03)	[1.046,1.161]	1.195*** (0.06)	[1.084,1.316]
Change in responsibilities:								
Spend more time taking care of children	1.012 (0.20)	[0.692,1.479]	2.176*** (0.52)	[1.363,3.475]	1.509*** (0.18)	[1.194,1.906]	1.400 (0.31)	[0.913,2.147]
Spend more time on household chores	0.983 (0.17)	[0.694,1.392]	0.816 (0.15)	[0.570,1.169]	1.180 (0.17)	[0.895,1.557]	1.013 (0.18)	[0.718,1.430]
Spend more time working in the family business	0.703 (0.21)	[0.394,1.253]	2.117** (0.67)	[1.136,3.943]	1.235 (0.19)	[0.917,1.663]	1.640** (0.38)	[1.037,2.593]
Economic shocks								
Faced with new health expenses	0.668 (0.21)	[0.360,1.240]	0.881 (0.16)	[0.624,1.245]	1.741*** (0.21)	[1.371,2.209]	1.309 (0.42)	[0.699,2.455]
Experienced adversity but did not reduce food consumption	3.636*** (0.99)	[2.128,6.213]	0.702 (0.38)	[0.245,2.011]	2.503** (1.08)	[1.074,5.834]	1.493** (0.29)	[1.021,2.184]
Reduced food consumption as response to experienced adversity	8.158*** (2.58)	[4.393,15.149]	1.173 (0.73)	[0.345,3.985]	2.154 (1.03)	[0.844,5.498]	1.680** (0.42)	[1.027,2.748]
Educational disruption								
Enrolled in/Planning to enrol in full time education and not participating in learning activities	1.588** (0.33)	[1.052,2.396]	1.043 (0.21)	[0.707,1.538]	0.600 (0.27)	[0.245,1.471]	0.828 (0.25)	[0.458,1.495]
Enrolled in/Planning to enrol in full time education and participating in learning activities	1.110 (0.38)	[0.565,2.182]	0.710 (0.17)	[0.450,1.120]	1.026 (0.12)	[0.816,1.290]	0.699* (0.15)	[0.465,1.051]
Past protective/risk factors								
Participant has long-term health problem, 2016 (Round 5)	1.298 (0.36)	[0.755,2.233]	1.226 (0.30)	[0.754,1.995]	1.624*** (0.28)	[1.163,2.266]	1.030 (0.29)	[0.591,1.793]

For peer review only

	Ethiopia		India		Peru		Vietnam	
	Odds Ratio	95% CI	Odds Ratio	95% CI	Odds Ratio	95% CI	Odds Ratio	95% CI
Total parent-child relationship score, 2012/2016 (Round 4/5)	0.959 (0.03)	[0.902,1.019]	0.912*** (0.03)	[0.861,0.967]	0.975 (0.02)	[0.944,1.007]	1.030 (0.03)	[0.976,1.088]
Total peer-child relationship score, 2016 (Round 5)	0.951 (0.03)	[0.895,1.011]	1.015 (0.04)	[0.948,1.088]	0.999 (0.02)	[0.964,1.035]	0.968 (0.04)	[0.896,1.047]
Middle/Top wealth tercile R5, 2016 (Round 5)	0.908 (0.19)	[0.608,1.356]	0.712* (0.13)	[0.495,1.023]	1.105 (0.15)	[0.845,1.444]	0.677** (0.13)	[0.467,0.981]
N	1533		1786		1496		1596	

*Note: Odds ratios are unadjusted odds ratios. Robust standard errors in parenthesis, *** significant at 1%, ** significant at 5%, * significant at 10%. Base categories are as follows: Male, Rural, Believe they are at no/low risk, Did not leave the house at all during the past 7 days, Did not spend more time taking care of children, Did not spend more time on household chores, Did not spend more time working in the family business, Did not face new health expenses, Did not suffer a shock, Never attended school or not enrolled in full-time education/not planning to enrol, Does not have long-term health condition, Lowest wealth tercile. All time-variant variables are measured in 2020 unless otherwise specified.*

Supplementary Table 7. Logistic regression results: Symptoms of at least mild depression (Younger Cohort only)

	Ethiopia		India		Peru		Vietnam	
	Odds Ratio	95% CI	Odds Ratio	95% CI	Odds Ratio	95% CI	Odds Ratio	95% CI
<u>*Structural factors*</u>								
Age in months	0.999 (0.02)	[0.959,1.041]	0.997 (0.02)	[0.954,1.041]	0.974* (0.01)	[0.945,1.003]	1.028 (0.03)	[0.975,1.084]
Female	1.318 (0.23)	[0.940,1.848]	1.225 (0.25)	[0.821,1.828]	1.282** (0.16)	[1.009,1.630]	1.438** (0.24)	[1.032,2.004]
Urban	1.533** (0.29)	[1.051,2.235]	0.969 (0.20)	[0.644,1.457]	1.175 (0.20)	[0.840,1.643]	1.093 (0.22)	[0.736,1.623]
<u>*COVID-19 related changes/behaviours*</u>								
Risk perception: believe they are at medium/high risk	0.498*** (0.08)	[0.361,0.687]	0.966 (0.16)	[0.693,1.346]	1.548*** (0.18)	[1.230,1.949]	1.025 (0.22)	[0.674,1.559]
Left house for any reason in the past 7 days	0.940 (0.28)	[0.520,1.702]	2.537*** (0.77)	[1.403,4.589]	0.928 (0.13)	[0.704,1.223]	1.163 (0.28)	[0.724,1.867]
Difference in subjective well-being between Round 5 (2016) and phone survey (2020)	1.049 (0.04)	[0.973,1.130]	1.102** (0.05)	[1.011,1.201]	1.079*** (0.03)	[1.020,1.142]	1.169*** (0.06)	[1.065,1.284]
Change in responsibilities:								
Spend more time taking care of children	1.061 (0.20)	[0.736,1.527]	2.170*** (0.51)	[1.370,3.438]	1.268* (0.16)	[0.992,1.621]	1.616** (0.33)	[1.087,2.403]
Spend more time on household chores	1.082 (0.19)	[0.763,1.533]	0.857 (0.16)	[0.592,1.241]	1.142 (0.17)	[0.858,1.520]	1.840*** (0.34)	[1.287,2.631]
Spend more time working in the family business	1.076 (0.29)	[0.636,1.823]	0.803 (0.34)	[0.354,1.823]	1.160 (0.18)	[0.850,1.582]	1.724** (0.38)	[1.121,2.652]

For peer review only

	Ethiopia		India		Peru		Vietnam	
	Odds Ratio	95% CI	Odds Ratio	95% CI	Odds Ratio	95% CI	Odds Ratio	95% CI
Economic shocks								
Faced with new health expenses	0.222*** (0.10)	[0.093,0.530]	1.062 (0.19)	[0.747,1.510]	1.851*** (0.23)	[1.450,2.363]	0.805 (0.28)	[0.410,1.580]
Experienced adversity but did not reduce food consumption	4.913*** (1.49)	[2.712,8.902]	0.651 (0.36)	[0.218,1.944]	2.541* (1.21)	[0.997,6.476]	1.763*** (0.36)	[1.181,2.631]
Reduced food consumption as response to experienced adversity	12.771*** (4.42)	[6.484,25.156]	0.913 (0.61)	[0.248,3.357]	3.620** (1.89)	[1.300,10.079]	2.292*** (0.57)	[1.410,3.726]
Educational disruption								
Enrolled in/Planning to enrol in full time education and not participating in learning activities	1.728*** (0.36)	[1.153,2.592]	1.103 (0.22)	[0.743,1.638]	0.743 (0.36)	[0.287,1.928]	0.944 (0.28)	[0.529,1.685]
Enrolled in/Planning to enrol in full time education and participating in learning activities	1.174 (0.41)	[0.591,2.333]	0.749 (0.18)	[0.469,1.196]	1.042 (0.13)	[0.816,1.329]	1.172 (0.25)	[0.768,1.788]
Past protective/risk factors								
Participant has long-term health problem, 2016 (Round 5)	0.540* (0.20)	[0.265,1.098]	1.461 (0.35)	[0.909,2.346]	1.502** (0.26)	[1.077,2.095]	1.466 (0.37)	[0.889,2.415]
Total parent-child relationship score, 2012/2016 (Round 4/5)	1.011 (0.03)	[0.954,1.072]	0.890*** (0.03)	[0.838,0.945]	0.924*** (0.02)	[0.892,0.957]	0.971 (0.03)	[0.918,1.026]
Total peer-child relationship score, 2016 (Round 5)	0.902*** (0.03)	[0.848,0.960]	1.000 (0.04)	[0.930,1.076]	1.025 (0.02)	[0.987,1.065]	0.987 (0.04)	[0.910,1.071]
Middle/Top wealth tercile R5, 2016 (Round 5)	0.763 (0.15)	[0.518,1.122]	1.275 (0.24)	[0.879,1.851]	1.166 (0.17)	[0.878,1.550]	1.189 (0.24)	[0.797,1.773]
N	1533		1786		1496		1596	

*Note: Odds ratios are unadjusted odds ratios. Robust standard errors in parenthesis, *** significant at 1%, ** significant at 5%, * significant at 10%. Base categories are as follows: Male, Rural, Believe they are at no/low risk, Did not leave the house at all during the past 7 days, Did not spend more time taking care of children, Did not spend more time on household chores, Did not spend more time working in the family business, Did not face new health expenses, Did not suffer a shock, Never attended school or not enrolled in full-time education/not planning to enrol, Does not have long-term health condition, Lowest wealth tercile. All time-variant variables are measured in 2020 unless otherwise specified.*

For peer review only

Supplementary Table 8. Logistic regression results: Symptoms of at least mild anxiety (Older Cohort only)

	Ethiopia		India		Peru		Vietnam	
	Odds Ratio	95% CI	Odds Ratio	95% CI	Odds Ratio	95% CI	Odds Ratio	95% CI
Structural factors								
Age in months	1.008 (0.03)	[0.956,1.063]	1.052* (0.03)	[0.999,1.108]	0.977 (0.03)	[0.923,1.034]	0.960 (0.04)	[0.879,1.047]
Female	0.754 (0.21)	[0.432,1.319]	1.954** (0.58)	[1.087,3.511]	1.125 (0.30)	[0.670,1.888]	0.790 (0.27)	[0.404,1.545]
Urban	1.087 (0.23)	[0.713,1.658]	0.561** (0.15)	[0.334,0.942]	1.825 (0.75)	[0.819,4.065]	0.961 (0.31)	[0.516,1.790]
COVID-19 related changes/behaviours								
Risk perception: believe they are at medium/high risk	1.466 (0.35)	[0.915,2.346]	1.383 (0.30)	[0.908,2.108]	1.693** (0.41)	[1.051,2.728]	1.060 (0.37)	[0.536,2.098]
Left house for any reason in the past 7 days	0.693 (0.33)	[0.274,1.751]	1.257 (0.38)	[0.697,2.267]	0.850 (0.31)	[0.414,1.746]	0.432* 0.21)	[0.169,1.109]
Difference in subjective well-being between Round 5 (2016) and phone survey (2020)	1.089* (0.05)	[0.987,1.201]	1.121* (0.07)	[0.983,1.278]	1.132* (0.08)	[0.990,1.294]	1.105 (0.12)	[0.901,1.356]
Change in responsibilities:								
Spend more time taking care of children	0.735 (0.21)	[0.420,1.285]	2.081***(0.52)	[1.271,3.405]	0.971 (0.25)	[0.590,1.599]	1.230 (0.46)	[0.595,2.544]
Spend more time on household chores	1.313 (0.38)	[0.742,2.325]	0.639* (0.16)	[0.394,1.034]	1.667 (0.53)	[0.893,3.114]	2.240**(0.87)	[1.043,4.808]
Spend more time working in the family business	1.275 (0.41)	[0.682,2.383]	0.498 (0.41)	[0.100,2.478]	1.599 (0.54)	[0.829,3.085]	2.126* (0.96)	[0.879,5.139]
Economic shocks								
Faced with new health expenses	1.523 (0.45)	[0.854,2.716]	1.435 (0.34)	[0.901,2.286]	1.572* (0.38)	[0.984,2.513]	0.808 (0.41)	[0.302,2.164]
Experienced adversity but did not reduce food consumption	0.908 (0.31)	[0.466,1.767]	0.560 (0.49)	[0.102,3.068]	1.312 (1.77)	[0.093,18.595]	1.988* (0.78)	[0.922,4.284]
Reduced food consumption as response to experienced adversity	4.323***(1.81)	[1.900,9.835]	0.567 (0.55)	[0.086,3.751]	2.691 (3.82)	[0.167,43.423]	1.986 (0.96)	[0.772,5.113]
Changes in employment status								
Did not work before the pandemic, but is working now	2.044 (0.97)	[0.809,5.165]	0.951 (0.47)	[0.358,2.525]	1.095 (0.62)	[0.363,3.303]	0.868 (0.78)	[0.149,5.071]
Worked before the pandemic and is working now/has a job	1.566* (0.39)	[0.956,2.567]	1.306 (0.36)	[0.756,2.255]	1.401 (0.56)	[0.638,3.075]	0.591 (0.34)	[0.194,1.796]
Worked before the pandemic and is not working	2.787***(0.85)	[1.534,5.063]	2.312** (0.95)	[1.037,5.156]	5.434***(2.55)	[2.167,13.626]	1.693 (1.03)	[0.513,5.582]

For peer review only

	Ethiopia		India		Peru		Vietnam	
	Odds Ratio	95% CI	Odds Ratio	95% CI	Odds Ratio	95% CI	Odds Ratio	95% CI
now/does not have a job								
Past protective/risk factors								
Participant has long-term health problem, 2016 (Round 5)	1.134 (0.33)	[0.637,2.019]	1.583* (0.40)	[0.960,2.612]	2.591***(0.91)	[1.298,5.171]	1.647 (0.54)	[0.862,3.150]
Total parent-child relationship score, 2012/2016 (Round 4/5)	0.957 (0.03)	[0.899,1.018]	0.939* (0.03)	[0.875,1.007]	0.997 (0.03)	[0.940,1.058]	1.118* (0.07)	[0.989,1.265]
Total peer-child relationship score, 2016 (Round 5)	0.948 (0.03)	[0.887,1.014]	1.041 (0.04)	[0.963,1.124]	1.011 (0.04)	[0.938,1.089]	0.858* (0.08)	[0.720,1.023]
Middle/Top wealth tercile R5, 2016 (Round 5)	1.033 (0.23)	[0.670,1.594]	0.871 (0.20)	[0.551,1.376]	0.635 (0.20)	[0.342,1.180]	0.488** 0.16)	[0.260,0.914]
Proxy baseline information								
Emotional problem scale (EPS) score, 2009 (Round 3)	1.074* (0.04)	[0.994,1.161]	1.007 (0.05)	[0.910,1.114]	1.222*** 0.07)	[1.093,1.366]	1.072 (0.07)	[0.940,1.221]
Subjective well-being, 2009 (Round 3)	0.979 (0.06)	[0.872,1.100]	1.131** (0.07)	[1.010,1.267]	0.924 (0.07)	[0.800,1.068]	0.975 (0.08)	[0.827,1.148]
N	650		836		391		700	

*Note: Odds ratios are unadjusted odds ratios. Robust standard errors in parenthesis, *** significant at 1%, ** significant at 5%, * significant at 10%. Base categories are as follows: Male, Rural, Believe they are at no/low risk, Did not leave the house at all during the past 7 days, Did not spend more time taking care of children, Did not spend more time on household chores, Did not spend more time working in the family business, Did not face new health expenses, Did not suffer a shock, Did not work at all in the past 12 month OR worked during the pandemic but not before and is not working now, Does not have long-term health condition, Lowest wealth tercile. All time-variant variables are measured in 2020 unless otherwise specified.*

Supplementary Table 9. Logistic regression results: Symptoms of at least mild depression (Older Cohort only)

Depression OC	Ethiopia		India		Peru		Vietnam	
	Odds Ratio	95% CI	Odds Ratio	95% CI	Odds Ratio	95% CI	Odds Ratio	95% CI
Structural factors								
Age in months	1.018 (0.03)	[0.962,1.077]	1.065**(0.03)	[1.006,1.127]	0.984 (0.03)	[0.927,1.044]	0.985 (0.04)	[0.915,1.060]
Female	0.834 (0.30)	[0.413,1.683]	1.301 (0.41)	[0.705,2.403]	1.266 (0.35)	[0.734,2.185]	1.093 (0.37)	[0.567,2.107]
Urban	1.395 (0.36)	[0.846,2.298]	0.549**(0.16)	[0.313,0.961]	1.162 (0.50)	[0.496,2.722]	0.512**(0.17)	[0.266,0.988]
COVID-19 related changes/behaviours								
Risk perception: believe they are at medium/high risk	1.595* (0.43)	[0.942,2.703]	0.956 (0.22)	[0.607,1.505]	1.610* 0.41)	[0.980,2.646]	1.672 (0.58)	[0.851,3.287]

For peer review only

Depression	OC	Ethiopia		India		Peru		Vietnam	
		Odds Ratio	95% CI	Odds Ratio	95% CI	Odds Ratio	95% CI	Odds Ratio	95% CI
	Left house for any reason in the past 7 days	1.216 (0.69)	[0.402,3.672]	1.780 (0.63)	[0.890,3.559]	1.778 (0.78)	[0.753,4.195]	0.287***(0.14)	[0.113,0.731]
	Difference subjective well-being between Round 5 (2016) and phone survey (2020)	1.221***(0.08)	[1.082,1.377]	1.175***(0.07)	[1.043,1.324]	1.076 (0.08)	[0.926,1.250]	1.129 (0.12)	[0.915,1.393]
Change in responsibilities:									
	Spend more time taking care of children	0.517** (0.16)	[0.279,0.959]	1.135 (0.33)	[0.637,2.023]	0.707 (0.20)	[0.401,1.247]	1.252 (0.50)	[0.571,2.746]
	Spend more time on household chores	1.457 (0.54)	[0.708,3.002]	0.858 (0.24)	[0.499,1.476]	1.026 (0.33)	[0.543,1.940]	0.912 (0.33)	[0.448,1.858]
	Spend more time working in the family business	1.215 (0.42)	[0.614,2.407]	1.258 (0.73)	[0.402,3.938]	1.534 (0.53)	[0.779,3.022]	1.888 (0.87)	[0.763,4.673]
Economic shocks									
	Faced with new health expenses	1.278 (0.43)	[0.657,2.487]	1.821** (0.48)	[1.089,3.045]	1.226 (0.31)	[0.745,2.017]	0.681 (0.39)	[0.223,2.079]
	Experienced adversity but did not reduce food consumption	1.566 (0.69)	[0.661,3.713]	1.027 (1.15)	[0.114,9.237]	0.463 (0.57)	[0.042,5.082]	1.513 (0.55)	[0.744,3.074]
	Reduced food consumption as response to experienced adversity	4.926***(2.50)	[1.825,13.296]	0.520 (0.66)	[0.042,6.360]	0.363 (0.48)	[0.027,4.887]	1.005 (0.51)	[0.374,2.699]
Changes in employment status									
	Did not work before the pandemic, but is working now[3]	3.429***(1.59)	[1.382,8.507]	0.270* (0.20)	[0.061,1.185]	1.044 (0.69)	[0.285,3.829]	1.000 (.)	[1.000,1.000]
	Worked before the pandemic and is working now/has a job	1.588 (0.45)	[0.915,2.758]	0.740 (0.21)	[0.422,1.297]	0.943 (0.43)	[0.387,2.299]	0.634 (0.37)	[0.203,1.978]
	Worked before the pandemic and is not working now/does not have a job	2.360** (0.87)	[1.145,4.865]	1.267 (0.55)	[0.541,2.968]	1.986 (1.00)	[0.741,5.323]	1.747 (1.07)	[0.523,5.832]
<u>*Past protective/risk factors*</u>									
	Participant has long-term health problem, 2016 (Round 5)	1.129 (0.40)	[0.562,2.268]	1.636* (0.46)	[0.941,2.844]	2.246**(0.74)	[1.176,4.291]	1.036 (0.35)	[0.530,2.025]

For peer review only

Depression	OC	Ethiopia		India		Peru		Vietnam	
		Odds Ratio	95% CI	Odds Ratio	95% CI	Odds Ratio	95% CI	Odds Ratio	95% CI
	Total parent-child relationship score, 2012/2016 (Round 4/5)	1.003 (0.04)	[0.934,1.078]	0.953 (0.04)	[0.877,1.035]	1.010 (0.03)	[0.944,1.080]	1.059 (0.06)	[0.950,1.180]
	Total peer-child relationship score, 2016 (Round 5)	1.049 (0.04)	[0.971,1.133]	1.041 (0.04)	[0.963,1.125]	0.943 (0.04)	[0.868,1.026]	1.001 (0.08)	[0.852,1.177]
	Middle/Top wealth tercile R5, 2016 (Round 5)	0.732 (0.19)	[0.445,1.205]	0.867 (0.22)	[0.522,1.440]	2.247**(0.77)	[1.144,4.414]	0.840 (0.27)	[0.445,1.585]
Proxy baseline information									
	Emotional problem scale (EPS) score, 2009 (Round 3)	1.090* (0.05)	[0.995,1.195]	1.101* (0.06)	[0.991,1.223]	1.129**(0.07)	[1.003,1.270]	1.114 (0.08)	[0.973,1.276]
	Subjective well-being, 2009 (Round 3)	0.894 (0.06)	[0.782,1.023]	1.077 (0.07)	[0.952,1.219]	0.869* (0.07)	[0.736,1.026]	0.952 (0.08)	[0.805,1.126]
	N	650		836		391		683	

*Note: Odds ratios are unadjusted odds ratios. Robust standard errors in parenthesis, *** significant at 1%, ** significant at 5%, * significant at 10%. Base categories are as follows: Male, Rural, Believe they are at no/low risk, Did not leave the house at all during the past 7 days, Did not spend more time taking care of children, Did not spend more time on household chores, Did not spend more time working in the family business, Did not face new health expenses, Did not suffer a shock, Did not work at all in the past 12 month OR worked during the pandemic but not before and is not working now, Does not have long-term health condition, Lowest wealth tercile. All time-variant variables are measured in 2020 unless otherwise specified. [3]In Vietnam only 17 people did not work before the pandemic but are working now. None showed depressive symptoms. As it predicts failure perfectly it drops out of the regressions.*

For peer review only

Supplementary Table 10. Ethiopia: Means of continuous control variables by at least mild anxiety/depression

		All	at least mild anxiety	no anxiety	p-value	at least mild depression	no depression	p-value
Structural factors								
	Age in months	250.86	264.14	247.97	0.000	255.55	250.00	0.014
COVID-19 related changes/behaviours								
	Difference in subjective well-being between Round 5 (2016) and phone survey (2020)	0.96	1.15	0.92	0.052	1.26	0.91	0.005
Past protective/risk factors								
	Total parent-child relationship score, 2012/2016 (Round 4/5)	26.26	25.98	26.32	0.036	26.20	26.27	0.686
	Total peer-child relationship score, 2016 (Round 5)	24.13	23.51	24.26	0.000	23.58	24.23	0.000
Proxy baseline information								
	Emotional problem scale (EPS) score, 2009 (Round 3)²	2.78	3.00	2.70	0.173	3.13	2.71	0.096
	Subjective well-being, 2009 (Round 3)²	5.22	5.18	5.23	0.649	5.24	5.22	0.896

Note: *Results are for the combined Younger Cohort/ Older Cohort sample unless indicated otherwise, ² refers to Older Cohort (25-26) only.*

For peer review only

Supplementary Table 11. India: Means of continuous control variables by at least mild anxiety/depression

		All	at least mild anxiety	no anxiety	p-value	at least mild depression	no depression	p-value
Structural factors								
	Age in months	253.30	261.16	252.32	0.000	258.12	252.77	0.037
COVID-19 related changes/behaviours								
	Difference in subjective well-being between Round 5 (2016) and phone survey (2020)	0.49	0.77	0.45	0.009	0.86	0.45	0.001
Past protective/risk factors								
	Total parent-child relationship score, 2012/2016 (Round 4/5)	27.63	26.94	27.72	0.000	26.84	27.72	0.000
	Total peer-child relationship score, 2016 (Round 5)	25.02	24.97	25.03	0.743	24.91	25.03	0.468
Proxy baseline information								
	Emotional problem scale (EPS) score, 2009 (Round 3)²	3.57	3.93	3.51	0.067	4.14	3.49	0.010
	Subjective well-being, 2009 (Round 3)²	5.00	5.29	4.96	0.013	4.97	5.00	0.850

Note: Results are for the combined Younger Cohort/ Older Cohort sample unless indicated otherwise, ² refers to Older Cohort (25-26) only.

For peer review only

Supplementary Table 12. Peru: Means of continuous control variables by at least mild anxiety/depression

		All	at least mild anxiety	no anxiety	p-value	at least mild depression	no depression	p-value
Structural factors								
	Age in months	244.23	245.19	243.57	0.315	242.56	245.00	0.151
COVID-19 related changes/behaviours								
	Difference in subjective well-being in Round 5 (2016) and phone survey (2020)	0.52	0.74	0.38	0.000	0.70	0.44	0.013
Past protective/risk factors								
	Total parent-child relationship score, 2012/2016 (Round 4/5)	24.86	24.60	25.04	0.009	24.28	25.12	0.000
	Total peer-child relationship score, 2016 (Round 5)	23.41	23.40	23.42	0.862	23.41	23.42	0.954
Proxy baseline information								
	Emotional problem scale (EPS) score, 2009 (Round 3)[2]	4.27	4.87	3.81	0.000	4.85	4.03	0.002
	Subjective well-being, 2009 (Round 3)[2]	6.82	6.72	6.89	0.073	6.72	6.87	0.135

Note: Results are for the combined Younger Cohort/ Older Cohort sample unless indicated otherwise, ² refers to Older Cohort (25-26) only.

For peer review only

Supplementary Table 13. Vietnam: Means of continuous control variables by at least mild anxiety/depression

		All	at least mild anxiety	no anxiety	p-value	at least mild depression	no depression	p-value
Structural factors								
	Age in months	252.95	248.28	253.43	0.067	246.49	253.63	0.010
COVID-19 related changes/behaviours								
	Difference in subjective well-being in Round 5 (2016) and phone survey (2020)	-0.39	0.14	-0.45	0.000	0.11	-0.45	0.000
Past protective/risk factors								
	Total parent-child relationship score, 2012/2016 (Round 4/5)	25.74	26.11	25.70	0.073	25.72	25.74	0.930
	Total peer-child relationship score, 2016 (Round 5)	22.57	22.40	22.59	0.230	22.52	22.58	0.704
Proxy baseline information								
	Emotional problem scale (EPS) score, 2009 (Round 3)[2]	3.68	3.96	3.65	0.314	4.18	3.64	0.086
	Subjective well-being, 2009 (Round 3)[2]	5.84	5.73	5.86	0.383	5.85	5.84	0.943

Note: *Results are for the combined Younger Cohort/ Older Cohort sample unless indicated otherwise, [2] refers to Older Cohort (25-26) only.*

For peer review only

Supplementary Table 14. Mental health outcomes by control variables (Ethiopia & India)

	Ethiopia				India			
	% at least mild Anxiety	p-value	% at least mild Depression	p-value	% at least mild Anxiety	p-value	% at least mild Depression	p-value
COVID-19 related changes/behaviours								
Risk perception: believe they are at no/low risk	20.30	0.048	20.75	0.000	10.10	0.092	10.10	0.735
Risk perception: believe they are at medium/high risk	16.79		13.09		12.17		9.70	
Did not leave house for any reason in the past 7 days	22.50	0.173	18.33	0.367	8.46	0.030	5.51	0.000
Left house for any reason in the past 7 days	17.60		15.27		11.74		11.07	
Change in responsibilities:								
Did not spend more time taking care of children	18.06	0.704	15.55	0.823	9.01	0.000	8.82	0.000
Spend more time taking care of children	17.38		15.17		20.21		14.79	
Did not spend more time on household chores	17.83	0.963	14.64	0.273	10.58	0.438	9.72	0.742
Spend more time on household chores	17.91		16.34		11.53		10.10	
Did not spend more time working in the family business	18.26	0.209	15.63	0.516	10.90	0.204	9.90	0.924
Spend more time working in the family business	15.19		14.13		14.81		10.19	
Economic shocks								
Was not faced with new health expenses	17.43	0.115	15.75	0.230	10.91	0.827	8.57	0.041
Faced with new health expenses	21.72		12.67		11.18		10.97	
Did not experience adversity	6.86	0.000	4.65	0.000	13.33	0.381	11.11	0.956
Experienced adversity but did not reduce food consumption	17.54		15.59		10.82		9.88	
Reduced food consumption as response to experienced adversity	36.39		31.29		14.29		10.20	
Changes in employment status								
Did not work at all in the past 12 month OR worked during the pandemic but not before the pandemic (and is not working now)	13.79	0.000	13.10	0.000	9.22	0.000	9.03	0.059
Did not work before the pandemic, but is working now	26.17		25.50		9.07		8.37	
Worked before the pandemic and is working now/has a job	18.34		14.91		12.89		10.86	
Worked before the pandemic and is not working now/does not have a job	31.49		22.65		19.67		15.57	
Educational disruption								
Never attended school or not enrolled in full-time education/not planning to enrol in learning activities[1]	18.25	0.212	13.45	0.056	13.01	0.000	11.00	0.012
Enrolled in/Planning to enrol in full time education and	16.58		17.41		10.69		10.40	

For peer review only

For peer review only - http://bmjopen.bmj.com/site/about/guidelines.xhtml

not participating in learning activities[1]								
Enrolled in/Planning to enrol in full time education and participating in learning activities[1]	21.13		15.47		6.57		6.57	
Past protective/risk factors								
Participant does not have a long-term health problem, 2016 (Round 5)	17.13	0.004	15.42	0.940	10.47	0.007	9.31	0.004
Participant has long-term health problem, 2016 (Round 5)	25.52		15.63		15.61		14.62	

Notes: If any missing answers to questions then the whole score is set to missing. p-value indicates significance of t-tests between dummy variables and o F-tests in case of categorical variables. Results are for the combined Younger Cohort/ Older Cohort sample unless indicated otherwise, [1] refers to Younger Cohort (18-19) only.

Supplementary Table 15. Mental health outcomes by control variables (Peru & Vietnam)

	**Peru**				**Vietnam**			
	**% at least mild Anxiety**	p-value	**% at least mild Depression**	p-value	**% at least mild Anxiety**	p-value	**% at least mild Depression**	p-value
COVID-19 related changes/behaviours								
Risk perception: believe they are at no/low risk	35.34	0.000	25.84	0.000	9.31	0.967	9.31	0.557
Risk perception: believe they are at medium/high risk	45.51		36.43		9.37		10.18	
Did not leave house for any reason in the past 7 days	42.82	0.393	33.33	0.421	9.54	0.892	11.66	0.184
Left house for any reason in the past 7 days	40.38		31.16		9.29		9.19	
Change in responsibilities:								
Did not spend more time taking care of children	36.83	0.000	29.57	0.009	8.19	0.001	8.19	0.000
Spend more time taking care of children	48.61		35.45		13.12		13.88	
Did not spend more time on household chores	33.96	0.001	28.77	0.157	7.44	0.003	6.46	0.000
Spend more time on household chores	42.86		32.40		11.09		12.36	
Did not spend more time working in the family business	40.09	0.105	30.92	0.141	8.46	0.000	8.60	0.000
Spend more time working in the family business	45.23		35.34		16.40		16.80	
Economic shocks								
Was not faced with new health expenses	36.12	0.000	27.24	0.000	8.99	0.041	9.27	0.171
Faced with new health expenses	52.15		41.94		14.00		12.67	

For peer review only

For peer review only - http://bmjopen.bmj.com/site/about/guidelines.xhtml

	Peru				Vietnam			
	**% at least mild Anxiety**	p-value	**% at least mild Depression**	p-value	**% at least mild Anxiety**	p-value	**% at least mild Depression**	p-value
Did not experience adversity	16.33	0.002	14.29	0.004	6.49	0.000	6.29	0.000
Experienced adversity but did not reduce food consumption	41.32		31.47		10.80		11.11	
Reduced food consumption as response to experienced adversity	44.26		40.16		12.95		13.77	
Changes in employment status								
Did not work at all in the past 12 month OR worked during the pandemic but not before the pandemic (and is not working now)	41.41	0.176	33.26	0.003	8.53	0.269	9.83	0.002
Did not work before the pandemic, but is working now	37.77		32.19		10.97		8.39	
Worked before the pandemic and is working now/has a job	39.57		27.89		8.81		8.11	
Worked before the pandemic and is not working now/does not have a job	45.91		38.99		12.01		15.26	
Educational disruption								
Never attended school or not enrolled in full-time education/not planning to enrol in learning activities[1]	40.74	0.611	30.74	0.490	9.95	0.374	8.91	0.387
Enrolled in/Planning to enrol in full time education and not participating in learning activities[1]	32.14		28.57		8.96		9.43	
Enrolled in/Planning to enrol in full time education and participating in learning activities[1]	41.47		33.33		8.04		10.82	
<u>*Past protective/risk factors*</u>								
Participant does not have a long-term health problem, 2016 (Round 5)	38.71	0.000	29.85	0.000	9.04	0.248	9.14	0.151
Participant has long-term health problem, 2016 (Round 5)	55.65		43.51		11.04		11.66	

Notes: If any missing answers to questions then the whole score is set to missing. p-value indicates significance of t-tests between dummy variables and o F-tests in case of categorical variables. Results are for the combined Younger Cohort/ Older Cohort sample unless indicated otherwise, [1] refers to Younger Cohort (18-19) only.

For peer review only

Supplementary Table 16. Logistic regression results: Symptoms of at least mild anxiety/depression by gender (Ethiopia)

	At least mild anxiety				At least mild depression			
	Male		Female		Male		Female	
	Odds Ratio	95% CI	Odds Ratio	95% CI	Odds Ratio	95% CI	Odds Ratio	95% CI
Structural factors								
Age in months	1.012*** (0.00)	[1.007,1.016]	1.003 (0.00)	[0.999,1.008]	1.007*** (0.00)	[1.002,1.012]	0.996* (0.00)	[0.991,1.001]
Urban	1.155 (0.22)	[0.799,1.670]	1.523** (0.32)	[1.003,2.312]	1.307 (0.26)	[0.880,1.942]	1.440* (0.31)	[0.938,2.210]
COVID-19 related changes/behaviours								
Risk perception: believe they are at medium/high risk	0.807 (0.14)	[0.569,1.146]	0.825 (0.16)	[0.562,1.211]	0.576*** (0.10)	[0.403,0.824]	0.659** (0.13)	[0.452,0.961]
Left house for any reason in the past 7 days	0.593 (0.29)	[0.229,1.533]	0.847 (0.25)	[0.480,1.494]	0.556 (0.25)	[0.233,1.327]	1.126 (0.35)	[0.614,2.064]
Difference in subjective well-being between Round 5 (2016) and phone survey (2020)	1.097** (0.04)	[1.016,1.185]	1.052 (0.05)	[0.967,1.146]	1.084* (0.05)	[0.999,1.177]	1.114** (0.05)	[1.019,1.218]
Change in responsibilities:								
Spend more time taking care of children	1.262 (0.34)	[0.747,2.132]	0.749 (0.14)	[0.513,1.093]	0.791 (0.21)	[0.468,1.339]	0.957 (0.20)	[0.640,1.433]
Spend more time on household chores	0.869 (0.20)	[0.558,1.353]	1.447 (0.33)	[0.929,2.252]	1.181 (0.25)	[0.776,1.797]	1.162 (0.27)	[0.731,1.845]
Spend more time working in the family business	0.750 (0.20)	[0.448,1.256]	0.713 (0.24)	[0.367,1.384]	0.643 (0.19)	[0.361,1.144]	1.498 (0.47)	[0.814,2.755]
Economic shocks								
Faced with new health expenses	0.733 (0.21)	[0.419,1.281]	1.115 (0.30)	[0.660,1.886]	0.438*** (0.13)	[0.241,0.797]	0.592* (0.19)	[0.319,1.096]
Experienced adversity but did not reduce food consumption	1.889** (0.53)	[1.091,3.270]	3.063*** (1.00)	[1.619,5.793]	3.658*** (1.35)	[1.776,7.537]	3.739*** (1.28)	[1.908,7.326]
Reduced food consumption as response to experienced adversity	5.891*** (1.92)	[3.107,11.172]	8.642*** (3.16)	[4.222,17.690]	12.987*** (5.25)	[5.884,28.663]	8.585*** (3.32)	[4.026,18.308]
Changes in employment status								
Did not work before the pandemic, but is working now	2.623*** (0.88)	[1.356,5.075]	2.853*** (0.97)	[1.470,5.538]	2.426*** (0.79)	[1.283,4.591]	2.754*** (0.99)	[1.357,5.590]
Worked before the pandemic and is working now/has a job	1.335 (0.30)	[0.859,2.074]	1.981*** (0.46)	[1.260,3.115]	1.057 (0.23)	[0.684,1.634]	1.744** (0.41)	[1.096,2.776]

For peer review only - http://bmjopen.bmj.com/site/about/guidelines.xhtml

	At least mild anxiety				At least mild depression			
	Male		Female		Male		Female	
	Odds Ratio	95% CI	Odds Ratio	95% CI	Odds Ratio	95% CI	Odds Ratio	95% CI
Worked before the pandemic and is not working now/does not have a job	2.196*** (0.61)	[1.268,3.801]	2.239*** (0.64)	[1.279,3.918]	1.020 (0.33)	[0.540,1.927]	2.647*** (0.79)	[1.476,4.746]
Past protective/risk factors								
Participant has long-term health problem, 2016 (Round 5)	1.848** (0.53)	[1.054,3.241]	1.204 (0.29)	[0.745,1.944]	0.919 (0.33)	[0.450,1.875]	0.998 (0.28)	[0.580,1.716]
Total parent-child relationship score, 2012/2016 (Round 4/5)	0.968 (0.03)	[0.914,1.026]	0.962 (0.03)	[0.902,1.026]	1.027 (0.03)	[0.965,1.092]	0.972 (0.03)	[0.908,1.039]
Total peer-child relationship score, 2016 (Round 5)	0.935** (0.03)	[0.877,0.998]	0.944* (0.03)	[0.890,1.002]	0.948 (0.03)	[0.884,1.017]	0.932** (0.03)	[0.875,0.993]
Middle/Top wealth tercile R5, 2016 (Round 5)	1.199 (0.24)	[0.811,1.773]	0.985 (0.21)	[0.643,1.511]	0.882 (0.18)	[0.587,1.327]	0.844 (0.19)	[0.539,1.321]
N	1173		1010		1173		1010	

*Note: Odds ratios are unadjusted odds ratios. Robust standard errors in parenthesis, *** significant at 1%, ** significant at 5%, * significant at 10%. Base categories are as follows: Rural, Believe they are at no/low risk, Did not leave the house at all during the past 7 days, Did not spend more time taking care of children, Did not spend more time on household chores, Did not spend more time working in the family business, Did not face new health expenses, Did not suffer a shock, Did not work at all in the past 12 month OR worked during the pandemic but not before and is not working now, Does not have long-term health condition, Lowest wealth tercile. All time-variant variables are measured in 2020 unless otherwise specified. Results are for the combined Younger Cohort/ Older Cohort sample.*

Supplementary Table 17. Logistic regression results: Symptoms of at least mild anxiety/depression by gender (India)

	At least mild anxiety				At least mild depression			
	Male		Female		Male		Female	
	Odds Ratio	95% CI	Odds Ratio	95% CI	Odds Ratio	95% CI	Odds Ratio	95% CI
Structural factors								
Age in months	0.999 (0.00)	[0.994,1.004]	1.003 (0.00)	[0.998,1.008]	0.998 (0.00)	[0.993,1.003]	1.002 (0.00)	[0.996,1.008]
Urban	0.428*** (0.12)	[0.252,0.728]	1.564** (0.35)	[1.012,2.417]	0.502*** (0.12)	[0.311,0.808]	1.113 (0.28)	[0.679,1.824]
COVID-19 related changes/behaviours								
Risk perception: believe they are at medium/high risk	1.520** (0.30)	[1.038,2.224]	1.149 (0.22)	[0.795,1.661]	1.048 (0.20)	[0.722,1.522]	0.927 (0.19)	[0.616,1.395]
Left house for any reason in the past 7 days	0.744 (0.28)	[0.356,1.555]	1.606** (0.36)	[1.040,2.482]	1.063 (0.43)	[0.482,2.342]	2.810*** (0.77)	[1.642,4.810]

	At least mild anxiety				At least mild depression			
	Male		Female		Male		Female	
	Odds Ratio	95% CI	Odds Ratio	95% CI	Odds Ratio	95% CI	Odds Ratio	95% CI
Difference in subjective well-being between Round 5 (2016) and phone survey (2020)	0.999 (0.05)	[0.904,1.105]	1.245*** (0.07)	[1.121,1.383]	1.041 (0.05)	[0.950,1.141]	1.248*** (0.07)	[1.117,1.395]
Change in responsibilities:								
Spend more time taking care of children	1.708* (0.50)	[0.967,3.015]	2.455*** (0.54)	[1.595,3.780]	1.327 (0.41)	[0.721,2.443]	1.668** (0.43)	[1.007,2.761]
Spend more time on household chores	1.356 (0.27)	[0.923,1.993]	0.440*** (0.09)	[0.297,0.651]	1.337 (0.26)	[0.915,1.955]	0.576** (0.13)	[0.376,0.883]
Spend more time working in the family business	1.944* (0.71)	[0.953,3.967]	1.210 (0.52)	[0.520,2.816]	1.100 (0.45)	[0.493,2.457]	1.043 (0.58)	[0.348,3.122]
Economic shocks								
Faced with new health expenses	0.786 (0.16)	[0.532,1.161]	1.349 (0.26)	[0.924,1.968]	1.203 (0.25)	[0.799,1.810]	1.477* (0.32)	[0.969,2.250]
Experienced adversity but did not reduce food consumption	0.894 (0.76)	[0.169,4.723]	0.500 (0.30)	[0.155,1.609]	1.749 (1.91)	[0.207,14.799]	0.384* (0.21)	[0.130,1.137]
Reduced food consumption as response to experienced adversity	1.272 (1.17)	[0.209,7.752]	0.691 (0.48)	[0.177,2.697]	1.651 (1.92)	[0.169,16.089]	0.398 (0.28)	[0.099,1.608]
Changes in employment status								
Did not work before the pandemic, but is working now	0.629 (0.21)	[0.326,1.213]	1.267 (0.40)	[0.687,2.334]	0.555* (0.18)	[0.294,1.051]	0.887 (0.29)	[0.472,1.669]
Worked before the pandemic and is working now/has a job	0.982 (0.28)	[0.562,1.718]	1.504* (0.33)	[0.978,2.314]	0.980 (0.25)	[0.591,1.626]	0.802 (0.21)	[0.485,1.328]
Worked before the pandemic and is not working now/does not have a job	2.812** (1.14)	[1.266,6.245]	2.286** (0.83)	[1.121,4.660]	2.908*** (1.12)	[1.367,6.188]	0.836 (0.40)	[0.325,2.148]
Past protective/risk factors								
Participant has long-term health problem, 2016 (Round 5)	1.310 (0.38)	[0.746,2.299]	1.466 (0.35)	[0.924,2.326]	1.353 (0.39)	[0.773,2.366]	1.785** (0.44)	[1.105,2.885]
Total parent-child relationship score, 2012/2016 (Round 4/5)	0.908*** (0.03)	[0.845,0.977]	0.934** (0.03)	[0.880,0.991]	0.908*** (0.03)	[0.846,0.973]	0.909*** (0.03)	[0.848,0.974]
Total peer-child relationship score, 2016 (Round 5)	1.039 (0.04)	[0.965,1.119]	1.014 (0.04)	[0.944,1.089]	1.040 (0.04)	[0.961,1.125]	0.994 (0.04)	[0.924,1.069]
Middle/Top wealth tercile R5, 2016 (Round 5)	0.804 (0.17)	[0.534,1.210]	0.764 (0.16)	[0.504,1.159]	1.324 (0.27)	[0.881,1.989]	0.877 (0.20)	[0.559,1.375]
N	1369		1253		1369		1253	

*Note: Odds ratios are unadjusted odds ratios. Robust standard errors in parenthesis, *** significant at 1%, ** significant at 5%, * significant at 10%. Base categories are as follows: Rural, Believe they are at no/low risk, Did not leave the house at all during the past 7 days, Did not spend more time taking care of children, Did not spend more time on household chores, Did not spend more time working in the family business, Did not face new health expenses, Did not suffer a shock, Did not work at all in the past 12 month OR worked during the pandemic but not before and is not working now, Does not have long-term health condition, Lowest wealth tercile. All time-variant variables are measured in 2020 unless otherwise specified. Results are for the combined Younger Cohort/ Older Cohort sample.*

For peer review only - http://bmjopen.bmj.com/site/about/guidelines.xhtml

Supplementary Table 18. Logistic regression results: Symptoms of at least mild anxiety/depression by gender (Peru)

	At least mild anxiety				At least mild depression			
	Male		Female		Male		Female	
	Odds Ratio	95% CI	Odds Ratio	95% CI	Odds Ratio	95% CI	Odds Ratio	95% CI
Structural factors								
Age in months	1.001 (0.00)	[0.997,1.005]	0.998 (0.00)	[0.994,1.002]	0.996* (0.00)	[0.991,1.001]	0.996* (0.00)	[0.992,1.000]
Urban	1.245 (0.27)	[0.817,1.898]	1.610** (0.34)	[1.070,2.423]	0.978 (0.21)	[0.639,1.498]	1.222 (0.27)	[0.790,1.889]
COVID-19 related changes/behaviours								
Risk perception: believe they are at medium/high risk	1.326* (0.19)	[0.994,1.767]	1.585*** (0.22)	[1.204,2.086]	1.513*** (0.23)	[1.117,2.051]	1.657*** (0.24)	[1.241,2.212]
Left house for any reason in the past 7 days	1.324 (0.28)	[0.878,1.996]	0.848 (0.14)	[0.609,1.181]	1.052 (0.22)	[0.697,1.588]	1.067 (0.19)	[0.756,1.505]
Difference in subjective well-being between Round 5 (2016) and phone survey (2020)	1.182*** (0.05)	[1.097,1.274]	1.064* (0.03)	[0.998,1.134]	1.131*** (0.05)	[1.043,1.227]	1.047 (0.04)	[0.978,1.121]
Change in responsibilities:								
Spend more time taking care of children	1.567*** (0.26)	[1.134,2.165]	1.285* (0.18)	[0.973,1.698]	1.529** (0.26)	[1.097,2.131]	0.932 (0.14)	[0.697,1.246]
Spend more time on household chores	1.220 (0.21)	[0.873,1.704]	1.293 (0.25)	[0.882,1.895]	1.039 (0.18)	[0.734,1.471]	1.105 (0.22)	[0.742,1.644]
Spend more time working in the family business	1.299 (0.25)	[0.896,1.885]	1.335 (0.29)	[0.871,2.046]	1.258 (0.25)	[0.848,1.865]	1.362 (0.30)	[0.883,2.101]
Economic shocks								
Faced with new health expenses	1.983*** (0.32)	[1.451,2.710]	1.532*** (0.23)	[1.148,2.044]	1.735*** (0.29)	[1.257,2.394]	1.757*** (0.26)	[1.308,2.361]
Experienced adversity but did not reduce food consumption	5.087** (3.94)	[1.114,23.224]	1.609 (0.88)	[0.548,4.728]	4.172* (3.08)	[0.983,17.705]	1.056 (0.63)	[0.330,3.378]
Reduced food consumption as response to experienced adversity	5.843** (4.83)	[1.157,29.524]	1.288 (0.77)	[0.397,4.178]	5.041** (4.02)	[1.057,24.045]	1.354 (0.87)	[0.383,4.782]
Changes in employment status								
Did not work before the pandemic, but is working now	0.684 (0.21)	[0.379,1.235]	0.945 (0.23)	[0.591,1.510]	1.065 (0.32)	[0.593,1.910]	0.790 (0.19)	[0.491,1.271]
Worked before the pandemic and is working now/has a job	0.958 (0.22)	[0.608,1.509]	1.049 (0.19)	[0.737,1.493]	0.899 (0.22)	[0.563,1.437]	0.799 (0.15)	[0.551,1.158]
Worked before the pandemic and is not working now/does not have a job	1.029 (0.27)	[0.616,1.719]	1.326 (0.26)	[0.900,1.952]	1.385 (0.37)	[0.824,2.329]	1.193 (0.25)	[0.797,1.786]

For peer review only

	At least mild anxiety				At least mild depression			
	Male		Female		Male		Female	
	Odds Ratio	95% CI	Odds Ratio	95% CI	Odds Ratio	95% CI	Odds Ratio	95% CI
Past protective/risk factors								
Participant has long-term health problem, 2016 (Round 5)	1.794** (0.41)	[1.142,2.818]	1.937*** (0.38)	[1.318,2.848]	1.662** (0.39)	[1.047,2.640]	1.564** (0.29)	[1.081,2.262]
Total parent-child relationship score, 2012/2016 (Round 4/5)	0.974 (0.02)	[0.931,1.020]	0.982 (0.02)	[0.947,1.019]	0.944** (0.02)	[0.898,0.992]	0.936*** (0.02)	[0.900,0.973]
Total peer-child relationship score, 2016 (Round 5)	1.010 (0.03)	[0.958,1.065]	0.990 (0.02)	[0.951,1.031]	1.002 (0.03)	[0.946,1.063]	1.011 (0.02)	[0.968,1.055]
Middle/Top wealth tercile R5, 2016 (Round 5)	0.777 (0.14)	[0.548,1.104]	1.257 (0.22)	[0.890,1.774]	1.137 (0.21)	[0.795,1.627]	1.294 (0.24)	[0.894,1.873]
N	957		930		957		930	

*Note: Odds ratios are unadjusted odds ratios. Robust standard errors in parenthesis, *** significant at 1%, ** significant at 5%, * significant at 10%. Base categories are as follows: Rural, Believe they are at no/low risk, Did not leave the house at all during the past 7 days, Did not spend more time taking care of children, Did not spend more time on household chores, Did not spend more time working in the family business, Did not face new health expenses, Did not suffer a shock, Did not work at all in the past 12 month OR worked during the pandemic but not before and is not working now, Does not have long-term health condition, Lowest wealth tercile. All time-variant variables are measured in 2020 unless otherwise specified. Results are for the combined Younger Cohort/ Older Cohort sample.*

Supplementary Table 19. Logistic regression results: Symptoms of at least mild anxiety/depression by gender (Vietnam)

	At least mild anxiety				At least mild depression			
	Male		Female		Male		Female	
	Odds Ratio	95% CI	Odds Ratio	95% CI	Odds Ratio	95% CI	Odds Ratio	95% CI
Structural factors								
Age in months	0.996 (0.00)	[0.990,1.002]	0.994** (0.00)	[0.988,1.000]	0.992** (0.00)	[0.985,0.999]	0.994** (0.00)	[0.988,1.000]
Urban	0.960 (0.22)	[0.608,1.517]	1.358 (0.27)	[0.916,2.014]	0.837 (0.22)	[0.500,1.402]	1.091 (0.22)	[0.740,1.607]
COVID-19 related changes/behaviours								
Risk perception: believe they are at medium/high risk	1.548* (0.41)	[0.921,2.603]	0.816 (0.21)	[0.498,1.337]	2.094** (0.61)	[1.180,3.715]	0.828 (0.19)	[0.525,1.304]
Left house for any reason in the past 7 days	0.666 (0.22)	[0.353,1.254]	1.535 (0.51)	[0.802,2.936]	0.748 (0.25)	[0.388,1.439]	1.225 (0.36)	[0.694,2.164]
Difference in subjective well-being between	1.296*** (0.10)	[1.116,1.506]	1.111* (0.06)	[0.994,1.241]	1.471*** (0.11)	[1.269,1.706]	1.016 (0.05)	[0.914,1.129]

	At least mild anxiety				At least mild depression			
	Male		Female		Male		Female	
	Odds Ratio	95% CI	Odds Ratio	95% CI	Odds Ratio	95% CI	Odds Ratio	95% CI
Round 5 (2016) and phone survey (2020)								
Change in responsibilities:								
Spend more time taking care of children	1.222 (0.37)	[0.673,2.220]	1.520* (0.35)	[0.971,2.381]	1.221 (0.39)	[0.648,2.300]	1.506* (0.34)	[0.970,2.339]
Spend more time on household chores	1.191 (0.27)	[0.757,1.872]	1.191 (0.26)	[0.777,1.825]	1.076 (0.27)	[0.655,1.770]	1.996*** (0.46)	[1.277,3.122]
Spend more time working in the family business	2.394*** (0.75)	[1.292,4.434]	1.624* (0.44)	[0.950,2.777]	2.373*** (0.76)	[1.261,4.463]	1.875** (0.48)	[1.132,3.106]
Economic shocks								
Faced with new health expenses	0.544 (0.29)	[0.191,1.547]	1.757* (0.57)	[0.933,3.309]	1.093 (0.49)	[0.453,2.636]	0.721 (0.27)	[0.345,1.508]
Experienced adversity but did not reduce food consumption	2.494*** (0.70)	[1.440,4.320]	1.230 (0.28)	[0.787,1.925]	2.430*** (0.71)	[1.370,4.309]	1.416 (0.33)	[0.902,2.224]
Reduced food consumption as response to experienced adversity	2.367** (0.86)	[1.159,4.833]	1.357 (0.38)	[0.785,2.346]	1.883* (0.72)	[0.889,3.987]	1.946** (0.51)	[1.162,3.259]
Changes in employment status								
Did not work before the pandemic, but is working now	1.605 (0.84)	[0.574,4.486]	0.814 (0.34)	[0.357,1.857]	0.254 (0.25)	[0.036,1.782]	0.913 (0.34)	[0.436,1.910]
Worked before the pandemic and is working now/has a job	1.127 (0.42)	[0.545,2.331]	0.948 (0.24)	[0.575,1.565]	1.157 (0.41)	[0.576,2.324]	0.708 (0.17)	[0.441,1.136]
Worked before the pandemic and is not working now/does not have a job	1.798 (0.72)	[0.817,3.958]	1.178 (0.37)	[0.635,2.187]	1.921* (0.75)	[0.898,4.108]	1.456 (0.40)	[0.848,2.502]
Past protective/risk factors								
Participant has long-term health problem, 2016 (Round 5)	1.944** (0.58)	[1.088,3.474]	1.055 (0.29)	[0.612,1.819]	2.231** (0.74)	[1.167,4.266]	1.063 (0.28)	[0.631,1.789]
Total parent-child relationship score, 2012/2016 (Round 4/5)	1.015 (0.04)	[0.940,1.096]	1.056 (0.04)	[0.989,1.127]	0.983 (0.04)	[0.904,1.071]	0.982 (0.03)	[0.925,1.043]
Total peer-child relationship score, 2016	1.014 (0.06)	[0.907,1.133]	0.897** (0.04)	[0.814,0.989]	1.055 (0.07)	[0.928,1.199]	0.961 (0.04)	[0.877,1.052]

For peer review only

	At least mild anxiety				At least mild depression			
	Male		Female		Male		Female	
	**Odds Ratio**	**95% CI**	**Odds Ratio**	**95% CI**	**Odds Ratio**	**95% CI**	**Odds Ratio**	**95% CI**
(Round 5)								
Middle/Top wealth tercile R5, 2016 (Round 5)	0.551** (0.14)	[0.336,0.903]	0.566*** (0.12)	[0.379,0.844]	1.015 (0.28)	[0.588,1.754]	1.130 (0.24)	[0.745,1.713]
N	1111		1185		1111		1185	

*Note: Odds ratios are unadjusted odds ratios. Robust standard errors in parenthesis, *** significant at 1%, ** significant at 5%, * significant at 10%. Base categories are as follows: Rural, Believe they are at no/low risk, Did not leave the house at all during the past 7 days, Did not spend more time taking care of children, Did not spend more time on household chores, Did not spend more time working in the family business, Did not face new health expenses, Did not suffer a shock, Did not work at all in the past 12 month OR worked during the pandemic but not before and is not working now, Does not have long-term health condition, Lowest wealth tercile. All time-variant variables are measured in 2020 unless otherwise specified. Results are for the combined Younger Cohort/ Older Cohort sample.*

For peer review only

Supplementary Table 20. Logistic regression results: Symptoms of at least mild anxiety/depression by gender for the Younger Cohort only (Ethiopia)

	At least mild anxiety				At least mild depression			
	Male		Female		Male		Female	
	Odds Ratio	95% CI	Odds Ratio	95% CI	Odds Ratio	95% CI	Odds Ratio	95% CI
Structural factors								
Age in months	1.011 (0.03)	[0.952,1.074]	0.990 (0.03)	[0.937,1.047]	1.005 (0.03)	[0.946,1.068]	0.997 (0.03)	[0.944,1.054]
Urban	1.421 (0.39)	[0.831,2.430]	1.712* (0.51)	[0.953,3.075]	1.341 (0.37)	[0.783,2.296]	1.634* (0.45)	[0.956,2.792]
COVID-19 related changes/behaviours								
Risk perception: believe they are at medium/high risk	0.550** (0.13)	[0.348,0.869]	0.875 (0.22)	[0.540,1.420]	0.461*** (0.11)	[0.290,0.733]	0.512*** (0.12)	[0.324,0.810]
Left house for any reason in the past 7 days	0.620 (0.29)	[0.249,1.545]	1.140 (0.47)	[0.504,2.579]	0.702 (0.34)	[0.269,1.832]	1.129 (0.42)	[0.546,2.336]
Difference in subjective well-being between Round 5 (2016) and phone survey (2020)	1.076 (0.05)	[0.973,1.189]	1.001 (0.06)	[0.897,1.117]	1.015 (0.06)	[0.910,1.131]	1.083 (0.06)	[0.975,1.204]
Change in responsibilities:								
Spend more time taking care of children	1.481 (0.44)	[0.823,2.666]	0.754 (0.19)	[0.460,1.236]	0.839 (0.25)	[0.463,1.522]	1.224 (0.30)	[0.757,1.979]
Spend more time on household chores	0.780 (0.19)	[0.479,1.271]	1.401 (0.41)	[0.793,2.475]	1.083 (0.26)	[0.671,1.749]	1.132 (0.32)	[0.647,1.979]
Spend more time working in the family business	0.542 (0.23)	[0.239,1.233]	0.842 (0.39)	[0.344,2.064]	0.475 (0.22)	[0.189,1.195]	2.336** (0.88)	[1.120,4.872]
Economic shocks								
Faced with new health expenses	0.673 (0.31)	[0.272,1.666]	0.645 (0.28)	[0.273,1.524]	0.278** (0.17)	[0.083,0.931]	0.199** (0.13)	[0.057,0.695]
Experienced adversity but did not reduce food consumption	3.346*** (1.34)	[1.530,7.320]	3.633*** (1.42)	[1.692,7.801]	6.595*** (3.49)	[2.338,18.604]	3.859*** (1.52)	[1.785,8.340]
Reduced food consumption as response to experienced adversity	6.188*** (2.97)	[2.418,15.835]	9.701*** (4.34)	[4.035,23.323]	19.255*** (11.13)	[6.202,59.780]	8.538*** (3.98)	[3.427,21.271]
Educational disruption								
Enrolled in/Planning to enrol in full time education and not participating in learning activities	1.188 (0.33)	[0.693,2.039]	2.556*** (0.87)	[1.313,4.972]	1.520 (0.45)	[0.845,2.732]	2.033** (0.62)	[1.122,3.683]
Enrolled in/Planning to enrol in full time education and participating in learning activities	0.810 (0.46)	[0.269,2.436]	1.600 (0.79)	[0.608,4.207]	0.735 (0.48)	[0.207,2.613]	1.524 (0.68)	[0.635,3.656]
Past protective/risk factors								
Participant has long-term health problem,	1.185	[0.464,3.028]	1.291	[0.630,2.646]	0.358	[0.086,1.494]	0.621	[0.260,1.483]

	At least mild anxiety				At least mild depression			
	Male		Female		Male		Female	
	Odds Ratio	95% CI	Odds Ratio	95% CI	Odds Ratio	95% CI	Odds Ratio	95% CI
2016 (Round 5)	(0.57)		(0.47)		(0.26)		(0.28)	
Total parent-child relationship score, 2012/2016 (Round 4/5)	0.971 (0.04)	[0.890,1.059]	0.943 (0.04)	[0.858,1.035]	1.056 (0.04)	[0.974,1.145]	0.970 (0.04)	[0.889,1.059]
Total peer-child relationship score, 2016 (Round 5)	0.934 (0.05)	[0.848,1.028]	0.970 (0.04)	[0.893,1.054]	0.892** (0.05)	[0.808,0.985]	0.915** (0.04)	[0.842,0.994]
Middle/Top wealth tercile R5, 2016 (Round 5)	0.922 (0.26)	[0.528,1.611]	0.923 (0.28)	[0.507,1.682]	0.792 (0.23)	[0.453,1.383]	0.756 (0.21)	[0.434,1.315]
N	825		708		825		708	

*Note: Odds ratios are unadjusted odds ratios. Robust standard errors in parenthesis, *** significant at 1%, ** significant at 5%, * significant at 10%. Base categories are as follows: Rural, Believe they are at no/low risk, Did not leave the house at all during the past 7 days, Did not spend more time taking care of children, Did not spend more time on household chores, Did not spend more time working in the family business, Did not face new health expenses, Did not suffer a shock, Never attended school or not enrolled in full-time education/not planning to enrol, Does not have long-term health condition, Lowest wealth tercile. All time-variant variables are measured in 2020 unless otherwise specified.*

Supplementary Table 21. Logistic regression results: Symptoms of at least mild anxiety/depression by gender for the Younger Cohort only (India)

	At least mild anxiety				At least mild depression			
	Male		Female		Male		Female	
	Odds Ratio	95% CI	Odds Ratio	95% CI	Odds Ratio	95% CI	Odds Ratio	95% CI
<u>*Structural factors*</u>								
Age in months	1.007 (0.03)	[0.947,1.069]	0.971 (0.03)	[0.911,1.034]	1.013 (0.03)	[0.955,1.075]	0.968 (0.03)	[0.908,1.031]
Urban	0.570* (0.18)	[0.303,1.070]	2.637*** (0.74)	[1.517,4.585]	0.769 (0.22)	[0.439,1.349]	1.367 (0.45)	[0.720,2.594]
<u>*COVID-19 related changes/behaviours*</u>								
Risk perception: believe they are at medium/high risk	1.455 (0.34)	[0.914,2.315]	0.995 (0.25)	[0.605,1.639]	0.969 (0.22)	[0.622,1.509]	0.989 (0.26)	[0.586,1.669]
Left house for any reason in the past 7 days	0.517* (0.21)	[0.238,1.125]	2.471*** (0.77)	[1.337,4.565]	0.913 (0.39)	[0.392,2.124]	3.923*** (1.55)	[1.811,8.496]
Difference in subjective well-being between Round 5 (2016) and phone survey (2020)	1.012 (0.06)	[0.898,1.141]	1.243*** (0.09)	[1.083,1.427]	1.027 (0.06)	[0.919,1.148]	1.215*** (0.09)	[1.058,1.395]
Change in responsibilities:								
Spend more time taking care of children	2.137*	[0.935,4.884]	2.311**	[1.213,4.403]	1.118	[0.407,3.074]	3.427***	[1.798,6.534]

For peer review only

	At least mild anxiety				At least mild depression			
	Male		Female		Male		Female	
	**Odds Ratio**	**95% CI**	**Odds Ratio**	**95% CI**	**Odds Ratio**	**95% CI**	**Odds Ratio**	**95% CI**
	(0.90)		(0.76)		(0.58)		(1.13)	
Spend more time on household chores	1.370 (0.35)	[0.826,2.274]	0.517*** (0.13)	[0.317,0.843]	1.207 (0.30)	[0.738,1.973]	0.673 (0.19)	[0.390,1.162]
Spend more time working in the family business	2.020* (0.86)	[0.879,4.640]	2.424** (1.09)	[1.005,5.849]	0.750 (0.38)	[0.278,2.021]	0.832 (0.58)	[0.212,3.262]
Economic shocks								
Faced with new health expenses	0.670 (0.17)	[0.413,1.088]	1.165 (0.30)	[0.699,1.944]	1.037 (0.25)	[0.645,1.667]	1.171 (0.36)	[0.646,2.121]
Experienced adversity but did not reduce food consumption	0.627 (0.52)	[0.125,3.157]	0.691 (0.53)	[0.152,3.137]	1.449 (1.56)	[0.177,11.888]	0.313* (0.21)	[0.085,1.153]
Reduced food consumption as response to experienced adversity	1.181 (1.09)	[0.193,7.208]	0.882 (0.78)	[0.155,5.013]	1.943 (2.25)	[0.201,18.787]	0.481 (0.42)	[0.087,2.668]
Educational disruption								
Enrolled in/Planning to enrol in full time education and not participating in learning activities	0.934 (0.27)	[0.530,1.644]	1.134 (0.34)	[0.628,2.049]	0.914 (0.26)	[0.520,1.607]	1.456 (0.47)	[0.770,2.753]
Enrolled in/Planning to enrol in full time education and participating in learning activities	0.684 (0.24)	[0.342,1.369]	0.659 (0.23)	[0.328,1.324]	0.540* (0.19)	[0.276,1.057]	1.122 (0.42)	[0.540,2.332]
Past protective/risk factors								
Participant has long-term health problem, 2016 (Round 5)	1.336 (0.46)	[0.676,2.640]	1.199 (0.46)	[0.568,2.530]	1.447 (0.49)	[0.748,2.802]	1.429 (0.53)	[0.692,2.951]
Total parent-child relationship score, 2012/2016 (Round 4/5)	0.918* (0.04)	[0.838,1.007]	0.922* (0.04)	[0.846,1.005]	0.921* (0.04)	[0.847,1.001]	0.864*** (0.04)	[0.787,0.948]
Total peer-child relationship score, 2016 (Round 5)	0.998 (0.05)	[0.908,1.096]	1.011 (0.05)	[0.910,1.123]	0.973 (0.05)	[0.877,1.080]	1.040 (0.06)	[0.934,1.157]
Middle/Top wealth tercile R5, 2016 (Round 5)	0.877 (0.23)	[0.524,1.468]	0.586* (0.17)	[0.333,1.033]	1.494 (0.41)	[0.877,2.546]	1.129 (0.34)	[0.627,2.033]
N	958		828		958		828	

*Note: Odds ratios are unadjusted odds ratios. Robust standard errors in parenthesis, *** significant at 1%, ** significant at 5%, * significant at 10%. Base categories are as follows: Rural, Believe they are at no/low risk, Did not leave the house at all during the past 7 days, Did not spend more time taking care of children, Did not spend more time on household chores, Did not spend more time working in the family business, Did not face new health expenses, Did not suffer a shock, Never attended school or not enrolled in full-time education/not planning to enrol, Does not have long-term health condition, Lowest wealth tercile. All time-variant variables are measured in 2020 unless otherwise specified.*

For peer review only

Supplementary Table 22. Logistic regression results: Symptoms of at least mild anxiety/depression by gender for the Younger Cohort only (Peru)

	At least mild anxiety				At least mild depression			
	Male		Female		Male		Female	
	**Odds Ratio**	**95% CI**	**Odds Ratio**	**95% CI**	**Odds Ratio**	**95% CI**	**Odds Ratio**	**95% CI**
Structural factors								
Age in months	0.981 (0.02)	[0.939,1.024]	1.004 (0.02)	[0.965,1.045]	0.953** (0.02)	[0.910,0.997]	0.991 (0.02)	[0.952,1.033]
Urban	1.090 (0.26)	[0.684,1.736]	1.658** (0.37)	[1.070,2.569]	1.043 (0.25)	[0.651,1.670]	1.268 (0.30)	[0.793,2.029]
COVID-19 related changes/behaviours								
Risk perception: believe they are at medium/high risk	1.375* (0.23)	[0.988,1.913]	1.488*** (0.23)	[1.100,2.014]	1.500** (0.26)	[1.064,2.114]	1.621*** (0.26)	[1.180,2.226]
Left house for any reason in the past 7 days	1.228 (0.27)	[0.804,1.876]	0.931 (0.17)	[0.650,1.332]	0.998 (0.22)	[0.645,1.546]	0.888 (0.17)	[0.614,1.284]
Difference in subjective well-being between Round 5 (2016) and phone survey (2020)	1.190*** (0.05)	[1.097,1.291]	1.060* (0.04)	[0.989,1.135]	1.137*** (0.05)	[1.036,1.247]	1.045 (0.04)	[0.970,1.126]
Change in responsibilities:								
Spend more time taking care of children	1.784*** (0.33)	[1.238,2.571]	1.384** (0.22)	[1.013,1.890]	1.823*** (0.35)	[1.247,2.665]	0.965 (0.16)	[0.699,1.331]
Spend more time on household chores	1.233 (0.25)	[0.832,1.826]	1.176 (0.25)	[0.771,1.792]	1.118 (0.23)	[0.748,1.671]	1.121 (0.24)	[0.731,1.720]
Spend more time working in the family business	1.094 (0.24)	[0.712,1.681]	1.426 (0.33)	[0.911,2.230]	1.038 (0.24)	[0.664,1.622]	1.238 (0.29)	[0.787,1.950]
Economic shocks								
Faced with new health expenses	2.093*** (0.38)	[1.460,3.000]	1.530*** (0.25)	[1.109,2.110]	2.089*** (0.40)	[1.435,3.041]	1.701*** (0.28)	[1.226,2.360]
Experienced adversity but did not reduce food consumption	4.608** (3.59)	[1.003,21.173]	1.475 (0.93)	[0.427,5.092]	3.693* (2.66)	[0.899,15.172]	1.615 (1.06)	[0.445,5.863]
Reduced food consumption as response to experienced adversity	3.800 (3.22)	[0.721,20.038]	1.249 (0.85)	[0.327,4.769]	4.074* (3.27)	[0.845,19.648]	2.556 (1.83)	[0.631,10.360]
Educational disruption								
Enrolled in/Planning to enrol in full time education and not participating in learning activities	0.559 (0.44)	[0.120,2.602]	0.552 (0.33)	[0.173,1.763]	1.190 (0.94)	[0.253,5.599]	0.407 (0.28)	[0.107,1.546]
Enrolled in/Planning to enrol in full time education and participating in learning activities	0.894 (0.16)	[0.625,1.278]	1.127 (0.18)	[0.825,1.538]	1.215 (0.23)	[0.841,1.754]	0.898 (0.15)	[0.646,1.248]
Past protective/risk factors								
Participant has long-term health problem, 2016 (Round 5)	2.008*** (0.54)	[1.186,3.398]	1.493* (0.33)	[0.964,2.312]	1.910** (0.53)	[1.108,3.293]	1.269 (0.28)	[0.827,1.947]
Total parent-child relationship score, 2012/2016	0.973 (0.03)	[0.922,1.028]	0.977 (0.02)	[0.938,1.019]	0.907***	[0.856,0.961]	0.931***	[0.891,0.973]

For peer review only

	At least mild anxiety				At least mild depression			
	Male		Female		Male		Female	
	Odds Ratio	95% CI	Odds Ratio	95% CI	Odds Ratio	95% CI	Odds Ratio	95% CI
(Round 4/5)					(0.03)		(0.02)	
Total peer-child relationship score, 2016 (Round 5)	1.014 (0.03)	[0.954,1.078]	0.989 (0.02)	[0.947,1.034]	1.034 (0.03)	[0.968,1.104]	1.023 (0.02)	[0.975,1.072]
Middle/Top wealth tercile R5, 2016 (Round 5)	0.892 (0.18)	[0.600,1.325]	1.247 (0.24)	[0.857,1.816]	1.034 (0.21)	[0.695,1.537]	1.239 (0.26)	[0.821,1.871]
N	745		751		745		751	

*Note: Odds ratios are unadjusted odds ratios. Robust standard errors in parenthesis, *** significant at 1%, ** significant at 5%, * significant at 10%. Base categories are as follows: Rural, Believe they are at no/low risk, Did not leave the house at all during the past 7 days, Did not spend more time taking care of children, Did not spend more time on household chores, Did not spend more time working in the family business, Did not face new health expenses, Did not suffer a shock, Never attended school or not enrolled in full-time education/not planning to enrol, Does not have long-term health condition, Lowest wealth tercile. All time-variant variables are measured in 2020 unless otherwise specified.*

Supplementary Table 23. Logistic regression results: Symptoms of at least mild anxiety/depression by gender for the Younger Cohort only (Vietnam)

	At least mild anxiety				At least mild depression			
	Male		Female		Male		Female	
	Odds Ratio	95% CI	Odds Ratio	95% CI	Odds Ratio	95% CI	Odds Ratio	95% CI
<u>*Structural factors*</u>								
Age in months	1.009 (0.04)	[0.933,1.091]	1.027 (0.04)	[0.954,1.106]	1.051 (0.04)	[0.976,1.131]	1.014 (0.04)	[0.938,1.095]
Urban	1.026 (0.30)	[0.576,1.826]	1.550* (0.41)	[0.921,2.609]	0.735 (0.24)	[0.386,1.399]	1.562* (0.40)	[0.942,2.590]
<u>*COVID-19 related changes/behaviours*</u>								
Risk perception: believe they are at medium/high risk	1.553 (0.49)	[0.834,2.890]	0.830 (0.25)	[0.464,1.482]	1.711 (0.61)	[0.851,3.439]	0.775 (0.22)	[0.446,1.347]
Left house for any reason in the past 7 days	0.862 (0.33)	[0.409,1.814]	1.783 (0.64)	[0.879,3.617]	0.944 (0.37)	[0.441,2.022]	1.480 (0.47)	[0.793,2.759]
Difference in subjective well-being between Round 5 (2016) and phone survey (2020)	1.247*** (0.10)	[1.063,1.462]	1.154** (0.07)	[1.021,1.305]	1.444*** (0.12)	[1.221,1.708]	1.008 (0.06)	[0.896,1.133]
Change in responsibilities:								
Spend more time taking care of children	1.005 (0.41)	[0.452,2.238]	1.720** (0.46)	[1.019,2.903]	1.425 (0.55)	[0.667,3.045]	1.796** (0.44)	[1.111,2.902]
Spend more time on household chores	0.948 (0.25)	[0.570,1.575]	1.057 (0.26)	[0.654,1.706]	1.079 (0.31)	[0.619,1.880]	2.902*** (0.80)	[1.694,4.972]

	At least mild anxiety				At least mild depression			
	Male		Female		Male		Female	
	Odds Ratio	95% CI	Odds Ratio	95% CI	Odds Ratio	95% CI	Odds Ratio	95% CI
Spend more time working in the family business	2.053** (0.73)	[1.018,4.140]	1.509 (0.46)	[0.830,2.745]	2.214** (0.79)	[1.099,4.459]	1.750* (0.50)	[0.998,3.068]
Economic shocks								
Faced with new health expenses	0.930 (0.50)	[0.323,2.684]	1.523 (0.62)	[0.688,3.368]	1.294 (0.63)	[0.498,3.365]	0.476 (0.24)	[0.181,1.255]
Experienced adversity but did not reduce food consumption	2.415*** (0.77)	[1.295,4.502]	1.091 (0.29)	[0.652,1.826]	2.411*** (0.81)	[1.245,4.668]	1.502 (0.40)	[0.888,2.542]
Reduced food consumption as response to experienced adversity	2.963*** (1.22)	[1.320,6.652]	1.166 (0.37)	[0.628,2.165]	2.427** (1.04)	[1.050,5.613]	2.449*** (0.74)	[1.350,4.441]
Educational disruption								
Enrolled in/Planning to enrol in full time education and not participating in learning activities	0.954 (0.44)	[0.390,2.333]	0.817 (0.34)	[0.358,1.862]	1.153 (0.58)	[0.431,3.085]	0.967 (0.35)	[0.476,1.966]
Enrolled in/Planning to enrol in full time education and participating in learning activities	0.678 (0.23)	[0.350,1.315]	0.714 (0.19)	[0.420,1.216]	1.250 (0.43)	[0.639,2.445]	1.058 (0.29)	[0.622,1.799]
Past protective/risk factors								
Participant has long-term health problem, 2016 (Round 5)	1.190 (0.58)	[0.458,3.088]	1.036 (0.37)	[0.517,2.074]	2.696** (1.17)	[1.151,6.317]	1.204 (0.40)	[0.630,2.300]
Total parent-child relationship score, 2012/2016 (Round 4/5)	1.004 (0.05)	[0.918,1.098]	1.055 (0.04)	[0.981,1.135]	0.925 (0.05)	[0.833,1.026]	0.999 (0.04)	[0.931,1.072]
Total peer-child relationship score, 2016 (Round 5)	1.058 (0.06)	[0.950,1.178]	0.916 (0.05)	[0.821,1.022]	1.063 (0.07)	[0.926,1.220]	0.948 (0.05)	[0.852,1.054]
Middle/Top wealth tercile R5, 2016 (Round 5)	0.583* (0.18)	[0.323,1.055]	0.741 (0.18)	[0.454,1.208]	1.314 (0.45)	[0.672,2.566]	1.164 (0.31)	[0.693,1.955]
N	790		806		790		806	

*Note: Odds ratios are unadjusted odds ratios. Robust standard errors in parenthesis, *** significant at 1%, ** significant at 5%, * significant at 10%. Base categories are as follows: Rural, Believe they are at no/low risk, Did not leave the house at all during the past 7 days, Did not spend more time taking care of children, Did not spend more time on household chores, Did not spend more time working in the family business, Did not face new health expenses, Did not suffer a shock, Never attended school or not enrolled in full-time education/not planning to enrol, Does not have long-term health condition, Lowest wealth tercile. All time-variant variables are measured in 2020 unless otherwise specified.*

For peer review only - http://bmjopen.bmj.com/site/about/guidelines.xhtml

Supplementary Table 24. Ethiopia: Mean score and rates of general anxiety disorder

	Mean GAD-7 score	CI95%	p-value	Minimal (0-4)	CI95%	p-value	Mild anxiety (5-9)	CI95%	p-value	Moderate anxiety (10-14)	CI95%	p-value	Severe anxiety (>=15)	CI95%	p-value
**Total**	**2.06**	**1.94; 2.19**		**82.13**	**80.46; 83.72**		**15.21**	**13.73; 16.78**		**2.34**	**1.74; 3.06**		**0.32**	**0.13; 0.66**	
Male	2.03	1.86; 2.20	0.603	82.52	80.35; 84.70	0.610	14.66	12.64; 16.69	0.445	2.47	1.58; 3.36	0.650	0.34	0.01; 0.67	0.856
Female	2.10	1.92; 2.28		81.68	79.30; 84.07		15.84	13.59; 18.10		2.18	1.28; 3.08		0.30	-0.04; 0.63	
Rural	1.78	1.62; 1.93	0.000	85.33	83.30; 87.35	0.000	12.55	10.66; 14.45	0.000	2.12	1.30; 2.94	0.470	0.00	0.00; 0.00	0.004
Urban	2.40	2.20; 2.60		78.39	75.84; 80.94		18.33	15.93; 20.72		2.59	1.61; 3.57		0.70	0.18; 1.21	
Poorest tercile	1.89	1.68; 2.10	0.056	85.10	82.49; 87.71	0.011	11.98	9.60; 14.36	0.003	2.65	1.47; 3.82	0.502	0.28	-0.11; 0.66	0.808
Middle/Richest terciles	2.15	1.99; 2.30		80.68	78.66; 82.71		16.79	14.88; 18.71		2.18	1.44; 2.93		0.34	0.04; 0.64	
No internet	1.90	1.74; 2.07	0.010	84.03	81.89; 86.18	0.017	13.20	11.22; 15.19	0.007	2.50	1.58; 3.41	0.608	0.27	-0.04; 0.57	0.653
With internet	2.23	2.05; 2.41		80.13	77.73; 82.53		17.33	15.05; 19.60		2.17	1.29; 3.04		0.38	0.01; 0.75	
N	2183														

Notes: If any missing answers to questions then the whole score is set to missing. p-values represent significance of t-test of equality between groups (Male-Female; Rural-Urban; Bottom-Top/Middle wealth tercile; Internet access through home computer/working smartphone (No- [illegible] *position in the 2016 (round 5) wealth distribution.*
Results are for the combined Younger Cohort/Older Cohort sample.

For peer review only

Supplementary Table 25. Ethiopia: Mean score and rates of depression

	Mean PHQ-8 score	CI95%	p-val	PHQ (0-4)	CI95%	p-val	PHQ (5-9)	CI95%	p-val	PHQ (10-14)	CI95%	p-val	PHQ (15-19)	CI95%	p-val	PHQ (>=20)	CI95%	p-val
**Total**	**1.89**	**1.78; 2.01**		**84.56**	**82.98; 86.05**		**13.33**	**11.93; 14.83**		**1.97**	**1.43; 2.64**		**0.09**	**0.01; 0.33**		**0.05**	**0.00; 0.25**	
Male	1.86	1.70; 2.02	0.569	85.08	83.04; 87.12	0.470	12.79	10.87; 14.70	0.422	1.88	1.10; 2.65	0.733	0.17	-0.07; 0.41	0.189	0.09	-0.08; 0.25	0.354
Female	1.93	1.76; 2.10		83.96	81.69; 86.23		13.96	11.82; 16.10		2.08	1.20; 2.96		0.00	0.00; 0.00		0.00	0.00; 0.00	
Rural	1.66	1.52; 1.81	0.000	87.11	85.19; 89.02	0.000	11.54	9.71; 13.36	0.007	1.27	0.63; 1.91	0.011	0.08	-0.08; 0.25	0.909	0.00	0.00; 0.00	0.279
Urban	2.16	1.97; 2.34		81.57	79.17; 83.97		15.44	13.20; 17.68		2.79	1.77; 3.81		0.10	-0.10; 0.29		0.10	-0.10; 0.29	
Poorest tercile	1.75	1.55; 1.95	0.086	85.65	83.09; 88.22	0.323	12.53	10.11; 14.96	0.444	1.53	0.63; 2.43	0.303	0.28	-0.11; 0.66	0.043	0.00	0.00; 0.00	0.484
Middle/Richest terciles	1.96	1.82; 2.10		84.03	82.15; 85.90		13.72	11.96; 15.48		2.18	1.44; 2.93		0.00	0.00; 0.00		0.07	-0.07; 0.20	
No internet	1.69	1.54; 1.85	0.001	85.91	83.87; 87.94	0.074	12.49	10.55; 14.43	0.235	1.52	0.80; 2.23	0.118	0.09	-0.09; 0.26	0.970	0.00	0.00; 0.00	0.304
With internet	2.10	1.93; 2.28		83.15	80.89; 85.40		14.22	12.12; 16.32		2.45	1.52; 3.38		0.09	-0.09; 0.28		0.09	-0.09; 0.28	
N		2183																

Notes: If any missing answers to questions then the whole score is set to missing. p-values represent significance of t-test of equality between groups (Male-Female; Rural-Urban; Bottom-Top/Middle wealth tercile; Internet access through home computer/working smartphone (No-Yes)). Poorest and middle/richest terciles refer to [illegible]
Results are for the combined Younger Cohort/ Older Cohort sample.

For peer review only

Supplementary Table 26.India: Mean score and rates of general anxiety disorder

	Mean GAD-7 score	CI95%	p-value	Minimal (0-4)	CI95%	p-value	Mild anxiety (5-9)	CI95%	p-value	Moderate anxiety (10-14)	CI95%	p-value	Severe anxiety (>=15)	CI95%	p-value
**Total**	**1.69**	**1.59; 1.78**		**88.94**	**87.68; 90.12**		**9.15**	**8.08; 10.32**		**1.64**	**1.19; 2.20**		**0.27**	**0.11; 0.55**	
Male	1.55	1.43; 1.67	0.003	90.72	89.19; 92.26	0.002	8.04	6.59; 9.48	0.038	1.10	0.54; 1.65	0.022	0.15	-0.06; 0.35	0.210
Female	1.84	1.69; 1.99		86.99	85.13; 88.86		10.38	8.69; 12.06		2.23	1.42; 3.05		0.40	0.05; 0.75	
Rural	1.76	1.65; 1.88	0.014	88.19	86.73; 89.66	0.055	9.56	8.23; 10.90	0.252	1.92	1.30; 2.55	0.071	0.32	0.06; 0.58	0.401
Urban	1.50	1.34; 1.66		90.80	88.73; 92.87		8.13	6.17; 10.09		0.93	0.24; 1.62		0.13	-0.13; 0.39	
Poorest tercile	1.83	1.66; 2.00	0.042	86.48	84.22; 88.74	0.004	11.02	8.95; 13.09	0.018	2.16	1.20; 3.12	0.137	0.34	-0.04; 0.73	0.602
Middle/Richest terciles	1.62	1.50; 1.73		90.18	88.79; 91.58		8.21	6.92; 9.50		1.38	0.83; 1.93		0.23	0.00; 0.45	
No internet	1.80	1.52; 2.08	0.410	87.84	84.11; 91.57	0.521	10.47	6.98; 13.97	0.403	1.69	0.22; 3.16	0.944	0.00	0.00; 0.00	0.345
With internet	1.67	1.57; 1.78		89.08	87.81; 90.35		8.99	7.82; 10.15		1.63	1.12; 2.15		0.30	0.08; 0.52	
N	2622														

Notes: If any missing answers to questions then the whole score is set to missing. p-values represent significance of t-test of equality between groups (Male-Female; Rural-Urban; Bottom-Top/Middle wealth tercile; Internet access through home computer/working smartphone (No-Yes)). Poorest and middle/richest terciles refer to the [illegible]tion in the 2016 (round 5) wealth distribution.
Results are for the combined Younger Cohort/ Older Cohort sample.

Supplementary Table 27. India: Mean score and rates of depression

	Mean PHQ-8 score	CI95%	p-value	No significant depressive symptoms(0-4)	CI95%	p-value	Mild depression (5-9)	CI95%	p-value	Moderate depressive symptoms(10-14)	CI95%	p-value	Moderately severe depressive symptoms(15-19)	CI95%	p-value
**Total**	**1.41**	**1.33; 1.5**		**90.08**	**88.88; 91.2**		**8.73**	**7.68; 9.88**		**1.03**	**0.68; 1.49**		**0.15**	**0.04; 0.39**	
Male	1.34	1.22; 1.45	0.067	90.36	88.79; 91.92	0.624	8.84	7.33; 10.34	0.843	0.73	0.28; 1.18	0.113	0.07	-0.07; 0.22	0.276
Female	1.50	1.37; 1.63		89.78	88.11; 91.46		8.62	7.06; 10.17		1.36	0.72; 2.00		0.24	-0.03; 0.51	
Rural	1.47	1.36; 1.58	0.039	89.16	87.75; 90.57	0.012	9.40	8.08; 10.72	0.056	1.28	0.77; 1.79	0.043	0.16	-0.02; 0.34	0.873
Urban	1.27	1.12; 1.42		92.40	90.50; 94.30		7.07	5.23; 8.90		0.40	-0.05; 0.85		0.13	-0.13; 0.39	
Poorest tercile	1.49	1.33; 1.65	0.220	89.20	87.15; 91.26	0.284	9.55	7.60; 11.49	0.296	1.02	0.36; 1.69	0.980	0.23	-0.09; 0.54	0.486
Middle/Richest terciles	1.37	1.27; 1.48		90.53	89.15; 91.90		8.32	7.03; 9.620		1.03	0.56; 1.51		0.11	-0.04; 0.27	
No internet	1.42	1.17; 1.67	0.941	89.86	86.42; 93.31	0.894	8.78	5.55; 12.02	0.974	1.35	0.03; 2.67	0.561	0.00	0.00; 0.00	0.475
With internet	1.41	1.32; 1.50		90.11	88.90; 91.33		8.73	7.58; 9.88		0.99	0.59; 1.39		0.17	0.00; 0.34	
N	2622														

Notes: If any missing answers to questions then the whole score is set to missing. p-values represent significance of t-test of equality between groups (Male-Female; Rural-Urban; Bottom-Top/Middle wealth tercile; Internet access through home computer/working smartphone (No-Yes)). Poorest and middle/richest terciles refer to the [illegible] in the 2016 (round 5) wealth distribution.
Results are for the combined Younger Cohort/ Older Cohort sample. No severe depressive symptoms were reported.

For peer review only

Supplementary Table 28. Peru: Mean score and rates of general anxiety disorder

	Mean GAD-7 score	CI95%	p-value	Minimal (0-4)	CI95%	p-value	Mild anxiety (5-9)	CI95%	p-value	Moderate anxiety (10-14)	CI95%	p-value	Severe anxiety (>=15)	CI95%	p-value
**Total**	**4.53**	**4.34; 4.72**		**59.14**	**56.88; 61.37**		**27.34**	**25.34; 29.42**		**10.55**	**9.20; 12.02**		**2.97**	**2.25; 3.84**	
Male	3.80	3.55; 4.04	0.000	66.35	63.36; 69.35	0.000	23.72	21.02; 26.42	0.000	7.94	6.23; 9.66	0.000	1.99	1.10; 2.87	0.011
Female	5.29	5.01; 5.57		51.72	48.51; 54.94		31.08	28.10; 34.05		13.23	11.05; 15.41		3.98	2.72; 5.24	
Rural	3.87	3.46; 4.27	0.001	66.29	61.37; 71.21	0.002	25.56	21.02; 30.10	0.402	5.62	3.22; 8.01	0.001	2.53	0.89; 4.16	0.588
Urban	4.69	4.47; 4.90		57.48	55.00; 59.96		27.76	25.51; 30.00		11.69	10.08; 13.30		3.07	2.20; 3.93	
Poorest tercile	4.05	3.74; 4.35	0.001	63.08	59.16; 66.99	0.020	27.01	23.41; 30.61	0.826	8.21	5.98; 10.43	0.026	1.71	0.66; 2.76	0.031
Middle/Richest terciles	4.75	4.51; 4.99		57.37	54.68; 60.06		27.50	25.07; 29.92		11.60	9.86; 13.34		3.53	2.53; 4.54	
No internet	4.96	4.14; 5.78	0.367	48.68	37.37; 60.00	0.058	36.84	25.92; 47.77	0.058	14.47	6.51; 22.44	0.255	0.00	0.00; 0.00	0.120
With internet	4.51	4.32; 4.71		59.58	57.32; 61.84		26.95	24.90; 28.99		10.38	8.97; 11.79		3.09	2.29; 3.89	
N	1887														

Notes: If any missing answers to questions then the whole score is set to missing. p-values represent significance of t-test of equality between groups (Male-Female; Rural-Urban; Bottom-Top/Middle wealth tercile; Internet access through home computer/working smartphone (No-Yes)). Poorest and middle/richest terciles refer to the [illegible] in the 2016 (round 5) wealth distribution.
Results are for the combined Younger Cohort/ Older Cohort sample.

Supplementary Table 29. Peru: Mean score and rates of depression

	Mean PHQ-8 score	CI 95%	p-val	PHQ (0-4)	CI 95%	p-val	PHQ (5-9)	CI 95%	p-val	PHQ (10-14)	CI 95%	p-val	PHQ (15-19)	CI 95%	p-val	PHQ (>=20)	CI 95%	p-val
**Total**	**3.61**	**3.43; 3.79**		**68.42**	**66.26; 70.51**		**21.94**	**20.09; 23.88**		**7.79**	**6.62; 9.09**		**1.48**	**0.99; 2.14**		**0.37**	**0.15; 0.76**	
Male	3.08	2.85; 3.32	0.000	72.62	69.79; 75.45	0.000	19.75	17.22; 22.27	0.020	6.58	5.01; 8.16	0.047	0.73	0.19; 1.27	0.006	0.31	-0.04; 0.67	0.677
Female	4.15	3.89; 4.41		64.09	61.00; 67.17		24.19	21.44; 26.95		9.03	7.19; 10.88		2.26	1.30; 3.21		0.43	0.01; 0.85	
Rural	3.11	2.73; 3.49	0.008	73.60	69.01; 78.18	0.020	19.38	15.27; 23.50	0.196	6.18	3.67; 8.69	0.208	0.84	-0.11; 1.79	0.267	0.00	0.00; 0.00	0.201
Urban	3.73	3.53; 3.92		67.21	64.86; 69.56		22.53	20.44; 24.63		8.16	6.79; 9.54		1.63	1.00; 2.27		0.46	0.12; 0.80	
Poorest tercile	3.07	2.79; 3.36	0.000	73.85	70.28; 77.41	0.001	19.66	16.43; 22.88	0.109	5.47	3.62; 7.32	0.012	0.68	0.01; 1.35	0.054	0.34	-0.13; 0.82	0.889
Middle/ Richest terciles	3.85	3.63; 4.07		65.98	63.40; 68.55		22.96	20.68; 25.25		8.83	7.29; 10.38		1.84	1.11; 2.57		0.38	0.05; 0.72	
No internet	3.34	2.62; 4.07	0.545	68.42	57.89; 78.95	0.999	25.00	15.19; 34.81	0.511	6.58	0.96; 12.19	0.688	0.00	0.00; 0.00	0.275	0.00	0.00; 0.00	0.587
With internet	3.62	3.44; 3.80		68.42	66.27; 70.56		21.81	19.91; 23.71		7.84	6.60; 9.08		1.55	0.98; 2.11		0.39	0.10; 0.67	
N	1887																	

Notes: If any missing answers to questions then the whole score is set to missing. p-values represent significance of t-test of equality between groups (Male-Female; Rural-Urban; Bottom-Top/Middle wealth tercile; Internet access through home computer/working smartphone (No-Yes)). Poorest and middle/richest terciles refer to the [illegible]
Results are for the combined Younger Cohort/ Older Cohort sample.

Supplementary Table 30. Vietnam: Mean score and rates of general anxiety disorder

	Mean GAD-7 score	CI95%	p-value	Minimal (0-4)	CI95%	p-value	Mild anxiety (5-9)	CI95%	p-value	Moderate anxiety (10-14)	CI95%	p-value	Severe anxiety (>=15)	CI95%	p-value
**Total**	**1.47**	**1.37; 1.56**		**90.68**	**89.42; 91.84**		**7.93**	**6.85; 9.11**		**1.09**	**0.71; 1.60**		**0.30**	**0.12; 0.63**	
Male	1.31	1.17; 1.44	0.001	92.08	90.49; 93.67	0.025	6.48	5.03; 7.93	0.013	1.17	0.54; 1.80	0.717	0.27	-0.04; 0.58	0.769
Female	1.62	1.48; 1.76		89.37	87.61; 91.12		9.28	7.63; 10.94		1.01	0.44; 1.58		0.34	0.01; 0.67	
Rural	1.47	1.34; 1.60	0.925	90.93	89.33; 92.53	0.652	7.62	6.15; 9.10	0.559	1.04	0.48; 1.61	0.819	0.40	0.05; 0.75	0.362
Urban	1.46	1.32; 1.60		90.38	88.60; 92.17		8.29	6.62; 9.95		1.14	0.50; 1.79		0.19	-0.07; 0.45	
Poorest tercile	1.60	1.42; 1.78	0.045	88.33	86.08; 90.59	0.006	10.00	7.89; 12.11	0.008	1.15	0.40; 1.90	0.830	0.51	0.01; 1.01	0.195
Middle/Richest terciles	1.40	1.29; 1.51		91.89	90.51; 93.26		6.86	5.59; 8.13		1.06	0.54; 1.57		0.20	-0.03; 0.42	
No internet	2.40	1.24; 3.56	0.017	77.14	63.02; 91.26	0.005	17.14	4.47; 29.82	0.042	2.86	-2.75; 8.46	0.310	2.86	-2.75; 8.46	0.006
With internet	1.45	1.36; 1.55		90.89	89.70; 92.08		7.78	6.68; 8.89		1.06	0.64; 1.48		0.27	0.05; 0.48	
N	2296														

Notes: If any missing answers to questions then the whole score is set to missing. p-values represent significance of t-test of equality between groups (Male-Female; Rural-Urban; Bottom-Top/Middle wealth tercile; Internet access through home computer/working smartphone (No-Yes)). Poorest and middle/richest terciles refer to the [illegible]tion in the 2016 (round 5) wealth distribution.

Results are for the combined Younger Cohort/ Older Cohort sample.

Supplementary Table 31. Vietnam: Mean score and rates of depression

	Mean PHQ-8 score	CI95%	p-value	No significant depressive symptoms(0-4)	CI95%	p-value	Mild depression (5-9)	CI95%	p-value	Moderate depr-essive symptoms (10-14)	CI95%	p-value	Moderately severe depressive symptoms(15-19)	CI95%	p-value
**Total**	**1.42**	**1.32; 1.52**		**90.51**	**89.23; 91.67**		**7.67**	**6.61; 8.83**		**1.39**	**0.96; 1.96**		**0.44**	**0.21; 0.80**	
Male	1.24	1.11; 1.37	0.000	92.35	90.78; 93.91	0.004	6.12	4.71; 7.53	0.007	1.17	0.54; 1.80	0.376	0.36	0.01; 0.71	0.595
Female	1.59	1.45; 1.74		88.78	86.98; 90.58		9.11	7.47; 10.75		1.60	0.89; 2.32		0.51	0.10; 0.91	
Rural	1.43	1.30; 1.56	0.852	90.61	88.99; 92.23	0.852	7.70	6.22; 9.19	0.939	1.20	0.6; 1.81	0.398	0.48	0.10; 0.87	0.716
Urban	1.41	1.26; 1.56		90.38	88.60; 92.17		7.62	6.01; 9.23		1.62	0.85; 2.38		0.38	0.01; 0.75	
Poorest tercile	1.40	1.23; 1.57	0.757	91.54	89.58; 93.49	0.226	6.28	4.58; 7.99	0.074	1.54	0.67; 2.40	0.671	0.64	0.08; 1.20	0.284
Middle/Richest terciles	1.43	1.31; 1.55		89.97	88.46; 91.49		8.38	6.98; 9.77		1.32	0.74; 1.89		0.33	0.04; 0.62	
No internet	2.20	1.05; 3.35	0.053	85.71	73.95; 97.48	0.330	8.57	-0.84; 17.99	0.839	2.86	-2.75; 8.46	0.457	2.86	-2.75; 8.46	0.028
With internet	1.41	1.31; 1.51		90.58	89.37; 91.78		7.65	6.55; 8.75		1.37	0.89; 1.85		0.40	0.14; 0.66	
N	2296														

Notes: If any missing answers to questions then the whole score is set to missing. p-values represent significance of t-test of equality between groups (Male-Female; Rural-Urban; Bottom-Top/Middle wealth tercile; Internet access through home computer/working smartphone (No-Yes)). Poorest and middle/richest terciles refer to the [illegible]
Results are for the combined Younger Cohort/ Older Cohort sample. No severe depressive symptoms were reported.

For peer review only

Supplementary Table 32. Correlation between GAD-7 and PHQ-8 scores

	Ethiopia	India	Peru	Vietnam
Correlation coefficient	0.629***	0.610***	0.700***	0.636***

*Note: Bonferri corrected p-value *** significant at 1%, ** significant at 5%, * significant at 10% for the unconditional correlation between GAD-7 and PHQ-8. Results are for the combined Younger Cohort/ Older Cohort sample.*

Supplementary Table 33. Joint rates

	Ethiopia		India		Peru		Vietnam	
	% of sample	95% CI	% of sample	95% CI	% of sample	95% CI	% of sample	95% CI
Display neither symptoms of anxiety nor depression	75.45	73.58; 77.24	83.87	82.40; 85.26	52.36	50.08; 54.63	85.50	83.99; 86.91
Display symptoms of at least mild anxiety only	9.12	7.94; 10.4	6.22	5.32; 7.21	16.06	14.43; 17.79	5.01	4.15; 5.98
Display symptoms of at least mild depression only	6.69	7.94; 10.4	5.07	5.32; 7.21	6.78	14.43; 17.79	5.18	4.15; 5.98
Display symptoms of at least mild anxiety and at least mild depression	8.75	7.60; 10.01	4.84	4.05; 5.74	24.80	22.87; 26.81	4.31	3.52; 5.22

Note: Results are for the combined Younger Cohort/ Older Cohort sample.

For peer review only

Supplementary Table 34. Correlation between subjective well-being and GAD-7/PHQ-8 scores

	Ethiopia				India				Peru				Vietnam			
	Subjective well-being (phone survey, 2020)		Subjective well-being (difference Round 5 (2016) and phone survey (2020))		Subjective well-being (phone survey, 2020)		Subjective well-being (difference Round 5 (2016) and phone survey (2020))		Subjective well-being (phone survey, 2020)		Subjective well-being (difference Round 5 (2016) and phone survey (2020))		Subjective well-being (phone survey, 2020)		Subjective well-being (difference Round 5 (2016) and phone survey (2020))	
	r	p-value	r	p-value	r	p-value	r	p-value	r	p-value	r	p-value	r	p-value	r	p-value
Mean GAD-7 score	-0.140	0.000	0.041	0.054	-0.107	0.000	0.064	0.001	-0.199	0.000	0.096	0.000	-0.253	0.000	0.118	0.000
Mean PHQ-8 score	-0.091	0.000	0.024	0.263	-0.097	0.000	0.055	0.005	-0.186	0.000	0.067	0.004	-0.207	0.000	0.119	0.000

Note: Results are for the combined Younger Cohort/ Older Cohort sample.

For peer review only

Supplementary Table 35. Subjective well-being score Round 5 (2016) / phone survey (2020) by anxiety/depression (Ethiopia & India)

	Ethiopia							India						
	**All**	**at least mild anxiety**	**no anxiety**	**p-value**	**at least mild depression**	**no depression**	**p-value**	**All**	**at least mild anxiety**	**no anxiety**	**p-value**	**at least mild depression**	**no depression**	**p-value**
(1) Subjective well-being, 2016 (Round 5)	5.69	5.43	5.75	0.000	5.61	5.71	0.303	5.05	4.94	5.06	0.178	4.98	5.06	0.381
(2) Subjective well-being, 2020 (Phone survey)	4.73	4.28	4.83	0.000	4.36	4.80	0.000	4.56	4.18	4.61	0.000	4.12	4.61	0.000
Difference in subjective well-being (1)-(2)	0.96	1.15	0.92	0.052	1.26	0.91	0.005	0.49	0.77	0.45	0.009	0.86	0.45	0.001

Note: Results are for the combined Younger Cohort/ Older Cohort sample.

For peer review only

Supplementary Table 36. Subjective well-being score Round 5 (2016) / phone survey (2020) by anxiety/depression (Peru & Vietnam)

	Peru							Vietnam						
	All	at least mild anxiety	no anxiety	p-value	at least mild depression	no depression	p-value	All	at least mild anxiety	no anxiety	p-value	at least mild depression	no depression	p-value
(1) Subjective well-being, 2016 (Round 5)	6.33	6.20	6.42	0.001	6.14	6.42	0.000	5.87	5.46	5.91	0.000	5.65	5.89	0.024
(2) Subjective well-being, 2020 (Phone survey)	5.81	5.47	6.05	0.000	5.44	5.98	0.000	6.26	5.33	6.36	0.000	5.54	6.34	0.000
Difference in subjective well-being (1)-(2)	0.52	0.74	0.38	0.000	0.70	0.44	0.013	-0.39	0.14	-0.45	0.000	0.11	-0.45	0.000

Note: Results are for the combined Younger Cohort/ Older Cohort sample.

For peer review only

Research checklist

STROBE Statement for: *Impact of the COVID-19 Pandemic on anxiety and depression symptoms of young people in the Global South: evidence from a four-country cohort study*

	Item No	Recommendation	Reported on page #
**Title and abstract**	1	(*a*) Indicate the study's design with a commonly used term in the title or the abstract	1
		(*b*) Provide in the abstract an informative and balanced summary of what was done and what was found	2
**Introduction**			
Background/rationale	2	Explain the scientific background and rationale for the investigation being reported	3
Objectives	3	State specific objectives, including any prespecified hypotheses	3-4
**Methods**			
Study design	4	Present key elements of study design early in the paper	4
Setting	5	Describe the setting, locations, and relevant dates, including periods of recruitment, exposure, follow-up, and data collection	4
Participants	6	(*a*) Give the eligibility criteria, and the sources and methods of selection of participants. Describe methods of follow-up	4
		(*b*) For matched studies, give matching criteria and number of exposed and unexposed	N/A
Variables	7	Clearly define all outcomes, exposures, predictors, potential confounders, and effect modifiers. Give diagnostic criteria, if applicable	4-5
Data sources/ measurement	8*	For each variable of interest, give sources of data and details of methods of assessment (measurement). Describe comparability of assessment methods if there is more than one group	4-5, 16
Bias	9	Describe any efforts to address potential sources of bias	9, 11
Study size	10	Explain how the study size was arrived at	4
Quantitative variables	11	Explain how quantitative variables were handled in the analyses. If applicable, describe which groupings were chosen and why	4-5
Statistical methods	12	(*a*) Describe all statistical methods, including those used to control for confounding	4
		(*b*) Describe any methods used to examine subgroups and interactions	4
		(*c*) Explain how missing data were addressed	6
		(*d*) If applicable, explain how loss to follow-up was addressed	N/A
		(*e*) Describe any sensitivity analyses	N/A

For peer review only - http://bmjopen.bmj.com/site/about/guidelines.xhtml
For peer review only

**Results**			
Participants	13*	(a) Report numbers of individuals at each stage of study—eg numbers potentially eligible, examined for eligibility, confirmed eligible, included in the study, completing follow-up, and analysed	1, 6<br>Supplementary Tables 1- 3
		(b) Give reasons for non-participation at each stage	1, 6<br>Supplementary Tables 1- 3
		(c) Consider use of a flow diagram	N/A
Descriptive data	14*	(a) Give characteristics of study participants (eg demographic, clinical, social) and information on exposures and potential confounders	4<br>Supplementary Table 5
		(b) Indicate number of participants with missing data for each variable of interest	1, 6<br>Supplementary Tables 1- 3
		(c) Summarise follow-up time (eg, average and total amount)	N/A
Outcome data	15*	Report numbers of outcome events or summary measures over time	4
Main results	16	(*a*) Give unadjusted estimates and, if applicable, confounder-adjusted estimates and their precision (eg, 95% confidence interval). Make clear which confounders were adjusted for and why they were included	6-14
		(*b*) Report category boundaries when continuous variables were categorized	N/A
		(*c*) If relevant, consider translating estimates of relative risk into absolute risk for a meaningful time period	N/A
Other analyses	17	Report other analyses done—eg analyses of subgroups and interactions, and sensitivity analyses	5, 8-14<br>Supplementary Tables 6-9, 16-23
**Discussion**			
Key results	18	Summarise key results with reference to study objectives	14
Limitations	19	Discuss limitations of the study, taking into account sources of potential bias or imprecision. Discuss both direction and magnitude of any potential bias	15
Interpretation	20	Give a cautious overall interpretation of results considering objectives, limitations, multiplicity of analyses, results from similar studies, and other relevant evidence	15
Generalisability	21	Discuss the generalisability (external validity) of the study results	15
**Other information**			
Funding	22	Give the source of funding and the role of the funders for the present study and, if applicable, for the original study on which the present article is based	17

For peer review only

*Give information separately for exposed and unexposed groups.

Note: An Explanation and Elaboration article discusses each checklist item and gives methodological background and published examples of transparent reporting. The STROBE checklist is best used in conjunction with this article (freely available on the Web sites of PLoS Medicine at http://www.plosmedicine.org/, Annals of Internal Medicine at http://www.annals.org/, and Epidemiology at http://www.epidem.com/). Information on the STROBE Initiative is available at http://www.strobe-statement.org.

For peer review only

BMJ Open

Impact of the COVID-19 Pandemic on anxiety and depression symptoms of young people in the Global South: evidence from a four-country cohort study

Journal:	*BMJ Open*
Manuscript ID	bmjopen-2021-049653.R1
Article Type:	Original research
Date Submitted by the Author:	18-Mar-2021
Complete List of Authors:	Porter, Catherine; Lancaster University, Management School<br>Favara, Marta; University of Oxford, Oxford Department of International Development<br>Hittmeyer, Annina; University of Oxford Department of International Development<br>Scott, Douglas; University of Oxford, Oxford Department of International Development<br>Sánchez Jiménez, Alan<br>Ellanki, Revathi; Centre for Economic and Social Studies<br>Woldehanna, T; Addis Ababa University, Department of Economics<br>Duc, Le Thuc; Vietnam Academy of Social Sciences<br>Craske, Michelle; University of California Los Angeles, UCLA Department of Psychology<br>Stein, Alan; University of Oxford; University of the Witwatersrand
**Primary Subject Heading**:	Mental health
Secondary Subject Heading:	Infectious diseases
Keywords:	COVID-19, Anxiety disorders < PSYCHIATRY, Depression & mood disorders < PSYCHIATRY

SCHOLARONE™
Manuscripts

I, the Submitting Author has the right to grant and does grant on behalf of all authors of the Work (as defined in the below author licence), an exclusive licence and/or a non-exclusive licence for contributions from authors who are: i) UK Crown employees; ii) where BMJ has agreed a CC-BY licence shall apply, and/or iii) in accordance with the terms applicable for US Federal Government officers or employees acting as part of their official duties; on a worldwide, perpetual, irrevocable, royalty-free basis to BMJ Publishing Group Ltd ("BMJ") its licensees and where the relevant Journal is co-owned by BMJ to the co-owners of the Journal, to publish the Work in this journal and any other BMJ products and to exploit all rights, as set out in our licence.

The Submitting Author accepts and understands that any supply made under these terms is made by BMJ to the Submitting Author unless you are acting as an employee on behalf of your employer or a postgraduate student of an affiliated institution which is paying any applicable article publishing charge ("APC") for Open Access articles. Where the Submitting Author wishes to make the Work available on an Open Access basis (and intends to pay the relevant APC), the terms of reuse of such Open Access shall be governed by a Creative Commons licence – details of these licences and which Creative Commons licence will apply to this Work are set out in our licence referred to above.

Other than as permitted in any relevant BMJ Author's Self Archiving Policies, I confirm this Work has not been accepted for publication elsewhere, is not being considered for publication elsewhere and does not duplicate material already published. I confirm all authors consent to publication of this Work and authorise the granting of this licence.

For peer review only

Impact of the COVID-19 Pandemic on anxiety and depression symptoms of young people in the Global South: evidence from a four-country cohort study

Catherine Porter[1], Marta Favara[2], Annina Hittmeyer[2], Douglas Scott[2], Alan Sánchez Jiménez[3], Revathi Ellanki[4], Tassew Woldehanna[5], Le Thuc Duc[6], Michelle G. Craske[7], Alan Stein[8,9,10]

17th March 2021

[1] Lancaster University Management School, Bailrigg, Lancaster LA1 4YX, United Kingdom
[2] Oxford Department of International Development, University of Oxford, 3 Mansfield Rd, Oxford OX1 3TB, United Kingdom
[3] Grupo de Análisis para el Desarrollo (GRADE), Av. Grau 915, Barranco, Lima 15063, Peru
[4] Centre for Economic and Social Studies (CESS), Nizamia Observatory Campus, Begumpet, Hyderabad 500016, Telangana, India
[5] Department of Economics, Addis Ababa University (AAU), P.O. Box 150175, Addis Ababa, Ethiopia
[6] Centre for Analysis and Forecasting, Vietnam Academy of Social Sciences, No.1, Lieu Giai Street, Hanoi, Vietnam
[7] Distinguished Professor Joanne and George Miller and Family Endowed Term Chair (BRI), Director, Anxiety and Depression Research Center (ADRC), Director, Innovative Treatment Network, UCLA Depression Grand Challenge, Associate Director, Staglin Family Music Center for Behavioral & Brain Health, Editor-in-Chief, Behaviour Research and Therapy, UCLA Department of Psychology and Department of Psychiatry & Biobehavioral Sciences, University of California, Franz Hall – Box 951563, Los Angeles, CA 90094-1563, United States
[8] Department of Psychiatry, University of Oxford, Warneford Hospital, Oxford OX3 7JX, United Kingdom
[9] Africa Health Research Institute (AHRI), KwaZulu-Natal, South Africa
[10] MRC/Wits Rural Public Health and Health Transitions Research Unit (Agincourt), School of Public Health, Faculty of Health Sciences, University of the Witwatersrand, Johannesburg, South Africa

Correspondence to:
Dr Catherine Porter, Lancaster University Management School, Bailrigg, Lancaster LA1 4YX, United Kingdom, **catherine.porter@lancaster.ac.uk,** +44 7905 760608

Wordcount: 4680 words

For peer review only

ABSTRACT (300 words)

Objective To provide evidence on the effect of the COVID-19 pandemic on the mental health of young people who grew up in poverty in Low and Middle Income Countries (LMICs).

Design A phone survey administered between August-October 2020 to participants of a population-based longitudinal cohort study established in 2002 comprising two cohorts born in 1994-5 and 2001-2 in Ethiopia, India (Andhra Pradesh and Telangana), Peru and Vietnam. We use logistic regressions to examine associations between mental health and pandemic-related stressors, structural factors (gender, age); and lifelong protective/risk factors (parent and peer relationship, wealth, long-term health problems, past emotional problems, subjective well-being) measured at younger ages.

Setting A geographically diverse, poverty-focused sample, also reaching those without mobile phones or internet access.

Participants 10,496 individuals were approached; 9,730 participated. Overall, 8,988 individuals were included in this study; 4,610 (51%) males and 4,378 (49%) females. Non-inclusion was due to non-location or missing data.

Main outcome measures Symptoms consistent with at least mild anxiety or depression were measured by Generalized Anxiety Disorder-7 (GAD-7, ≥5) or Patient Health Questionnaire-8 (PHQ-8, ≥5).

Results Rates of symptoms of at least mild anxiety (depression) were highest in Peru at 41% (32%) [95% CI, 38.63-43.12; (29.49-33.74)], and lowest in Vietnam at 9% (9%) [95% CI, 8.16- 10.58; (8.33- 10.77)], mirroring COVID-19 mortality rates. Females were most affected in all countries except Ethiopia. Pandemic-related stressors such as health risks/expenses, economic adversity, food insecurity, educational or employment disruption were risk factors for anxiety and depression, though showed varying levels of importance across countries. Prior parent/peer relationships were protective factors, while long-term health or emotional problems were risk factors.

Conclusion Pandemic-related health, economic and social stress presents significant risks to the mental health of young people in LMICs where mental health support is limited, but urgently needed to prevent long-term consequences.

Strengths and limitations of this study

- The study uses data from adolescents and young adults who grew up in poverty in four LMICs which were diversely affected by the COVID-19 pandemic, therefore investigating a globally vulnerable, but understudied group both in terms of age and wealth.
- This study reaches a broad sample of young people who grew up in poverty, including those without internet or direct access to a mobile phone.
- A key strength is combining a broad range of pandemic-related stressors from survey data on experiences of COVID-19 with previously measured information on longer-term risk and protective factors, therefore contributing to a more complete picture of COVID-19 effects.
- A limitation of the study is that it does not have a directly comparable pre-COVID baseline for depression/anxiety, however, proxy variables are used as a baseline and the explanatory variables capture dynamics that happened during the pandemic.
- A further limitation is possible underreporting due to stigma associated with mental health, despite piloting and validation, as well as possible bias in self-reported experiences of pandemic-related stressors due to feelings of anxiety or depression.

For peer review only - http://bmjopen.bmj.com/site/about/guidelines.xhtml

INTRODUCTION

The Coronavirus disease 2019 (COVID-19) is creating concerns about the mental health of young people around the globe. There has been a call for research funders and researchers to "deploy resources to understand the psychological effects"[1] of the COVID-19 pandemic and the ensuing "mental health crisis".[2] The crisis likely exacerbates previous risk factors of poverty and vulnerability. The Lancet Commission on Global Mental Health had already identified poverty as a key risk factor for the onset and persistence of mental disorders. [3] A recent study[4] found that those with the lowest income were much more likely to suffer from anxiety and depressive disorders than their wealthier counterparts and point to the bidirectional causal relationship between poverty and mental health.

Several studies have examined the mental health impacts of the pandemic, predominantly in high-income countries.[5,6,7] The few studies from Low-and-Middle-income countries (LMICs) have primarily relied on convenience samples, and internet-based surveys (e.g.[8-11]), which are unlikely to reach the rural poor, though one study[12] investigated the effect of immediate lockdown orders on (adult) women's mental health and experiences of intimate partner violence using a phone survey in rural Bangladesh.

Half of all mental health conditions develop by 14 years of age and 75% by early adulthood.[3] In developed countries young women aged 16-24 are the most likely to have experienced a deterioration in mental health during the pandemic.[13] Thus, understanding risk and protective factors during the pandemic at this age is critical to prevention, especially for the poorest. There is little research on the mental health of adolescents in LMICs, though they make up the bulk of the global adolescent population.[14]

This study examines the impact of the COVID-19 pandemic on the mental health of nearly 10,000 young people from a 20-year cohort study operating in four LMICs: Ethiopia, India (Andhra Pradesh and Telangana), Peru and Vietnam. When the cohorts were originally recruited, the objective was to ensure that families living in poverty were substantially represented. [15-18] Nowadays, these countries represent a diverse set of experiences during the pandemic, in terms of number and severity of cases, as well as policy responses. Figure 1 shows that COVID-19 has had by far the most striking impact in Peru in terms of deaths per population, followed by India. In contrast, Vietnam has been hailed as a success story in controlling the spread of the virus.

Our structural framework is a set of three hypothesised channels through which the pandemic may affect mental health, which are illustrated in Figure 2. The first, is the country level environment. Pressure on mental health is likely to be greater in countries which are more affected by the pandemic. Second, within each country, stressors related to changes in circumstances/behaviours/wellbeing that occurred due to the pandemic are hypothesised to negatively affect mental health[19 20], which we term as *COVID-19 related stressors.* These include individuals' perceived infection risk, economic adversities, changes in employment status and increased household responsibilities, educational disruption, and changes in subjective well-being between 2016 and the pandemic. Third, we consider individual, household, and contextual *background characteristics* which may be protective and/or interact negatively with pandemic-related stressors. We hypothesised that residents of urban areas may have difficulties social distancing in slum-like conditions and are possibly more likely than residents in rural areas to develop mental health conditions. Similarly, depression and anxiety symptoms have been shown to increase more among females than males in other countries[5 21]. Changes imposed by COVID-19 in time use[22], education and

For peer review only - http://bmjopen.bmj.com/site/about/guidelines.xhtml

work may also impact the genders differently[23 24]. Further, we exploited information collected in previous survey rounds to investigate characteristics measured during childhood and adolescence: positive social interactions (parent and peer relations), household wealth, and long-term health conditions. We also include *proxy baseline information for mental health* in the form of past emotional problems and subjective well-being measured 10 years ago at age 15. Our data and methods allow us to discuss the first hypothesis and to directly test the second and third hypotheses

METHODS

Study design and participants

A phone survey[25-28] (see Supplementary documents) was administered between August and October 2020 as part of the Young Lives study[29], a longitudinal survey established in 2002 following two cohorts of children born in 1994-5 and 2000-1 in Ethiopia, India (Andhra Pradesh and Telangana), Peru and Vietnam[a]

For peer review only

[a] The choice to focus on these four countries was made by the original study team in 2001. The Young Lives study was designed to monitor the effectiveness of the Millennium Development Goals (2000-2015) in reducing childhood poverty in varied political-economic and socio-cultural settings. This led to the selection of four study countries, reflecting a diverse set of geography and development stages.[29]

For peer review only - http://bmjopen.bmj.com/site/about/guidelines.xhtml

Respondents have been surveyed in person every three years, with five consecutive rounds completed by 2016. In 2020, the cohort members are aged 18-19 (younger cohort) and 25-26 years (older cohort). The original sample was selected to include a significant coverage of poorer areas.[15-18] 93% of the cohort (9,704) were tracked in 2019. The sample was reduced to 8,988 individuals due to missing values for any question (Supplementary Table 1 and Supplementary Table 2) including between 0.1% (Vietnam) and 2% (Peru) who did not respond to the mental health questions (Supplementary Table 3).

The phone survey was administered over mobile telephone by up to 15 trained interviewers per country, who were provided with the hardware, software and internet access required for working from home. In Ethiopia, roughly 51% of participants did not have access to the internet via a working smartphone or a home computer. Thus, we used local guides who provided sanitised mobile phones for those who did not have them. Responses were recorded in an electronic questionnaire using *Surveybe* Implementer software.

Symptoms of anxiety and depression were measured using the Generalized Anxiety Disorder-7 (GAD-7) scale and the Patient Health Questionnaire depression scale-8 (PHQ-8). The GAD-7 has been validated[30] and used in all four study countries.[31-36] The PHQ-9 was also validated[37-41] and used in several studies.[33-36 42-47] The 9th question of PHQ was dropped due to ethical concerns about how to provide support. The scales were slightly adapted for administration in a phone survey. First, we asked participants whether they were alone in the room and if not, whether they could find a quiet space and/or make sure their phone speaker was off. Second, for each item in GAD-7 and PHQ-8 we asked whether the symptom had been observed (Yes/No) over the past 14 days, and if "Yes" we then asked about the frequency. The scales were administered as the last section of the survey.

GAD-7 scores between 5 and 9, 10 and 14 and above 15 represent mild, moderate, and severe anxiety, respectively.[48] A PHQ-8 score between 5 and 9, 10 and 14, 15 and 19 and above 19 were considered representative of mild, moderate, moderately severe, and severe depression, respectively.[49] Cronbach's Alpha[50] for both scales was close to or above 0.7.[51] Inter-item correlations fell within the recommended range (0.15 to 0.50[52 53]; Supplementary Table 4).

Statistical analysis

In Table 1 we present t-tests to show differences between groups (e.g., male/female, urban/rural). Logistic regressions were used to examine the relationship between a range of stressors on a binary variable indicating (at least) mild anxiety (GAD-7≥5) and (at least) mild depression (PHQ-8 ≥5), as reported in the Results section. We include two sets of stressors hypothesised to be associated with mental health: *changes in circumstances/behaviours/wellbeing that occurred due to the pandemic (COVID-19 related stressors)* and*, background characteristics* that might act as risk or protective factors. We also include *proxy baseline information* (emotional problems and subjective wellbeing measured 10 years earlier). The characteristics of the sample population are shown in Supplementary Table 5. Supplementary Figure 1 gives an overview of the variables used in the analysis and the respective ages when they were measured.

The first set of *COVID-19 related stressors* include perceived COVID-19 infection risk, the extent to which people practice self-isolation (having left the house in the past 7 days), increased household responsibilities (including spending more time caring for children, on household chores or working in a family business), suffering from any adverse economic events (including increases in the price of food, incurring increased health expenditures, fewer clients in a family business, and if so, whether the

For peer review only - http://bmjopen.bmj.com/site/about/guidelines.xhtml

household reduced food consumption to cope with it) and changes in working status compared to before the pandemic. In further analysis, for the 19-year-old cohort, we replaced working status with engagement with education, given that more than half of this group were still enrolled when the pandemic began (Supplementary tables 6 and 7). Finally, among the *COVID-19 related stressors*, we included the change in subjective well-being (SWB) between 2016 and the pandemic. SWB was measured in Round 5 (2016) at ages 15 and 22, and in the phone survey. Cantril's Ladder (1965)[54] asks respondents to visualise a ladder of nine steps; the bottom (top) step representing their worst (best) possible life. Respondents are asked to identify which step they presently stand on. The difference *in subjective well-being* is a continuous variable ranging from -8 to +8.

The second set of *background characteristics* include *individual and household-level risk and protective factors: Gender, age, location,* as well as long-term health problems and past household wealth[55], both measured in 2016, and Parent-child and Peer-child relationships, measured using the total raw scores of the Marsh Self-Description Questionnaires II[56] and I[57]. Both scores range between 8 and 32, with higher scores representing more positive relationships. Peer relationships were obtained at ages 15 (younger cohort) and 22 (older cohort) in 2016. Parent relationships were obtained at ages 15 (younger cohort, 2016) and 19 (older cohort, 2013).

GAD-7 and PHQ-8 were not measured in previous survey rounds. Therefore, we control for p*roxy baseline information* including emotional problems and SWB*,* both available for the 25-year-old cohort only in 2009 (round 3) at the age of 15 (Supplementary tables 8 and 9). The Emotional Problem Scale comes from the self-completed, Strengths and Difficulties Questionnaire (SDQ). Total scores range from 0 to 10, a higher score indicates more severe emotional problems. We report extensive disaggregated rates of mental health issues (Supplementary table 10 to supplementary table 15).

Changes in responsibilities, the labour market and education environment may affect males and females differently. Therefore, we re-estimated the regressions separately by gender (Supplementary table 16 to supplementary table 23). We report odds ratios, robust standard errors and the 95% confidence intervals for all regressions.

Ethics statement

The survey was approved by the institutional research ethics committees at the University of Oxford (UK), the University of Addis Ababa (Ethiopia), the Centre for Economic and Social Studies in Hyderabad (India), the Instituto de Investigación Nutricional (Peru) and the Hanoi University of Public Health (Vietnam). Participants were asked for their verbal informed consent before the study commenced and were assured of confidentiality. A consultation guide was provided to all participants with resources for support in issues raised by the questionnaire, including mental health. Approval number (Oxford): CUREC 1A/ ODID CIA-20-034.

Patient and public involvement

No patients or the public were involved in the study design, setting the research questions, interpretation or writing up of results, or reporting of the research as it is a prospective cohort study.

RESULTS

For peer review only - http://bmjopen.bmj.com/site/about/guidelines.xhtml

Across the four countries, 93% of the Young Lives sample, who were located during the last tracking exercise in November 2019, participated in the phone survey. Only between 0.1% (Vietnam) and 2% (Peru) did not respond to the mental health questions. The results presented in this section refer to the 19-year-olds and 25-year-olds together, unless differently specified.

Country comparisons

Both mild anxiety (41%, 95% CI 38.63-43.12) and mild depression (32%, 29.49-33.74) rates were highest in Peru, followed by Ethiopia (anxiety: 18%, 16.28-19.54; depression: 15%, 13.95- 17.02), see Table 1. The rates of moderate/severe anxiety and depression were highest in Peru, 13.5% (95% CI 12.00-15.14) and 9.6% (8.35-11.07), and below 3% in the other countries (Supplementary tables 24 to 31). Females had significantly higher rates of anxiety symptoms in all countries except Ethiopia and higher rates of depression in Peru and Vietnam. In Peru, almost half of all females had symptoms consistent with at least mild anxiety. Rates of anxiety and depression in rural areas were significantly lower than urban rates in Ethiopia and Peru, but significantly higher in India. The poorest wealth tercile had significantly lower rates of anxiety in Ethiopia and Peru, but higher rates in India and Vietnam. In Peru, the poorest wealth tercile also had significantly lower rates of depression. Those not having any access to the internet, although a minority, had significantly higher levels of anxiety (Vietnam p<0.01, Peru p<0.1).

We note a high correlation between GAD-7 and PHQ-8 scores[b] (minimum 0.610 (p <0.01) (India) and maximum 0.700 (p <0.01) (Peru), and the rate of having both (at least mild) anxiety and depression symptoms was high, with values of up to 24.8% (95% CI 22.87- 26.81) in Peru (Supplementary Table 32 and 33).

The significant risk and protective factors were similar. For brevity, the main results refer to associations with experiencing at least mild anxiety symptoms (see Table 2), pooling the two cohorts and females and males together (unless differently specified). At the end, we comment on the differences between these results and those for depression (see Table 3).

---

[b] We use Pearson's correlation coefficient with Bonferroni corrected p-values to investigate i) the relationship between GAD-7 and PHQ-8 raw scores and ii) the relationship between subjective well-being (SWB) and GAD-7 and PHQ-8 raw scores

For peer review only - http://bmjopen.bmj.com/site/about/guidelines.xhtml

Table 1. Rates of at least mild depression and anxiety in Ethiopia, India, Peru and Vietnam

Rates of at least mild anxiety	Ethiopia		India		Peru		Vietnam	
	% at least mild Anxiety	CI95%	% at least mild Anxiety	CI95%	% at least mild Anxiety	CI95%	% at least mild Anxiety	CI95%
**Total**	**17.87**	**16.28; 19.54**	**11.06**	**9.88; 12.32**	**40.86**	**38.63; 43.12**	**9.32**	**8.16; 10.58**
Male	17.48	15.30; 19.65	9.28***	7.74; 10.81	33.65***	30.65; 36.64	7.92**	6.33; 9.51
Female	18.32	15.93; 20.70	13.01	11.14; 14.87	48.28	45.06; 51.49	10.63	8.88; 12.39
Rural	14.67***	12.65; 16.70	11.81*	10.34; 13.27	33.71***	28.79; 38.63	9.07	7.47; 10.67
Urban	21.61	19.06; 24.16	9.20	7.13; 11.27	42.52	40.04; 45	9.62	7.83; 11.40
Poorest tercile	14.90**	12.29; 17.51	13.52***	11.26; 15.78	36.92**	33.01; 40.84	11.67***	9.41; 13.92
Middle/Richest terciles	19.32	17.29; 21.34	9.82	8.42; 11.21	42.63	39.94; 45.32	8.11	6.74; 9.49
No internet	15.97**	13.82; 18.11	12.16	8.43; 15.89	51.32*	40; 62.63	22.86***	8.74; 36.98
With internet	19.87	17.47; 22.27	10.92	9.65; 12.19	40.42	38.16; 42.68	9.11	7.92; 10.30
**Rates of at least mild depression**	**% at least mild Depression**	**CI95%**	**% at least mild Depression**	**CI95%**	**% at least mild Depression**	**CI95%**	**% at least mild Depression**	**CI95%**
**Total**	**15.44**	**13.95; 17.02**	**9.92**	**8.80; 11.12**	**31.58**	**29.49; 33.74**	**9.49**	**8.33; 10.77**
Male	14.92	12.88; 16.96	9.64	8.08; 11.21	27.38***	24.55; 30.21	7.65***	6.09; 9.22
Female	16.04	13.77; 18.31	10.22	8.54; 11.89	35.91	32.83; 39.00	11.22	9.42; 13.02
Rural	12.89***	10.98; 14.81	10.84**	9.43; 12.25	26.40**	21.82; 30.99	9.39	7.77; 11.01
Urban	18.43	16.03; 20.83	7.60	5.70; 9.50	32.79	30.44; 35.14	9.62	7.83; 11.40
Poorest tercile	14.35	11.78; 16.91	10.80	8.74; 12.85	26.15***	22.59; 29.72	8.46	6.51; 10.42
Middle/Richest terciles	15.97	14.10; 17.85	9.47	8.10; 10.85	34.02	31.45; 36.60	10.03	8.51; 11.54
No internet	14.09*	12.06; 16.13	10.14	6.69; 13.58	31.58	21.05; 42.11	14.29	2.52; 26.05
With internet	16.85	14.60; 19.11	9.89	8.67; 11.10	31.58	29.44; 33.73	9.42	8.22; 10.63
N	2183		2622		1887		2296	

*Notes: If any missing answers to questions then the whole score is set to missing. *** p<0.01; ** p<0.05; *p<0.1. Stars represent significance of t-test of equality between groups (Male-Female; Rural-Urban; Bottom-Top/Middle wealth tercile; Internet access through home computer/working smartphone (No-Yes)). Poorest and middle/richest terciles refer to the household's position in the 2016 (round 5) wealth distribution. Internet access refers to having internet access through a home computer and/or a working smartphone.*

For peer review only

Table 2. Logistic regression results: Symptoms of at least mild anxiety

	Ethiopia		India		Peru		Vietnam	
	**Odds Ratio**	**95% CI**	**Odds Ratio**	**95% CI**	**Odds Ratio**	**95% CI**	**Odds Ratio**	**95% CI**
COVID-19 related stressors								
Risk perception: believe they are at medium/high risk	0.813 (0.11)	[0.629,1.052]	1.265* (0.16)	[0.980,1.633]	1.460*** (0.15)	[1.200,1.777]	1.022 (0.19)	[0.717,1.459]
Left house for any reason in the past 7 days	0.817 (0.20)	[0.501,1.332]	1.401* (0.27)	[0.955,2.056]	0.994 (0.13)	[0.776,1.274]	1.054 (0.25)	[0.666,1.668]
Difference in subjective well-being R5-Call2	1.070** (0.03)	[1.012,1.133]	1.099** (0.04)	[1.020,1.184]	1.105*** (0.03)	[1.053,1.159]	1.181*** (0.05)	[1.081,1.291]
Change in responsibilities:								
Spend more time taking care of children	0.891 (0.14)	[0.652,1.219]	2.209*** (0.37)	[1.593,3.063]	1.354*** (0.14)	[1.100,1.667]	1.401* (0.25)	[0.986,1.991]
Spend more time on household chores	1.059 (0.16)	[0.794,1.414]	0.756* (0.11)	[0.565,1.011]	1.204 (0.15)	[0.943,1.537]	1.209 (0.19)	[0.888,1.645]
Spend more time working in the family business	0.772 (0.16)	[0.520,1.149]	1.605* (0.46)	[0.916,2.811]	1.288* (0.18)	[0.976,1.699]	1.790*** (0.37)	[1.196,2.679]
Economic adversities								
Faced with new health expenses	0.937 (0.18)	[0.646,1.359]	1.046 (0.14)	[0.798,1.370]	1.733*** (0.19)	[1.404,2.139]	1.240 (0.33)	[0.733,2.098]
Experienced adversity but did not reduce food consumption	2.362*** (0.49)	[1.567,3.561]	0.621 (0.28)	[0.253,1.524]	2.495** (1.00)	[1.140,5.461]	1.624*** (0.28)	[1.161,2.270]
Reduced food consumption as response to experienced adversity	7.187*** (1.71)	[4.510,11.454]	0.871 (0.46)	[0.310,2.446]	2.402** (1.06)	[1.009,5.718]	1.673** (0.37)	[1.089,2.570]
Changes in employment status								
Did not work before the pandemic, but is working now	2.667*** (0.64)	[1.671,4.259]	0.993 (0.22)	[0.643,1.533]	0.866 (0.16)	[0.609,1.232]	1.104 (0.35)	[0.594,2.053]
Worked before the pandemic and is working now/has a job	1.570*** (0.24)	[1.156,2.132]	1.288 (0.22)	[0.925,1.795]	1.068 (0.15)	[0.815,1.399]	0.978 (0.20)	[0.653,1.464]
Worked before the pandemic and is not working now/does not have a job	2.294*** (0.45)	[1.559,3.376]	2.504*** (0.65)	[1.501,4.175]	1.205 (0.18)	[0.893,1.628]	1.346 (0.33)	[0.834,2.171]
Educational disruption								
Enrolled in/Planning to enrol in full time education and not participating in learning activities[a]	1.588** (0.33)	[1.052,2.396]	1.043 (0.21)	[0.707,1.538]	0.600 (0.27)	[0.245,1.471]	0.828 (0.25)	[0.458,1.495]
Enrolled in/Planning to enrol in full time education and participating in learning activities[a]	1.110 (0.38)	[0.565,2.182]	0.710 (0.17)	[0.450,1.120]	1.026 (0.12)	[0.816,1.290]	0.699* (0.15)	[0.465,1.051]
Background characteristics								
Age in months	1.008*** (0.00)	[1.005,1.011]	1.001 (0.00)	[0.998,1.005]	0.999 (0.00)	[0.997,1.002]	0.995** (0.00)	[0.991,0.999]
Female	1.074 (0.16)	[0.810,1.426]	1.601*** (0.27)	[1.149,2.231]	1.706*** (0.18)	[1.389,2.095]	1.295* (0.20)	[0.961,1.746]

For peer review only - http://bmjopen.bmj.com/site/about/guidelines.xhtml

	Ethiopia		India		Peru		Vietnam	
	Odds Ratio	95% CI	Odds Ratio	95% CI	Odds Ratio	95% CI	Odds Ratio	95% CI
Urban	1.276* (0.18)	[0.974,1.670]	0.903 (0.15)	[0.658,1.238]	1.441** (0.22)	[1.072,1.937]	1.206 (0.18)	[0.895,1.625]
Participant has long-term health problem, 2016 (Round 5)	1.424* (0.27)	[0.984,2.060]	1.399* (0.25)	[0.989,1.981]	1.805*** (0.26)	[1.354,2.406]	1.337 (0.27)	[0.897,1.991]
Total parent-child relationship score, 2012/2016 (Round 4/5)	0.967 (0.02)	[0.926,1.009]	0.923*** (0.02)	[0.883,0.965]	0.976* (0.01)	[0.949,1.004]	1.041 (0.03)	[0.992,1.093]
Total peer-child relationship score, 2016 (Round 5)	0.941*** (0.02)	[0.901,0.982]	1.025 (0.03)	[0.975,1.078]	1.001 (0.02)	[0.970,1.033]	0.941* (0.03)	[0.876,1.011]
Middle/Top wealth tercile R5, 2016 (Round 5)	1.124 (0.16)	[0.846,1.495]	0.781* (0.11)	[0.587,1.038]	1.016 (0.13)	[0.797,1.295]	0.550*** (0.09)	[0.404,0.747]
Proxy baseline information								
Emotional problem scale (EPS) score, 2009 (Round 3)[b]	1.074* (0.04)	[0.994,1.161]	1.007 (0.05)	[0.910,1.114]	1.222*** (0.07)	[1.093,1.366]	1.072 (0.07)	[0.940,1.221]
Subjective well-being, 2009 (Round 3)[b]	0.979 (0.06)	[0.872,1.100]	1.131** (0.07)	[1.010,1.267]	0.924 (0.07)	[0.800,1.068]	0.975 (0.08)	[0.827,1.148]
N	2183		2622		1887		2296	

*Note: Odds ratios are unadjusted odds ratios. Robust standard errors in parenthesis, *** significant at 1%, ** significant at 5%, * significant at 10%. Base categories are as follows: Believe they are at no/low risk, Did not leave the house at all during the past 7 days, Did not spend more time taking care of children, Did not spend more time on household chores, Did not spend more time working in the family business, Did not face new health expenses, Did not suffer a shock, Did not work at all in the past 12 months OR worked during the pandemic but not before and is not working now, Never attended school or not enrolled in full-time education/not planning to enrol, Male, Rural, Does not have a long-term health condition, Lowest wealth tercile. All time-variant variables are measured in 2020 unless otherwise specified. The regression was run on the joint Younger Cohort /Older Cohort sample except for the following results which were added from independent regression specifications run on the Younger and Older Cohort only:*

[a]results come from a separate regression where Educational disruption was substituted for Changes in employment status and which was run on the Younger Cohort (18-19) sample only (Supplementary Table 6).

[b] results come from a separate regression where we added proxy baseline information measured in 2009 at age 15 and which was run on the Older Cohort (25-26) sample only (Supplementary Table 8)

Table 3. Logistic regression results: Symptoms of at least mild depression

	Ethiopia		India		Peru		Vietnam	
	Odds Ratio	95% CI	Odds Ratio	95% CI	Odds Ratio	95% CI	Odds Ratio	95% CI
COVID-19 related stressors								
Risk perception: believe they are at medium/high risk	0.620*** (0.08)	[0.481,0.800]	0.962 (0.13)	[0.736,1.258]	1.595*** (0.17)	[1.297,1.963]	1.107 (0.20)	[0.778,1.575]
Left house for any reason in the past 7 days	0.995 (0.26)	[0.596,1.661]	2.285*** (0.53)	[1.454,3.590]	1.055 (0.14)	[0.813,1.368]	0.965 (0.21)	[0.629,1.480]

	Ethiopia		India		Peru		Vietnam	
	Odds Ratio	95% CI	Odds Ratio	95% CI	Odds Ratio	95% CI	Odds Ratio	95% CI
Difference in subjective well-being R5-Call2	1.089*** (0.03)	[1.026,1.155]	1.119*** (0.04)	[1.041,1.202]	1.080*** (0.03)	[1.026,1.137]	1.163*** (0.05)	[1.068,1.267]
Change in responsibilities:								
Spend more time taking care of children	0.852 (0.14)	[0.623,1.165]	1.611** (0.30)	[1.121,2.316]	1.145 (0.13)	[0.919,1.425]	1.455** (0.26)	[1.021,2.074]
Spend more time on household chores	1.128 (0.17)	[0.834,1.526]	0.877 (0.14)	[0.649,1.186]	1.061 (0.14)	[0.821,1.371]	1.550*** (0.25)	[1.133,2.119]
Spend more time working in the family business	0.948 (0.20)	[0.633,1.419]	1.053 (0.35)	[0.551,2.011]	1.302* (0.19)	[0.975,1.738]	1.804*** (0.35)	[1.228,2.650]
Economic adversities								
Faced with new health expenses	0.522*** (0.11)	[0.342,0.796]	1.289* (0.19)	[0.967,1.716]	1.739*** (0.19)	[1.401,2.159]	0.909 (0.26)	[0.523,1.580]
Experienced adversity but did not reduce food consumption	3.808*** (0.95)	[2.336,6.206]	0.646 (0.32)	[0.244,1.713]	2.049* (0.86)	[0.896,4.686]	1.695*** (0.30)	[1.203,2.390]
Reduced food consumption as response to experienced adversity	10.890*** (3.00)	[6.341,18.701]	0.641 (0.38)	[0.203,2.027]	2.531** (1.17)	[1.019,6.285]	1.907*** (0.41)	[1.249,2.912]
Changes in employment status								
Did not work before the pandemic, but is working now	2.657*** (0.64)	[1.655,4.265]	0.685* (0.16)	[0.438,1.072]	0.920 (0.17)	[0.643,1.316]	0.715 (0.24)	[0.375,1.363]
Worked before the pandemic and is working now/has a job	1.378** (0.22)	[1.010,1.881]	0.873 (0.15)	[0.620,1.229]	0.841 (0.12)	[0.634,1.114]	0.810 (0.16)	[0.552,1.190]
Worked before the pandemic and is not working now/does not have a job	1.679** (0.36)	[1.104,2.555]	1.597 (0.45)	[0.914,2.790]	1.283 (0.20)	[0.940,1.751]	1.509* (0.34)	[0.974,2.336]
Educational disruption								
Enrolled in/Planning to enrol in full time education and not participating in learning activities[a]	1.728*** (0.36)	[1.153,2.592]	1.103 (0.22)	[0.743,1.638]	0.743 (0.36)	[0.287,1.928]	0.944 (0.28)	[0.529,1.685]
Enrolled in/Planning to enrol in full time education and participating in learning activities[a]	1.174 (0.41)	[0.591,2.333]	0.749 (0.18)	[0.469,1.196]	1.042 (0.13)	[0.816,1.329]	1.172 (0.25)	[0.768,1.788]
<u>*Background characteristics*</u>								
Age in months	1.001 (0.00)	[0.998,1.005]	1.000 (0.00)	[0.997,1.004]	0.996** (0.00)	[0.993,0.999]	0.994*** (0.00)	[0.989,0.998]
Female	1.142 (0.17)	[0.847,1.541]	1.239 (0.21)	[0.890,1.725]	1.317** (0.15)	[1.059,1.637]	1.405** (0.21)	[1.048,1.883]
Urban	1.356** (0.20)	[1.019,1.805]	0.733* (0.12)	[0.526,1.022]	1.114 (0.17)	[0.820,1.513]	0.961 (0.15)	[0.706,1.308]
Participant has long-term health problem, 2016 (Round 5)	0.954 (0.21)	[0.620,1.470]	1.566** (0.28)	[1.101,2.226]	1.590*** (0.23)	[1.197,2.113]	1.328 (0.27)	[0.889,1.982]
Total parent-child relationship score, 2012/2016 (Round 4/5)	1.004 (0.02)	[0.960,1.050]	0.909*** (0.02)	[0.867,0.954]	0.939*** (0.01)	[0.911,0.968]	0.983 (0.02)	[0.938,1.031]
Total peer-child relationship score, 2016 (Round 5)	0.938*** (0.02)	[0.895,0.983]	1.015 (0.03)	[0.963,1.070]	1.007 (0.02)	[0.973,1.042]	0.988 (0.04)	[0.920,1.062]

For peer review only

For peer review only - http://bmjopen.bmj.com/site/about/guidelines.xhtml

	Ethiopia		India		Peru		Vietnam	
	**Odds Ratio**	**95% CI**	**Odds Ratio**	**95% CI**	**Odds Ratio**	**95% CI**	**Odds Ratio**	**95% CI**
Middle/Top wealth tercile R5, 2016 (Round 5)	0.884 (0.13)	[0.656,1.192]	1.074 (0.16)	[0.802,1.437]	1.249* (0.16)	[0.968,1.613]	1.068 (0.18)	[0.770,1.482]
Proxy baseline information								
Emotional problem scale (EPS) score, 2009 (Round 3)[b]	1.090* (0.05)	[0.995,1.195]	1.101* (0.06)	[0.991,1.223]	1.129** (0.07)	[1.003,1.270]	1.114 (0.08)	[0.973,1.276]
Subjective well-being, 2009 (Round 3)[b]	0.894 (0.06)	[0.782,1.023]	1.077 (0.07)	[0.952,1.219]	0.869* (0.07)	[0.736,1.026]	0.952 (0.08)	[0.805,1.126]
N	2183		2622		1887		2296	

*Note: Odds ratios are unadjusted odds ratios. Robust standard errors in parenthesis, *** significant at 1%, ** significant at 5%, * significant at 10%. Base categories are as follows: Believe they are at no/low risk, Did not leave the house at all during the past 7 days, Did not spend more time taking care of children, Did not spend more time on household chores, Did not spend more time working in the family business, Did not face new health expenses, Did not suffer a shock, Did not work at all in the past 12 months OR worked during the pandemic but not before and is not working now, Never attended school or not enrolled in full-time education/not planning to enrol, Male, Rural, Does not have a long-term health condition, Lowest wealth tercile. All time-variant variables are measured in 2020 unless otherwise specified. The regression was run on the joint Younger Cohort /Older Cohort sample except for the following results which were added from independent regression specifications run on the Younger and Older Cohort only:*

[a]results come from a separate regression where Educational disruption was substituted for Changes in employment status and which was run on the Younger Cohort (19-20) sample only (Supplementary Table 7).

[b] results come from a separate regression where we added proxy baseline information measured in 2009 at age 15 and which was run on the Older Cohort (25-26) sample only (Supplementary Table 9).

For peer review only - http://bmjopen.bmj.com/site/about/guidelines.xhtml

Logistic regression results (odds ratios): At least mild anxiety

COVID-related stressors

COVID-19 infection risk perception: The odds of those who believed that they were at medium/high risk of catching the virus were 1.27 (95% CI 0.98 - 1.63, p<0.1) (India) to 1.46 (1.20 - 1.78, p<0.01) (Peru) times higher than for those who believed themselves to have no/low risk. The former group had rates of at least mild anxiety of 12% (India) and 45% (Peru).

Leaving the house for at least one day a week: No significant effects, except in India where it increased the odds of anxiety by 1.40 (95% CI, 0.96-2.06, p<0.1).

Economic adversity: For those who suffered from economic adversity (e.g. fewer clients in the family business, food price increase) odds of anxiety were higher (p<0.01 (Ethiopia and Vietnam), (2.50, 95% CI 1.14-5.46, p<0.05) (2.40, 1.01-5.72, p<0.05) (Peru)) even if it did not cause reduced food consumption. Moreover, in Ethiopia and Vietnam, those who reduced food consumption as a coping strategy had 7.19 (95% CI 4.51-11.45, p<0.01) (Ethiopia), 1.67 (1.09-2.57, p<0.05) (Vietnam) higher odds than those who experienced an adverse event but did not need to reduce food consumption in response 2.36 (1.57-3.56, p<0.01) (Ethiopia) and 1.62 (1.16 - 2.27, p<0.01) (Vietnam) (both compared to those who did not experience any adverse event at all). In Ethiopia, 36% of those who reduced food consumption reported at least mild anxiety compared to 7% of those who did not experience an adverse event (p<0.0001). In Ethiopia, odds were higher among females than males, but in Peru and Vietnam significant for males only. Facing new health expenses significantly increased the odds by 1.73 (95% CI 1.40 - 2.14) (p <0.01) in Peru. Over half (52%, p- <0.0001) of those who faced new health expenses report at least mild anxiety (although not significant in India or Ethiopia, significant risk factor for females in Vietnam (1.76, 95% CI 0.93-3.31, p<0.1)).

Increased responsibilities: Spending more time on childcare during the lockdown increased odds of anxiety by 2.21 (95% CI 1.59-3.06, p<0.01) in India, 1.35 (1.10-1.67, p<0.01) in Peru and 1.4 (0.99-1.99, p<0.1) in Vietnam. Rates for those who spent more time taking care of children were 20% vs 9% (India), 49% vs 37% (Peru) and 13% vs 8% (Vietnam), (all p<0.001). Spending more time on household chores *lowered* odds in India for anxiety (females only). For those who spend more time working in the family business the odds of anxiety were 1.61 (95% CI 0.92-2.81, p<0.1) times higher in India, 1.29 (0.98-1.67, p<0.1, n.s. for females) in Peru and 1.80 (1.20-2.68, p<0.01) in Vietnam (higher odds among males). In Vietnam, those who spent more time working in the family business reported rates of 16% (p<0.0001), the highest among the Vietnamese sample.

Changes in employment status: In Ethiopia, those who participated in the labour market had higher odds of anxiety than those who did not (e.g. full-time students, stay-at-home parents). However, the odds of those who were pushed into the labour market (2.67, 95% CI 1.67-4.26, p<0.01) or lost their job (2.29, 1.56-3.38, p<0.01) were higher than those who simply participated (1.57, 1.16-2.13, p<0.01) (all in comparison to non-participants). In India, losing a job increased the risk of anxiety by 2.50 (95% CI 1.50-4.18, p<0.01). In Peru and Vietnam, there were no employment effects. Rates of at least mild anxiety among those who lost their jobs were among the highest in each country 31% (Ethiopia, p<0.001), 20% (India, p<0.001), 46% (Peru, n.s.), 12% (Vietnam, n.s.).

For peer review only - http://bmjopen.bmj.com/site/about/guidelines.xhtml

Educational disruption (19-year-old cohort only): Students who were enrolled in Ethiopia before the pandemic and were unable to access virtual classes or complete homework had 1.59 (95% CI 1.05-2.40, p<0.05) times higher odds of anxiety than those who were not enrolled. In Vietnam, those who were enrolled and engaged in learning activities had lower odds of anxiety (0.70, 95% CI 0.47-1.05, p<0.1) than those who were not enrolled (base category). The full YC only regression results can be found in Supplementary Table 6; the education results split by gender (again YC only) are located in Supplementary Tables 20-23.

Background characteristics

For *females*, the odds were 1.30 (95% CI 0.96-1.75, p<0.1) (Vietnam), 1.60 (1.15-2.23, p<0.01) (India) and 1.70 (1.39-2.10, p<0.01) (Peru) times greater than the odds for males (n.s. in Ethiopia). Urban location increased odds significantly in Ethiopia and Peru. Age was not significant in India and Peru, protective in Vietnam, and a risk factor in Ethiopia.

Long-term health problems (measured in 2016): The odds of at least mild anxiety were 1.42 (95% CI 0.98-2.06, p<0.1) (Ethiopia), 1.40 (1.00-2.00, p<0.1) (India) and 1.80 (1.35-2.41, p<0.01) (Peru) times as large as the odds for those who did not (n.s. in Vietnam). In Peru, those reporting long-term health problems had the highest rates of at least mild anxiety, 56% (p<0.0001).

Parent-child relationship (measured at ages 15 for the younger cohort, in 2016 and measured at age 19 for the older cohort, in 2013) *and peer-child relationship* (at ages 15 for the younger cohort and at age 22 for the older cohort, in 2016): Strong parent-child relationships were a significant protective factor in India and Peru, while peer-child relationships were a significant protective factor in Ethiopia and Vietnam.

Past household wealth (measured in 2016): i.e. being in the middle/highest wealth tercile vs the lowest was a marginally significant protective factor in India (0.78, 95% CI 0.59-1.04, p<0.1) and significant in Vietnam (0.55, 0.40-0.75, p<0.01).

Proxy baseline information

Past emotional problems and well-being (25-year-old cohort only): For a one-point increase in previous emotional problems at age 15 (measured in 2009), the odds of at least mild anxiety increased by a factor of 1.22 (1.09 - 1.37, p <0.01) (Peru) and 1.07 (95% CI 0.99 - 1.16, p <0.1) (Ethiopia). Notably, the effect of the COVID-19 related stressors holds when controlling for past proxy baseline information. The full older cohort only regression results can be found in Supplementary Table 8.

Significant differences between anxiety and depression logistic regression results

As previously mentioned, the results for anxiety and depression (see Table 3, Supplementary Tables 7, 9, 16-23, 34-36) are qualitatively similar. Here we note significant differences in the results between the two independent variables for each country. In Ethiopia, food insecurity had a higher impact on depression for males than females. In India, subjective high infection risk increased anxiety, but not depression, and those faced with new health expenses had higher odds of depression, but not anxiety. Females also had higher rates of anxiety but not depression. In Peru, childcare was not a risk factor for depression, and past SWB was a protective factor. In Vietnam, losing a job was a significant risk factor for depression, while good peer relations and education were not significant determinants of depression.

For peer review only - http://bmjopen.bmj.com/site/about/guidelines.xhtml

DISCUSSION

We examined the impact of the COVID-19 pandemic on the mental health of young people in Ethiopia, India, Peru and Vietnam. The sample has broad coverage of the poorer population in each country, and we interviewed 93% of those located during the tracking prior to the pandemic, including those without internet access and in Ethiopia also those without mobile phone, who would be excluded from an online survey. Internet access has had both positive and negative effects in the pandemic[58]. In our sample, those without access to internet have significantly higher rates of anxiety in Vietnam and Peru.

The four countries have had different experiences of the pandemic – Peru is the most affected country in terms of deaths per population, and Vietnam the least. While Vietnam has recorded only 35 deaths in total (not per day), with no deaths since September 3rd, 2020, on October 15th (end date of our survey) Peru had registered 33,577 deaths.[59] Another aspect which has likely contributed to the difference is the length of the lockdown, which creates stress and reduces household income. In Peru, this was 107 days consecutive days at the national level, followed by an additional period of local lockdowns, such that certain areas of the country were in lockdown for up 199 days. In contrast, Vietnam had a very short and successful lockdown, one further localised lockdown, but by September life was already back to normal. Ethiopia restricted certain activities and closed schools, but did not impose a strict lockdown, though faced other challenges (locust infestations, food price inflation and violence).

This study reveals a strong relationship between the severity of the pandemic and the rates of mental health conditions in our sample, both in terms of anxiety and depression symptoms. Rates of at least mild anxiety (depression) were four (three) times higher in Peru compared to Vietnam. Furthermore, the 2020 survey showed a significant fall in subjective well-being from 2016 in all countries except Vietnam. The fall in subjective well-being is highly correlated with anxiety and depression symptoms. In the absence of baseline measures of GAD-7 and PhQ-8, a strong correlation between SWB and our mental health indicators is important, as it suggests that SWB is a useful proxy baseline.

The economic impact of the pandemic has affected certain groups of young people in all study countries, even Vietnam, and Ethiopia where there was no full national lockdown. Overall, our findings confirmed that those experiencing COVID-19 related stressors had worse mental health, although the relative importance of stressors varied across countries: increased health expenses and believing they were at a medium/high infection risk was detrimental for young people in Peru, but increased food insecurity was much more important in Ethiopia, reflecting high rates of food price inflation in 2019, which continued into 2020. Moreover, good peer relations in earlier years were a protective factor for anxiety and depression only in Ethiopia. In Peru and Vietnam, there were no employment effects on anxiety, likely for very different reasons – in Peru health concerns were more important, and in Vietnam, the labour market was relatively resilient.

Exploiting the longitudinal data allowed us to investigate individual and household-level protective and risk factors. As expected, parent and peer relations measured during childhood and adolescence were protective, though in different ways across countries. Strong parental relationships were a significant protective factor in India and Peru, whereas peer relationships were more important in Ethiopia and Vietnam. Those reporting long-term health problems were twice as likely to display symptoms consistent with at least mild anxiety, this effect being particularly pronounced in Peru. Previous relative wealth was a significant protective factor only in India and Vietnam. Pre-pandemic emotional problems were risk

For peer review only - http://bmjopen.bmj.com/site/about/guidelines.xhtml

factors, especially in Ethiopia and Peru. The associations with COVID-19 related stressors were robust to the inclusion of pre-pandemic emotional problems and past subjective well-being.

Other studies have used longitudinal data to document the impact of the pandemic on mental health,[5 12] though none investigate a comparable population of young people, of a similar age to those in our study countries, though results from the UK have similar findings. The closest study to ours, a phone survey in a developing country finds a deterioration in maternal mental health in rural Bangladesh.[12] Our study shows lower rates of anxiety and depression in rural areas in Ethiopia and Peru, but significantly higher in India. We are able to disaggregate the effect of a range of COVID-19 related stressors, which we can relate individually to other studies. A study in Hubei province, China [11], showed the importance of income losses during the pandemic. Studies of college students in China[8] and Bangladesh[10] show that educational disruption significantly increased anxiety and depression, similar to our results in Vietnam and Ethiopia. Social support was negatively correlated with the level of anxiety[8], similar to our findings regarding parent/peer-child relationships. In Jordan[9], female healthcare professionals, female university students and university students with chronic disease were at higher risk of developing depression, similar to our results for long-term health problems.

Even controlling for other factors, we found females to be more vulnerable to anxiety in India, Peru and Vietnam and more vulnerable to depression in Peru and Vietnam. which is similar to most COVID-19 related[5 9 11] and pre-pandemic studies[60 61]. However, in Ethiopia, we found no significant gender effects. Previous studies in Ethiopia had mixed results on gender differences.[62 63] Relatedly, and during the COVID-19 pandemic, one[8] study finds no gender differences among Chinese college students while another[10] finds that male Bangladeshi students had higher depressive symptoms than females.

Strengths and limitations

This study's strength combines survey data about experiences of COVID-19 with long-term information from two cohorts of participants of a population-based cohort study in four LMICs. The study was able to cover the poorest, those without internet (and without mobile phone in Ethiopia), and examine the role of a broad range of pandemic-related stressors and of individual and household-level risk and protective factors. Our study has a number of limitations. We do not have a directly comparable pre-COVID baseline for depression/anxiety. However, we use proxy variables for baseline and our explanatory variables capture dynamics during the pandemic. As for other studies, there may be underreporting in all four countries[64-67] because of stigma associated with mental health, despite piloting and validation. There is evidence in the literature of stigmatization of mental health in all four countries, as in other LMICs meta-analysis[68 69], but nothing to suggest this is associated with the differences we find. Furthermore, our analysis identifies high risk groups *within* each country. Additionally, self-reported variables may be biased due to feelings of anxiety or depression. The findings are not fully generalizable to the whole population of LMICs due to the poverty-focused design and age group, however they broadly represent poor young people in the study countries.

CONCLUSION

Adolescents and young people have been a lower priority for COVID-19 interventions, given the lower rates of hospitalization and death for this age group. This research shows that the pandemic is having important effects on the mental health of certain groups of young people, even in countries with fewer

For peer review only - http://bmjopen.bmj.com/site/about/guidelines.xhtml

cases. Mental health services are very limited in LMICs making it urgent to develop evidence-based and sustainable prevention programmes in response to the pandemic. As a short-term measure, funding for (and awareness of) telephone helplines can be increased, and Cash Transfer Programmes (CTPs) should be expanded or conditionality waived to cover young people hardest hit by the pandemic, and include mobile phone messaging to provide accurate non-stigmatised information about COVID-19 and available mental health support services. This could help break the cycle between poverty and mental illness, lowering the risk of long-term consequences.[70] Further research on mental health in Peru (the country hardest hit by the pandemic among our four countries) should be conducted including estimating the impact on mental health resulting from lockdown length/intensity and COVID-19 cases/deaths on the district level.

For peer review only

FIGURE CAPTIONS

Figure 1. Cumulative confirmed COVID-19 cases per million people in the four Young Lives countries

Source: Johns Hopkins University CSSE COVID-19 Data[71], accessed via our world in data[59]. Last updated 15 December 2020. Testing and challenges in the attribution of the cause of death means that the number of confirmed deaths may not be an accurate count of the true number of deaths from COVID-19.

Figure 2. Theoretical framework of the hypothesized impact of COVID-19 stressors, background characteristics and the country level environment on mental health

(1), (2), and (3) are the channels as discussed in the framework. Red font colour indicates hypothesized risk factors, blue font colour hypothesized protective factors. In case of gender, females are the hypothesized vulnerable group. Urban participants are hypothesized to be more vulnerable than rural residents (location). Black font colour indicates a potential effect in either direction. White font colour refers to outcome variables. (Solid) lines indicates that the variable was measured in previous in-person rounds. Filled sub-circles are categorical/composite variables. Boxes with no fill colour indicate robustness checks to the main framework. Dotted arrows indicate that the variable was only considered implicitly for descriptive statistics. Solid arrows indicate use in logistic regressions. Changes in employment status and changes in the educational status (=educational disruption) are used interchangeably for the Younger Cohort. Mental health was measured using the GAD-7 and PHQ-8 and a cut-off of five reflecting at least mild symptoms of anxiety/depression was chosen.

For peer review only - http://bmjopen.bmj.com/site/about/guidelines.xhtml

Author affiliations

[1] Lancaster University Management School, Bailrigg, Lancaster LA1 4YX, United Kingdom
[2] Oxford Department of International Development, University of Oxford, 3 Mansfield Rd, Oxford OX1 3TB, United Kingdom
[3] Grupo de Análisis para el Desarrollo (GRADE), Av. Grau 915, Barranco, Lima 15063, Peru
[4] Centre for Economic and Social Studies (CESS), Nizamia Observatory Campus, Begumpet, Hyderabad 500016, Telangana, India
[5] Department of Economics, Addis Ababa University (AAU), P.O. Box 150175, Addis Ababa, Ethiopia
[6] Centre for Analysis and Forecasting, Vietnam Academy of Social Sciences, No.1, Lieu Giai Street, Hanoi, Vietnam
[7] Distinguished Professor Joanne and George Miller and Family Endowed Term Chair (BRI), Director, Anxiety and Depression Research Center (ADRC), Director, Innovative Treatment Network, UCLA Depression Grand Challenge, Associate Director, Staglin Family Music Center for Behavioral & Brain Health, Editor-in-Chief, Behaviour Research and Therapy, UCLA Department of Psychology and Department of Psychiatry & Biobehavioral Sciences, University of California, Franz Hall – Box 951563, Los Angeles, CA 90094-1563, United States
[8] Department of Psychiatry, University of Oxford, Warneford Hospital, Oxford OX3 7JX, United Kingdom
[9] Africa Health Research Institute (AHRI), KwaZulu-Natal, South Africa
[10] MRC/Wits Rural Public Health and Health Transitions Research Unit (Agincourt), School of Public Health, Faculty of Health Sciences, University of the Witwatersrand, Johannesburg, South Africa

Acknowledgments We particularly wish to thank the Young Lives respondents and their families for generously giving us their time and cooperation during this difficult time as well as fieldworkers and field managers in the four study countries. We are gratefully indebted to Professor George Patton for his helpful comments. Further, we would like to thank Professor Graham Thornicroft, Dr. Julio Torales, and Dr. Gabriel Teck for their helpful feedback during the review process.

Author Contributions CP and MF conceived the study. CP, MF, DS, and ASJ designed the study. MF, ASJ, RE, TW, LTD led data collection. CP and AH did the statistical analyses. CP, AH and MF wrote the first draft of the Article. AS and MC provided comments and input to the several drafts of the article. MF, DS, ASJ verified the underlying data. AS, MF, CP and AH assessed scale validation and methodology. AH prepared the supplementary files. All authors critically reviewed multiple versions of the manuscript and approved the final version. The authors alone are responsible for the views expressed in this article.

Funding statement Young Lives at Work is funded by UK aid from the Foreign, Commonwealth and Development Office (FCDO), under Department for International Development, UK Government grant number 200425. The funders of the study had no role in study design, data collection, data analysis, data interpretation, or writing of the report. The views expressed are those of the authors. They are not necessarily those of, or endorsed by, the University of Oxford, Young Lives, FCDO. All authors had full access to the anonymized data in the study and had final responsibility for the decision to submit for publication.

Competing interests CP, MF, AH, DS, ASJ, RE, TW and LTD report grants from the Foreign, Commonwealth and Development Office (FCDO), during the conduct of the study. MC and AS have nothing to disclose.

Patient consent for publication Not required.

For peer review only

Ethics approval The survey was approved by the institutional research ethics committees at the University of Oxford (UK), the University of Addis Ababa (Ethiopia), the Centre for Economic and Social Studies in Hyderabad (India), the Instituto de Investigación Nutricional (Peru) and the Hanoi University of Public Health (Vietnam). Participants were asked for their verbal informed consent before the study commenced and were assured of confidentiality. A consultation guide was provided to all participants with resources for support in issues raised by the questionnaire, including mental health.

Data sharing statement The entire individual participant data collected during the phone survey and previous in-person rounds, after de-identification, is available including data dictionaries. Furthermore the questionnaire, attrition reports and the field work manual are available at https://www.younglives.org.uk/ . The data will be available in January 2021, with no end date to anyone who wishes to access the data for any purpose, via the UK Data Archive (study number 8678, DOI: 10.5255/UKDA-SN-8678-1).

For peer review only

For peer review only - http://bmjopen.bmj.com/site/about/guidelines.xhtml

Reference List

1. Holmes EA, O'Connor RC, Perry VH, et al. Multidisciplinary research priorities for the COVID-19 pandemic: a call for action for mental health science. *The Lancet Psychiatry* 2020;7(6):547-60. doi: 10.1016/s2215-0366(20)30168-1
2. WHO. Substantial investment needed to avert mental health crisis. 2020. https://www.who.int/news/item/14-05-2020-substantial-investment-needed-to-avert-mental-health-crisis (accessed 14.05.2020).
3. Patel V, Saxena S, Lund C, et al. The Lancet Commission on global mental health and sustainable development. *The Lancet* 2018;392(10157):1553-98. doi: 10.1016/s0140-6736(18)31612-x
4. Ridley M, Rao G, Schilbach F, et al. Poverty, depression, and anxiety: Causal evidence and mechanisms. *Science* 2020;370(6522) doi: 10.1126/science.aay0214 [published Online First: 2020/12/12]
5. Pierce M, Hope H, Ford T, et al. Mental health before and during the COVID-19 pandemic: a longitudinal probability sample survey of the UK population. *The Lancet Psychiatry* 2020;7(10):883-92. doi: 10.1016/s2215-0366(20)30308-4
6. Pan K-Y, Kok AAL, Eikelenboom M, et al. The mental health impact of the COVID-19 pandemic on people with and without depressive, anxiety, or obsessive-compulsive disorders: a longitudinal study of three Dutch case-control cohorts. *The Lancet Psychiatry* 2020 doi: 10.1016/s2215-0366(20)30491-0
7. Czeisler MÉ, Lane RI, Petrosky E, et al. Mental Health, Substance Use, and Suicidal Ideation During the COVID-19 Pandemic — United States. *MMWR Morb Mortal Wkly Rep 2020* 2020;69:1049–57. doi: 10.15585/mmwr.mm6932a1external
8. Cao W, Fang Z, Hou G, et al. The psychological impact of the COVID-19 epidemic on college students in China. *Psychiatry Res* 2020;287:112934. doi: 10.1016/j.psychres.2020.112934 [published Online First: 2020/04/02]
9. Naser AY, Dahmash EZ, Al-Rousan R, et al. Mental health status of the general population, healthcare professionals, and university students during 2019 coronavirus disease outbreak in Jordan: A cross-sectional study. *Brain Behav* 2020;10(8):e01730. doi: 10.1002/brb3.1730 [published Online First: 2020/06/25]
10. Islam MA, Barna SD, Raihan H, et al. Depression and anxiety among university students during the COVID-19 pandemic in Bangladesh: A web-based cross-sectional survey. *PLoS One* 2020;15(8):e0238162. doi: 10.1371/journal.pone.0238162 [published Online First: 2020/08/28]
11. Li X, Lu P, Hu L, et al. Factors Associated with Mental Health Results among Workers with Income Losses Exposed to COVID-19 in China. *Int J Environ Res Public Health* 2020;17(15) doi: 10.3390/ijerph17155627 [published Online First: 2020/08/08]
12. Hamadani JD, Hasan MI, Baldi AJ, et al. Immediate impact of stay-at-home orders to control COVID-19 transmission on socioeconomic conditions, food insecurity, mental health, and intimate partner violence in Bangladeshi women and their families: an interrupted time series. *The Lancet Global Health* 2020;8(11):e1380-e89. doi: 10.1016/s2214-109x(20)30366-1
13. Banks J, Xu X. The mental health effects of the first two months of lockdown and social distancing during the Covid-19 pandemic in the UK. *IFS Working Paper W20/16* 2020
14. Blum R, Boyden J. Understand young people in low-income countries. *Nature* 2018;544
15. Escobal J, Flores E. An Assessment of the Young Lives Sampling Approach in Peru. Technical Note. Oxford: Young Lives, 2008.
16. Kumra N. An Assessment of the Young Lives Sampling Approach in Andhra Pradesh, India. Technical Note. Oxford: Young Lives, 2008.
17. Nguyen NP. An Assessment of the Young Lives Sampling Approach in Vietnam. Technical Note. Oxford: Young Lives, 2008.

For peer review only - http://bmjopen.bmj.com/site/about/guidelines.xhtml

18. Outes-Leon I, Sanchez A. An Assessment of the Young Lives Sampling Approach in Ethiopia. Technical Note. Oxford: Young Lives, 2008.
19. Ling GHT, Md Suhud NAB, Leng PC, et al. Factors Influencing Asia-Pacific Countries' Success Level in Curbing COVID-19: A Review Using a Social-Ecological System (SES) Framework. *Int J Environ Res Public Health* 2021;18(4) doi: 10.3390/ijerph18041704 [published Online First: 2021/02/14]
20. Al Omari O, Al Sabei S, Al Rawajfah O, et al. Prevalence and Predictors of Depression, Anxiety, and Stress among Youth at the Time of COVID-19: An Online Cross-Sectional Multicountry Study. *Depress Res Treat* 2020;2020:8887727. doi: 10.1155/2020/8887727 [published Online First: 2020/10/17]
21. Khademian F, Delavari S, Koohjani Z, et al. An investigation of depression, anxiety, and stress and its relating factors during COVID-19 pandemic in Iran. *BMC Public Health* 2021;21(1):275. doi: 10.1186/s12889-021-10329-3 [published Online First: 2021/02/05]
22. Favara M, Freund R, Porter C, et al. Young lives, interrupted: Short-term effects of the COVID-19 pandemic on adolescents in low- and middle-income countries. *Covid Economics* 2021;67:172-98.
23. Burki T. The indirect impact of COVID-19 on women. *The Lancet Infectious Diseases* 2020;20(8):904-05. doi: 10.1016/s1473-3099(20)30568-5
24. Reichelt M, Makovi K, Sargsyan A. The impact of COVID-19 on gender inequality in the labor market and gender-role attitudes. *European Societies* 2020;23(sup1):S228-S45. doi: 10.1080/14616696.2020.1823010
25. Scott D, Thuc DL, Hittmeyer A, et al. Listening to Young Lives at Work in Vietnam: Second Call. *Listening to Young Lives at Work Phone Survey* 2020; 2.4. https://www.younglives.org.uk/sites/www.younglives.org.uk/files/YOL-Vietnam-Headlines-PhoneSurvey2-Nov20.pdf (accessed 24.11.2020).
26. Porter C, Woldehanna T, Freund R, et al. Listening to Young Lives at Work in Ethiopia: Second Call. *Listening to Young Lives at Work Phone Survey* 2020; 2.1. https://www.younglives.org.uk/sites/www.younglives.org.uk/files/YOL-Ethiopia-Headlines-PhoneSurvey2-Nov20.pdf (accessed 24.11.2020).
27. Favara M, Ellanki R, Molina MdlÁ, et al. Listening to Young Lives at Work in Andhra Pradesh and Telangana: Second Call. *Listening to Young Lives at Work Phone Survey* 2020; 2.2. https://www.younglives.org.uk/sites/www.younglives.org.uk/files/YOL-India-Headlines-PhoneSurvey2-Nov20.pdf (accessed 24.11.2020).
28. Sánchez A, Cueto S, Mary Penny JL, et al. Listening to Young Lives at Work in Peru: Second Call. *Listening to Young Lives at Work Phone Survey* 2020; 2.3. https://www.younglives.org.uk/sites/www.younglives.org.uk/files/YOL-Peru-Headlines-PhoneSurvey2-Nov20-2%2023rd%20.pdf (accessed 24.11.2020).
29. Barnett I, Ariana P, Petrou S, et al. Cohort profile: the Young Lives study. *Int J Epidemiol* 2013;42(3):701-8. doi: 10.1093/ije/dys082 [published Online First: 2012/05/24]
30. Zhong QY, Gelaye B, Zaslavsky AM, et al. Diagnostic Validity of the Generalized Anxiety Disorder - 7 (GAD-7) among Pregnant Women. *PLoS One* 2015;10(4):e0125096. doi: 10.1371/journal.pone.0125096 [published Online First: 2015/04/29]
31. Gezie LD, Yalew AW, Gete YK, et al. Socio-economic, trafficking exposures and mental health symptoms of human trafficking returnees in Ethiopia: using a generalized structural equation modelling. *Int J Ment Health Syst* 2018;12:62. doi: 10.1186/s13033-018-0241-z [published Online First: 2018/11/06]
32. Dadi AF, Dachew BA, Kisi T, et al. Anxiety and associated factors among prisoners in North West of Amhara Regional State, Ethiopia. *BMC Psychiatry* 2016;16:83. doi: 10.1186/s12888-016-0792-y [published Online First: 2016/04/03]

For peer review only - http://bmjopen.bmj.com/site/about/guidelines.xhtml

33. Hakim A, Tak H, Nagar S, et al. Assessment of prevalence of depression and anxiety and factors associated with them in undergraduate medical students of Dr. S. N. Medical College, Jodhpur. *International Journal Of Community Medicine And Public Health* 2017;4(9) doi: 10.18203/2394-6040.ijcmph20173826
34. Collier KM, Weiss B, Pollack A, et al. Explanatory variables for women's increased risk for mental health problems in Vietnam. *Soc Psychiatry Psychiatr Epidemiol* 2020;55(3):359-69. doi: 10.1007/s00127-019-01761-3 [published Online First: 2019/08/30]
35. Pham Tien N, Pham Thanh T, Nguyen Hanh D, et al. Utilization of mental health services among university students in Vietnam. *International Journal of Mental Health* 2020:1-23. doi: 10.1080/00207411.2020.1816114
36. Pollack AA, Weiss B, Trung LT. Mental health, life functioning and risk factors among people exposed to frequent natural disasters and chronic poverty in Vietnam. *BJPsych Open* 2016;2(3):221-32. doi: 10.1192/bjpo.bp.115.002170 [published Online First: 2016/07/28]
37. Carroll HA, Hook K, Perez OFR, et al. Establishing reliability and validity for mental health screening instruments in resource-constrained settings: Systematic review of the PHQ-9 and key recommendations. *Psychiatry Res* 2020;291:113236. doi: 10.1016/j.psychres.2020.113236 [published Online First: 2020/07/01]
38. Zhong QY, Gelaye B, Rondon MB, et al. Using the Patient Health Questionnaire (PHQ-9) and the Edinburgh Postnatal Depression Scale (EPDS) to assess suicidal ideation among pregnant women in Lima, Peru. *Arch Womens Ment Health* 2015;18(6):783-92. doi: 10.1007/s00737-014-0481-0 [published Online First: 2014/11/06]
39. Gelaye B, Williams MA, Lemma S, et al. Validity of the Patient Health Questionnaire-9 for depression screening and diagnosis in East Africa. *Psychiatry Res* 2013;210(2):653-61. doi: 10.1016/j.psychres.2013.07.015 [published Online First: 2013/08/27]
40. Woldetensay YK, Belachew T, Tesfaye M, et al. Validation of the Patient Health Questionnaire (PHQ-9) as a screening tool for depression in pregnant women: Afaan Oromo version. *PLoS One* 2018;13(2):e0191782. doi: 10.1371/journal.pone.0191782 [published Online First: 2018/02/07]
41. Nguyen TQ, Bandeen-Roche K, Bass JK, et al. A tool for sexual minority mental health research: The Patient Health Questionnaire (PHQ-9) as a depressive symptom severity measure for sexual minority women in Viet Nam. *J Gay Lesbian Ment Health* 2016;20(2):173-91. doi: 10.1080/19359705.2015.1080204 [published Online First: 2016/09/20]
42. Mokona H, Yohannes K, Ayano G. Youth unemployment and mental health: prevalence and associated factors of depression among unemployed young adults in Gedeo zone, Southern Ethiopia. *Int J Ment Health Syst* 2020;14:61. doi: 10.1186/s13033-020-00395-2 [published Online First: 2020/08/13]
43. Molla GL, Sebhat HM, Hussen ZN, et al. Depression among Ethiopian Adults: Cross-Sectional Study. *Psychiatry J* 2016;2016:1468120. doi: 10.1155/2016/1468120 [published Online First: 2016/06/02]
44. Gupta R, Taneja N, Anand T, et al. Internet Addiction, Sleep Quality and Depressive Symptoms Amongst Medical Students in Delhi, India. *Community Ment Health J* 2020 doi: 10.1007/s10597-020-00697-2 [published Online First: 2020/08/28]
45. Roberts T, Shidhaye R, Patel V, et al. Health care use and treatment-seeking for depression symptoms in rural India: an exploratory cross-sectional analysis. *BMC Health Serv Res* 2020;20(1):287. doi: 10.1186/s12913-020-05162-0 [published Online First: 2020/04/08]
46. Hernandez-Vasquez A, Rojas-Roque C, Vargas-Fernandez R, et al. Dynamics of depressive symptoms and within-country migration among Peruvian women. *Rural Remote Health* 2020;20(2):5692. doi: 10.22605/RRH5692 [published Online First: 2020/04/03]

For peer review only - http://bmjopen.bmj.com/site/about/guidelines.xhtml

47. Hernandez-Vasquez A, Vargas-Fernandez R, Bendezu-Quispe G, et al. Depression in the Peruvian population and its associated factors: analysis of a national health survey. *J Affect Disord* 2020;273:291-97. doi: 10.1016/j.jad.2020.03.100 [published Online First: 2020/05/19]
48. Spitzer RL, Kroenke K, Williams JB, et al. A brief measure for assessing generalized anxiety disorder: the GAD-7. *Arch Intern Med* 2006;166(10):1092-7. doi: 10.1001/archinte.166.10.1092 [published Online First: 2006/05/24]
49. Kroenke K, Strine TW, Spitzer RL, et al. The PHQ-8 as a measure of current depression in the general population. *J Affect Disord* 2009;114(1-3):163-73. doi: 10.1016/j.jad.2008.06.026 [published Online First: 2008/08/30]
50. Cronbach LJ. Coefficient alpha and the internal structure of tests. *Psychometrika* 1951;16(3):297-334. doi: 10.1007/bf02310555
51. Nunnally J, Bernstein I. Psychometric Theory. 3 ed. New York: McGraw-Hill 1994.
52. Briggs SR, Cheek JM. The role of factor analysis in the development and evaluation of personality scales. *Journal of Personality* 1986;54(1):106-48. doi: 10.1111/j.1467-6494.1986.tb00391.x
53. Clark LA, Watson D. Constructing validity: Basic issues in objective scale development. *Psychological Assessment* 1995;7(3):309-19. doi: 10.1037/1040-3590.7.3.309
54. Cantril H. The pattern of human concerns: New Brunswick, N. J. : Rutgers University Press, [1965] 1965.
55. Briones K. 'How Many Rooms Are There in Your House?'Constructing the Young Lives Wealth Index. 2017; 43. https://www.younglives.org.uk/sites/www.younglives.org.uk/files/YL-TN43_0.pdf.
56. Marsh HW. Self Description Questionnaire (SDQ) II: A theoretical and empirical basis for the measurement of multiple dimensions of adolescent self-concept. San Antonio, TX Psychological Corporation 1990.
57. Marsh HW. The Self Description Questionnaire (SDQ): A theoretical and empirical basis for the measurement of multiple dimensions of preadolescent self-concept: A test manual and research monograph. San Antonio, TX Psychological Corporation 1988.
58. Amsalem D, Dixon LB, Neria Y. The Coronavirus Disease 2019 (COVID-19) Outbreak and Mental Health: Current Risks and Recommended Actions. *JAMA Psychiatry* 2021;78(1):9-10. doi: 10.1001/jamapsychiatry.2020.1730 [published Online First: 2020/06/25]
59. Ritchie H, Ortiz-Ospina E, Beltekian D, et al. Coronavirus Pandemic Data Explorer. In: OurWorldInData.org, ed.: Oxford Martin School, University of Oxford 2020.
60. McLean CP, Asnaani A, Litz BT, et al. Gender differences in anxiety disorders: prevalence, course of illness, comorbidity and burden of illness. *J Psychiatr Res* 2011;45(8):1027-35. doi: 10.1016/j.jpsychires.2011.03.006 [published Online First: 2011/03/29]
61. Salk RH, Hyde JS, Abramson LY. Gender differences in depression in representative national samples: Meta-analyses of diagnoses and symptoms. *Psychol Bull* 2017;143(8):783-822. doi: 10.1037/bul0000102 [published Online First: 2017/04/28]
62. Dachew BA, Bifftu BB, Tiruneh BT, et al. Prevalence of mental distress and associated factors among university students in Ethiopia: a meta-analysis. *J Ment Health* 2019:1-8. doi: 10.1080/09638237.2019.1630717 [published Online First: 2019/06/30]
63. Hailemariam S, Tessema F, Asefa M, et al. The prevalence of depression and associated factors in Ethiopia: findings from the National Health Survey. *International Journal of Mental Health Systems* 2012;6(23)
64. Nguyen A. Cultural and social attitudes towards mental illness in Ho Chi Minh City, Vietnam. *Seattle University Research Journal, Spring* 2003;2:27–31.
65. Reta Y, Tesfaye M, Girma E, et al. Public Stigma against People with Mental Illness in Jimma Town, Southwest Ethiopia. *PLoS One* 2016;11(11):e0163103. doi: 10.1371/journal.pone.0163103 [published Online First: 2016/11/29]

For peer review only - http://bmjopen.bmj.com/site/about/guidelines.xhtml

66. Arriola-Vigo JA, Stovall JG, Moon TD, et al. Perceptions of Community Involvement in the Peruvian Mental Health Reform Process Among Clinicians and Policy-Makers: A Qualitative Study. *International Journal of Health Policy and Management* 2019;8(12):711-22. doi: 10.15171/ijhpm.2019.68

67. Gaiha SM, Taylor Salisbury T, Koschorke M, et al. Stigma associated with mental health problems among young people in India: a systematic review of magnitude, manifestations and recommendations. *BMC Psychiatry* 2020;20(1):538. doi: 10.1186/s12888-020-02937-x [published Online First: 2020/11/18]

68. Kane JC, Elafros MA, Murray SM, et al. A scoping review of health-related stigma outcomes for high-burden diseases in low- and middle-income countries. *BMC Med* 2019;17(1):17. doi: 10.1186/s12916-019-1250-8 [published Online First: 2019/02/16]

69. Mascayano F, Armijo JE, Yang LH. Addressing stigma relating to mental illness in low- and middle-income countries. *Front Psychiatry* 2015;6:38. doi: 10.3389/fpsyt.2015.00038 [published Online First: 2015/03/31]

70. Bauer A, Garman E, McDaid D, et al. Integrating youth mental health into cash transfer programmes in response to the COVID-19 crisis in low-income and middle-income countries. *The Lancet Psychiatry* 2021 doi: 10.1016/s2215-0366(20)30382-5

71. Dong E, Du H, Gardner L. An interactive web-based dashboard to track COVID-19 in real time. *The Lancet Infectious Diseases* 2020;20(5):533-34. doi: 10.1016/s1473-3099(20)30120-1

For peer review only

For peer review only - http://bmjopen.bmj.com/site/about/guidelines.xhtml

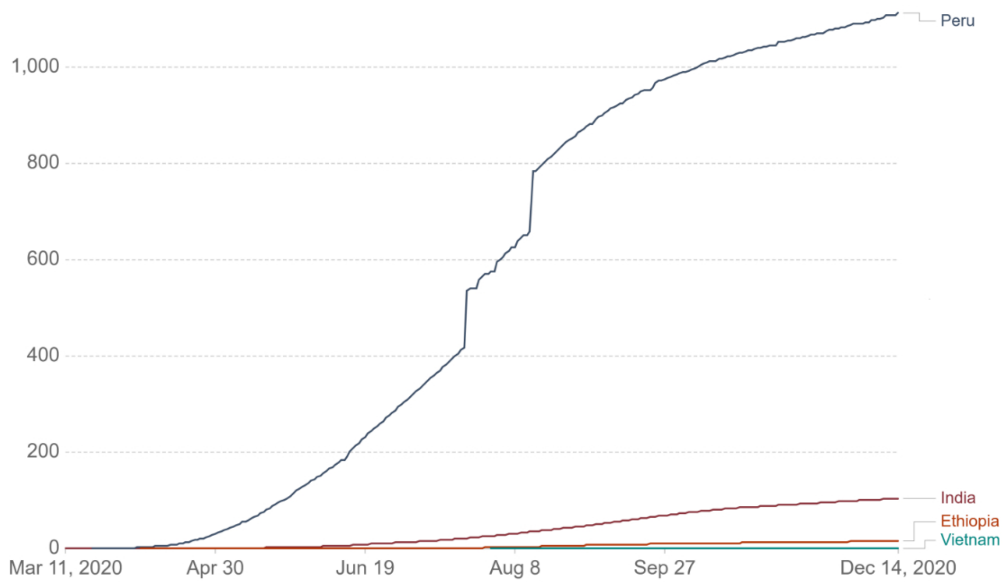

Figure 1. Cumulative confirmed COVID-19 cases per million people in the four Young Lives countries. Source: Johns Hopkins University CSSE COVID-19 Data52, accessed via our world in data. Last updated 15 December 2020. Testing and challenges in the attribution of the cause of death means that the number of confirmed deaths may not be an accurate count of the true number of deaths from COVID-19.

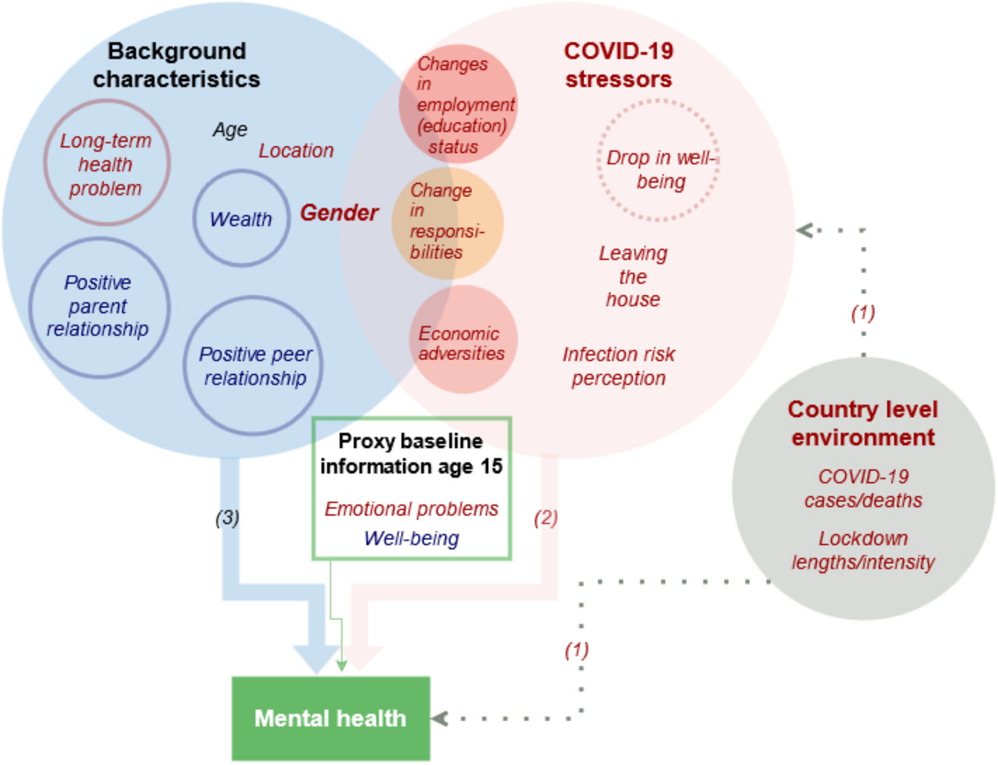

Figure 2. Theoretical framework of the hypothesized impact of COVID-19 stressors, background characteristics and the country level environment on mental health
(1), (2), and (3) are the channels as discussed in the framework. Red font colour indicates hypothesized risk factors, blue font colour hypothesized protective factors. In case of gender, females are the hypothesized vulnerable group. Urban participants are hypothesized to be more vulnerable than rural residents (location). Black font colour indicates a potential effect in either direction. White font colour refers to outcome variables. (Solid) lines indicates that the variable was measured in previous in-person rounds. Filled sub-circles are categorical/composite variables. Boxes with no fill colour indicate robustness checks to the main framework. Dotted arrows indicate that the variable was only considered implicitly for descriptive statistics. Solid arrows indicate use in logistic regressions. Changes in employment status and changes in the educational status (=educational disruption) are used interchangeably for the Younger Cohort. Mental health was measured using the GAD-7 and PHQ-8 and a cut-off of five reflecting at least mild symptoms of anxiety/depression was chosen.

250x191mm (72 x 72 DPI)

Supplementary documents

Supplement to: Impact of the COVID-19 Pandemic on anxiety and depression symptoms of young people in the Global South: evidence from a four-country cohort study

- Survey questionnaire

Listening to Young Lives at Work: COVID-19 phone survey Second survey call

Data Handlers 2
Section 1. Introduction 7
Section 2. COVID-19: Behaviours and Risk Perceptions 10
Section 3. Socio-economic status 14
Section 4. Recent life history and Economic changes 18
4.1 Recent life history 18
4.2 Economic Shocks since the COVID-19 Outbreak 19
Section 5. Food Security, Prices and Health 22
5.1 Food Security 22
5.2 Food Prices 23
5.3 Individual Health 24
Section 6. Current Education 27
Section 7. Time Use 30
Section 8. Employment and Earnings 31
8.1 Labour Force participation 31
8.2 Main activity in the past 7 days 33
8.3 Main activity before COVID-19 38
8.4 Main activity during lockdown period 40
Section 9. Subjective wellbeing and Mental Health 42
9.1 Subjective wellbeing 42
9.2 Mental Health 43
Concluding remarks 46
Locating Information and contact info 48
Comments 49
Enumerator Statement 51
Codeboxes 52

For peer review only

Data Handlers

ETHIOPIA

CHILDID AND DATE

**0.1**	INSERT CHILD ID	**ET __ __ - 1 - __ __ __**	CHILDID
**0.2**	DATE OF INTERVIEW	__ __ / __ __ / 2 0 2 0<br>(day) (month) (year)	DINT
**0.3**	PHONE NUMBER USED FOR THE INTERVIEW		PHNUMB

DATA HANDLERS

Fieldworker that initiates questionnaire	**Name:**<br>✍______________________________	Code: [__ __ __]	FLWRIDPS
		**Date of check:**<br>__ __ / __ __ / 2 0 2 0<br>(day) (month) (year)	DINT
	ENUMERATOR ID number :<br>________________________		ID
	**Start time** (Use 24 hours):	[__ __ : __ __]	
Fieldworker that finishes questionnaire	**Name:**<br>✍______________________________	Code: [__ __ __]	ENDFLDPS
		**Date of check:**<br>__ __ / __ __ / 2 0 2 0<br>(day) (month) (year)	ENDDINT
	ENUMERATOR ID number 2:<br>____________________________		ID2
	**End time** (Use 24 hours):	[__ __ : __ __]	

COMMENTS/OBSERVATIONS:

✍____

For peer review only

INDIA

CHILDID AND DATE

0.1	INSERT CHILD ID	IN _ _ _ _ _ _	CHILDID
0.2	DATE OF INTERVIEW	_ _ / _ _ / 2 0 2 0<br>(day) (month) (year)	DINT
0.3	PHONE NUMBER USED FOR THE INTERVIEW		PHNUMB

DATA HANDLERS

Fieldworker that initiates questionnaire	**Name:**<br>✍ ____________________	Code: [_ _ _]	FLWRIDPS
		**Date of check:**<br>_ _ / _ _ / 2 0 2 0<br>(day) (month) (year)	DINT
	ID: ____________________		ID
	**Start time** (Use 24 hours):	[_ _ : _ _]	
Fieldworker that finishes questionnaire	**Name:**<br>✍ ____________________	Code: [_ _ _]	ENDFLDPS
		**Date of check:**<br>_ _ / _ _ / 2 0 2 0<br>(day) (month) (year)	ENDDINT
	ID: ____________________		ID2
	**End time** (Use 24 hours):	[_ _ : _ _]	
**Supervisor**	**Name:**<br>✍ ____________________	Code: [_ _ _]	SUPRIDPS
		**Date of check:**<br>_ _ / _ _ / 2 0 2 0<br>(day) (month) (year)	SUPCDATED

For peer review only

COMMENTS/OBSERVATIONS:

✍ ____

PERU

CHILDID AND DATE

**0.1**	INSERT CHILD ID	**PE __ __ - 1 - __ __ __**	CHILDID
**0.2**	DATE OF INTERVIEW	__ __ / __ __ / 2 0 2 0<br>(day) (month) (year)	DINT
**0.3**	PHONE NUMBER USED FOR THE INTERVIEW		PHNUMB

DATA HANDLERS

Fieldworker that initiates questionnaire	**Name:**<br>✍ ________________________	Code: [__ __ __]	FLWRIDR6
		**Date of check:**<br>__ __ / __ __ / 2 0 2 0<br>(day) (month) (year)	DINT
	ID: ________________________		ID
	**Start time** (Use 24 hours):	[__ __ : __ __]	
Fieldworker that finishes questionnaire	**Name:**<br>✍ ________________________	Code: [__ __ __]	ENDFLDPS
		**Date of check:**<br>__ __ / __ __ / 2 0 2 0<br>(day) (month) (year)	ENDDINT
	ID2: ________________________		ID2
	**End time** (Use 24 hours):	[__ __ : __ __]	

For peer review only

Supervisor	Name: ✍_______________	Code: [__ __ __]	SUPRIDPS
		Date of check: __ __ / __ __ / 2 0 2 0 (day) (month) (year)	SUPCDATED

COMMENTS/OBSERVATIONS:

✍_______________

VIETNAM

CHILDID AND DATE

**0.1**	INSERT CHILD ID	**VN**__ __ __ __ __ __	CHILDID
**0.2**	DATE OF INTERVIEW	__ __ / __ __ / 2 0 2 0 (day) (month) (year)	DINT
**0.3**	PHONE NUMBER USED FOR THE INTERVIEW		PHNUMB

DATA HANDLERS

Fieldworker that initiates questionnaire	**Name:** ✍_______________	Code: [__ __ __]	FLWRIDPS
		**Date of check:** __ __ / __ __ / 2 0 2 0 (day) (month) (year)	DINT
	ID: _______________		ID
	**Start time** (Use 24 hours):	[__ __ : __ __]	
Fieldworker that finishes questionnaire	**Name:** ✍_______________	Code: [__ __ __]	ENDFLDPS

For peer review only

		**Date of check:** __ __ / __ __ / 2 0 2 0 (day) (month) (year)	ENDDINT
	ID: ______________________________		ID2
	**End time** (Use 24 hours):	[__ __ : __ __]	
**Supervisor**	**Name:** ✍____	Code: [__ __ __]	SUPRIDPS
		**Date of check:** __ __ / __ __ / 2 0 2 0 (day) (month) (year)	SUPCDATED

COMMENTS/OBSERVATIONS:

✍____

For peer review only

Section 1. Introduction

**ENUMERATOR**: Start the dialogue with YL Child as follows:<br><br>**SAY**: Hello, (are you) / is this [YL Child´s name] ? Hi, I am [first and last name of ENUMERATOR], of the Young Lives Study. The last time we talked to you over the phone was in [DATE OF CALL 1], how are you?<br><br>On that occasion we told you that we were going to contact you again to invite you to participate in a phone survey with questions related to the Covid-19 /Coronavirus, the quarantine, and its effects on your health, wellbeing, education, economy and work and your household. The information we are collecting from you and the other Young Lives Children in our country will help the authorities of the government and our health system to know how to better prevent and deal with these types of emergencies, for the benefit of the entire population.<br><br>The entire survey takes approximately <u>45 minutes</u>. As always, the survey will be done with your consent and on the day and time that is most convenient for you. You know that everything you tell me is <u>confidential</u>.<br><br>Finally, as I told you the last time we talked, there is a <u>reward</u> in gratitude to the time that you have given us to answer all the questions.<br><br>[ET and VN] As for the first call, each participant will be given [ET: will be given Birr 100 or an equivalent Mobile card; VN: will be given VND 50,000 or a mobile card] which is the approximate equivalent to the value (cost) of the present we used to give to you when we visited you in person.<br>[PE and IN] This time, as it is not possible to visit the participants in their homes, we will not be able to personally carry and deliver any presents, so, only for this time all participants will be given [PE: S /. 50 soles, which is the approximate equivalent to the value (cost) of the present], [IN: Rs. 600, which is the approximate equivalent to the value (cost) of the present] as we anticipated to you in the previous call.		
**Q.1**	**SAY**: [YL Child´s name], Do you agree? Will you give me your consent?<br>01=Yes -> Go to next section (Section 2. COVID-19)<br>00=No	O No O Yes
**Q.2**	**SAY**: Why don't you want to participate?<br><br>01= doesn´t have time/can/t do it, because he/she has to go to study… to work, etc." -> Go to Q.3<br><br>02=he/she has doubts and/or don´t trust regarding the information they will give -> Go to Q.4<br><br>03: If the justification given by the person for Not Participating, (in this first call), is linked to a very delicate situation, a Health, Economic or other type of emergency (specifying whether it is personal or related to the family) -> Go to Farewell F3	[__ __]
**Q.3**	**ENUMERATOR:** Explain that you can call again the day and time that is more convenient for he/she…and also, mention again that it will be a conversation of no more than 45 minutes…<br>**SAY**: Would you like to participate? Do you give us your consent to go ahead?<br><br>01=Yes -> **ENUMERATOR**: Schedule Time for the call -> Go to Farewell F2<br>00=No -> **ENUMERATOR**: take note of the reason -> Go to Farewell F1	O No O Yes

For peer review only

	**Q.4**	**ENUMERATOR**: Explain again the Purpose of the conversation ..., the confidentiality of what they tell you and the importance that it has, (for the future of the Health system in our country), that they share their experience along with the experience of other young people of the Young Lives study during the "Coronavirus" the Covid-19 pandemic<br>**SAY**: What do you think? Would you like to participate?<br><br>01=Yes-> Go to next section (Section 2. COVID-19)<br>00=No -> Go to Farewell F1	O No O Yes
**F1**		**SAY**: YL Child´s name don´t worry, your decision is absolutely free and voluntary, we understand and respect it. It is necessary that you know that choosing not to participate in this survey doesn´t affect at all your link with Young Lives because you are still an important part of the study until you decide. That is why is important and necessary that you know that [between November and December, DATE OF PHONE SURVEY THIRD CALL], I will call you again to check how you and your family are doing. This is going to be a very short call, no more than 15 minutes. As always, the survey will be done with your consent and on the day and time that is most convenient for you according to your activities. I will give you a much broader and more detailed explanation so that you can freely decide whether or not you want to participate in the aforementioned survey.At the moment, I can tell you that it has been very good for me to meet and talk with you.Thank you very much for your attention and remember that in [between November and December, DATE OF PHONE SURVEY THIRD CALL], I am calling you again.	
**F2**	**F2a**	Is there a day of the week that usually works better for you?<br>**ENUMERATOR**: Mark all that apply	
		01 Monday	☐
		02 Tuesday	☐
		03 Wednesday	☐
		04 Thursday	☐
		05 Friday	☐
		06 Saturday	☐
		07 Sunday	☐
	**F2b**	When is it easier to reach you?<br>**ENUMERATOR**: Read options and mark all that apply	
		01=Mornings	☐
		02=Afternoons	☐
		03= Evening (after dinner)	☐
		Other comments: ________________	[____]

	F2c	**SAY**: You will remember that whenever we have visited you, at the end of the interview, in gratitude to the time that you have given us to answer all the questions, we have given you a [**PE**: a small present, e.g. backpack, watch, mini radio, etc.); **ET**: a small amount of money or transferred mobile card; **IN**: money purse, vanity bag, etc; **VN**: a small amount of money], right?<br><br>[ET and VN] As for this call and the previous one, each participant will be given [ET: will be given Birr 100 or an equivalent Mobile card; VN: will be given VND 50,000 or a mobile card] which is the approximate equivalent to the value (cost) of the present we used to give to you when we visisted you in person and in gratitude to the time that you have given us.<br><br>[PE and IN] As it is not possible to visit the participants in their homes, we will not be able to personally carry and deliver any presents, so, only for this time, all participants will be given [PE: S /. 50 soles, which is the approximate equivalent to the value (cost) of the present], [IN: Rs. 600, which is the approximate equivalent to the value (cost) of the present] , as we anticipated to you in the previous call.<br><br>Ok, thanks a lot for now. I will call you again at the agreed time.	[__________]
F3		**ENUMERATOR**: collect and write down all the information you can, important details that exactly describe the problem/situation (… ask how they think it could be solved, if they have done any paperwork or management…, or if they are doing something to solve it…, what is their greatest difficulty, etc.)**SAY**:I will present your case, will consult with the supervisors and with the people in charge of the Young Lives study, to find out how or who could help them directly. * According to the case and according to the time, set the day/date on which you would be giving the answer.**ENUMERATOR**: Follow up on the case, and it is probable, (depending on the circumstances), that only when everything is "apparently solved" or in "the process of being solve", in a conversation with he/she you will be able to mention the compensation of [PE: S/. 50 in total; ET: 100 BIRR per survey call; IN: 600 INR in total; VN: 50,000 VND per survey call] as a way to show gratitude for their time, if they decide to participate in the CALL 2 survey and to inform them about the future survey that will take place [between November and December, DATE OF PHONE SURVEY THIRD CALL].If respondent sounds desperate/ suicidal please refer to the manual for further instructions on how to proceed.	[__________]

For peer review only

Section 2. COVID-19: Behaviours and Risk Perceptions

Behaviours and preventive measures adopted

SAY: We would like to ask you few questions about your personal behaviors since the outbreak of coronavirus/COVID-19.

**Q.1**	In the past 7 days, on how many days did you stay at home all day, without going out at all?<br><br>-79=NK<br><br>[**CAPI:** if 0-6 or -79 ask Q.2, if Q.1=7 Go to Q.3]<br><br>[**ENUMERATOR**: if the respondent say "Don't know" ask him/her for his/her best estimate	[__]
**Q.2**	Why did you leave the house during the past 7 days?<br><br>**ENUMERATOR**: please **READ** the alternatives and enter the corresponding responses<br><br>00=No<br>01=Yes<br>88=NA	
01	To buy food	O No O Yes O N/A
02	To buy medicine; visit doctor	O No O Yes O N/A
03	To to go the bank	O No O Yes O N/A
04	To exercise, walk the dog	O No O Yes O N/A
05	To take care of a household member	O No O Yes O N/A
06	To work or work related reasons, with permission	O No O Yes O N/A
07	To work or work related reasons, without permission	O No O Yes O N/A
08	To go to church/mosque/temple/funerals/weddings	O No O Yes O N/A
09	Recreation, meeting friends and family	O No O Yes O N/A
10	To allow my children to go out	O No O Yes O N/A
11	Other, specify [________________________]	O No O Yes O N/A
**Q.3**	How often have you done any of these things to prevent the infection, in the past 7 days?<br><br>00=Never<br>01=Sometime<br>02=Always<br>79=RTA<br><br>**ENUMERATOR**: Please **read** each statement. Note that the YL Child should report about his/her own behaviors rather than what other family members did	
01	Wash your hands with soap more often than you used to	[__ __]
02	Avoid handshakes/ physical greetings	[__ __]

For peer review only

03	Avoid groups meeting such as family gatherings, parties; going to church/temples, funerals/marriages etc.	[__ __]
04	Wear a face mask when outside	[__ __]
05	Keep a distance of at least 1-2 meters from other people	[__ __]
06	Wear gloves when outside	[__ __]
07	Wear a facial shield/protector when outside	[__ __]
**Q.4**	Have you done anything else to prevent catching the infection or spreading it to others? For example changing your behaviour, eating certain food or drink or avoiding certain things?<br><br>**ENUMERATOR**: Please do not read, just mark with "x" all the ones mentioned. Please note that the YL child should report about his/her own behaviors rather than what other family members did	
01	Cancel travel plans	☐
02	Stock up on more food than normal	☐
03	Reduce the number of times you go to the market/grocery store	☐
04	Work from home	☐
05	Shower immediately upon entering the house	☐
06	Take off all clothes that were worn outside	☐
07	Drinking lemon	☐
08	Adding hot pepper to food	☐
09	Adding garlic/ginger/turmeric to food or eating garlic	☐
10	Hot bath when you come in from being out	☐
11	Going outside into the sun	☐
12	Any other herbal medicine/local prevention/homeopathic medicine	☐
13	Avoiding congested transport systems	☐
14	Using hand sanitizers more often	☐
15	Other, specify	☐

For peer review only

Risk perception and attitude to risk

**Q.5**	Do you think your chance of getting infected with Corona is low, medium, or high, or do you have no risk at all?<br><br>00=No risk<br>01=Low<br>02=Medium **-> Skip to Q.7**<br>03=High **-> Skip to Q.7**<br>77=NK **-> Skip to Q.7**<br><br>**ENUMERATOR:** Please, if the YL Child reported to have (or she/he belives to have) already got it, ask her/him to think about the chance to get it again.	[__ __]
**Q.6**	Why do you think you are at low/not at risk?<br><br>**ENUMERATOR**: Do **not** read the alternatives. Mark all that apply	
01	I'm young	☐
02	God protect me	☐
03	The hot/wet weather	☐
04	I haven't travelled	☐
05	COVID is a lie to try to get money	☐
06	I don't know anyone with COVID-19	☐
07	I'm not white or chinese	☐
08	COVID is not in my country region / continent/ community	☐
09	I already got it	☐
10	Other (specify)	[_______________]

SAY: Now, I would like to understand what is your general attitude to risk

**Q.7**	Suppose I offered you a lottery ticket that wins 150 Birr/620 INR/155 Soles/600,000 VND with 50-50 chance. How much would you be willing to pay for such a ticket?<br><br>**ENUMERATOR**: if the [YL Child] seems not to understand please SAY "50-50 chance means that you have an equal chance to win the lottery or to not get anything and waste what you pay for the ticket". If the [YL Child] say s/he would not be willing to pay for a lottery ticket or is not interested in playing the lottery, please code as 0.<br>77=NK	[_____]

SAY: I am going to read you now a list of five statements that people like you have done or experienced during the lockdown period.

(CAPI: This questions apply ONLY to PE and IN): Sample is split into Group A and Group B. Group A gets Q8a only and Group B gets Q8b only.

Group A answer Q.8a and then Go to the next section; Group B answer Q.8b and then Go to the next section).

**Q.8a**	**SAY**: Now I'm going to read you 5 statements. Please listen to me very carefully and tell me how many of the following things you have done, or happened to you. You don't have to tell me about each one, only how many of the 5 statements are true for you.<br>An easy way to keep score is with the fingers of your hand. I mean, close your fist, and as I read, raise a finger every time you agree. Then at the end we can check how many fingers you have raised<br><br>**ENUMERATOR**: Make sure the participant has heard the list and repeat it if necessary.<br>**ENUMERATOR**: When you finish mentioning the statements, ask the young person many fingers s/he has raised. Record the number that the respondent tells you. [0-5]<br>1. During the lockdown I was able to spend more free time than previously doing exercise<br>2. I felt much closer to my family during the lockdown than I did before<br>3. During the lockdown I started drinking more alcohol than usual<br>4. During the lockdown there were more arguments in our household than there were before<br>5. I was physically hurt more often by someone in my household during the lockdown	[__ __]

**Q.8b**	**SAY**: Now I'm going to read you 4 statements. Please listen to me very carefully and tell me how many of the following things you have done, or happened to you. You don't have to tell me about each one, only how many of the 4 statements are true for you.<br>An easy way to keep score is with the fingers of your hand. I mean, close your fist, and as I read, raise a finger every time you agree. Then at the end we can check how many fingers you have raised<br><br>**ENUMERATOR**: Make sure the participant has heard the list and repeat it if necessary.<br>**ENUMERATOR**: When you finish mentioning the statements, ask the young person many fingers s/he has raised. Record the number that the respondent tells you. [0-4]<br>-<br>1. During the lockdown I was able to spend more free time than previously doing exercise<br>2. I felt much closer to my family during the lockdown than I did before<br>3. During the lockdown I started drinking more alcohol than usual<br>4. During the lockdown there were more arguments in our household than there were before	[__ __]

Section 3. Socio-economic status

SAY: Now I am going to ask some questions about the place where you live.

**Q.1**	How many people, including yourself, are currently living with you?<br><br>**ENUMERATOR**: Enter the number of people currently living in the same household as the YL Individual, YL individual included. Please remember that a household is defined as a group of individuals that meets three conditions: 1. live under the same 'roof' or within the same compound/homestead/stand; 2. share food from a common source at least once a day; 3. they contribute to or share in, a common resource pool, aside from food and shelter.	[__ __]
**Q.2**	**SAY**: How many rooms are there in the house (excluding kitchen, bathroom, garage) ?<br><br>**ENUMERATOR**: Do not include kitchen, bathrooms, passage, garage, warehouses or rooms divided with non-permanent items such as a curtain.<br><br>77=NK	[__ __]
**Q.3**	Do you have a separate kitchen/cooking area?<br><br>00=No<br>01=Yes<br>77=NK	○ No ○ Yes ○ NK
**Q.4**	Do you have access to electricity?<br><br>00=No<br>01=Yes<br>77=NK	○ No ○ Yes ○ NK
**Q.5**	Is your dwelling separated from the outside by walls on all sides?	○ No ○ Yes ○ NK
**Q.6**	Is your dwelling completely covered by a roof?	○ No ○ Yes ○ NK
**Q.7**	What is the main source of drinking water for members of your household?<br><br>Enter codes from **CODEBOX #1**	[__ __]<br>Specify__________

**Q.8**	What kind of toilet facility does your household use?<br><br>Enter codes from **CODEBOX #2**	[__ __]<br>Specify__________

**Q.9**	Do you always have access to the following when you need it:<br><br>**ENUMERATOR**: Read answer options aloud before reading each entry 01-03<br><br>01 = Yes<br>02 = No<br>77=NK	
01	Clean water for washing hands	O No O Yes O NK
02	Soap for washing your hands	O No O Yes O NK
03	Hand sanitizer	O No O Yes O NK

SAY: I am now going to ask you whether the household owns specific items.
FIELDWORKER: Ask about each item.

ALL COUNTRIES

	**Household Items**	**Q.10**<br>Does anyone in the household own this item?<br><br>Tick in the box if anyone owns it.<br><br>If NO tick ► Skip to next row
1	Working television	☐
2	Working radio	☐
3	Working smartphone	☐
4	Access to internet at home (through computer)	☐

Ownership of Saving accounts (FOR OC)

SAY: If someone wants to keep money at a bank, he/she can open a savings account. The money in the savings account is yours. Sometimes, employers open savings account for their employees to deposit their payments.

**Q.11oc**<br><br>**(CAPI: ET, PE, IN)**	Do you or your partner currently own a bank account?<br><br>00 = No **-> Skip to Section 4. EconChanges**<br>01= Yes, I have a bank account<br>02= Yes, my partner has a bank account<br>03=Yes, we both have bank accounts<br>77=NK<br>79=RTA	[__ __]

**Q.11oc (CAPI: VN)**	Do you or someone else in your household own a bank account?<br><br>00= No **-> Skip to Section 4. EconChanges**<br>01= Yes, I have a bank account<br>02= Yes, someone else in my household has a bank account<br>03= Yes, myself and someone else in my household has a bank account<br>77=NK<br>79=RTA	[__ __]
**Q.12oc**	[CAPI: This question is for PE only]<br>In which institution do you or your partner keep your bank account(s)?<br>Allow for up to 3 answers.<br><br>01 = Banco de la Nación<br>02 = Commercial Bank<br>03 = Financial Firm<br>04 = Municipal savings banks<br>05 = Rural savings banks<br>06 = Edpymes<br>07 = Other (specify) ____________________<br>77=NK<br>88=NA<br>79=RTA	1. [__ __]<br>2. [__ __]<br>3. [__ __]

Ownership of Saving accounts (for YC)

SAY: If someone wants to keep money at a bank, he/she can open a savings account. The money in the savings account is yours. Sometimes, employers open savings account for their employees to deposit their payments.

**Q.11yc**	Do you or someone else in your household own a bank account?<br><br>00= No **-> Skip to Section 4. EconChanges**<br>01= Yes, I have a bank account<br>02= Yes, someone else in my household has a bank account<br>03= Yes, myself and someone else in my household has a bank account<br>77=NK<br>79=RTA	[__ __]

**Q.12yc**	[CAPI: This question is for PE only]<br>In which institution do you and/or someone else in your household keep your bank account(s)?<br>Allow for up to 3 answers.<br><br>01 = Banco de la Nación<br>02 = Commercial Bank<br>03 = Financial Firm<br>04 = Municipal savings banks<br>05 = Rural savings banks<br>06 = Edpymes<br>07 = Other (specify) ____________________<br>77=NK<br>88=NA<br>79=RTA	1. [__ __]<br>2. [__ __]<br>3. [__ __]

For peer review only

Section 4. Recent life history and Economic changes

4.1 Recent life history

SAY: I am now going to ask you to think about your current circumstances and how and why they might have changed over recent periods.

**Q.1**	How would you describe the household you were living in?<br><br>**FIELDWORKER:** Please read the alternatives<br><br>01=Very rich<br>02=Rich<br>03=Comfortable – manage to get by<br>04=Struggle – never have quite enough<br>05=Poor<br>06=Destitute	
01	Currently	[__ __]
02	Before [date of Outbreak], when the COVID crisis started	[__ __]

**Q.2**	Have the total household expenses been affected by the current COVID-19 pandemic?<br><br>00=No<br>01= Yes, they have gone up<br>02=Yes, they have gone down<br>77=NK	[__ __]
**Q.3**	During the past month, did the **total (monthly) income of your household** change compared to how it was before [date of outbreak], i.e. when the pandemic started?<br><br>00=No<br>01= Yes, it has gone up<br>02=Yes, it has gone down<br>77=NK	[__ __]

4.2 Economic Shocks since the COVID-19 Outbreak

SAY: Now I am going to ask you about the most important events and changes that have happened (that negatively affected the household economy) since [DATE OUTBREAK]

CAPI: Add a flag for those households that in Call 1 reported:
1. At least one household member (including the YL respondent) lost her/his job, was suspended without payment/had their salary cut or lost all or most of their income from their own business

EVENTS		Q.4<br>Has any of the following events affected your household since [DATE OUTBREAK]?00=No01=Yes77=NK**ENUMERATOR**: **read** the events one by one. Make sure to remind to the respondent that this question refer to shocks happened since the outbreak.
**1**	Job loss	O No O Yes O NK
**2**	Some household members not received payment/full payment since [DATE OUTBREAK]	O No O Yes O NK
**3**	Nonfarm business closure	O No O Yes O NK
**4**	Less work, less or no clients in a nonfarm family business	O No O Yes O NK
**5**	Disruption of farming, livestock, fishing activities, and supply chain disruption	O No O Yes O NK
**6**	Increase in price of farming/business inputs	O No O Yes O NK
**7**	Fall in the price of farming/business output	O No O Yes O NK
**8**	Increase in price of major food items consumed	O No O Yes O NK
**9**	Illness, injury, or death of income earning member of household	O No O Yes O NK
**10**	Theft/looting of cash and other property	O No O Yes O NK
**11**	New health expenses	O No O Yes O NK
**12**	New expenses: for example expenses required by the employer to continue working: buying a laptop, hire internet, etc	O No O Yes O NK
**13**	Locusts (**CAPI**: ET only)	O No O Yes O NK
**14**	Burning down of property by riot/violence (Oromia) (CAPI: ET only)	O No O Yes O NK
**15**	Violence/riot related injuries/loss of life (Oromia) (CAPI: ET only)	O No O Yes O NK
16	Violence related arrest of family members (CAPI: ET ONLY)	O No O Yes O NK
17	Flooding (CAPI: ET only)	O No O Yes O NK

CAPI: Enable Q.5 if YL child answered Yes to at least one of the events mentioned in Q.4

REACTIONS		**Q.5** How did your household cope with this/these events that negatively affected your household since [DATE OUTBREAK]? **ENUMERATOR**: Do **not read** the alternatives. Mark with an "x" all options that apply.
**1**	Sale of assets	☐
**2**	Engaged in additional income generating activity	☐
**3**	Received assistance from friends and family	☐
**4**	Took a loan from a financial institution	☐
**5**	Credited purchases	☐
**6**	Delayed payments	☐
**7**	Sold harvest in advance	☐
**8**	Reduced food consumption	☐
**9**	Relied on savings	☐
**10**	Received assistance from NGO/Faith based organization(CAPI: ET only)	☐
**11**	Took an advance payment from employer	☐
**12**	Received assistance from Government	☐
**13**	Was covered by insurance policy	☐
**14**	Did nothing	☐
**15**	Took any other type of informal loans	☐
**16**	Migrate	☐
**17**	Switched to another job	☐
**18**	Other, Specify	☐

CAPI: Q.5a and Q.5b applies to PE only

**Q.5a**	Since (Outbreak date), did anyone in your household withdraw money from their CTS ("Compensacion por tiempo de servicios") account?<br><br>01=Yes<br>00=No<br>77=NK	○ No ○ Yes ○ NK
**Q.5b**	Since (Outbreak date), did anyone in your household withdraw money from their pension fund ("AFP")?<br><br>01=Yes<br>00=No<br>77=NK	○ No ○ Yes ○ NK

SAY: I am going to read you now a list of five statements that people like you have done or experienced during the lockdown period.
(CAPI: This questions apply ONLY to PE and IN): Sample is split into Group A and Group B. Group A gets Q6a only and Group B gets Q6b only.

Group A answer Q.6a and then Go to the next section; Group B answer Q.6b and then Go to the next section).

**Q.6a**	**SAY**: Now I'm going to read you 4 statements. Please listen to me very carefully and tell me how many of the following things you have done it, or it happened to you. You don't have to tell me about each one, only how many of the 4 statements are true for you.<br>An easy way to keep score is with the fingers of your hand. I mean, close your fist, and as I read, raise a finger every time you agree. Then at the end we can check how many fingers you have raised<br><br>**ENUMERATOR**: Make sure the participant has heard the list and repeat it if necessary.<br>**ENUMERATOR**: When you finish mentioning the statements, ask the young person many fingers s/he has raised. Record the number that the respondent tells you. [0-4]<br>1. I had more caring responsibilities when the schools were closed during the lockdown<br>2. I spent much more time working to earn money during the lockdown than I normally would have<br>3. I talked on the phone to my friends much more than usual during the lockdown<br>4. During the lockdown I ran out of the money I needed for basic things more often than before	[__ __]

**Q.6b**	**SAY**: Now I'm going to read you 5 statements. Please listen to me very carefully and tell me how many of the following things you have done it, or it happened to you. You don't have to tell me about each one, only how many of the 5 statements are true for you.<br>An easy way to keep score is with the fingers of your hand. I mean, close your fist, and as I read, raise a finger every time you agree. Then at the end we can check how many fingers you have raised<br><br>**ENUMERATOR**: Make sure the participant has heard the list and repeat it if necessary.<br>**ENUMERATOR**: When you finish mentioning the statements, ask the young person many fingers s/he has raised. Record the number that the respondent tells you. [0-5]<br>1. I had more caring responsibilities when the schools were closed during the lockdown<br>2. I spent much more time working to earn money during the lockdown than I normally would have<br>3. I talked on the phone to my friends much more than usual during the lockdown<br>4. During the lockdown I ran out of the money I needed for basic things more often than before<br>5. I was physically hurt more often by someone in my household during the lockdown	[__ __]

Section 5. Food Security, Prices and Health

5.1 Food Security

**SAY**: Now I would like to ask you some questions about food. Since [Outbreak date], was there a time when:<br>00=No<br>01=Yes<br>77=NK		
**Q.1**	You or others in your household worried about not having enough food to eat because of a lack of money or other resources?	○ No ○ Yes ○ NK
**Q.2**	Still thinking about the time since [Outbreak date], was there a time when you or others in your household were unable to eat healthy and nutritious food because of a lack of money or other resources?	○ No ○ Yes ○ NK
**Q.3**	Was there a time since [Outbreak date] when you or others in your household ate only a few kinds of foods because of a lack of money or other resources?	○ No ○ Yes ○ NK
**Q.4**	Was there a time since [Outbreak date] when you or others in your household had to skip a meal because there was not enough money or other resources to get food?	○ No ○ Yes ○ NK
**Q.5**	Still thinking about the time since [Outbreak date], was there a time when you or others in your household ate less than you thought you should because of a lack of money or other resources?	○ No ○ Yes ○ NK
**Q.6**	Was there a time since [Outbreak date] when your household ran out of food because of a lack of money or other resources?	○ No ○ Yes ○ NK
**Q.7**	Since [Outbreak date], was there a time when you or others in your household were hungry but did not eat because there was not enough money or other resources for food?	○ No ○ Yes ○ NK
**Q.8**	Was there a time since [Outbreak date] when you or others in your household went without eating for a whole day because of a lack of money or other resources?	○ No ○ Yes ○ NK

5.2 Food Prices

Q.9	For each of these particular food items, can you please tell me if the price per unit has increased, decreased, or stayed in the same in comparison to before [DATE OF OUTBREAK]?<br><br>01=Increased<br>02=Decreased<br>03=Stayed the same<br>04=Item unavailable<br>-77=NK<br><br>**CAPI**: The list of food items varies across countries.	
	ETHIOPIA	
01	Injera/bread (teff, maize, wheat, and/or barley)	[__ __]
02	Inset	[__ __]
03	Oil/fats/butter	[__ __]
04	Milk/cheese/yogurt	[__ __]
05	Vegetables (including onion, tomato, potato, cabbage, garlic)	[__ __]
06	Pulses (peas and beans)	[__ __]
07	Sugar	[__ __]
	INDIA	
01	Rice and other cereals	[__ __]
02	Vegetables including onion	[__ __]
03	Oil, fat, ghee, butter	[__ __]
04	Milk and milk products	[__ __]
05	Meat and meat products including fish	[__ __]
06	Pulses	[__ __]
07	Any sugar, honey, sweets, sugary sweet drinks	[__ __]
08	Eggs	[__ __]
	PERU	
01	Bread	[__ __]
02	Rice	[__ __]
03	Oil	[__ __]
04	Milk	[__ __]
05	Chicken	[__ __]
06	Pulses	[__ __]
07	Sugar	[__ __]
08	Fruits	[__ __]
	VIETNAM	
01	Rice	[__ __]
02	Chicken	[__ __]
03	Fish	[__ __]
04	Milk	[__ __]
05	Vegetables	[__ __]
06	Fruits	[__ __]
07	Sugar	[__ __]

5.3 Individual Health

SAY: Now I would like to ask some questions about your health. But before doing it I would like to ask you about your family:

[**CAPI**: create a flag for those households were
1.One or more hh members were reported as infected by COVID in call 1
2. One of the hh members died from covid as reported in call 1]

**Q.1**	Since [FIST CALL DATE] when we last spoke to you, did anyone in your household (excluding you) get sick with COVID (either confirmed by a test, or you highly suspect it because of the symptoms display).<br><br>**SAY**: Typical symptom are high temperature, continuous cough, breathing problems, chest pain, loss of smell & taste<br><br>**ENUMERATOR**: Encourage the YL Child answer this question. If theYL Child answers "I don't know", emphasize that what he seeks to capture is his/her perceptions. Repeat symptoms if necessary. If, despite the above, YL Child repeat "I don't know" please mark it and add any relevant comment.<br><br>00= No<br>01= Yes, one person<br>02= Yes, more than one person<br>77=NK _Any comment? Specify {________________}	[__ __]

SAY: Now I would like to ask some questions about your health.

**Q.2**	Since [FIRST CALL DATE], have you been tested for COVID? Which type of test did you take?<br>[**CAPI**: this question should be answer to everyone regardless of what they answer in Q.1]<br><br>00 = No test **->Skip to Q.5**<br>01 = Quick test (blood drop)<br>02 = Nose and throat sample<br>03 = Other, specify {______________________}<br>04=Both types of test<br>77 = NK **->Skip to Q.5**	[__ __]
**Q.3**	How many times have you been tested, since [FIRST CALL DATE]?	[__ __]
**Q.4**	Did any of those tests turn to be positive?<br>00=No<br>01=Yes ->Skip to Q.6<br>77=NK	○ No ○ Yes ○ NK

**Q.5**	Have you been infected or you think you might have been infected since [FIRST CALL DATE] when we talked last time?<br><br>**SAY**: Typical symptom are: high temperature, continuous cough, breathing problems, chest pain, loss of smell & taste<br><br>00=No -> Go to the next section<br>01=Yes<br>77=NK _Any comment? Specify {________________}<br><br>**ENUMERATOR**: Encourage the YL Child to answer this question Yes or No. If the YL Child answers "I don't know", emphasize that what he seeks to capture is his/her perceptions. Repeat symptoms if necessary. If, despite the above, YL Child repeat "I don't know" please mark it and add any relevant comment.<br><br>**CAPI**: add a validation check. If Q.2=01,02,03,04 AND Q.4=01, then it should be Q.5=01; If Q.2=00, 77 then Q.5 can assume any value	O No O Yes O NK
**Q.6**	Did you receive treatment, since [FIST CALL DATE] ?<br><br>01 = Yes<br>00 = No **-> Skip to Q.8**<br>77=NK **-> Skip to Q.8**	O No O Yes O NK
**Q.7**	What type of treatment did you receive?<br><br>[**CAPI**] : Once answered go to Q.9]<br><br>**ENUMERATOR:** do not read the options. If the YL Child report more than one treatment please ask about the main one.<br><br>01 = Instructions by phone/WhatsApp<br>02 = Was under observation at home<br>03 = Hospitalized in private clinic<br>04 = Hospitalized in public hospital<br>05 = Hospitalized in health post (not relevant for IN)<br>06 = Was in Intensive Care Unit<br>07= Self-prescribed traditional medicine<br>08=Traditional medicine prescribed by local traditional doctors or healer<br>09=Self-prescribed modern medicine<br>10= Prescribed modern medicine by doctor<br>11= Other, specify	[__ __]

**Q.8**	Why didn't you receive treatment?<br><br>**ENUMERATOR:** do not read the options. If the YL Child report more than a reason please ask about the main one.<br><br>00= Not tested<br>01 = Rejected by the health center<br>02 = No health center where we live<br>03 = Told to stay at home<br>04 = Told that treatment was not required<br>06= I rejected seeking treatment/not interested<br>07= Family advise me not to take treatment<br>08= Fear of isolation from the community<br>09= Fear of being culprit of bringing the virus to the community<br>10= Belief illness can be cured at home.<br>11 = Other, specify	[__ __]
**Q.9**	How are you now?<br><br>01 = Fully recovered<br>02 = Recovered, but still have some problems<br>03 = Still ill	[__ __]

r review only

Section 6. Current Education

SAY: Now I would like to ask you some questions about your **current educational status**.

**Q.1**	PE: Are you currently enrolled in full-time education?<br>VN: Are you currently enrolled in full-time education/Are you planning to be enrolled in full-time education in September?<br>ET: Are you currently enrolled in full-time education/Are you planning to be enrolled in full-time education when the school/educational institutes reopen?<br>IN: Are you planning to be enroleld in full-time education in the next academic year (mid-August/September)?<br><br>ENUMERATOR (ET only): please note that if the interview happens before the beginning of the academic year in September, you should ask whether the [YL Child] plans to be enrolled in full-time education starting in September. If the interview happened later, and the academic year already started, then you should refer to the current enrolment.<br><br>**ENUMERATOR**: this includes online and blended sessions<br><br>00 = No ► Skip to Q.5<br>01 = Yes, attending regularly/ IN: Yes, I'm planning to attend regularly<br>02 = Yes, but attending irregularly/IN: Yes, even if I will be able to attend irregularly<br>03 = Never attended ► Skip to next section<br>04 = Registered for this school year but classes are suspended/ IN: Yes, I'm registered for the next school year but classes are suspended<br>77 = NK ► Skip to next section<br>79 = Refused to answer ► Skip to next section	[__ __]
**Q.2**	PE: What is the education level that you are currently studying (ET, VN:or you will be studying in September; IN: you will be studying in August/September) ?<br><br>**ENUMERATOR**: If the [YL Child] reports primary or secondary education level, ask him/her to specify what grade. Enter code from CODEBOX #3	[__ __]
**Q.3**	What type of school, institute or university (PE: are you currently attending) (ET/VN:or you will be attending in September; IN: will you be attending/ planning to attend in August/September) ?<br><br>**ENUMERATOR**: Enter code from CODEBOX #4<br><br>**CAPI**: Skip pattern varies across countries<br>**ET**: if Q.2 (CODEBOX#3)= 21;22;24; 25;32-40; 43-48; Ask Q.4 and ► Skip to Q.10; if Q.2=00-14;16-17;26-31; 41-42; 88, 77 ►Skip to Q.10<br>**IN YC:** if Q.2 (CODEBOX#3)= 13-15 Ask Q.4 and ► Skip to Q.10; if Q.2= 00-12;28,29,77,79►Skip to Q.10<br>**IN OC**: if Q.2 (CODEBOX#3)= 09-10 Ask Q.4 and ► Skip to Q.10; if Q.2= 00;01-0-6,08,77,79►Skip to Q.10<br>**PE:** if Q.2 (CODEBOX#3)= 13-16, 19, 21, 22 Ask Q.4 and ► Skip to Q.10; if Q.2=00-11; 17, 18, 20, 77, 88►Skip to Q.10<br>**VN:** if Q.2 (CODEBOX#3)= 13-16 Ask Q.4 and ► Skip to Q.10; if Q.2=00-12,17,28,29,50,77,79►Skip to Q.10	[__ __]

**Q.4a**	What major?<br>**ENUMERATOR**: Write the name of the major. Please look for the right code at the end of the call and add it in the line below.	[________________]
**Q.4b**	**ENUMERATOR**: Enter code from CODEBOX #5<br><br>-> ET, IN, VN Go to Q. 10<br>-> PE: Go to Q.4c and then go to Q.10	[__ __]
**Q.4c**	[CAPI : PE only]<br>Do you plan to continue your studies in the next 12 months?<br><br>00= No<br>01= Yes<br>77 = NK<br>88 = NA<br><br>-> Go to Q.10	[__ __]
**Q.5**	What is the (highest) education level that you completed?<br><br>**ENUMERATOR**: If the [YL Child] reports primary or secondary education level, ask him/her to specify what grade. Enter code from CODEBOX #3	[__ __]
**Q.6**	What type of school, institute or university was the last one you attended?<br>**ENUMERATOR**: Enter code from CODEBOX #4CAPI: Skip pattern varies across countries<br>**ET**: if Q.5 (CODEBOX#3)= 21;22;24; 25;32-40; 43-48; Go to Q.7 ; if Q.5= 00-14;16-17;26-31; 41-42; 88, 77 ►Skip to Q.8<br>**IN YC**: if Q.5 (CODEBOX#3)= 13-15 Go to Q.7 ; if Q.5= 00-12;28,29,77,79►Skip to Q.8<br>**IN OC**: if Q.5 (CODEBOX#3)= 09-10 Go to Q.7; if Q.5= 00;01-0-6,08,77,79►Skip to Q.8<br>**PE**: if Q.5 (CODEBOX#3)= 13-16, 19, 21, 22 Go to Q.7 ; if Q.5=00-11; 17, 18, 20, 77, 88►Skip to Q.8<br>**VN**: if Q.5 (CODEBOX#3)= 13-16 Go to Q.7; if Q.5=00-12,17,28,29,50,77,79►Skip to Q.8	[__ __]
**Q.7a**	What major?<br>**ENUMERATOR**: Write the name of the major. Please look for the right code at the end of the call and add it in the line below.	[________________]
**Q.7b**	**ENUMERATOR**: Enter code from CODEBOX #5	[__ __]
**Q.8**	When was the last time you attended full-time education?<br><br>**ENUMERATOR**: if the [YL Child] attended full-time education for the last time in the current year (2020) please ask and report in what month.<br><br>01-12= Jan-Dec 2020 ► Go to Q.9<br>13 = 2019 ► [ET,IN,VN skip to next section], PE: skip to Q.12<br>14 = Before 2019 ► [ET,IN,VN skip to next section], PE: skip to Q.12<br>77 = NK ► [ET,IN,VN skip to next section], PE: skip to Q.12<br>79 = Refused to answer ► [ET,IN,VN skip to next section], PE: skip to Q.12	[__ __]

**Q.9**	Why did you stop attending full-time education?<br>**ENUMERATOR**: do not read the options.<br><br>01 = Because of the quarantine, my school cancelled classes<br>02 = Because of the quarantine, my school offers virtual classess but I don't have the equipment<br>03 = Because of the quarantine, I couldn't pay the fees<br>04 = Because of the quarantine, I had to look for a job<br>05 = Because of the COVID, someone in the household became ill<br>06= The course has finished<br>07 = Other, specify (related to COVID or quarantine) : ______<br>08 = Other Specify: ______	[__ __]
**Q.10**	Since the lockdown/ Response to COVID (ET), have you engaged in any of these education or learning activities?<br>**ENUMERATOR**: read all options and mark all that apply	
	01 = Attend classes in person	☐
	02 = Virtual classes with lesson teacher (laptop/computer/smartphone)	☐
	03= Completed assignments provided by the teacher	☐
	04= Watched educational TV programs	☐
	05= Listened to educational programs on radio	☐
	06= Used learning apps	☐
	07 = Other Specify: ______	[____________]
**Q.11**	During the lockdown/ Response to COVID (ET), how were you in contact with your teachers?<br>**ENUMERATOR**: read all options and mark all that apply	
	00= I have never been in contact -> Go to next section	☐
	01 = SMS	☐
	02 = Online applications	☐
	03= Email	☐
	04= Telephone (audio)	☐
	05= WhatsApp/Telegram (ET)	☐
	06= Facebook	☐
	07 = Other Specify: ______	[____________]
**Q.12**	[CAPI=PE only]What do you think will be your **main activity** in the next 12 months?**ENUMERATOR**: Read all options and mark the alternative that applies the most	
	01= Studying	[__ __]
	02= Looking for a job	
	03= Work in a family business	
	04= Start my own business	
	05= Work as employees/wage worker	
	06=Help in home activities / taking care of family members	
	07=Others	

Section 7. Time Use

SAY: Now I want you to think about how you spent your time during a typical weekday when the country was in full lockdown in [MONTH_COUNTRY_SPECIFIC]/ ET: during the Response to COVID-19]

Q.1	**SAY**: Now I want you to think about how you spent your time during a typical weekday when the [ET, IN, PE: country was in full lockdown in] [VN: when the city/province is/was in social distancing] [IN, PE: MONTH_COUNTRY_SPECIFIC] / ET: when the Response to COVID-19 started]? [CAPI: do not specify any time for VN]<br><br>Do you agree, partially agree or disagree with the following statement:<br><br>01=Agree<br>02=Partially agree<br>03=Disagree<br>77=NK<br>88=NA	
01	I spent more time taking care of children than before	[__ __]
02	I spent more time on household chores than before	[__ __]
03	I spent more time working at home/from home than before	[__ __]
04	I spent more time working outside home than before	[__ __]
05	I spent more time studying/learning	[__ __]
06	I spent more time working in family business than before	[__ __]
07	I spent more time sleeping	[__ __]
08	I spent more time playing/doing nothing	[__ __]

For peer review only

Section 8. Employment and Earnings

8.1 Labour Force participation

SAY: Now I would like to ask you about your current and previous paid and unpaid work activities. This includes work done inside and outside the household, and EXCLUDES non-working activities (e.g. housewife, student, taking care of other household members, such as children, disabled, illed, elderly). Working as housemaid or childcare (not for relatives) should be considered as working activity.

**Q.1**	At any time during the last 12 months, did you work for at least 1 hour, in your own business, for a household member or for someone else?<br><br>00=No ► Skip to Q.6<br>01=Yes	○ No ○ Yes
**Q.2**	Thinking back to January-February (for PE)/December-February (for IN, VN)/ January-mid-March (for ET), just before the COVID crisis began, were you working for at least 1 hour, in your own business, for a household member or for someone else?<br><br>00=No<br>01=Yes	○ No ○ Yes
**Q.3**	During the **last week** (from Monday through Sunday), did you work for at least 1 hour, in your own business, for a household member or for someone else?<br><br>00=No<br>01=Yes ► Skip to "Section 8.2"	○ No ○ Yes
**Q.4**	Do you currently have a job even though you did NOT work **last week** (from Monday through Sunday)?<br><br>00=No ► Skip to Q.6<br>01=Yes	○ No ○ Yes
**Q.5**	Why were you not able to work as usual?<br>**FIELDWORKER**: Do not read the options, mark all that applies<br><br>[**CAPI**: Once this question is answered ► Skip to "Section 8.2"]	
01	Temporarily laid off for reduction of staff/less business due COVID-19	☐
02	Workplace closed and cannot work from home	☐
03	Reduction in working hours due to less business	☐
04	Temporarily absent	☐
05	Sick or not feeling well	☐
06	Need to care for ill relative	☐
07	Has to care of children because school is closed	☐
08	Has to help children with homework because school is closed	☐
09	More domestic work because of the outbreak	☐
10	More time needed to buy food and cook	☐
11	Abandoned the job to protect myself and the household from COVID-19	☐
12	Others	☐

**Q.6**	Did you look for work **last week** (from Monday through Sunday)?<br><br>00=No<br>01=Yes ► If Q.1=00, skip to Subjective Wellbeing and Mental Health section;<br>Q.1=01; Q.2=01; Q.3=00; Q.4=00 -> Go to Section 8.3 and Section 8.4<br>Q.1=01; Q.2=00; Q.3=00; Q.4=00 -> Go to Section 8.4	○ No ○ Yes
**Q.7**	What is the main reason you did NOT look for work **last week**?<br><br>**ENUMERATOR**: do not read the option. Prompt th respondent to report the MAIN reason<br><br>01=housewife/childcare<br>02=student<br>03=disabled<br>04=illness<br>05=waiting for response from potential employer<br>06=waiting for recall by previous employer<br>07=waiting for busy season<br>08=lockdown<br>09=other, specify<br><br>Once this question is answered ► If Q.1=00, skip to Subjective Wellbeing and Mental Health section;<br>Q.1=01; Q.2=01; Q.3=00; Q.4=00 -> Go to Section 8.3 and Section 8.4<br>Q.1=01; Q.2=00; Q.3=00; Q.4=00 -> Go to Section 8.4	[__ __]

CAPI: these are the 10 potential ending with instructions on which one of the next sections on labor market should be administered:

01	Q.1=00 -> Q.6==00 -> Q.7 > Section 9.1 Subjective wellbeing and mental health
02	Q.1=00 -> Q.6==01 -> Section 9.1 "Subjective wellbeing and mental health"
03	Q.1=01 -> Q.2==00 -> Q.3==00 -> Q.4==00 -> Q.6==01 -> Go to Section 8.4
04	Q.1=01 -> Q.2==00 -> Q.3==00 -> Q.4==00 -> Q.6==00 -> Q.7 -> Go to Section 8.4
05	Q.1=01 -> Q.2==00 -> Q.3==00 -> Q.4==01 -> Q.5 -> Section 8.2 and Section 8.4
06	Q.1=01 -> Q.2==00 -> Q.3==01 -> Section 8.2 and Section 8.4
07	Q.1=01 -> Q.2==01 -> Q.3==00 -> Q.4==00 -> Q.6==01-> Go to Section 8.3 and Section 8.4
08	Q.1=01 -> Q.2==01 -> Q.3==00 -> Q.4==00 -> Q.6==00 -> Q.7 -> Go to Section 8.3 and Section 8.4
09	Q.1=01 -> Q.2==01 -> Q.3==00 -> Q.4==01-> Q.5 -> Section 8.2 and Section 8.3 and Section 8.4
10	Q.1=01 -> Q.2==01 -> Q.3==01 -> Section 8.2 and Section 8.3 and Section 8.4

For peer review only - http://bmjopen.bmj.com/site/about/guidelines.xhtml

8.2 Main activity in the past 7 days

CAPI: This sub-section will be asked only if [YL Child] reported having worked **at least one hour in the last 7 days or has a job but couldn't work last week** (Q.3=01 **or** Q.4=01 in the "Section 8.1 Labour force participation").

SAY: Now I would like to ask you about your **most important paid or unpaid work activitities during the last 7 days**. This includes work done inside and outside the household, and EXCLUDES non-working activities (e.g. housewife, student, taking care of other household members, such as children, disabled, illed, elderly). Working as housemaid or childcare (not for relatives) should be considered as working activity. If you has a job but you couldn't work last week please still refer to the job you have to answer the following questions.

FIELDWORKER: If the YL Child report more than one activity, record the **most important** paid or unpaid work activity. The most important activity is the activity on which the [YL Child] spent more time on in the last 7 days.

**Q.1**	Description of work activity<br><br>**ENUMERATOR:** Describe the work activity.	[_____]
**Q.2**	Type of work activity<br><br>**ENUMERATOR:** Record the most important paid or unpaid work activity.<br><br>Enter code from **CODEBOX #6**	[__ __]<br><br>Specify _________
**Q.3**	Economic sector code of the job/occupation<br><br>Enter code from **CODEBOX #7**	[__ __]
**Q.4**	Who do you do this activity for?<br><br>01=Private company/enterprise or cooperative<br>02=For a household member<br>03=Other private individual/household (excluding own household)<br>04=Public sector/government<br>05=A rural public works program<br>06=Own account/self-employed (own business or farm)<br>07= Other, specify<br><br>**ENUMERATOR:** In case of more than one type of employer in the same activity, refer to the most important employer in terms of time spent.	[__ __]<br><br>Specify _________

CAPI: Q.5-Q.6 are different for IN/PE/ET and for VN

ET, IN,PE version

Q.5	**ENUMERATOR:** Specify the average number of weeks worked in the month.<br>Enter 01-04<br>-79=NK	[__ __]
Q.6	**ENUMERATOR:** Specify the average number of days per week Days per weekEnter 01-07-79=NK	[__ __]
VN version		
Q.5	**ENUMERATOR:** Specify the average number of months worked in the year.<br>Enter 01-12<br>-79=NK	[__ __]
Q.6	**ENUMERATOR:** Specify the average number of days worked in the month<br>Enter 0-31<br><br>-79=NK	[__ __]
Q.7	**ENUMERATOR:** Record the average number of hours per day.<br><br>Hours per day<br>Enter 01-24<br>-79=NK	[__ __]
Q.8	For how many months have you performed this activity in the last 12 months?<br><br>Months per year<br>Enter 01-12<br>-79=NK	[__ __]
Q.9	Which form of payment was received or is expected from this activity?<br>00=None ► Skip to Q.14<br>01= Cash<br>02= In-kind (non-cash payment) ► Skip to Q.13<br>03= Both in cash and in-kind<br>04= Debt relief<br>05=Pocket money<br>06=Other, specify<br><br>77=NK ► Skip to Q.14<br>79=Refused to answer ► Skip to Q.14	[__ __]

**Q.10**	What period of time does this payment usually cover?<br><br>**ENUMERATOR:** If payment is reported in different periods, please convert to the most convenient measure.<br><br>01= Per hour ► Skip to Q.12<br>02= Per day ► Skip to Q.12<br>03= Per week ► Skip to Q.12<br>04= Per month ► Skip to Q.12<br>05= Per year ►Skip to Q.12 [ENUMERATOR: prompt the respondents to use a different period; report yearly earning only if absolutely needed]<br>06= Per piece<br>07= Fortnightly ► Skip to Q.12<br>08= Other, specify ►Skip to Q.12<br><br>77=NK ► Skip to Q.16<br>79=Refused to answer ► Skip to Q.16	[__ __]

**Q.11**	How many pieces are produced by day?Enter number of pieces77=NK79=Refused to answer	[__ __]	
**Q.12-Q.13**	What are the net earnings per *[prefill from Q10]* from this activity?<br><br>**ENUMERATOR**: This asks for NET earnings (the sum of all wages/salaries, tips, gratuities, bonuses and the value of any in-kind payment after deducting taxes and any other work-related payments). If own business, make sure to capture profits/self-determined wage assigned to respondent from this activity, net of production costs. If in-cash or in-kind payment is non-existent, assign a zero to the corresponding payment.	**Q.12**<br><br>In cash<br>Enter in currency unit<br><br>-8888=NA<br><br>[__________]	**Q.13**<br><br>In-kind<br>Enter value in currency unit<br><br>-8888=NA<br><br>[__________]

**Q.14**	Apart from this activity, did you do another activity in the last week that was paid or is expected to be paid?<br><br>00= No ► Skip to Q.16<br>01= Yes	○ No ○ Yes

**Q.15**	We would like to ask you about your total earnings from all paid activities, including the main working activity.<br><br>What period of time is better suited to calculate your total earnings?<br><br>01= Per hour<br>02= Per day<br>03= Per week<br>04= Per month<br>05= Per year [**ENUMERATOR**: prompt the respondents to use a different period; report yearly earning only if absolutely needed]<br>07= Fortnightly<br>08= Other, specify<br><br>77=NK ► Skip to Q.17<br>79=Refused to answer ► Skip to Q.17	[__ __]
**Q.16**	What are the total earnings per [Period of time, Q.15] from all paid activities, including the main working activity?<br><br>77=NK ► Skip to Q.17<br>79=Refused to answer ► Skip to Q.17<br><br>**ENUMERATOR**: As for the main activity earnings this refer to the NET earnings (the sum of all wages/salaries, tips, gratuities, bonuses and the value of any in-kind payment after deducting taxes and any other work-related payments).	Enter value in currency unit<br><br>-8888=NA

WORKING ARRANGEMENT- ALL WORKERS

**Q.17**	Do you have health insurance paid by the employer?<br><br>00=No<br>01=Yes<br>77=NK	O No O Yes O NK
**Q.18**	Do you have a written contract for this [MAIN ACTIVITY]?<br>00=No ► Skip to next section<br>01=Yes	O No O Yes

Q.19	What is the type of contract for this [MAIN ACTIVITY]?<br><br>CAPI: the options are country specific	
	ETHIOPIA<br>01 = Permanent contract ► Skip to next section<br>02= Fixed term/Temporary contract<br>03= Trial period<br>04= Youth training agreements / pre-professional practices<br>05=Other, specify	[__ __]
	**INDIA**<br>01 = Permanent contract ► Skip to next section<br>02= Fixed term/Temporary contract<br>03= Probation period<br>04= Apprenticeship<br>05=Internship<br>06=Other, specify	[__ __]
	**PERU**<br>01 = Permanent contract ► Skip to next section<br>02= Fixed term contract<br>03= Trial period<br>04= Youth training agreements / pre-professional practices<br>05= Independent contract (RPH)<br>06= Special regime for the public sector (CAS)<br>07=Other, specify	[__ __]
	**VIETNAM**<br>01=Permanent contract, go to next section; ► Skip to next section<br>02=Fixed term contract;<br>03=Contract for specific work ► Skip to next section<br>04=Other, specify	[__ __]
**Q.20**	What is the duration of the contract that you have?<br><br>01=3 months or less<br>02=More than 3 months to 6 months<br>03=More than 6 months to 1 year<br>04=More than 1 year	[__ __]

8.3 Main activity before COVID-19

FIELDWORKER: This sub-section will be asked only if [YL Child] reported having worked **at least one hour during December-February, just before the COVID crisis began (as reported in the Labour Force Participation Section]**

[**CAPI**: Section 8.3 should be enabled only if Q.2=01 in the "Labour force participation" sub-section. If Q.2=0, skip to Section 8.4]

SAY: Now I would like to ask you about your most important paid or unpaid work activities during January-February (for PE)/December-February (for IN, VN)/ January-mid-March (for ET), just before the COVID crisis began. This includes work done inside and outside the household, and **EXCLUDES** non-working activities (e.g. housewife, student, taking care of other household members, such as children, disabled, illed, elderly). Working as housemaid or childcare (not for relatives) should be considered as working activity.

FIELDWORKER: Record the **most important** paid or unpaid work activity. The most important activity is the activity on which the [YL Child] spent more time on in the week before [OUTBREAK_DATE]

**Q.1**	Was this the same activity you did in the last 7 days? Is it with the same employer?<br><br>**ENUMERATOR**: for those who didn't work in the past 7 days but has a job please ask the respondent to refer to that job in answering this question<br><br>00=Different activity and different employer -> Go to Q.2 and continue<br>01= Same employer but different activity -> Go to Q.2 and continue (do not ask Q.5)<br>02= Same activity but different employer > Go to Q.5 and continue<br>03= Same activity same employer-> Go to Q.6	[__ __]
**Q.2**	Description of work activity<br><br>**ENUMERATOR:** Describe the work activity.	[________]
**Q.3**	Type of work activity<br><br>**ENUMERATOR:** Record the most important paid or unpaid work activity.<br><br>Enter code from **CODEBOX #6**	[__ __]<br><br>Specify<br>________
**Q.4**	Economic sector code of the job/occupation<br>Enter code from **CODEBOX #7**	[__ __]

**Q.5**	Who do you do this activity for?<br><br>01=Private company/enterprise or cooperative<br>02=For a household member<br>03=Other private individual/household (excluding own household)<br>04=Public sector/government<br>05=A rural public works program<br>06=Own account/self-employed (own business or farm)<br>07= Other, specify<br><br>**ENUMERATOR:** In case of more than one type of employer in the same activity, **refer to the most important employer <u>in terms of time spent</u>.**	[__ __]
**Q.6**	Which form of payment was received or is expected from this activity?<br><br>00=None ► Skip to Q.11<br>01= Cash (including pocket money)<br>02= In-kind (non-cash payment) ► Skip to Q.10<br>03= Both in cash and in-kind<br>04= Debt relief<br>05=Pocket money<br>06=Other, specify<br><br>77=NK ► Skip to Q.11<br>79=Refused to answer ► Skip to Q.11	[__ __]<br><br>Specify<br>________
**Q.7**	What period of time does this payment usually cover?<br><br>**ENUMERATOR:** If payment is reported in different periods, please convert to the most convenient measure.<br><br>01= Per hour ► Skip to Q.9<br>02= Per day ► Skip to Q.9<br>03= Per week ► Skip to Q.9<br>04= Per month ► Skip to Q.9<br>05= Per year ► Skip to Q.9<br>06= Per piece<br>07= Fortnightly ► Skip to Q.9<br>08= Other, specify ► Skip to Q.9<br><br>77=NK<br>79=Refused to answer	[__ __]

**Q.8**	How many pieces are produced by day?<br><br>Enter number of pieces<br><br>77=NK<br>79=Refused to answer	[__ __]

Q.9-Q.10	What are the net earnings per [prefill from Q.7] from this activity?<br><br>**FIELDWORKER**: This asks for NET earnings (the sum of all wages/salaries, tips, gratuities, bonuses and the value of any in-kind payment after deducting taxes and any other work-related payments). If own business, make sure to capture profits/self-determined wage assigned to respondent from this activity, net of production costs. If in-cash or in-kind payment is non-existent, assign a zero to the corresponding payment.	**Q.9**<br>In cash<br><br>Enter in currency unit<br><br>-8888=NA<br><br>[___________]	**Q.10**<br>In-kind<br><br>Enter value in currency unit<br><br>-8888=NA<br><br>[___________]

**Q.11**	After the [OUTBREAK_DATE] did you move away from the village/town where your household is located?<br><br>01=Yes<br>00=No -> Skip to Section 8.4	○ No ○ Yes
**Q.12**	Why did you move?<br><br>**ENUMERATOR**: do not read the options. Mark only one. If the YL Child replies with two or more reasons aks about the main reason<br><br>01 Looking for work<br>02 Moved in with relatives<br>03 Going back to my original town<br>04 To access health services<br>05 Others, Specify _______________	[__ __]

8.4 Main activity during lockdown period

CAPI: Section 8.4 should be enabled if Q.1=01 in the "Section 8.1 Labour force participation". If Q.1=0, skip to next section
FIELDWORKER: This sub-section will be asked only if [YL Child] reported having worked **at least one hour in the past 12 months** in the Labor Force Participation Section

**Q.1**	During the lockdown/ET: COVID-19 Response, were you able to work either at your place of work or remotely? 01= Yes, working from work place -> Go to Q.302= Yes, working remotely -> Go to Q.303= Yes, combining remote work and working from workplace -> Go to Q.304= No -> Go to Q.2 and then go to Next section05= I did not have a job when lockdown/ET: COVID-19 Response started -> Go to Next Section	[__ __]

**Q.2**	Why not?<br><br>**FIELDWORKER**: Do not read the options, mark all that apply	
01	Temporarily laid off for reduction of staff/less business due covid-19	☐
02	Workplace closed and cannot work from home	☐
03	Reduction in working hours due to less business	☐
04	Temporarily absent	☐
05	Sick or not feeling well	☐
06	Need to care for ill relative	☐
07	Has to take care of children because school is closed	☐
08	Has to help children with homework because school is closed	☐
09	More domestic work because of the outbreak	☐
10	More time needed to buy food and cook	☐
11	Abandoned the job to protect myself and the household from COVID-19	☐
12	Others	☐

**Q.3**	Were/are you able to work as usual? Same number of working hours/amount of work?<br><br>**FIELDWORKER**: Do not read the options, mark all that apply	
01	Yes, same amount/hours of work	☐
02	No, reduction in hours/amount of work due to less business	☐
03	No, reduction in hours/amount of work to care for ill relative	☐
04	No, reduction in hours/amount of work to take care of children as school is closed	☐
05	No, reduction in hours/amount of work to help children with homework because school is closed	☐
06	No, reduction in hours/amount of work as more domestic work because of the outbreak	☐
07	No, reduction in hours/amount of work as more time needed to buy food and cook	☐
08	ETHIOPIA ONLY: No, reduction in hours/days/amount of work due to employers/government action to reduce the spread of covid-19 between workers	☐
09	Others	☐

Section 9. Subjective wellbeing and Mental Health

9.1 Subjective wellbeing

SAY: Now, I'm going to ask you some questions about your wellbeing and I would like to make sure that you feel comfortable enough to answers those questions.

**Q.1**	Are you alone in the room? Or could you find a quiet space?<br><br>00=No -> **SAY**: Ok, don't worry. Just make sure that you are not on speakers, so the conversation is between the two of us only. Let me ask few questions and please remember that you can decide not to answer if you wish.<br><br>01=Yes -> **SAY**: Great. Just make sure that you are not on speakers, so the conversation is between the two of us only. Let me ask few questions then, and remember that you can decide not to answer if you wish	O No O Yes

SAY: Now, I want you to think of a ladder with nine steps. Suppose the ninth step, **at the very top**, represents the best possible life for you, and first step**, at the bottom** represents the worst possible life for you. Having in mind that scale…

**Q.2**	Where on the ladder do you feel you personally stand at the present time?<br><br>Record step number: 01-09<br><br>77=NK<br>79=Refused to answer	[__ __]

For peer review only

9.2 Mental Health

GAD-7 (Anxiety)

SAY: I am going to read you some questions and I want you to tell me whether these situations have occurred to you or not in t weeks. If this has happened to you, I will also ask you how often this happened

			**Q.4** How often the situation occurred in the last two weeks?
**Q.3-Q.4**	**FIELDWORKER:** read the table line by line.<br><br>**Q.3. SAY:** In the last two weeks, have you been…?<br><br>00=No, not at all<br>01=Yes, even if a little bit<br><br>**CAPI:** Enable Q.4, for those items where Q.3 =01. If the answer is 00=No, move to the next line<br><br>**ENUMERATOR:** please make sure that the [YL Child] understand that No means never not even for a moment or a day in the past two week.		01= Less than half the days<br>02=More than half the days<br>03=Nearly everyday<br>77=NK<br>79=RTA<br>88=NA
**01**	Feeling nervous, anxious or on edge	○ No, not at all<br>○ Yes, even if a little bit	[__]
**02**	Not being able to stop or control worrying	○ No, not at all<br>○ Yes, even if a little bit	[__]
**03**	Worrying too much about different things	○ No, not at all<br>○ Yes, even if a little bit	[__]
**04**	Trouble relaxing/ Can't relax	○ No, not at all<br>○ Yes, even if a little bit	[__]
**05**	Being so restless that it's hard to sit still	○ No, not at all<br>○ Yes, even if a little bit	[__]
**06**	Becoming easily annoyed or irritable	○ No, not at all<br>○ Yes, even if a little bit	[__]
**07**	Feeling afraid as if something awful might happen	○ No, not at all<br>○ Yes, even if a little bit	[__]

PHQ-8
Say: I am going to read you some questions and I want you to tell me whether these situations have ocurred to you or not. If thi happened to you, I also want to know how often have occurred in the last two weeks

			**Q.6** How often the situation occurred in the last two weeks?
**Q.5-Q.6**	**FIELDWORKER:** read the table line by line.<br><br>**Q.5. SAY:** In the last two weeks, have you been bothered by any of the following problems…?<br><br>00=No, not at all<br>01=Yes, even if a little bit<br><br>**CAPI:** Enable Q.6, for those items where Q.5 is 01=Yes. If the answer is 00=No, move to the next line<br><br>**ENUMERATOR**: please make sure that the [YL Child] understand that No means never not even for a moment or a day in the past two week.		01= Less than half the days<br>02=More than half the days<br>03=Nearly everyday<br>77=NK<br>79=RTA<br>88=NA
**01**	Little interest or pleasure in doing things	O No, not at all<br>O Yes, even if a little bit	[__]
**02**	Feeling down, depressed or hopeless	O No, not at all<br>O Yes, even if a little bit	[__]
**03**	Trouble falling or staying asleep, or sleeping too much	O No, not at all<br>O Yes, even if a little bit	[__]
**04**	Feeling tired or having little energy	O No, not at all<br>O Yes, even if a little bit	[__]
**05**	Poor appetite or overeating	O No, not at all<br>O Yes, even if a little bit	[__]
**06**	Feeling bad about yourself - or that you are a failure or have let yourself or your family down	O No, not at all<br>O Yes, even if a little bit	[__]
**07**	Trouble concentrating on things, such as reading the newspaper or watching television	O No, not at all<br>O Yes, even if a little bit	[__]
**08**	Moving or speaking so slowly that other people could have noticed. Or the opposite - being so fidgety or restless that you have been moving around a lot more than usual.	O No, not at all<br>O Yes, even if a little bit	[__]

SAY: Now, I would like to ask you

**Q.7**	[CAPI: PE only] Since the beginning of the pandemic, in general, how do you think your relationship **with your father and mother** has changed?01= It has deteriorated a lot02= It has deteriorated a little03= It remains the same04= It has improved05= It has improved a lot79 = RTA 88 = NA	[__ __]
**Q.8**	[CAPI: PE only]<br><br>Since the beginning of the pandemic, in general, how do you think your relationship **with the rest of household members** has changed?<br><br>01= It has deteriorated a lot<br>02= It has deteriorated a little<br>03= It remains the same<br>04= It has improved<br>05= It has improved a lot<br>79 = RTA<br>88 = NA	[__ __]

**Q.9**	What are you most looking forward to do after COVID-19 is over?<br><br>**FIELDWORKER**: Do not read the options. If the respondent mention more than one please ask about the most desired one.	
01	Going back to education	[__ __]
02	Going back to work/workplace	
03	Visit family	
04	Visit friends	
05	Go to the city center	
06	Go to the park	
07	Go out for dinner	
08	Play a team sport	
09	To go to church/mosque/temple/ceremonies/weddings	
10	None	
11	Other (specify)	[____________]

Concluding remarks

**Q.1**	Is there anything else you would like to tell us?	Specify [___________]

SAY: YL Child´s name It has been very good for me to talk to you, and as I have said, in these circumstances/in these moments your information is very interesting and important.

In [November/ December, DATE OF PHONE SURVEY THIRD CALL] I will call you again to find out how you and your family are and ask you few questions to understand better how do you feel, how is your family. The information that you and the other Young Lives participants will provide us with will be extremely valuable to understand how things are changing and what policies can be effective in improve the situation. The final survey takes approximately 15 minutes.

**Q.2**	Is there a day of the week that usually works better for you?<br>**ENUMERATOR**: Mark all that apply	
01	Monday	☐
02	Tuesday	☐
03	Wednesday	☐
04	Thursday	☐
05	Friday	☐
06	Saturday	☐
07	Sunday	☐
**Q.3**	Is it easier to reach you in the mornings or afternoons?<br>**ENUMERATOR**: Read options and mark all that apply	
	01=Mornings	☐
	02=Afternoons	☐
	03= Evening (after dinner)	☐

Other comments	Specify [________________________________]

For peer review only - http://bmjopen.bmj.com/site/about/guidelines.xhtml

**SAY**: You will remember that whenever we have visited you, at the end of the interview, in gratitude to the time that you have given us to answer all the questions, we have given you a [PE: a small present, e.g. backpack, watch, mini radio, etc.); ET: a small amount of money or transferred mobile card; IN: money purse, vanity bag, etc; VN: a small amount of money], right?<br><br>[PE and IN] As I mentioned at the beginning, this time we can't visit the participants in their homes, so, only for this time, each participant will be given [PE: S /. 50 soles, which is the approximate equivalent to the value (cost) of the present], [IN: Rs. 600, which is the approximate equivalent to the value (cost) of the present] , as we anticipated to you in the previous call. Would you prefer to receive the 50 soles now or in November, at the end of the survey?"	[______________]
[ET and VN] As I mentioned at the beginning, this time we can't visit the participants in their homes, so, only for this time, each participant will be given [ET: Birr 100 or Mobile card which is the approximate equivalent to the value of the money] [VN: VND 50,000 or a mobile card which is he approximate equivalent to the value of the money][The delivery of the money would be made by means of a "Money Order" by : [PE: Banco de la Nación; ET: Commercial Bank of Ethiopia; VN: Commercial Bank of Viet Nam] or any other bank that the participant indicates, so that they can collect it from there. Or if you don't have access to a Bank [PE: you can receive the reward from any BCP or Banco de la Nacion Agent located near your living place; ET: we may send you mobile card equivalent to the money. Or if you prefer to receive the money, we can send you the amount through our local guide. Please let us know your preference; VN: we may send you mobile card equivalent to the money. Please let us know your preference.] [IN: The amount will be sent through any nationalised banks by RTGS or we don't want to give us your bank account or you don't have one, we will send the amount by "Money Order" and it will take 4 or 5 days to reach them through Post office. In both cases we will send a photocopy of the receipt].Now, before I say goodbye, I would like to verify your address and your contact numbers. -> **Go to next section (Locating Information)**	[______________]

For peer review only

Locating Information and contact info
[No publicly archived]

SAY: That's all for now, again: Thank you very much for your help and remember that in [between November and December, DATE OF PHONE SURVEY THIRD CALL] I am calling again.

Thank you very much and stay safe!

For peer review only

Comments

CAPI: This section should be enabled for all respondents regardless of whether they decide to participate or not
FIELDWORKER: Please use this section to take notes whether the YL child asked for further information or if you noticed anything that gave you cause for concern in relation to this young person/family. On this second aspect, please make sure to refer those cases to the field coordinator as soon as possible.

CAPI: enable the following comment fields C.1 and C.2 for all respondents, including those not going ahead with the survey (farewell F1 and farewell F2)
ENUMERATOR please provide with the following information:

**C.1**	Has the [YL Child] asked for information?<br><br>[CAPI] : If No --> Skip to C.3	○ No(0) ○ Yes(1)
**C.2**	Related to what?<br>ENUMERATOR: Do not read. Mark all that apply	
01	related to COVID-19 (symptoms, treatments, how it is transmitted etc..)	☐
02	related to health insurance	☐
03	related to economic support offered by the government	☐
04	related to jobs	☐
05	related to education	☐
06	related to scholarship	☐
07	relate to present offered to YL survey participations	☐
08	related to violence	☐
09	related to mental health	☐
10	Others, Specify	☐
11	Specify	[______________]
Any other comments?		

**C.3**	Was there anything that gave you cause for concern in relation to this young person/family?	○ No(0) ○ Yes(1)
**C.4**	Related to what?<br>ENUMERATOR: Mark all that apply	
01	Poor health	☐
02	Struggle to get something to eat	☐
03	Anxious, distressed about not having a job	☐
04	Related to personal violence	☐

05	Related to family violence	☐
06	Related to mental health	☐
07	Others, Specify	☐
08	Specify	[______________]
Any other comments?		________________________________

For peer review only

Enumerator Statement

PERU

I declare that I have complied with the consent process, informing the Young Lives participant everything related to the survey, reading the consent text, and answering all of his/her doubts, questions and/or queries. Also, I regulate my conduct in accordance with the requirements of the Young Lives Code of conduct.

If the above statement is true, please fill in:

Your full name in capital letters [**CAPI**: pre-fill using Data Handlers information]	[____]
Your 8-digit ID national number	[_ _ _ _ _ _ _ _]
The full name of the YL participant [**CAPI**: pre-fill using Data Handlers information]	[____]
The 8-digit ID national number of the YL participant	[_ _ _ _ _ _ _ _]

ETHIOPIA

I declare that I have complied with the consent process, informing the Young Lives participant everything related to the survey, reading the consent text, and answering all of his/her doubts, questions and/or queries. Also, I regulate my conduct in accordance with the requirements of the Young Lives Code of conduct.

If the above statement is true, please fill in:

Your full name in capital letters [**CAPI**: pre-fill using Data Handlers information]	[____]
Your Kebele ID number [**CAPI**: pre-fill using Data Handlers information]	[____]
The full name of the YL participant	[____]

INDIA

I declare that I have complied with the consent process, informing the Young Lives participant everything related to the survey, reading the consent text, and answering all of his/her doubts, questions and/or queries. Also, I regulate my conduct in accordance with the requirements of the Young Lives Code of conduct.

If the above statement is true, please fill in:

Your full name in capital letters [**CAPI**: pre-fill using Data Handlers information]	[____]
The full name of the YL participant [**CAPI**: pre-fill using Data Handlers information]	[____]

VIETNAM

I declare that I have complied with the consent process, informing the Young Lives participant everything related to the survey, reading the consent text, and answering all of his/her doubts, questions and/or queries. Also, I regulate my conduct in accordance with the requirements of the Young Lives Code of conduct.

If the above statement is true, please fill in:

Your full name in capital letters [**CAPI**: pre-fill using Data Handlers information]	[____]
The full name of the YL participant [**CAPI**: pre-fill using Data Handlers information]	[____]

Codeboxes

CODEBOX #1
Source of drinking water

ET

01=Bore well in dwelling/yard/plot
02=Bore well in another location
03=Bought water (delivery or bottled)
04= Piped into dwelling/yard/plot
05=Piped into neighbours dwelling/yard/plot
06=Piped into relatives' dwelling/yard/plot
07=Piped (communal)
08=Protected spring or well in dwelling/yard/plot
09=Protected spring or well in another location
10=Public standpipe/tubewell
11=Rain water (during rainy season)
12=Tubewell in dwelling/yard/plot
13=Unprotected well/spring/pond/river/stream/ canal/lake
14=Water tank in dwelling/yard/plot
15=Water tank in another location
16=Other source in dwelling/yard/plot, specify ________
17=Other source in another location, specify ________

PE

01= Piped water to the house/plot (public network)
02 = Piped water to the house/plot (non-public network)
03= Well, tube well with hand pump in house/plot
04= Well, tube well with hand pump in another location
05= Common tap, water fountain, public well
06= Lake, river,/ ditch / stream/ natural spring/unprotected canal
07= Water truck delivery
08= Unprotected Well in another location
09= Unprotected Well in house/plot
10 = Piped water from neighbors/relatives
11 = Untreated water from neighbors/ relatives
12=Other source in house/plot, specify ________
13=Other source in another location, specify ________
77 = NK
88 = NA

For peer review only

IN, VN

01=Bore well in the dwelling/yard/plot
02=Bore well in another location
03=Bought water (delivery or bottled)
04= Piped into dwelling/yard/plot
05=Piped into neighbours dwelling/yard/plot
06=Piped into relatives' dwelling/yard/plot
07=Protected spring or well in dwelling/yard/plot
08=Protected spring or well in another location
09=Public standpipe/tubewell
10=Rain water
11=Tubewell in dwelling/yard/plot
12=Unprotected well/spring/pond/river/stream/ canal
13=Unprotected water tank in dwelling/yard/plot
14=Unprotected water tank in another location
15=Water tank (community/protected)
16=Other source in dwelling/yard/plot, specify ________
17=Other source in another location, specify ________

CODEBOX #2

Toilet facility

ET

01=Flush toilet/septic tank (private)
02=Flush toilet/septic tank (shared)
02=Forest/field/open place
05=Pit latrine (communal)
06=Pit latrine (household's)
07=Bucket
10=Others (household only) [Specify_____________]
11=Others (communal) [Specify_____________]

IN

01=Flush toilet/septic tank
02=Forest/field/open place
03=Neighbours toilet
05=Pit latrine (communal)
06=Pit latrine (household's)
07=Relatives toilet
09=Toilet in health post
10=Others (household only) [Specify_____________]
11=Others (communal) [Specify_____________]

PE

01= Flush toilet/septic tank
02=Forest/field/open place
03=Neighbours toilet

For peer review only

04= None (river, stream, irrigation ditch)
05= Pit latrine (communal)
06= Pit latrine (household's)
07=Relatives toilet
09=Toilet in health facility / market
10=Others (household only) [Specify____________]
11=Others (communal) [Specify____________]

VN

01= Flush toilet/septic tank
02=Forest/field/open place
03=Neighbours toilet
05= Pit latrine (communal)
06= Pit latrine (household's)
10=Others (household only) [Specify____________]
11=Others (communal) [Specify____________]

CODEBOX #3
Education

ET

00 = None
01-08 = Grade 1-8 (Primary)
09-10= Grade 9-10 (Secondary First Cycle)
11-12 = Grade 11-12 (Secondary Second Cycle Preparatory Programme)
13 = First cycle of primary teaching certificate (grade 1-4)/1st year
14 = First cycle of primary teaching certificate (grade 1-4)/2nd year
17 = Second cycle of primary teaching certificate (grades 5-8)/1st year
16 = Second cycle of primary teaching certificate (grades 5-8)/2nd year
21 = TVET/1st year/level (include diplomas such as accounting diploma)
22 = TVET/2nd year/level (include diplomas such as accounting diploma)
26 = TVET/3rd or 4th year/level (include diplomas such as accounting diploma)
27=Secondary education, teacher (diploma holder)/1st or 2nd year
30 = Secondary education, teacher (bachelor's degree holder and above)/1st or 2nd or 3rd year
31=Preschool teacher certificate (6 months to one year)
32 = Undergraduate degree (1st year, regular (R))
33 = Undergraduate degree (2nd year, regular (R))
34 = Undergraduate degree (3rd year, regular (R))
35 = Undergraduate degree (4th year, regular (R))
36 = Undergraduate degree (1st year or equivalent, non-regular (NR), summer/distant/ evening/weekend student)
37 = Undergraduate degree (2nd year or equivalent, non-regular (NR), summer/distant/ evening/weekend student)

For peer review only

38 = Undergraduate degree (3rd year or equivalent, non-regular (NR), summer/distant/ evening/weekend student)
39 = Undergraduate degree (4th year or equivalent, non-regular (NR), summer/distant/ evening/weekend student)
43= Undergraduate degree (5rd year, regular (R))
44 = Undergraduate degree (6th year, regular (R))
45= Undergraduate degree (6th year, regular (R))
46= Undergraduate degree (5th year or equivalent, non-regular (NR), summer/distant/ evening/weekend student)
47 = Undergraduate degree (6th year or equivalent, non-regular (NR), summer/distant/ evening/weekend student)
48 = Undergraduate degree (7th year or equivalent, non-regular (NR), summer/distant/ evening/weekend student)
40=Masters or doctoral at university
28=Adult literacy
29=Religious education
88= NA
77=NK
41 =Other, (specify)________

IN
YC

00 = None
01-12= Grade
13=Post-secondary technological institute
14=Vocational
15=University degree (graduate)
28=Adult literacy
29=Religious education
77=NK
79=Refused to answer

OC

00=No certificate (or Pre-school)
01=Primary (Class 5)
02=Upper primary (Class 8)
03=Matriculation certificate (Class 10)
04=Senior Secondary school certificate / Intermediate certificate (Class 12)
05=ITI certificate
06=Diploma in technical education
08=Other, specify
09=University degree (graduate)

For peer review only

10=Post-graduate university degree (completed)
77=NK
79=Refused to answer

PE

00 = None
01-11 = Grade
13 = Incomplete technical or pedagogical institute (incl. "Escuela de Sub Officiales")
14 = Complete technical or pedagogical institute (incl. "Escuela de Sub Oficiales")
15 = Incomplete university (incl. "Escuela de Oficiales")
16 = Complete university (incl. "Escuela de Oficiales")
17 = Adult literacy program
19 = Masters or doctoral at university
18 = Other (Specify)
20 = Some form of formal or informal preschool
21 = Cent. Técnico Productivo CETPRO/Cent. Edu. Ocupacional CEO Incomplete
22 = Cent. Técnico Productivo CETPRO/ Cent. Edu. Ocupacional CEO Complete
77 = NK
88 = NA

VN

00 = None
01-12= Grade
13=Post-secondary technological institute
14=Vocational
15=Degree (graduate)
16=Post-graduate degree (e.g. Masters, PhD.)
17=Other, specify
28=Adult literacy
29=Religious education
50=Any pre-primary
77=NK
79=Refused to answer

CODEBOX #4
Type of educational institution

ET

01 = Private/domestic
02= Public (part student fees, part government funded);
03= Mission (NGO/Charity/Religious) with fees
04= Mission (NGO/Charity/Religious) free of charge
05= Government funded
06=Others, specify
07=Community school
08=Private-foreign like UNISA, SRI-SAI
09=domestic-foreign joint programme
77=NK
88=NA

IN

01=Private (unaided)
02=NGO/Charity/not-for-profit
03=Public (Government)
04=Informal or non-formal Community (e.g. mothers' cooperative)
06=Charitable trust
07=Bridge school
08=Mix of public and private (private aided)
11=Other, specify
77=NK
79=Refused to answer

PE

01 = Private, for profit
02 = NGO / Charity / Church (not for profit),
03 = Public, local municipality
04 = Public, of the national government,
06 = Informal,
07 = Half public / Half Private
05 = Other (specify)
08 = Center of public alternative basic education
09 = Center of private alternative basic education

For peer review only

10 = Public, Colegio de Alto Rendimiento (COAR)
11 = Public, Jornada Escolar Completa (JEC)
77 = NK,
79=RTA,
88 = NA

VN

01 =Private
02=NGO/Charity/ Religious (not for profit)
03=Public
06=Informal
07=Half public/Half Private
08=Centre for continuing education
**05=Others, specify**
**77=NK**
**88=NA**

For peer review only

Codebox #5:
Major classification

ET

	**Engineering and Technology**
1	Chemical Engineering
2	Civil Engineering / construction
3	Electrical Engineering
4	Mechanical Engineering
5	Information Technology/ Computer science
	**Natural and Computational Sciences**
10	Biology, Physics, or Chemistry
11	Mathematics or statistics
12	Geology or earth science
	**Medicine and Health Sciences**
20	Medicine
21	Health officer
22	Laboratory/radiology/ Midwifery/Nursing
23	Pharmacy
24	Animal Science/veterinary
	**Agriculture and Life Sciences**
30	Animal Production
31	Plant science/Crop Production
32	Natural resource management
33	development agent
	**Business and Economics**
40	Accounting / Management
41	Economics
42	Public Administration
	**Education and journalism**
50	Local languages and literature
51	Foreign language and literature
52	Education Development and Planning Management
53	Curriculum
54	Journalism & Communication
	**Social Science and Humanities**
60	Geography or history
61	Psychology
62	Law / political science
63	Humanities
64	Social work and/ Social Anthropology
70	Others (please mention)

For peer review only

IN

CODES
1=Teacher training
2=Fine Arts
3=Humanities and Social Sciences
4=Sciences
5=Journalism and Communication
6=Business and management
7=Law and Legal Services
8=Mathematics and Statistics
9=Computer and Information Technology
10=Food processing
11=Other technologies (Bio-technology etc)
12=Engineering
13=Agriculture, Forestry and Aquaculture
14=Veterinary
15=Medical, Pharmacy and Health Sciences
16=Tourism, Hotels, Recreation and Sports
17=Armed force and Police
18=Others Specify

PE

CODES
11=Preschool and Primary school
12=Secondary school
13=Physical Education
14=Special Education
15=Artistic Education
16=Technical Education
19=Other educational majors
21=Humanities
22=Arts
31=Social Sciences and Sciences of behaviour
32=Communication Sciences
33=Administration and Commercial Sciences
34=Economic and Accounting Sciences
35=Law, Political and Legal Sciences
41=Life Sciences
42=Physical and Chemical Sciences
43=Mathematics and Statistics
44=Computing
51=Systems and Telecommunications Engineering
52=Industrial Engineering and Production
53=Construction, Sanitary and Architecture Engineering

For peer review only

59=Other Engineering
61=Agriculture, Forestry and Aquaculture
62=Veterinary
71=Health Sciences
01=Armed Forces
02=Police Forces

VN

**CODES**
1=Education and teacher training
2=Art
3=Humanities
4=Social Sciences and Sciences of behaviour
5=Journalism and information
6=Business and management
7=Law and Legal Services
8=Life Sciences
9=Natural sciences
10=Mathematics and Statistics
11=Computer and Communications
12=Food processing
13=Other technologies
14=Construction and Architecture Engineering
15=Agriculture, Forestry and Aquaculture
16=Veterinary
17=Health Sciences
18=Social services
19=Tourism, Hotels, Recreation and Sports
20=Transports
21=Environmental Protection
22=Armed force and Police

For peer review only

Codebox #6:
Work activities

AGRICULTURE & ALLIED AGRICULTURE
01=Self Employed<br>(Food crops)
02=Self Employed<br>(Non-food, including horticulture, sericulture and floriculture)
03 = Self Employed (Aquaculture)
04 = Self Employed<br>(Livestock)

05=Wage Employment (Agriculture)
06=Annual Farm Servant
07=Other (allied) agriculture, specify
**NON-AGRICULTURE**
08=Self Employed (Manufacturing)
09 = Self Employed (Services)
10= Self Employed (Business)
11 = Self Employed (Other non-Agriculture)
12=Wage Employment (Unsalaried/ irregular; Non-agriculture)
13=Regular Salaried Employment
15 = Begging
19=Other non-agriculture, specify

Codebox #7:
Economic sector

01=Agriculture, forestry and fishing
02=Mining and quarrying
03=Manufacturing
04=Electricity, gas, steam and air conditioning supply
05=Water supply; sewerage, waste management and remediation activities
06=Construction
07=Wholesale and retail trade; repair of motor vehicles and motorcycles
08=Transportation and storage
09=Accommodation and food service activities
10=Information and communication
11=Financial and insurance activities
12=Real estate activities
13=Professional, scientific and technical activities
14=Administrative and support service activities
15=Public administration and defence; compulsory social security
16=Education
17=Human health and social work activities
18=Arts, entertainment and recreation
19=Other service activities
20=Activities of households as employers; undifferentiated goods- and services-producing activities of households for own use
21=Activities of extraterritorial organizations and bodies
77=NK
88=NA
79=RTA

For peer review only

Supplementary tables

Supplement to: Impact of the COVID-19 Pandemic on anxiety and depression symptoms of young people in the Global South: evidence from a four-country cohort study

Supplementary Table 1. Sample size 3
Supplementary Table 2. Attrition rates (%) 3
Supplementary Table 3. Non-response rate to mental health questions (%)........ 4
Supplementary Table 4. Measures of reliability and internal consistency........ 4
Supplementary Table 5. Sample description 5
Supplementary Table 6. Logistic regression results: Symptoms of at least mild anxiety (Younger Cohort only) 7
Supplementary Table 7. Logistic regression results: Symptoms of at least mild depression (Younger Cohort only) 8
Supplementary Table 8. Logistic regression results: Symptoms of at least mild anxiety (Older Cohort only) 10
Supplementary Table 9. Logistic regression results: Symptoms of at least mild depression (Older Cohort only) 11
Supplementary Table 10. Ethiopia: Means of continuous control variables by at least mild anxiety/depression 14
Supplementary Table 11. India: Means of continuous control variables by at least mild anxiety/depression 15
Supplementary Table 12. Peru: Means of continuous control variables by at least mild anxiety/depression 16
Supplementary Table 13. Vietnam: Means of continuous control variables by at least mild anxiety/depression 17
Supplementary Table 14. Mental health outcomes by control variables (Ethiopia & India)........ 18
Supplementary Table 15. Mental health outcomes by control variables (Peru & Vietnam) 19
Supplementary Table 16. Logistic regression results: Symptoms of at least mild anxiety/depression by gender (Ethiopia)........ 21
Supplementary Table 17. Logistic regression results: Symptoms of at least mild anxiety/depression by gender (India)........ 22
Supplementary Table 18. Logistic regression results: Symptoms of at least mild anxiety/depression by gender (Peru) 24
Supplementary Table 19. Logistic regression results: Symptoms of at least mild anxiety/depression by gender (Vietnam) 25
Supplementary Table 20. Logistic regression results: Symptoms of at least mild anxiety/depression by gender for the Younger Cohort only (Ethiopia)........ 28
Supplementary Table 21. Logistic regression results: Symptoms of at least mild anxiety/depression by gender for the Younger Cohort only (India)........ 29
Supplementary Table 22. Logistic regression results: Symptoms of at least mild anxiety/depression by gender for the Younger Cohort only (Peru)........ 31

For peer review only

Supplementary Table 23. Logistic regression results: Symptoms of at least mild anxiety/depression by gender for the Younger Cohort only (Vietnam) 32
Supplementary Table 24. Ethiopia: Mean score and rates of general anxiety disorder 34
Supplementary Table 25. Ethiopia: Mean score and rates of depression................. 35
Supplementary Table 26.India: Mean score and rates of general anxiety disorder .. 36
Supplementary Table 27. India: Mean score and rates of depression...................... 37
Supplementary Table 28. Peru: Mean score and rates of general anxiety disorder . 38
Supplementary Table 29. Peru: Mean score and rates of depression 39
Supplementary Table 30. Vietnam: Mean score and rates of general anxiety disorder 40
Supplementary Table 31. Vietnam: Mean score and rates of depression................. 41
Supplementary Table 32. Correlation between GAD-7 and PHQ-8 scores 42
Supplementary Table 33. Joint rates 42
Supplementary Table 34. Correlation between subjective well-being and GAD-7/PHQ-8 scores 43
Supplementary Table 35. Subjective well-being score Round 5 (2016) / phone survey (2020) by anxiety/depression (Ethiopia & India) 44
Supplementary Table 36. Subjective well-being score Round 5 (2016) / phone survey (2020) by anxiety/depression (Peru & Vietnam) 45

For peer review only

Supplementary Table 1. Sample size

	Ethiopia			India			Peru			Vietnam			Total		
	Full sample	YC	OC	Full sample	YC	OC	Full sample	YC	OC	Full sample	YC	OC	Full sample	YC	OC
Round 5, 2016	2626	1812	814	2822	1900	922	2468	1860	608	2848	1938	910	10764	7510	3254
Tracking samples, 2019-2020	2634	1803	831	2815	1902	913	2215	1701	514	2832	1922	910	10496	7328	3168
Phone survey, 2020	2439	1665	774	2754	1868	886	2018	1561	457	2519	1691	828	9730	6785	2945
Mental health measures available	2433	1659	774	2753	1867	886	1977	1535	442	2517	1690	827	9680	6751	2929
Included in our analysis	2183	1533	650	2622	1786	836	1887	1496	391	2296	1596	700	8988	6411	2577

Notes: YC stands for Younger Cohort, OC for Older Cohort.

Supplementary Table 2. Attrition rates (%)

	Ethiopia			India			Peru			Vietnam			Total		
	Full sample	YC	OC	Full sample	YC	OC	Full sample	YC	OC	Full sample	YC	OC	Full sample	YC	OC
Tracking sample to phone survey	7.4	7.7	6.9	2.2	1.8	3.0	8.9	8.2	11.1	11.1	12.0	9.0	7.3	7.4	7.0

For peer review only

Supplementary Table 3. Non-response rate to mental health questions (%)

Ethiopia			India			Peru			Vietnam			Total		
Full sample	YC	OC	Full sample	YC	OC	Full sample	YC	OC	Full sample	YC	OC	Full sample	YC	OC
0.2	0.4	0.0	0.0	0.1	0.0	2.0	1.7	3.3	0.1	0.1	0.1	0.5	0.5	0.5

Supplementary Table 4. Measures of reliability and internal consistency

		Cronbach's Alpha	Inter-item correlation	Kaiser–Meyer–Olkin measure of sampling adequacy
Ethiopia	GAD-7	0.728	0.277	0.792
	PHQ-8	0.652	0.190	0.744
India	GAD-7	0.691	0.242	0.777
	PHQ-8	0.637	0.180	0.744
Peru	GAD-7	0.804	0.369	0.872
	PHQ-8	0.773	0.299	0.839
Vietnam	GAD-7	0.749	0.299	0.814
	PHQ-8	0.748	0.271	0.807

Note: Results are for the combined Younger Cohort/ Older Cohort sample.

For peer review only

Supplementary Table 5. Sample description

	Ethiopia		India		Peru		Vietnam	
	Mean	sd	Mean	sd	Mean	sd	Mean	sd
COVID-19 related stressors								
Risk perception: believe they are at medium/high risk	69.31	0.461	46.38	0.499	54.27	0.498	21.39	0.410
Left house for any reason in the past 7 days	94.50	0.228	79.25	0.406	80.45	0.397	87.67	0.329
Difference in subjective well-being 2016-2020	0.96	2.068	0.49	1.949	0.52	2.066	-0.39	1.876
Change in responsibilities:								
Spend more time taking care of children	29.00	0.454	18.31	0.387	34.23	0.475	22.91	0.420
Spend more time on household chores	46.82	0.499	50.95	0.500	77.53	0.417	51.44	0.500
Spend more time working in the family business	12.96	0.336	4.12	0.199	15.00	0.357	10.89	0.312
Economic adversities								
Faced with new health expenses	10.12	0.302	55.95	0.497	29.57	0.456	6.53	
Did not experience adversity	20.71	0.405	1.72	0.130	2.60	0.159	42.25	0.494
Experienced adversity but did not reduce food consumption	65.83	0.474	92.68	0.261	90.94	0.287	41.94	0.494
Reduced food consumption as response to experienced adversity	13.47	0.341	5.61	0.230	6.47	0.246	15.81	0.365
Changes in employment status								
Did not work at all in the past 12 month OR worked during the pandemic but not before the pandemic (and is not working now)	46.17	0.499	41.38	0.493	24.06	0.428	23.48	0.424
Did not work before the pandemic, but is working now	6.83	0.252	16.40	0.370	12.35	0.329	6.75	0.251
Worked before the pandemic and is working now/has a job	38.71	0.487	37.57	0.484	46.74	0.499	56.36	0.496
Worked before the pandemic and is not working now/does not have a job	8.29	0.276	4.65	0.211	16.85	0.374	13.41	0.341
Educational disruption								
Never attended school or not enrolled in full-time education/not planning to enrol[a]	32.68	0.469	33.87	0.473	58.96	0.492	47.49	0.500
Enrolled in/Planning to enrol in full time education and not participating in learning activities[a]	58.71	0.493	36.28	0.481	1.87	0.136	12.72	0.333
Enrolled in/Planning to enrol in full time education and participating in learning activities[a]	8.61	0.281	29.84	0.458	39.17	0.488	39.79	0.490
Background characteristics								
Female	46.27	0.499	47.79	0.500	49.28	0.500	51.61	0.500
Urban	45.99	0.499	28.60	0.452	81.13	0.391	45.73	0.498
Child has long-term health problem, 2016 (Round 5)	8.80	0.283	11.48	0.319	12.67	0.333	14.20	0.349
Total parent-child relationship score, 2012/2016 (Round 4/5)	26.26	2.848	27.63	2.916	24.86	3.610	25.74	3.204

For peer review only

For peer review only - http://bmjopen.bmj.com/site/about/guidelines.xhtml

	Ethiopia		India		Peru		Vietnam	
	Mean	sd	Mean	sd	Mean	sd	Mean	sd
Total peer-child relationship score, 2016 (Round 5)	24.13	2.866	25.02	2.631	23.41	3.204	22.57	
Middle/Top wealth tercile R5, 2016 (Round 5)	67.11	0.470	66.44	0.472	69.00	0.463	66.03	2.257
Proxy baseline information								
Emotional problem scale (EPS) score, 2009 (Round 3)[b]	2.78	2.473	3.57	2.348	4.27	2.323	3.68	2.143
Subjective well-being, 2009 (Round 3)[b]	4.86	1.719	4.73	1.815	6.12	1.616	5.43	1.604
N	2183		2622		1887		2296	

Note: Mean values are reported. Self-reported risk belief coded the question "chance of getting infected with Coronavirus" into no/low risk, or medium/high risk. All time-variant variables are measured in 2020 unless otherwise specified. [a] refers to Younger Cohort (18-19) only, [b] refers to Older Cohort (25-26) only. Around 30% of the sample in Ethiopia, India and Vietnam are part of the Older Cohort. In Peru they make up 20% of the sample. The average age of the Younger Cohort in Ethiopia, India, Peru, Vietnam in years (months) is 18 (226), 18 (227), 18 (227), and 18 (227). The average age of the Older Cohort in Ethiopia, India, Peru, Vietnam in years (months) is 25 (309), 25 (310), 25 (311), and 25 (311).

For peer review only

Supplementary Table 6. Logistic regression results: Symptoms of at least mild anxiety (Younger Cohort only)

	Ethiopia		India		Peru		Vietnam	
	Odds Ratio	95% CI	Odds Ratio	95% CI	Odds Ratio	95% CI	Odds Ratio	95% CI
COVID-19 related changes/behaviours								
Risk perception: believe they are at medium/high risk	0.679** (0.12)	[0.487,0.947]	1.244 (0.21)	[0.898,1.724]	1.432*** (0.16)	[1.149,1.784]	1.031 (0.22)	[0.673,1.579]
Left house for any reason in the past 7 days	0.885 (0.27)	[0.482,1.627]	1.576* (0.41)	[0.948,2.620]	1.023 (0.14)	[0.784,1.335]	1.304 (0.34)	[0.783,2.171]
Difference in subjective well-being between Round 5 (2016) and phone survey (2020)	1.043 (0.04)	[0.969,1.123]	1.103** (0.05)	[1.007,1.207]	1.102*** (0.03)	[1.046,1.161]	1.195*** (0.06)	[1.084,1.316]
Change in responsibilities:								
Spend more time taking care of children	1.012 (0.20)	[0.692,1.479]	2.176*** (0.52)	[1.363,3.475]	1.509*** (0.18)	[1.194,1.906]	1.400 (0.31)	[0.913,2.147]
Spend more time on household chores	0.983 (0.17)	[0.694,1.392]	0.816 (0.15)	[0.570,1.169]	1.180 (0.17)	[0.895,1.557]	1.013 (0.18)	[0.718,1.430]
Spend more time working in the family business	0.703 (0.21)	[0.394,1.253]	2.117** (0.67)	[1.136,3.943]	1.235 (0.19)	[0.917,1.663]	1.640** (0.38)	[1.037,2.593]
Economic shocks								
Faced with new health expenses	0.668 (0.21)	[0.360,1.240]	0.881 (0.16)	[0.624,1.245]	1.741*** (0.21)	[1.371,2.209]	1.309 (0.42)	[0.699,2.455]
Experienced adversity but did not reduce food consumption	3.636*** (0.99)	[2.128,6.213]	0.702 (0.38)	[0.245,2.011]	2.503** (1.08)	[1.074,5.834]	1.493** (0.29)	[1.021,2.184]
Reduced food consumption as response to experienced adversity	8.158*** (2.58)	[4.393,15.149]	1.173 (0.73)	[0.345,3.985]	2.154 (1.03)	[0.844,5.498]	1.680** (0.42)	[1.027,2.748]
Educational disruption								
Enrolled in/Planning to enrol in full time education and not participating in learning activities	1.588** (0.33)	[1.052,2.396]	1.043 (0.21)	[0.707,1.538]	0.600 (0.27)	[0.245,1.471]	0.828 (0.25)	[0.458,1.495]
Enrolled in/Planning to enrol in full time education and participating in learning activities	1.110 (0.38)	[0.565,2.182]	0.710 (0.17)	[0.450,1.120]	1.026 (0.12)	[0.816,1.290]	0.699* (0.15)	[0.465,1.051]
Background characteristics								
Age in months	1.000 (0.02)	[0.960,1.042]	0.987 (0.02)	[0.946,1.030]	0.991 (0.01)	[0.963,1.020]	1.020 (0.03)	[0.967,1.077]
Female	1.216 (0.21)	[0.874,1.692]	1.420* (0.30)	[0.940,2.143]	1.758*** (0.20)	[1.402,2.205]	1.453** (0.25)	[1.034,2.041]
Urban	1.565** (0.31)	[1.059,2.312]	1.198 (0.24)	[0.806,1.781]	1.362* (0.22)	[0.986,1.882]	1.322 (0.26)	[0.901,1.940]
Participant has long-term health problem, 2016 (Round 5)	1.298 (0.36)	[0.755,2.233]	1.226 (0.30)	[0.754,1.995]	1.624*** (0.28)	[1.163,2.266]	1.030 (0.29)	[0.591,1.793]
Total parent-child relationship score, 2012/2016 (Round 4/5)	0.959 (0.03)	[0.902,1.019]	0.912*** (0.03)	[0.861,0.967]	0.975 (0.02)	[0.944,1.007]	1.030 (0.03)	[0.976,1.088]

For peer review only

	Ethiopia		India		Peru		Vietnam	
	Odds Ratio	95% CI	Odds Ratio	95% CI	Odds Ratio	95% CI	Odds Ratio	95% CI
Total peer-child relationship score, 2016 (Round 5)	0.951 (0.03)	[0.895,1.011]	1.015 (0.04)	[0.948,1.088]	0.999 (0.02)	[0.964,1.035]	0.968 (0.04)	[0.896,1.047]
Middle/Top wealth tercile R5, 2016 (Round 5)	0.908 (0.19)	[0.608,1.356]	0.712* (0.13)	[0.495,1.023]	1.105 (0.15)	[0.845,1.444]	0.677** (0.13)	[0.467,0.981]
N	1533		1786		1496		1596	

*Note: Odds ratios are unadjusted odds ratios. Robust standard errors in parenthesis, *** significant at 1%, ** significant at 5%, * significant at 10%. Base categories are as follows: Believe they are at no/low risk, Did not leave the house at all during the past 7 days, Did not spend more time taking care of children, Did not spend more time on household chores, Did not spend more time working in the family business, Did not face new health expenses, Did not suffer a shock, Never attended school or not enrolled in full-time education/not planning to enrol, Male, Rural, Does not have long-term health condition, Lowest wealth tercile. All time-variant variables are measured in 2020 unless otherwise specified.*

Supplementary Table 7. Logistic regression results: Symptoms of at least mild depression (Younger Cohort only)

	Ethiopia		India		Peru		Vietnam	
	Odds Ratio	95% CI	Odds Ratio	95% CI	Odds Ratio	95% CI	Odds Ratio	95% CI
<u>*COVID-19 related changes/behaviours*</u>								
Risk perception: believe they are at medium/high risk	0.498*** (0.08)	[0.361,0.687]	0.966 (0.16)	[0.693,1.346]	1.548*** (0.18)	[1.230,1.949]	1.025 (0.22)	[0.674,1.559]
Left house for any reason in the past 7 days	0.940 (0.28)	[0.520,1.702]	2.537*** (0.77)	[1.403,4.589]	0.928 (0.13)	[0.704,1.223]	1.163 (0.28)	[0.724,1.867]
Difference in subjective well-being between Round 5 (2016) and phone survey (2020)	1.049 (0.04)	[0.973,1.130]	1.102** (0.05)	[1.011,1.201]	1.079*** (0.03)	[1.020,1.142]	1.169*** (0.06)	[1.065,1.284]
Change in responsibilities:								
Spend more time taking care of children	1.061 (0.20)	[0.736,1.527]	2.170*** (0.51)	[1.370,3.438]	1.268* (0.16)	[0.992,1.621]	1.616** (0.33)	[1.087,2.403]
Spend more time on household chores	1.082 (0.19)	[0.763,1.533]	0.857 (0.16)	[0.592,1.241]	1.142 (0.17)	[0.858,1.520]	1.840*** (0.34)	[1.287,2.631]
Spend more time working in the family business	1.076 (0.29)	[0.636,1.823]	0.803 (0.34)	[0.354,1.823]	1.160 (0.18)	[0.850,1.582]	1.724** (0.38)	[1.121,2.652]
Economic shocks								
Faced with new health expenses	0.222*** (0.10)	[0.093,0.530]	1.062 (0.19)	[0.747,1.510]	1.851*** (0.23)	[1.450,2.363]	0.805 (0.28)	[0.410,1.580]
Experienced adversity but did not reduce food consumption	4.913*** (1.49)	[2.712,8.902]	0.651 (0.36)	[0.218,1.944]	2.541* (1.21)	[0.997,6.476]	1.763*** (0.36)	[1.181,2.631]
Reduced food consumption as response to experienced	12.771*** (4.42)	[6.484,25.156]	0.913 (0.61)	[0.248,3.357]	3.620** (1.89)	[1.300,10.079]	2.292*** (0.57)	[1.410,3.726]

For peer review only

	Ethiopia		India		Peru		Vietnam	
	Odds Ratio	95% CI	Odds Ratio	95% CI	Odds Ratio	95% CI	Odds Ratio	95% CI
adversity								
Educational disruption								
Enrolled in/Planning to enrol in full time education and not participating in learning activities	1.728*** (0.36)	[1.153,2.592]	1.103 (0.22)	[0.743,1.638]	0.743 (0.36)	[0.287,1.928]	0.944 (0.28)	[0.529,1.685]
Enrolled in/Planning to enrol in full time education and participating in learning activities	1.174 (0.41)	[0.591,2.333]	0.749 (0.18)	[0.469,1.196]	1.042 (0.13)	[0.816,1.329]	1.172 (0.25)	[0.768,1.788]
Background characteristics								
Age in months	0.999 (0.02)	[0.959,1.041]	0.997 (0.02)	[0.954,1.041]	0.974* (0.01)	[0.945,1.003]	1.028 (0.03)	[0.975,1.084]
Female	1.318 (0.23)	[0.940,1.848]	1.225 (0.25)	[0.821,1.828]	1.282** (0.16)	[1.009,1.630]	1.438** (0.24)	[1.032,2.004]
Urban	1.533** (0.29)	[1.051,2.235]	0.969 (0.20)	[0.644,1.457]	1.175 (0.20)	[0.840,1.643]	1.093 (0.22)	[0.736,1.623]
Participant has long-term health problem, 2016 (Round 5)	0.540* (0.20)	[0.265,1.098]	1.461 (0.35)	[0.909,2.346]	1.502** (0.26)	[1.077,2.095]	1.466 (0.37)	[0.889,2.415]
Total parent-child relationship score, 2012/2016 (Round 4/5)	1.011 (0.03)	[0.954,1.072]	0.890*** (0.03)	[0.838,0.945]	0.924*** (0.02)	[0.892,0.957]	0.971 (0.03)	[0.918,1.026]
Total peer-child relationship score, 2016 (Round 5)	0.902*** (0.03)	[0.848,0.960]	1.000 (0.04)	[0.930,1.076]	1.025 (0.02)	[0.987,1.065]	0.987 (0.04)	[0.910,1.071]
Middle/Top wealth tercile R5, 2016 (Round 5)	0.763 (0.15)	[0.518,1.122]	1.275 (0.24)	[0.879,1.851]	1.166 (0.17)	[0.878,1.550]	1.189 (0.24)	[0.797,1.773]
N	1533		1786		1496		1596	

*Note: Odds ratios are unadjusted odds ratios. Robust standard errors in parenthesis, *** significant at 1%, ** significant at 5%, * significant at 10%. Base categories are as follows: Believe they are at no/low risk, Did not leave the house at all during the past 7 days, Did not spend more time taking care of children, Did not spend more time on household chores, Did not spend more time working in the family business, Did not face new health expenses, Did not suffer a shock, Never attended school or not enrolled in full-time education/not planning to enrol, Male, Rural, Does not have long-term health condition, Lowest wealth tercile. All time-variant variables are measured in 2020 unless otherwise specified.*

For peer review only

Supplementary Table 8. Logistic regression results: Symptoms of at least mild anxiety (Older Cohort only)

	Ethiopia		India		Peru		Vietnam	
	**Odds Ratio**	**95% CI**	**Odds Ratio**	**95% CI**	**Odds Ratio**	**95% CI**	**Odds Ratio**	**95% CI**
COVID-19 related changes/behaviours								
Risk perception: believe they are at medium/high risk	1.466 (0.35)	[0.915,2.346]	1.383 (0.30)	[0.908,2.108]	1.693** (0.41)	[1.051,2.728]	1.060 (0.37)	[0.536,2.098]
Left house for any reason in the past 7 days	0.693 (0.33)	[0.274,1.751]	1.257 (0.38)	[0.697,2.267]	0.850 (0.31)	[0.414,1.746]	0.432* 0.21)	[0.169,1.109]
Difference in subjective well-being between Round 5 (2016) and phone survey (2020)	1.089* (0.05)	[0.987,1.201]	1.121* (0.07)	[0.983,1.278]	1.132* (0.08)	[0.990,1.294]	1.105 (0.12)	[0.901,1.356]
Change in responsibilities:								
Spend more time taking care of children	0.735 (0.21)	[0.420,1.285]	2.081***(0.52)	[1.271,3.405]	0.971 (0.25)	[0.590,1.599]	1.230 (0.46)	[0.595,2.544]
Spend more time on household chores	1.313 (0.38)	[0.742,2.325]	0.639* (0.16)	[0.394,1.034]	1.667 (0.53)	[0.893,3.114]	2.240**(0.87)	[1.043,4.808]
Spend more time working in the family business	1.275 (0.41)	[0.682,2.383]	0.498 (0.41)	[0.100,2.478]	1.599 (0.54)	[0.829,3.085]	2.126* (0.96)	[0.879,5.139]
Economic shocks								
Faced with new health expenses	1.523 (0.45)	[0.854,2.716]	1.435 (0.34)	[0.901,2.286]	1.572* (0.38)	[0.984,2.513]	0.808 (0.41)	[0.302,2.164]
Experienced adversity but did not reduce food consumption	0.908 (0.31)	[0.466,1.767]	0.560 (0.49)	[0.102,3.068]	1.312 (1.77)	[0.093,18.595]	1.988* (0.78)	[0.922,4.284]
Reduced food consumption as response to experienced adversity	4.323***(1.81)	[1.900,9.835]	0.567 (0.55)	[0.086,3.751]	2.691 (3.82)	[0.167,43.423]	1.986 (0.96)	[0.772,5.113]
Changes in employment status								
Did not work before the pandemic, but is working now	2.044 (0.97)	[0.809,5.165]	0.951 (0.47)	[0.358,2.525]	1.095 (0.62)	[0.363,3.303]	0.868 (0.78)	[0.149,5.071]
Worked before the pandemic and is working now/has a job	1.566* (0.39)	[0.956,2.567]	1.306 (0.36)	[0.756,2.255]	1.401 (0.56)	[0.638,3.075]	0.591 (0.34)	[0.194,1.796]
Worked before the pandemic and is not working now/does not have a job	2.787***(0.85)	[1.534,5.063]	2.312** (0.95)	[1.037,5.156]	5.434***(2.55)	[2.167,13.626]	1.693 (1.03)	[0.513,5.582]
Background characteristics								
Age in months	1.008 (0.03)	[0.956,1.063]	1.052* (0.03)	[0.999,1.108]	0.977 (0.03)	[0.923,1.034]	0.960 (0.04)	[0.879,1.047]
Female	0.754 (0.21)	[0.432,1.319]	1.954** (0.58)	[1.087,3.511]	1.125 (0.30)	[0.670,1.888]	0.790 (0.27)	[0.404,1.545]

For peer review only

	Ethiopia		India		Peru		Vietnam	
	Odds Ratio	95% CI	Odds Ratio	95% CI	Odds Ratio	95% CI	Odds Ratio	95% CI
Urban	1.087 (0.23)	[0.713,1.658]	0.561** (0.15)	[0.334,0.942]	1.825 (0.75)	[0.819,4.065]	0.961 (0.31)	[0.516,1.790]
Participant has long-term health problem, 2016 (Round 5)	1.134 (0.33)	[0.637,2.019]	1.583* (0.40)	[0.960,2.612]	2.591***(0.91)	[1.298,5.171]	1.647 (0.54)	[0.862,3.150]
Total parent-child relationship score, 2012/2016 (Round 4/5)	0.957 (0.03)	[0.899,1.018]	0.939* (0.03)	[0.875,1.007]	0.997 (0.03)	[0.940,1.058]	1.118* (0.07)	[0.989,1.265]
Total peer-child relationship score, 2016 (Round 5)	0.948 (0.03)	[0.887,1.014]	1.041 (0.04)	[0.963,1.124]	1.011 (0.04)	[0.938,1.089]	0.858* (0.08)	[0.720,1.023]
Middle/Top wealth tercile R5, 2016 (Round 5)	1.033 (0.23)	[0.670,1.594]	0.871 (0.20)	[0.551,1.376]	0.635 (0.20)	[0.342,1.180]	0.488** 0.16)	[0.260,0.914]
Proxy baseline information								
Emotional problem scale (EPS) score, 2009 (Round 3)	1.074* (0.04)	[0.994,1.161]	1.007 (0.05)	[0.910,1.114]	1.222*** 0.07)	[1.093,1.366]	1.072 (0.07)	[0.940,1.221]
Subjective well-being, 2009 (Round 3)	0.979 (0.06)	[0.872,1.100]	1.131** (0.07)	[1.010,1.267]	0.924 (0.07)	[0.800,1.068]	0.975 (0.08)	[0.827,1.148]
N	650		836		391		700	

*Note: Odds ratios are unadjusted odds ratios. Robust standard errors in parenthesis, *** significant at 1%, ** significant at 5%, * significant at 10%. Base categories are as follows: Believe they are at no/low risk, Did not leave the house at all during the past 7 days, Did not spend more time taking care of children, Did not spend more time on household chores, Did not spend more time working in the family business, Did not face new health expenses, Did not suffer a shock, Did not work at all in the past 12 month OR worked during the pandemic but not before and is not working now, Male, Rural, Does not have long-term health condition, Lowest wealth tercile. All time-variant variables are measured in 2020 unless otherwise specified.*

Supplementary Table 9. Logistic regression results: Symptoms of at least mild depression (Older Cohort only)

Depression	OC	Ethiopia		India		Peru		Vietnam	
		Odds Ratio	95% CI	Odds Ratio	95% CI	Odds Ratio	95% CI	Odds Ratio	95% CI
COVID-19 related changes/behaviours									
	Risk perception: believe they are at medium/high risk	1.595* (0.43)	[0.942,2.703]	0.956 (0.22)	[0.607,1.505]	1.610* 0.41)	[0.980,2.646]	1.672 (0.58)	[0.851,3.287]
	Left house for any reason in the past 7 days	1.216 (0.69)	[0.402,3.672]	1.780 (0.63)	[0.890,3.559]	1.778 (0.78)	[0.753,4.195]	0.287***(0.14)	[0.113,0.731]
	Difference subjective well-being between Round 5 (2016) and	1.221***(0.08)	[1.082,1.377]	1.175***(0.07)	[1.043,1.324]	1.076 (0.08)	[0.926,1.250]	1.129 (0.12)	[0.915,1.393]

For peer review only

Depression	OC	Ethiopia		India		Peru		Vietnam	
		**Odds Ratio**	**95% CI**	**Odds Ratio**	**95% CI**	**Odds Ratio**	**95% CI**	**Odds Ratio**	**95% CI**
	phone survey (2020)								
Change in responsibilities:									
	Spend more time taking care of children	0.517** (0.16)	[0.279,0.959]	1.135 (0.33)	[0.637,2.023]	0.707 (0.20)	[0.401,1.247]	1.252 (0.50)	[0.571,2.746]
	Spend more time on household chores	1.457 (0.54)	[0.708,3.002]	0.858 (0.24)	[0.499,1.476]	1.026 (0.33)	[0.543,1.940]	0.912 (0.33)	[0.448,1.858]
	Spend more time working in the family business	1.215 (0.42)	[0.614,2.407]	1.258 (0.73)	[0.402,3.938]	1.534 (0.53)	[0.779,3.022]	1.888 (0.87)	[0.763,4.673]
Economic shocks									
	Faced with new health expenses	1.278 (0.43)	[0.657,2.487]	1.821** (0.48)	[1.089,3.045]	1.226 (0.31)	[0.745,2.017]	0.681 (0.39)	[0.223,2.079]
	Experienced adversity but did not reduce food consumption	1.566 (0.69)	[0.661,3.713]	1.027 (1.15)	[0.114,9.237]	0.463 (0.57)	[0.042,5.082]	1.513 (0.55)	[0.744,3.074]
	Reduced food consumption as response to experienced adversity	4.926***(2.50)	[1.825,13.296]	0.520 (0.66)	[0.042,6.360]	0.363 (0.48)	[0.027,4.887]	1.005 (0.51)	[0.374,2.699]
Changes in employment status									
	Did not work before the pandemic, but is working now[3]	3.429***(1.59)	[1.382,8.507]	0.270* (0.20)	[0.061,1.185]	1.044 (0.69)	[0.285,3.829]	1.000 (.)	[1.000,1.000]
	Worked before the pandemic and is working now/has a job	1.588 (0.45)	[0.915,2.758]	0.740 (0.21)	[0.422,1.297]	0.943 (0.43)	[0.387,2.299]	0.634 (0.37)	[0.203,1.978]
	Worked before the pandemic and is not working now/does not have a job	2.360** (0.87)	[1.145,4.865]	1.267 (0.55)	[0.541,2.968]	1.986 (1.00)	[0.741,5.323]	1.747 (1.07)	[0.523,5.832]
<u>*Background characteristics*</u>									
	Age in months	1.018 (0.03)	[0.962,1.077]	1.065**(0.03)	[1.006,1.127]	0.984 (0.03)	[0.927,1.044]	0.985 (0.04)	[0.915,1.060]
	Female	0.834 (0.30)	[0.413,1.683]	1.301 (0.41)	[0.705,2.403]	1.266 (0.35)	[0.734,2.185]	1.093 (0.37)	[0.567,2.107]
	Urban	1.395 (0.36)	[0.846,2.298]	0.549**(0.16)	[0.313,0.961]	1.162 (0.50)	[0.496,2.722]	0.512**(0.17)	[0.266,0.988]
	Participant has long-term health problem, 2016 (Round 5)	1.129 (0.40)	[0.562,2.268]	1.636* (0.46)	[0.941,2.844]	2.246**(0.74)	[1.176,4.291]	1.036 (0.35)	[0.530,2.025]
	Total parent-child relationship score, 2012/2016 (Round 4/5)	1.003 (0.04)	[0.934,1.078]	0.953 (0.04)	[0.877,1.035]	1.010 (0.03)	[0.944,1.080]	1.059 (0.06)	[0.950,1.180]

For peer review only

Depression	OC	Ethiopia		India		Peru		Vietnam	
		Odds Ratio	95% CI	Odds Ratio	95% CI	Odds Ratio	95% CI	Odds Ratio	95% CI
	Total peer-child relationship score, 2016 (Round 5)	1.049 (0.04)	[0.971,1.133]	1.041 (0.04)	[0.963,1.125]	0.943 (0.04)	[0.868,1.026]	1.001 (0.08)	[0.852,1.177]
	Middle/Top wealth tercile R5, 2016 (Round 5)	0.732 (0.19)	[0.445,1.205]	0.867 (0.22)	[0.522,1.440]	2.247**(0.77)	[1.144,4.414]	0.840 (0.27)	[0.445,1.585]
Proxy baseline information									
	Emotional problem scale (EPS) score, 2009 (Round 3)	1.090* (0.05)	[0.995,1.195]	1.101* (0.06)	[0.991,1.223]	1.129**(0.07)	[1.003,1.270]	1.114 (0.08)	[0.973,1.276]
	Subjective well-being, 2009 (Round 3)	0.894 (0.06)	[0.782,1.023]	1.077 (0.07)	[0.952,1.219]	0.869* (0.07)	[0.736,1.026]	0.952 (0.08)	[0.805,1.126]
	N	650		836		391		683	

*Note: Odds ratios are unadjusted odds ratios. Robust standard errors in parenthesis, *** significant at 1%, ** significant at 5%, * significant at 10%. Base categories are as follows: Believe they are at no/low risk, Did not leave the house at all during the past 7 days, Did not spend more time taking care of children, Did not spend more time on household chores, Did not spend more time working in the family business, Did not face new health expenses, Did not suffer a shock, Did not work at all in the past 12 month OR worked during the pandemic but not before and is not working now, Male, Rural, Does not have long-term health condition, Lowest wealth tercile. All time-variant variables are measured in 2020 unless otherwise specified. [3]In Vietnam only 17 people did not work before the pandemic but are working now. None showed depressive symptoms. As it predicts failure perfectly it drops out of the regressions.*

For peer review only

Supplementary Table 10. Ethiopia: Means of continuous control variables by at least mild anxiety/depression

		All	at least mild anxiety	no anxiety	p-value	at least mild depression	no depression	p-value
COVID-19 related changes/behaviours								
	Difference in subjective well-being between Round 5 (2016) and phone survey (2020)	0.96	1.15	0.92	0.052	1.26	0.91	0.005
Background characteristics								
	Age in months	250.86	264.14	247.97	0.000	255.55	250.00	0.014
	Total parent-child relationship score, 2012/2016 (Round 4/5)	26.26	25.98	26.32	0.036	26.20	26.27	0.686
	Total peer-child relationship score, 2016 (Round 5)	24.13	23.51	24.26	0.000	23.58	24.23	0.000
Proxy baseline information								
	Emotional problem scale (EPS) score, 2009 (Round 3)²	2.78	3.00	2.70	0.173	3.13	2.71	0.096
	Subjective well-being, 2009 (Round 3)²	5.22	5.18	5.23	0.649	5.24	5.22	0.896

Note: *Results are for the combined Younger Cohort/ Older Cohort sample unless indicated otherwise, ² refers to Older Cohort (25-26) only.*

For peer review only

Supplementary Table 11. India: Means of continuous control variables by at least mild anxiety/depression

		All	at least mild anxiety	no anxiety	p-value	at least mild depression	no depression	p-value
COVID-19 related changes/behaviours								
	Difference in subjective well-being between Round 5 (2016) and phone survey (2020)	0.49	0.77	0.45	0.009	0.86	0.45	0.001
Background characteristics								
	Age in months	253.30	261.16	252.32	0.000	258.12	252.77	0.037
	Total parent-child relationship score, 2012/2016 (Round 4/5)	27.63	26.94	27.72	0.000	26.84	27.72	0.000
	Total peer-child relationship score, 2016 (Round 5)	25.02	24.97	25.03	0.743	24.91	25.03	0.468
Proxy baseline information								
	Emotional problem scale (EPS) score, 2009 (Round 3)²	3.57	3.93	3.51	0.067	4.14	3.49	0.010
	Subjective well-being, 2009 (Round 3)²	5.00	5.29	4.96	0.013	4.97	5.00	0.850

Note: *Results are for the combined Younger Cohort/ Older Cohort sample unless indicated otherwise, ² refers to Older Cohort (25-26) only.*

For peer review only

Supplementary Table 12. Peru: Means of continuous control variables by at least mild anxiety/depression

		All	at least mild anxiety	no anxiety	p-value	at least mild depression	no depression	p-value
COVID-19 related changes/behaviours								
	Difference in subjective well-being in Round 5 (2016) and phone survey (2020)	0.52	0.74	0.38	0.000	0.70	0.44	0.013
Background characteristics								
	Age in months	244.23	245.19	243.57	0.315	242.56	245.00	0.151
	Total parent-child relationship score, 2012/2016 (Round 4/5)	24.86	24.60	25.04	0.009	24.28	25.12	0.000
	Total peer-child relationship score, 2016 (Round 5)	23.41	23.40	23.42	0.862	23.41	23.42	0.954
Proxy baseline information								
	Emotional problem scale (EPS) score, 2009 (Round 3)²	4.27	4.87	3.81	0.000	4.85	4.03	0.002
	Subjective well-being, 2009 (Round 3)²	6.82	6.72	6.89	0.073	6.72	6.87	0.135

Note: Results are for the combined Younger Cohort/ Older Cohort sample unless indicated otherwise, ² refers to Older Cohort (25-26) only.

For peer review only

Supplementary Table 13. Vietnam: Means of continuous control variables by at least mild anxiety/depression

		All	at least mild anxiety	no anxiety	p-value	at least mild depression	no depression	p-value
COVID-19 related changes/behaviours								
	Difference in subjective well-being in Round 5 (2016) and phone survey (2020)	-0.39	0.14	-0.45	0.000	0.11	-0.45	0.000
Background characteristics								
	Age in months	252.95	248.28	253.43	0.067	246.49	253.63	0.010
	Total parent-child relationship score, 2012/2016 (Round 4/5)	25.74	26.11	25.70	0.073	25.72	25.74	0.930
	Total peer-child relationship score, 2016 (Round 5)	22.57	22.40	22.59	0.230	22.52	22.58	0.704
Proxy baseline information								
	Emotional problem scale (EPS) score, 2009 (Round 3)²	3.68	3.96	3.65	0.314	4.18	3.64	0.086
	Subjective well-being, 2009 (Round 3)²	5.84	5.73	5.86	0.383	5.85	5.84	0.943

Note: *Results are for the combined Younger Cohort/ Older Cohort sample unless indicated otherwise, ² refers to Older Cohort (25-26) only.*

For peer review only

Supplementary Table 14. Mental health outcomes by control variables (Ethiopia & India)

	Ethiopia				India			
	% at least mild Anxiety	p-value	% at least mild Depression	p-value	% at least mild Anxiety	p-value	% at least mild Depression	p-value
COVID-19 related changes/behaviours								
Risk perception: believe they are at no/low risk	20.30	0.048	20.75	0.000	10.10	0.092	10.10	0.735
Risk perception: believe they are at medium/high risk	16.79		13.09		12.17		9.70	
Did not leave house for any reason in the past 7 days	22.50	0.173	18.33	0.367	8.46	0.030	5.51	0.000
Left house for any reason in the past 7 days	17.60		15.27		11.74		11.07	
Change in responsibilities:								
Did not spend more time taking care of children	18.06	0.704	15.55	0.823	9.01	0.000	8.82	0.000
Spend more time taking care of children	17.38		15.17		20.21		14.79	
Did not spend more time on household chores	17.83	0.963	14.64	0.273	10.58	0.438	9.72	0.742
Spend more time on household chores	17.91		16.34		11.53		10.10	
Did not spend more time working in the family business	18.26	0.209	15.63	0.516	10.90	0.204	9.90	0.924
Spend more time working in the family business	15.19		14.13		14.81		10.19	
Economic shocks								
Was not faced with new health expenses	17.43	0.115	15.75	0.230	10.91	0.827	8.57	0.041
Faced with new health expenses	21.72		12.67		11.18		10.97	
Did not experience adversity	6.86	0.000	4.65	0.000	13.33	0.381	11.11	0.956
Experienced adversity but did not reduce food consumption	17.54		15.59		10.82		9.88	
Reduced food consumption as response to experienced adversity	36.39		31.29		14.29		10.20	
Changes in employment status								
Did not work at all in the past 12 month OR worked during the pandemic but not before the pandemic (and is not working now)	13.79	0.000	13.10	0.000	9.22	0.000	9.03	0.059
Did not work before the pandemic, but is working now	26.17		25.50		9.07		8.37	
Worked before the pandemic and is working now/has a job	18.34		14.91		12.89		10.86	
Worked before the pandemic and is not working now/does not have a job	31.49		22.65		19.67		15.57	
Educational disruption								
Never attended school or not enrolled in full-time education/not planning to enrol in learning activities[1]	18.25	0.212	13.45	0.056	13.01	0.000	11.00	0.012
Enrolled in/Planning to enrol in full time education and	16.58		17.41		10.69		10.40	

For peer review only

not participating in learning activities[1]								
Enrolled in/Planning to enrol in full time education and participating in learning activities[1]	21.13		15.47		6.57		6.57	
Background characteristics								
Participant does not have a long-term health problem, 2016 (Round 5)	17.13	0.004	15.42	0.940	10.47	0.007	9.31	0.004
Participant has long-term health problem, 2016 (Round 5)	25.52		15.63		15.61		14.62	

Notes: If any missing answers to questions then the whole score is set to missing. p-value indicates significance of t-tests between dummy variables and o F-tests in case of categorical variables. Results are for the combined Younger Cohort/ Older Cohort sample unless indicated otherwise, [1] refers to Younger Cohort (18-19) only.

For peer review only

Supplementary Table 15. Mental health outcomes by control variables (Peru & Vietnam)

	Peru				Vietnam			
	**% at least mild Anxiety**	p-value	**% at least mild Depression**	p-value	**% at least mild Anxiety**	p-value	**% at least mild Depression**	p-value
COVID-19 related changes/behaviours								
Risk perception: believe they are at no/low risk	35.34	0.000	25.84	0.000	9.31	0.967	9.31	0.557
Risk perception: believe they are at medium/high risk	45.51		36.43		9.37		10.18	
Did not leave house for any reason in the past 7 days	42.82	0.393	33.33	0.421	9.54	0.892	11.66	0.184
Left house for any reason in the past 7 days	40.38		31.16		9.29		9.19	
Change in responsibilities:								
Did not spend more time taking care of children	36.83	0.000	29.57	0.009	8.19	0.001	8.19	0.000
Spend more time taking care of children	48.61		35.45		13.12		13.88	
Did not spend more time on household chores	33.96	0.001	28.77	0.157	7.44	0.003	6.46	0.000
Spend more time on household chores	42.86		32.40		11.09		12.36	
Did not spend more time working in the family business	40.09	0.105	30.92	0.141	8.46	0.000	8.60	0.000
Spend more time working in the family business	45.23		35.34		16.40		16.80	
Economic shocks								
Was not faced with new health expenses	36.12	0.000	27.24	0.000	8.99	0.041	9.27	0.171
Faced with new health expenses	52.15		41.94		14.00		12.67	

	Peru				Vietnam			
	**% at least mild Anxiety**	p-value	**% at least mild Depression**	p-value	**% at least mild Anxiety**	p-value	**% at least mild Depression**	p-value
Did not experience adversity	16.33	0.002	14.29	0.004	6.49	0.000	6.29	0.000
Experienced adversity but did not reduce food consumption	41.32		31.47		10.80		11.11	
Reduced food consumption as response to experienced adversity	44.26		40.16		12.95		13.77	
Changes in employment status								
Did not work at all in the past 12 month OR worked during the pandemic but not before the pandemic (and is not working now)	41.41	0.176	33.26	0.003	8.53	0.269	9.83	0.002
Did not work before the pandemic, but is working now	37.77		32.19		10.97		8.39	
Worked before the pandemic and is working now/has a job	39.57		27.89		8.81		8.11	
Worked before the pandemic and is not working now/does not have a job	45.91		38.99		12.01		15.26	
Educational disruption								
Never attended school or not enrolled in full-time education/not planning to enrol in learning activities[1]	40.74	0.611	30.74	0.490	9.95	0.374	8.91	0.387
Enrolled in/Planning to enrol in full time education and not participating in learning activities[1]	32.14		28.57		8.96		9.43	
Enrolled in/Planning to enrol in full time education and participating in learning activities[1]	41.47		33.33		8.04		10.82	
Background characteristics								
Participant does not have a long-term health problem, 2016 (Round 5)	38.71	0.000	29.85	0.000	9.04	0.248	9.14	0.151
Participant has long-term health problem, 2016 (Round 5)	55.65		43.51		11.04		11.66	

Notes: If any missing answers to questions then the whole score is set to missing. p-value indicates significance of t-tests between dummy variables and o F-tests in case of categorical variables. Results are for the combined Younger Cohort/ Older Cohort sample unless indicated otherwise, [1] refers to Younger Cohort (18-19) only.

For peer review only

Supplementary Table 16. Logistic regression results: Symptoms of at least mild anxiety/depression by gender (Ethiopia)

	At least mild anxiety				At least mild depression			
	Male		Female		Male		Female	
	Odds Ratio	95% CI	Odds Ratio	95% CI	Odds Ratio	95% CI	Odds Ratio	95% CI
COVID-19 related changes/behaviours								
Risk perception: believe they are at medium/high risk	0.807 (0.14)	[0.569,1.146]	0.825 (0.16)	[0.562,1.211]	0.576*** (0.10)	[0.403,0.824]	0.659** (0.13)	[0.452,0.961]
Left house for any reason in the past 7 days	0.593 (0.29)	[0.229,1.533]	0.847 (0.25)	[0.480,1.494]	0.556 (0.25)	[0.233,1.327]	1.126 (0.35)	[0.614,2.064]
Difference in subjective well-being between Round 5 (2016) and phone survey (2020)	1.097** (0.04)	[1.016,1.185]	1.052 (0.05)	[0.967,1.146]	1.084* (0.05)	[0.999,1.177]	1.114** (0.05)	[1.019,1.218]
Change in responsibilities:								
Spend more time taking care of children	1.262 (0.34)	[0.747,2.132]	0.749 (0.14)	[0.513,1.093]	0.791 (0.21)	[0.468,1.339]	0.957 (0.20)	[0.640,1.433]
Spend more time on household chores	0.869 (0.20)	[0.558,1.353]	1.447 (0.33)	[0.929,2.252]	1.181 (0.25)	[0.776,1.797]	1.162 (0.27)	[0.731,1.845]
Spend more time working in the family business	0.750 (0.20)	[0.448,1.256]	0.713 (0.24)	[0.367,1.384]	0.643 (0.19)	[0.361,1.144]	1.498 (0.47)	[0.814,2.755]
Economic shocks								
Faced with new health expenses	0.733 (0.21)	[0.419,1.281]	1.115 (0.30)	[0.660,1.886]	0.438*** (0.13)	[0.241,0.797]	0.592* (0.19)	[0.319,1.096]
Experienced adversity but did not reduce food consumption	1.889** (0.53)	[1.091,3.270]	3.063*** (1.00)	[1.619,5.793]	3.658*** (1.35)	[1.776,7.537]	3.739*** (1.28)	[1.908,7.326]
Reduced food consumption as response to experienced adversity	5.891*** (1.92)	[3.107,11.172]	8.642*** (3.16)	[4.222,17.690]	12.987*** (5.25)	[5.884,28.663]	8.585*** (3.32)	[4.026,18.308]
Changes in employment status								
Did not work before the pandemic, but is working now	2.623*** (0.88)	[1.356,5.075]	2.853*** (0.97)	[1.470,5.538]	2.426*** (0.79)	[1.283,4.591]	2.754*** (0.99)	[1.357,5.590]
Worked before the pandemic and is working now/has a job	1.335 (0.30)	[0.859,2.074]	1.981*** (0.46)	[1.260,3.115]	1.057 (0.23)	[0.684,1.634]	1.744** (0.41)	[1.096,2.776]
Worked before the pandemic and is not working now/does not have a job	2.196*** (0.61)	[1.268,3.801]	2.239*** (0.64)	[1.279,3.918]	1.020 (0.33)	[0.540,1.927]	2.647*** (0.79)	[1.476,4.746]
Background characteristics								
Age in months	1.012*** (0.00)	[1.007,1.016]	1.003 (0.00)	[0.999,1.008]	1.007*** (0.00)	[1.002,1.012]	0.996* (0.00)	[0.991,1.001]

For peer review only

	At least mild anxiety				At least mild depression			
	Male		Female		Male		Female	
	Odds Ratio	95% CI	Odds Ratio	95% CI	Odds Ratio	95% CI	Odds Ratio	95% CI
Urban	1.155<br>(0.22)	[0.799,1.670]	1.523**<br>(0.32)	[1.003,2.312]	1.307<br>(0.26)	[0.880,1.942]	1.440*<br>(0.31)	[0.938,2.210]
Participant has long-term health problem, 2016 (Round 5)	1.848**<br>(0.53)	[1.054,3.241]	1.204<br>(0.29)	[0.745,1.944]	0.919<br>(0.33)	[0.450,1.875]	0.998<br>(0.28)	[0.580,1.716]
Total parent-child relationship score, 2012/2016 (Round 4/5)	0.968<br>(0.03)	[0.914,1.026]	0.962<br>(0.03)	[0.902,1.026]	1.027<br>(0.03)	[0.965,1.092]	0.972<br>(0.03)	[0.908,1.039]
Total peer-child relationship score, 2016 (Round 5)	0.935**<br>(0.03)	[0.877,0.998]	0.944*<br>(0.03)	[0.890,1.002]	0.948<br>(0.03)	[0.884,1.017]	0.932**<br>(0.03)	[0.875,0.993]
Middle/Top wealth tercile R5, 2016 (Round 5)	1.199<br>(0.24)	[0.811,1.773]	0.985<br>(0.21)	[0.643,1.511]	0.882<br>(0.18)	[0.587,1.327]	0.844<br>(0.19)	[0.539,1.321]
N	1173		1010		1173		1010	

*Note: Odds ratios are unadjusted odds ratios. Robust standard errors in parenthesis, *** significant at 1%, ** significant at 5%, * significant at 10%. Base categories are as follows: Believe they are at no/low risk, Did not leave the house at all during the past 7 days, Did not spend more time taking care of children, Did not spend more time on household chores, Did not spend more time working in the family business, Did not face new health expenses, Did not suffer a shock, Did not work at all in the past 12 month OR worked during the pandemic but not before and is not working now, Rural, Does not have long-term health condition, Lowest wealth tercile. All time-variant variables are measured in 2020 unless otherwise specified. Results are for the combined Younger Cohort/ Older Cohort sample.*

Supplementary Table 17. Logistic regression results: Symptoms of at least mild anxiety/depression by gender (India)

	At least mild anxiety				At least mild depression			
	Male		Female		Male		Female	
	Odds Ratio	95% CI	Odds Ratio	95% CI	Odds Ratio	95% CI	Odds Ratio	95% CI
COVID-19 related changes/behaviours								
Risk perception: believe they are at medium/high risk	1.520**<br>(0.30)	[1.038,2.224]	1.149<br>(0.22)	[0.795,1.661]	1.048<br>(0.20)	[0.722,1.522]	0.927<br>(0.19)	[0.616,1.395]
Left house for any reason in the past 7 days	0.744<br>(0.28)	[0.356,1.555]	1.606**<br>(0.36)	[1.040,2.482]	1.063<br>(0.43)	[0.482,2.342]	2.810***<br>(0.77)	[1.642,4.810]
Difference in subjective well-being between Round 5 (2016) and phone survey (2020)	0.999<br>(0.05)	[0.904,1.105]	1.245***<br>(0.07)	[1.121,1.383]	1.041<br>(0.05)	[0.950,1.141]	1.248***<br>(0.07)	[1.117,1.395]
Change in responsibilities:								
Spend more time taking care of children	1.708*<br>(0.50)	[0.967,3.015]	2.455***<br>(0.54)	[1.595,3.780]	1.327<br>(0.41)	[0.721,2.443]	1.668**<br>(0.43)	[1.007,2.761]
Spend more time on household chores	1.356	[0.923,1.993]	0.440***	[0.297,0.651]	1.337	[0.915,1.955]	0.576**	[0.376,0.883]

For peer review only

	At least mild anxiety				At least mild depression			
	Male		Female		Male		Female	
	Odds Ratio	95% CI	Odds Ratio	95% CI	Odds Ratio	95% CI	Odds Ratio	95% CI
	(0.27)		(0.09)		(0.26)		(0.13)	
Spend more time working in the family business	1.944*	[0.953,3.967]	1.210	[0.520,2.816]	1.100	[0.493,2.457]	1.043	[0.348,3.122]
	(0.71)		(0.52)		(0.45)		(0.58)	
Economic shocks								
Faced with new health expenses	0.786	[0.532,1.161]	1.349	[0.924,1.968]	1.203	[0.799,1.810]	1.477*	[0.969,2.250]
	(0.16)		(0.26)		(0.25)		(0.32)	
Experienced adversity but did not reduce food consumption	0.894	[0.169,4.723]	0.500	[0.155,1.609]	1.749	[0.207,14.799]	0.384*	[0.130,1.137]
	(0.76)		(0.30)		(1.91)		(0.21)	
Reduced food consumption as response to experienced adversity	1.272	[0.209,7.752]	0.691	[0.177,2.697]	1.651	[0.169,16.089]	0.398	[0.099,1.608]
	(1.17)		(0.48)		(1.92)		(0.28)	
Changes in employment status								
Did not work before the pandemic, but is working now	0.629	[0.326,1.213]	1.267	[0.687,2.334]	0.555*	[0.294,1.051]	0.887	[0.472,1.669]
	(0.21)		(0.40)		(0.18)		(0.29)	
Worked before the pandemic and is working now/has a job	0.982	[0.562,1.718]	1.504*	[0.978,2.314]	0.980	[0.591,1.626]	0.802	[0.485,1.328]
	(0.28)		(0.33)		(0.25)		(0.21)	
Worked before the pandemic and is not working now/does not have a job	2.812**	[1.266,6.245]	2.286**	[1.121,4.660]	2.908***	[1.367,6.188]	0.836	[0.325,2.148]
	(1.14)		(0.83)		(1.12)		(0.40)	
Background characteristics								
Age in months	0.999	[0.994,1.004]	1.003	[0.998,1.008]	0.998	[0.993,1.003]	1.002	[0.996,1.008]
	(0.00)		(0.00)		(0.00)		(0.00)	
Urban	0.428***	[0.252,0.728]	1.564**	[1.012,2.417]	0.502***	[0.311,0.808]	1.113	[0.679,1.824]
	(0.12)		(0.35)		(0.12)		(0.28)	
Participant has long-term health problem, 2016 (Round 5)	1.310	[0.746,2.299]	1.466	[0.924,2.326]	1.353	[0.773,2.366]	1.785**	[1.105,2.885]
	(0.38)		(0.35)		(0.39)		(0.44)	
Total parent-child relationship score, 2012/2016 (Round 4/5)	0.908***	[0.845,0.977]	0.934**	[0.880,0.991]	0.908***	[0.846,0.973]	0.909***	[0.848,0.974]
	(0.03)		(0.03)		(0.03)		(0.03)	
Total peer-child relationship score, 2016 (Round 5)	1.039	[0.965,1.119]	1.014	[0.944,1.089]	1.040	[0.961,1.125]	0.994	[0.924,1.069]
	(0.04)		(0.04)		(0.04)		(0.04)	
Middle/Top wealth tercile R5, 2016 (Round 5)	0.804	[0.534,1.210]	0.764	[0.504,1.159]	1.324	[0.881,1.989]	0.877	[0.559,1.375]
	(0.17)		(0.16)		(0.27)		(0.20)	
N	1369		1253		1369		1253	

*Note: Odds ratios are unadjusted odds ratios. Robust standard errors in parenthesis, *** significant at 1%, ** significant at 5%, * significant at 10%. Base categories are as follows: Believe they are at no/low risk, Did not leave the house at all during the past 7 days, Did not spend more time taking care of children, Did not spend more time on household chores, Did not spend more time working in the family business, Did not face new health expenses, Did not suffer a shock, Did not work at all in the past 12 month OR worked during the pandemic but not before and is not working now, Rural, Does not have long-term health condition, Lowest wealth tercile. All time-variant variables are measured in 2020 unless otherwise specified. Results are for the combined Younger Cohort/ Older Cohort sample.*

For peer review only

For peer review only - http://bmjopen.bmj.com/site/about/guidelines.xhtml

Supplementary Table 18. Logistic regression results: Symptoms of at least mild anxiety/depression by gender (Peru)

	At least mild anxiety				At least mild depression			
	Male		Female		Male		Female	
	Odds Ratio	95% CI	Odds Ratio	95% CI	Odds Ratio	95% CI	Odds Ratio	95% CI
<u>*COVID-19 related changes/behaviours*</u>								
Risk perception: believe they are at medium/high risk	1.326* (0.19)	[0.994,1.767]	1.585*** (0.22)	[1.204,2.086]	1.513*** (0.23)	[1.117,2.051]	1.657*** (0.24)	[1.241,2.212]
Left house for any reason in the past 7 days	1.324 (0.28)	[0.878,1.996]	0.848 (0.14)	[0.609,1.181]	1.052 (0.22)	[0.697,1.588]	1.067 (0.19)	[0.756,1.505]
Difference in subjective well-being between Round 5 (2016) and phone survey (2020)	1.182*** (0.05)	[1.097,1.274]	1.064* (0.03)	[0.998,1.134]	1.131*** (0.05)	[1.043,1.227]	1.047 (0.04)	[0.978,1.121]
Change in responsibilities:								
Spend more time taking care of children	1.567*** (0.26)	[1.134,2.165]	1.285* (0.18)	[0.973,1.698]	1.529** (0.26)	[1.097,2.131]	0.932 (0.14)	[0.697,1.246]
Spend more time on household chores	1.220 (0.21)	[0.873,1.704]	1.293 (0.25)	[0.882,1.895]	1.039 (0.18)	[0.734,1.471]	1.105 (0.22)	[0.742,1.644]
Spend more time working in the family business	1.299 (0.25)	[0.896,1.885]	1.335 (0.29)	[0.871,2.046]	1.258 (0.25)	[0.848,1.865]	1.362 (0.30)	[0.883,2.101]
Economic shocks								
Faced with new health expenses	1.983*** (0.32)	[1.451,2.710]	1.532*** (0.23)	[1.148,2.044]	1.735*** (0.29)	[1.257,2.394]	1.757*** (0.26)	[1.308,2.361]
Experienced adversity but did not reduce food consumption	5.087** (3.94)	[1.114,23.224]	1.609 (0.88)	[0.548,4.728]	4.172* (3.08)	[0.983,17.705]	1.056 (0.63)	[0.330,3.378]
Reduced food consumption as response to experienced adversity	5.843** (4.83)	[1.157,29.524]	1.288 (0.77)	[0.397,4.178]	5.041** (4.02)	[1.057,24.045]	1.354 (0.87)	[0.383,4.782]
Changes in employment status								
Did not work before the pandemic, but is working now	0.684 (0.21)	[0.379,1.235]	0.945 (0.23)	[0.591,1.510]	1.065 (0.32)	[0.593,1.910]	0.790 (0.19)	[0.491,1.271]
Worked before the pandemic and is working now/has a job	0.958 (0.22)	[0.608,1.509]	1.049 (0.19)	[0.737,1.493]	0.899 (0.22)	[0.563,1.437]	0.799 (0.15)	[0.551,1.158]
Worked before the pandemic and is not working now/does not have a job	1.029 (0.27)	[0.616,1.719]	1.326 (0.26)	[0.900,1.952]	1.385 (0.37)	[0.824,2.329]	1.193 (0.25)	[0.797,1.786]
<u>*Background characteristics*</u>								
Age in months	1.001 (0.00)	[0.997,1.005]	0.998 (0.00)	[0.994,1.002]	0.996* (0.00)	[0.991,1.001]	0.996* (0.00)	[0.992,1.000]
Urban	1.245 (0.27)	[0.817,1.898]	1.610** (0.34)	[1.070,2.423]	0.978 (0.21)	[0.639,1.498]	1.222 (0.27)	[0.790,1.889]
Participant has long-term health problem, 2016 (Round 5)	1.794** (0.41)	[1.142,2.818]	1.937*** (0.38)	[1.318,2.848]	1.662** (0.39)	[1.047,2.640]	1.564** (0.29)	[1.081,2.262]
Total parent-child relationship score, 2012/2016	0.974	[0.931,1.020]	0.982	[0.947,1.019]	0.944**	[0.898,0.992]	0.936***	[0.900,0.973]

For peer review only

	At least mild anxiety				At least mild depression			
	Male		Female		Male		Female	
	Odds Ratio	95% CI	Odds Ratio	95% CI	Odds Ratio	95% CI	Odds Ratio	95% CI
(Round 4/5)	(0.02)		(0.02)		(0.02)		(0.02)	
Total peer-child relationship score, 2016 (Round 5)	1.010 (0.03)	[0.958,1.065]	0.990 (0.02)	[0.951,1.031]	1.002 (0.03)	[0.946,1.063]	1.011 (0.02)	[0.968,1.055]
Middle/Top wealth tercile R5, 2016 (Round 5)	0.777 (0.14)	[0.548,1.104]	1.257 (0.22)	[0.890,1.774]	1.137 (0.21)	[0.795,1.627]	1.294 (0.24)	[0.894,1.873]
N	957		930		957		930	

*Note: Odds ratios are unadjusted odds ratios. Robust standard errors in parenthesis, *** significant at 1%, ** significant at 5%, * significant at 10%. Base categories are as follows: Believe they are at no/low risk, Did not leave the house at all during the past 7 days, Did not spend more time taking care of children, Did not spend more time on household chores, Did not spend more time working in the family business, Did not face new health expenses, Did not suffer a shock, Did not work at all in the past 12 month OR worked during the pandemic but not before and is not working now, Rural, Does not have long-term health condition, Lowest wealth tercile. All time-variant variables are measured in 2020 unless otherwise specified. Results are for the combined Younger Cohort/ Older Cohort sample.*

Supplementary Table 19. Logistic regression results: Symptoms of at least mild anxiety/depression by gender (Vietnam)

	At least mild anxiety				At least mild depression			
	Male		Female		Male		Female	
	Odds Ratio	95% CI	Odds Ratio	95% CI	Odds Ratio	95% CI	Odds Ratio	95% CI
COVID-19 related changes/behaviours								
Risk perception: believe they are at medium/high risk	1.548* (0.41)	[0.921,2.603]	0.816 (0.21)	[0.498,1.337]	2.094** (0.61)	[1.180,3.715]	0.828 (0.19)	[0.525,1.304]
Left house for any reason in the past 7 days	0.666 (0.22)	[0.353,1.254]	1.535 (0.51)	[0.802,2.936]	0.748 (0.25)	[0.388,1.439]	1.225 (0.36)	[0.694,2.164]
Difference in subjective well-being between Round 5 (2016) and phone survey (2020)	1.296*** (0.10)	[1.116,1.506]	1.111* (0.06)	[0.994,1.241]	1.471*** (0.11)	[1.269,1.706]	1.016 (0.05)	[0.914,1.129]
Change in responsibilities:								
Spend more time taking care of children	1.222 (0.37)	[0.673,2.220]	1.520* (0.35)	[0.971,2.381]	1.221 (0.39)	[0.648,2.300]	1.506* (0.34)	[0.970,2.339]
Spend more time on	1.191 (0.27)	[0.757,1.872]	1.191 (0.26)	[0.777,1.825]	1.076 (0.27)	[0.655,1.770]	1.996*** (0.46)	[1.277,3.122]

	At least mild anxiety				At least mild depression			
	Male		Female		Male		Female	
	Odds Ratio	95% CI	Odds Ratio	95% CI	Odds Ratio	95% CI	Odds Ratio	95% CI
household chores								
Spend more time working in the family business	2.394*** (0.75)	[1.292,4.434]	1.624* (0.44)	[0.950,2.777]	2.373*** (0.76)	[1.261,4.463]	1.875** (0.48)	[1.132,3.106]
Economic shocks								
Faced with new health expenses	0.544 (0.29)	[0.191,1.547]	1.757* (0.57)	[0.933,3.309]	1.093 (0.49)	[0.453,2.636]	0.721 (0.27)	[0.345,1.508]
Experienced adversity but did not reduce food consumption	2.494*** (0.70)	[1.440,4.320]	1.230 (0.28)	[0.787,1.925]	2.430*** (0.71)	[1.370,4.309]	1.416 (0.33)	[0.902,2.224]
Reduced food consumption as response to experienced adversity	2.367** (0.86)	[1.159,4.833]	1.357 (0.38)	[0.785,2.346]	1.883* (0.72)	[0.889,3.987]	1.946** (0.51)	[1.162,3.259]
Changes in employment status								
Did not work before the pandemic, but is working now	1.605 (0.84)	[0.574,4.486]	0.814 (0.34)	[0.357,1.857]	0.254 (0.25)	[0.036,1.782]	0.913 (0.34)	[0.436,1.910]
Worked before the pandemic and is working now/has a job	1.127 (0.42)	[0.545,2.331]	0.948 (0.24)	[0.575,1.565]	1.157 (0.41)	[0.576,2.324]	0.708 (0.17)	[0.441,1.136]
Worked before the pandemic and is not working now/does not have a job	1.798 (0.72)	[0.817,3.958]	1.178 (0.37)	[0.635,2.187]	1.921* (0.75)	[0.898,4.108]	1.456 (0.40)	[0.848,2.502]
Background characteristics								
Age in months	0.996 (0.00)	[0.990,1.002]	0.994** (0.00)	[0.988,1.000]	0.992** (0.00)	[0.985,0.999]	0.994** (0.00)	[0.988,1.000]
Urban	0.960 (0.22)	[0.608,1.517]	1.358 (0.27)	[0.916,2.014]	0.837 (0.22)	[0.500,1.402]	1.091 (0.22)	[0.740,1.607]
Participant has long-term health problem, 2016 (Round 5)	1.944** (0.58)	[1.088,3.474]	1.055 (0.29)	[0.612,1.819]	2.231** (0.74)	[1.167,4.266]	1.063 (0.28)	[0.631,1.789]
Total parent-child relationship score, 2012/2016 (Round 4/5)	1.015 (0.04)	[0.940,1.096]	1.056 (0.04)	[0.989,1.127]	0.983 (0.04)	[0.904,1.071]	0.982 (0.03)	[0.925,1.043]
Total peer-child relationship score, 2016 (Round 5)	1.014 (0.06)	[0.907,1.133]	0.897** (0.04)	[0.814,0.989]	1.055 (0.07)	[0.928,1.199]	0.961 (0.04)	[0.877,1.052]

For peer review only

	At least mild anxiety				At least mild depression			
	Male		Female		Male		Female	
	**Odds Ratio**	**95% CI**	**Odds Ratio**	**95% CI**	**Odds Ratio**	**95% CI**	**Odds Ratio**	**95% CI**
Middle/Top wealth tercile R5, 2016 (Round 5)	0.551** (0.14)	[0.336,0.903]	0.566*** (0.12)	[0.379,0.844]	1.015 (0.28)	[0.588,1.754]	1.130 (0.24)	[0.745,1.713]
N	1111		1185		1111		1185	

*Note: Odds ratios are unadjusted odds ratios. Robust standard errors in parenthesis, *** significant at 1%, ** significant at 5%, * significant at 10%. Base categories are as follows: Believe they are at no/low risk, Did not leave the house at all during the past 7 days, Did not spend more time taking care of children, Did not spend more time on household chores, Did not spend more time working in the family business, Did not face new health expenses, Did not suffer a shock, Did not work at all in the past 12 month OR worked during the pandemic but not before and is not working now, Rural, Does not have long-term health condition, Lowest wealth tercile. All time-variant variables are measured in 2020 unless otherwise specified. Results are for the combined Younger Cohort/ Older Cohort sample.*

For peer review only

Supplementary Table 20. Logistic regression results: Symptoms of at least mild anxiety/depression by gender for the Younger Cohort only (Ethiopia)

	At least mild anxiety				At least mild depression			
	Male		Female		Male		Female	
	Odds Ratio	95% CI	Odds Ratio	95% CI	Odds Ratio	95% CI	Odds Ratio	95% CI
COVID-19 related changes/behaviours								
Risk perception: believe they are at medium/high risk	0.550** (0.13)	[0.348,0.869]	0.875 (0.22)	[0.540,1.420]	0.461*** (0.11)	[0.290,0.733]	0.512*** (0.12)	[0.324,0.810]
Left house for any reason in the past 7 days	0.620 (0.29)	[0.249,1.545]	1.140 (0.47)	[0.504,2.579]	0.702 (0.34)	[0.269,1.832]	1.129 (0.42)	[0.546,2.336]
Difference in subjective well-being between Round 5 (2016) and phone survey (2020)	1.076 (0.05)	[0.973,1.189]	1.001 (0.06)	[0.897,1.117]	1.015 (0.06)	[0.910,1.131]	1.083 (0.06)	[0.975,1.204]
Change in responsibilities:								
Spend more time taking care of children	1.481 (0.44)	[0.823,2.666]	0.754 (0.19)	[0.460,1.236]	0.839 (0.25)	[0.463,1.522]	1.224 (0.30)	[0.757,1.979]
Spend more time on household chores	0.780 (0.19)	[0.479,1.271]	1.401 (0.41)	[0.793,2.475]	1.083 (0.26)	[0.671,1.749]	1.132 (0.32)	[0.647,1.979]
Spend more time working in the family business	0.542 (0.23)	[0.239,1.233]	0.842 (0.39)	[0.344,2.064]	0.475 (0.22)	[0.189,1.195]	2.336** (0.88)	[1.120,4.872]
Economic shocks								
Faced with new health expenses	0.673 (0.31)	[0.272,1.666]	0.645 (0.28)	[0.273,1.524]	0.278** (0.17)	[0.083,0.931]	0.199** (0.13)	[0.057,0.695]
Experienced adversity but did not reduce food consumption	3.346*** (1.34)	[1.530,7.320]	3.633*** (1.42)	[1.692,7.801]	6.595*** (3.49)	[2.338,18.604]	3.859*** (1.52)	[1.785,8.340]
Reduced food consumption as response to experienced adversity	6.188*** (2.97)	[2.418,15.835]	9.701*** (4.34)	[4.035,23.323]	19.255*** (11.13)	[6.202,59.780]	8.538*** (3.98)	[3.427,21.271]
Educational disruption								
Enrolled in/Planning to enrol in full time education and not participating in learning activities	1.188 (0.33)	[0.693,2.039]	2.556*** (0.87)	[1.313,4.972]	1.520 (0.45)	[0.845,2.732]	2.033** (0.62)	[1.122,3.683]
Enrolled in/Planning to enrol in full time education and participating in learning activities	0.810 (0.46)	[0.269,2.436]	1.600 (0.79)	[0.608,4.207]	0.735 (0.48)	[0.207,2.613]	1.524 (0.68)	[0.635,3.656]
Background characteristics								
Age in months	1.011 (0.03)	[0.952,1.074]	0.990 (0.03)	[0.937,1.047]	1.005 (0.03)	[0.946,1.068]	0.997 (0.03)	[0.944,1.054]
Urban	1.421 (0.39)	[0.831,2.430]	1.712* (0.51)	[0.953,3.075]	1.341 (0.37)	[0.783,2.296]	1.634* (0.45)	[0.956,2.792]
Participant has long-term health problem, 2016 (Round 5)	1.185 (0.57)	[0.464,3.028]	1.291 (0.47)	[0.630,2.646]	0.358 (0.26)	[0.086,1.494]	0.621 (0.28)	[0.260,1.483]

For peer review only

	At least mild anxiety				At least mild depression			
	Male		Female		Male		Female	
	Odds Ratio	95% CI	Odds Ratio	95% CI	Odds Ratio	95% CI	Odds Ratio	95% CI
Total parent-child relationship score, 2012/2016 (Round 4/5)	0.971 (0.04)	[0.890,1.059]	0.943 (0.04)	[0.858,1.035]	1.056 (0.04)	[0.974,1.145]	0.970 (0.04)	[0.889,1.059]
Total peer-child relationship score, 2016 (Round 5)	0.934 (0.05)	[0.848,1.028]	0.970 (0.04)	[0.893,1.054]	0.892** (0.05)	[0.808,0.985]	0.915** (0.04)	[0.842,0.994]
Middle/Top wealth tercile R5, 2016 (Round 5)	0.922 (0.26)	[0.528,1.611]	0.923 (0.28)	[0.507,1.682]	0.792 (0.23)	[0.453,1.383]	0.756 (0.21)	[0.434,1.315]
N	825		708		825		708	

*Note: Odds ratios are unadjusted odds ratios. Robust standard errors in parenthesis, *** significant at 1%, ** significant at 5%, * significant at 10%. Base categories are as follows: Believe they are at no/low risk, Did not leave the house at all during the past 7 days, Did not spend more time taking care of children, Did not spend more time on household chores, Did not spend more time working in the family business, Did not face new health expenses, Did not suffer a shock, Never attended school or not enrolled in full-time education/not planning to enrol, Rural, Does not have long-term health condition, Lowest wealth tercile. All time-variant variables are measured in 2020 unless otherwise specified.*

Supplementary Table 21. Logistic regression results: Symptoms of at least mild anxiety/depression by gender for the Younger Cohort only (India)

	At least mild anxiety				At least mild depression			
	Male		Female		Male		Female	
	Odds Ratio	95% CI	Odds Ratio	95% CI	Odds Ratio	95% CI	Odds Ratio	95% CI
COVID-19 related changes/behaviours								
Risk perception: believe they are at medium/high risk	1.455 (0.34)	[0.914,2.315]	0.995 (0.25)	[0.605,1.639]	0.969 (0.22)	[0.622,1.509]	0.989 (0.26)	[0.586,1.669]
Left house for any reason in the past 7 days	0.517* (0.21)	[0.238,1.125]	2.471*** (0.77)	[1.337,4.565]	0.913 (0.39)	[0.392,2.124]	3.923*** (1.55)	[1.811,8.496]
Difference in subjective well-being between Round 5 (2016) and phone survey (2020)	1.012 (0.06)	[0.898,1.141]	1.243*** (0.09)	[1.083,1.427]	1.027 (0.06)	[0.919,1.148]	1.215*** (0.09)	[1.058,1.395]
Change in responsibilities:								
Spend more time taking care of children	2.137* (0.90)	[0.935,4.884]	2.311** (0.76)	[1.213,4.403]	1.118 (0.58)	[0.407,3.074]	3.427*** (1.13)	[1.798,6.534]
Spend more time on household chores	1.370 (0.35)	[0.826,2.274]	0.517*** (0.13)	[0.317,0.843]	1.207 (0.30)	[0.738,1.973]	0.673 (0.19)	[0.390,1.162]
Spend more time working in the family business	2.020* (0.86)	[0.879,4.640]	2.424** (1.09)	[1.005,5.849]	0.750 (0.38)	[0.278,2.021]	0.832 (0.58)	[0.212,3.262]
Economic shocks								

For peer review only

	At least mild anxiety				At least mild depression			
	Male		Female		Male		Female	
	Odds Ratio	95% CI	Odds Ratio	95% CI	Odds Ratio	95% CI	Odds Ratio	95% CI
Faced with new health expenses	0.670 (0.17)	[0.413,1.088]	1.165 (0.30)	[0.699,1.944]	1.037 (0.25)	[0.645,1.667]	1.171 (0.36)	[0.646,2.121]
Experienced adversity but did not reduce food consumption	0.627 (0.52)	[0.125,3.157]	0.691 (0.53)	[0.152,3.137]	1.449 (1.56)	[0.177,11.888]	0.313* (0.21)	[0.085,1.153]
Reduced food consumption as response to experienced adversity	1.181 (1.09)	[0.193,7.208]	0.882 (0.78)	[0.155,5.013]	1.943 (2.25)	[0.201,18.787]	0.481 (0.42)	[0.087,2.668]
Educational disruption								
Enrolled in/Planning to enrol in full time education and not participating in learning activities	0.934 (0.27)	[0.530,1.644]	1.134 (0.34)	[0.628,2.049]	0.914 (0.26)	[0.520,1.607]	1.456 (0.47)	[0.770,2.753]
Enrolled in/Planning to enrol in full time education and participating in learning activities	0.684 (0.24)	[0.342,1.369]	0.659 (0.23)	[0.328,1.324]	0.540* (0.19)	[0.276,1.057]	1.122 (0.42)	[0.540,2.332]
Background characteristics								
Age in months	1.007 (0.03)	[0.947,1.069]	0.971 (0.03)	[0.911,1.034]	1.013 (0.03)	[0.955,1.075]	0.968 (0.03)	[0.908,1.031]
Urban	0.570* (0.18)	[0.303,1.070]	2.637*** (0.74)	[1.517,4.585]	0.769 (0.22)	[0.439,1.349]	1.367 (0.45)	[0.720,2.594]
Participant has long-term health problem, 2016 (Round 5)	1.336 (0.46)	[0.676,2.640]	1.199 (0.46)	[0.568,2.530]	1.447 (0.49)	[0.748,2.802]	1.429 (0.53)	[0.692,2.951]
Total parent-child relationship score, 2012/2016 (Round 4/5)	0.918* (0.04)	[0.838,1.007]	0.922* (0.04)	[0.846,1.005]	0.921* (0.04)	[0.847,1.001]	0.864*** (0.04)	[0.787,0.948]
Total peer-child relationship score, 2016 (Round 5)	0.998 (0.05)	[0.908,1.096]	1.011 (0.05)	[0.910,1.123]	0.973 (0.05)	[0.877,1.080]	1.040 (0.06)	[0.934,1.157]
Middle/Top wealth tercile R5, 2016 (Round 5)	0.877 (0.23)	[0.524,1.468]	0.586* (0.17)	[0.333,1.033]	1.494 (0.41)	[0.877,2.546]	1.129 (0.34)	[0.627,2.033]
N	958		828		958		828	

*Note: Odds ratios are unadjusted odds ratios. Robust standard errors in parenthesis, *** significant at 1%, ** significant at 5%, * significant at 10%. Base categories are as follows: Believe they are at no/low risk, Did not leave the house at all during the past 7 days, Did not spend more time taking care of children, Did not spend more time on household chores, Did not spend more time working in the family business, Did not face new health expenses, Did not suffer a shock, Never attended school or not enrolled in full-time education/not planning to enrol, Rural, Does not have long-term health condition, Lowest wealth tercile. All time-variant variables are measured in 2020 unless otherwise specified.*

For peer review only

Supplementary Table 22. Logistic regression results: Symptoms of at least mild anxiety/depression by gender for the Younger Cohort only (Peru)

	At least mild anxiety				At least mild depression			
	Male		Female		Male		Female	
	Odds Ratio	95% CI	Odds Ratio	95% CI	Odds Ratio	95% CI	Odds Ratio	95% CI
COVID-19 related changes/behaviours								
Risk perception: believe they are at medium/high risk	1.375* (0.23)	[0.988,1.913]	1.488*** (0.23)	[1.100,2.014]	1.500** (0.26)	[1.064,2.114]	1.621*** (0.26)	[1.180,2.226]
Left house for any reason in the past 7 days	1.228 (0.27)	[0.804,1.876]	0.931 (0.17)	[0.650,1.332]	0.998 (0.22)	[0.645,1.546]	0.888 (0.17)	[0.614,1.284]
Difference in subjective well-being between Round 5 (2016) and phone survey (2020)	1.190*** (0.05)	[1.097,1.291]	1.060* (0.04)	[0.989,1.135]	1.137*** (0.05)	[1.036,1.247]	1.045 (0.04)	[0.970,1.126]
Change in responsibilities:								
Spend more time taking care of children	1.784*** (0.33)	[1.238,2.571]	1.384** (0.22)	[1.013,1.890]	1.823*** (0.35)	[1.247,2.665]	0.965 (0.16)	[0.699,1.331]
Spend more time on household chores	1.233 (0.25)	[0.832,1.826]	1.176 (0.25)	[0.771,1.792]	1.118 (0.23)	[0.748,1.671]	1.121 (0.24)	[0.731,1.720]
Spend more time working in the family business	1.094 (0.24)	[0.712,1.681]	1.426 (0.33)	[0.911,2.230]	1.038 (0.24)	[0.664,1.622]	1.238 (0.29)	[0.787,1.950]
Economic shocks								
Faced with new health expenses	2.093*** (0.38)	[1.460,3.000]	1.530*** (0.25)	[1.109,2.110]	2.089*** (0.40)	[1.435,3.041]	1.701*** (0.28)	[1.226,2.360]
Experienced adversity but did not reduce food consumption	4.608** (3.59)	[1.003,21.173]	1.475 (0.93)	[0.427,5.092]	3.693* (2.66)	[0.899,15.172]	1.615 (1.06)	[0.445,5.863]
Reduced food consumption as response to experienced adversity	3.800 (3.22)	[0.721,20.038]	1.249 (0.85)	[0.327,4.769]	4.074* (3.27)	[0.845,19.648]	2.556 (1.83)	[0.631,10.360]
Educational disruption								
Enrolled in/Planning to enrol in full time education and not participating in learning activities	0.559 (0.44)	[0.120,2.602]	0.552 (0.33)	[0.173,1.763]	1.190 (0.94)	[0.253,5.599]	0.407 (0.28)	[0.107,1.546]
Enrolled in/Planning to enrol in full time education and participating in learning activities	0.894 (0.16)	[0.625,1.278]	1.127 (0.18)	[0.825,1.538]	1.215 (0.23)	[0.841,1.754]	0.898 (0.15)	[0.646,1.248]
Background characteristics								
Age in months	0.981 (0.02)	[0.939,1.024]	1.004 (0.02)	[0.965,1.045]	0.953** (0.02)	[0.910,0.997]	0.991 (0.02)	[0.952,1.033]
Urban	1.090 (0.26)	[0.684,1.736]	1.658** (0.37)	[1.070,2.569]	1.043 (0.25)	[0.651,1.670]	1.268 (0.30)	[0.793,2.029]
Participant has long-term health problem, 2016 (Round 5)	2.008*** (0.54)	[1.186,3.398]	1.493* (0.33)	[0.964,2.312]	1.910** (0.53)	[1.108,3.293]	1.269 (0.28)	[0.827,1.947]
Total parent-child relationship score, 2012/2016 (Round 4/5)	0.973 (0.03)	[0.922,1.028]	0.977 (0.02)	[0.938,1.019]	0.907*** (0.03)	[0.856,0.961]	0.931*** (0.02)	[0.891,0.973]

For peer review only

	At least mild anxiety				At least mild depression			
	Male		Female		Male		Female	
	Odds Ratio	95% CI	Odds Ratio	95% CI	Odds Ratio	95% CI	Odds Ratio	95% CI
Total peer-child relationship score, 2016 (Round 5)	1.014 (0.03)	[0.954,1.078]	0.989 (0.02)	[0.947,1.034]	1.034 (0.03)	[0.968,1.104]	1.023 (0.02)	[0.975,1.072]
Middle/Top wealth tercile R5, 2016 (Round 5)	0.892 (0.18)	[0.600,1.325]	1.247 (0.24)	[0.857,1.816]	1.034 (0.21)	[0.695,1.537]	1.239 (0.26)	[0.821,1.871]
N	745		751		745		751	

*Note: Odds ratios are unadjusted odds ratios. Robust standard errors in parenthesis, *** significant at 1%, ** significant at 5%, * significant at 10%. Base categories are as follows: Believe they are at no/low risk, Did not leave the house at all during the past 7 days, Did not spend more time taking care of children, Did not spend more time on household chores, Did not spend more time working in the family business, Did not face new health expenses, Did not suffer a shock, Never attended school or not enrolled in full-time education/not planning to enrol, Rural, Does not have long-term health condition, Lowest wealth tercile. All time-variant variables are measured in 2020 unless otherwise specified.*

Supplementary Table 23. Logistic regression results: Symptoms of at least mild anxiety/depression by gender for the Younger Cohort only (Vietnam)

	At least mild anxiety				At least mild depression			
	Male		Female		Male		Female	
	Odds Ratio	95% CI	Odds Ratio	95% CI	Odds Ratio	95% CI	Odds Ratio	95% CI
<u>*COVID-19 related changes/behaviours*</u>								
Risk perception: believe they are at medium/high risk	1.553 (0.49)	[0.834,2.890]	0.830 (0.25)	[0.464,1.482]	1.711 (0.61)	[0.851,3.439]	0.775 (0.22)	[0.446,1.347]
Left house for any reason in the past 7 days	0.862 (0.33)	[0.409,1.814]	1.783 (0.64)	[0.879,3.617]	0.944 (0.37)	[0.441,2.022]	1.480 (0.47)	[0.793,2.759]
Difference in subjective well-being between Round 5 (2016) and phone survey (2020)	1.247*** (0.10)	[1.063,1.462]	1.154** (0.07)	[1.021,1.305]	1.444*** (0.12)	[1.221,1.708]	1.008 (0.06)	[0.896,1.133]
Change in responsibilities:								
Spend more time taking care of children	1.005 (0.41)	[0.452,2.238]	1.720** (0.46)	[1.019,2.903]	1.425 (0.55)	[0.667,3.045]	1.796** (0.44)	[1.111,2.902]
Spend more time on household chores	0.948 (0.25)	[0.570,1.575]	1.057 (0.26)	[0.654,1.706]	1.079 (0.31)	[0.619,1.880]	2.902*** (0.80)	[1.694,4.972]
Spend more time working in the family business	2.053** (0.73)	[1.018,4.140]	1.509 (0.46)	[0.830,2.745]	2.214** (0.79)	[1.099,4.459]	1.750* (0.50)	[0.998,3.068]
Economic shocks								
Faced with new health expenses	0.930 (0.50)	[0.323,2.684]	1.523 (0.62)	[0.688,3.368]	1.294 (0.63)	[0.498,3.365]	0.476 (0.24)	[0.181,1.255]

For peer review only

	At least mild anxiety				At least mild depression			
	Male		Female		Male		Female	
	Odds Ratio	95% CI	Odds Ratio	95% CI	Odds Ratio	95% CI	Odds Ratio	95% CI
Experienced adversity but did not reduce food consumption	2.415*** (0.77)	[1.295,4.502]	1.091 (0.29)	[0.652,1.826]	2.411*** (0.81)	[1.245,4.668]	1.502 (0.40)	[0.888,2.542]
Reduced food consumption as response to experienced adversity	2.963*** (1.22)	[1.320,6.652]	1.166 (0.37)	[0.628,2.165]	2.427** (1.04)	[1.050,5.613]	2.449*** (0.74)	[1.350,4.441]
Educational disruption								
Enrolled in/Planning to enrol in full time education and not participating in learning activities	0.954 (0.44)	[0.390,2.333]	0.817 (0.34)	[0.358,1.862]	1.153 (0.58)	[0.431,3.085]	0.967 (0.35)	[0.476,1.966]
Enrolled in/Planning to enrol in full time education and participating in learning activities	0.678 (0.23)	[0.350,1.315]	0.714 (0.19)	[0.420,1.216]	1.250 (0.43)	[0.639,2.445]	1.058 (0.29)	[0.622,1.799]
Background characteristics								
Age in months	1.009 (0.04)	[0.933,1.091]	1.027 (0.04)	[0.954,1.106]	1.051 (0.04)	[0.976,1.131]	1.014 (0.04)	[0.938,1.095]
Urban	1.026 (0.30)	[0.576,1.826]	1.550* (0.41)	[0.921,2.609]	0.735 (0.24)	[0.386,1.399]	1.562* (0.40)	[0.942,2.590]
Participant has long-term health problem, 2016 (Round 5)	1.190 (0.58)	[0.458,3.088]	1.036 (0.37)	[0.517,2.074]	2.696** (1.17)	[1.151,6.317]	1.204 (0.40)	[0.630,2.300]
Total parent-child relationship score, 2012/2016 (Round 4/5)	1.004 (0.05)	[0.918,1.098]	1.055 (0.04)	[0.981,1.135]	0.925 (0.05)	[0.833,1.026]	0.999 (0.04)	[0.931,1.072]
Total peer-child relationship score, 2016 (Round 5)	1.058 (0.06)	[0.950,1.178]	0.916 (0.05)	[0.821,1.022]	1.063 (0.07)	[0.926,1.220]	0.948 (0.05)	[0.852,1.054]
Middle/Top wealth tercile R5, 2016 (Round 5)	0.583* (0.18)	[0.323,1.055]	0.741 (0.18)	[0.454,1.208]	1.314 (0.45)	[0.672,2.566]	1.164 (0.31)	[0.693,1.955]
N	790		806		790		806	

*Note: Odds ratios are unadjusted odds ratios. Robust standard errors in parenthesis, *** significant at 1%, ** significant at 5%, * significant at 10%. Base categories are as follows: Believe they are at no/low risk, Did not leave the house at all during the past 7 days, Did not spend more time taking care of children, Did not spend more time on household chores, Did not spend more time working in the family business, Did not face new health expenses, Did not suffer a shock, Never attended school or not enrolled in full-time education/not planning to enrol, Rural, Does not have long-term health condition, Lowest wealth tercile. All time-variant variables are measured in 2020 unless otherwise specified.*

For peer review only
For peer review only - http://bmjopen.bmj.com/site/about/guidelines.xhtml

Supplementary Table 24. Ethiopia: Mean score and rates of general anxiety disorder

	Mean GAD-7 score	CI95%	p-value	Minimal (0-4)	CI95%	p-value	Mild anxiety (5-9)	CI95%	p-value	Moderate anxiety (10-14)	CI95%	p-value	Severe anxiety (>=15)	CI95%	p-value
**Total**	**2.06**	**1.94; 2.19**		**82.13**	**80.46; 83.72**		**15.21**	**13.73; 16.78**		**2.34**	**1.74; 3.06**		**0.32**	**0.13; 0.66**	
Male	2.03	1.86; 2.20	0.603	82.52	80.35; 84.70	0.610	14.66	12.64; 16.69	0.445	2.47	1.58; 3.36	0.650	0.34	0.01; 0.67	0.856
Female	2.10	1.92; 2.28		81.68	79.30; 84.07		15.84	13.59; 18.10		2.18	1.28; 3.08		0.30	-0.04; 0.63	
Rural	1.78	1.62; 1.93	0.000	85.33	83.30; 87.35	0.000	12.55	10.66; 14.45	0.000	2.12	1.30; 2.94	0.470	0.00	0.00; 0.00	0.004
Urban	2.40	2.20; 2.60		78.39	75.84; 80.94		18.33	15.93; 20.72		2.59	1.61; 3.57		0.70	0.18; 1.21	
Poorest tercile	1.89	1.68; 2.10	0.056	85.10	82.49; 87.71	0.011	11.98	9.60; 14.36	0.003	2.65	1.47; 3.82	0.502	0.28	-0.11; 0.66	0.808
Middle/Richest terciles	2.15	1.99; 2.30		80.68	78.66; 82.71		16.79	14.88; 18.71		2.18	1.44; 2.93		0.34	0.04; 0.64	
No internet	1.90	1.74; 2.07	0.010	84.03	81.89; 86.18	0.017	13.20	11.22; 15.19	0.007	2.50	1.58; 3.41	0.608	0.27	-0.04; 0.57	0.653
With internet	2.23	2.05; 2.41		80.13	77.73; 82.53		17.33	15.05; 19.60		2.17	1.29; 3.04		0.38	0.01; 0.75	
N	2183														

Notes: If any missing answers to questions then the whole score is set to missing. p-values represent significance of t-test of equality between groups (Male-Female; Rural-Urban; Bottom-Top/Middle wealth tercile; Internet access through home computer/working smartphone (No-Yes)). Poorest and middle/richest terciles refer to the household's position in the 2016 (round 5) wealth distribution.
Results are for the combined Younger Cohort/Older Cohort sample.

For peer review only

Supplementary Table 25. Ethiopia: Mean score and rates of depression

	Mean PHQ-8 score	CI95%	p-val	PHQ (0-4)	CI95%	p-val	PHQ (5-9)	CI95%	p-val	PHQ (10-14)	CI95%	p-val	PHQ (15-19)	CI95%	p-val	PHQ (>=20)	CI95%	p-val
**Total**	**1.89**	**1.78; 2.01**		**84.56**	**82.98; 86.05**		**13.33**	**11.93; 14.83**		**1.97**	**1.43; 2.64**		**0.09**	**0.01; 0.33**		**0.05**	**0.00; 0.25**	
Male	1.86	1.70; 2.02	0.569	85.08	83.04; 87.12	0.470	12.79	10.87; 14.70	0.422	1.88	1.10; 2.65	0.733	0.17	-0.07; 0.41	0.189	0.09	-0.08; 0.25	0.354
Female	1.93	1.76; 2.10		83.96	81.69; 86.23		13.96	11.82; 16.10		2.08	1.20; 2.96		0.00	0.00; 0.00		0.00	0.00; 0.00	
Rural	1.66	1.52; 1.81	0.000	87.11	85.19; 89.02	0.000	11.54	9.71; 13.36	0.007	1.27	0.63; 1.91	0.011	0.08	-0.08; 0.25	0.909	0.00	0.00; 0.00	0.279
Urban	2.16	1.97; 2.34		81.57	79.17; 83.97		15.44	13.20; 17.68		2.79	1.77; 3.81		0.10	-0.10; 0.29		0.10	-0.10; 0.29	
Poorest tercile	1.75	1.55; 1.95	0.086	85.65	83.09; 88.22	0.323	12.53	10.11; 14.96	0.444	1.53	0.63; 2.43	0.303	0.28	-0.11; 0.66	0.043	0.00	0.00; 0.00	0.484
Middle/Richest terciles	1.96	1.82; 2.10		84.03	82.15; 85.90		13.72	11.96; 15.48		2.18	1.44; 2.93		0.00	0.00; 0.00		0.07	-0.07; 0.20	
No internet	1.69	1.54; 1.85	0.001	85.91	83.87; 87.94	0.074	12.49	10.55; 14.43	0.235	1.52	0.80; 2.23	0.118	0.09	-0.09; 0.26	0.970	0.00	0.00; 0.00	0.304
With internet	2.10	1.93; 2.28		83.15	80.89; 85.40		14.22	12.12; 16.32		2.45	1.52; 3.38		0.09	-0.09; 0.28		0.09	-0.09; 0.28	
N		2183																

Notes: If any missing answers to questions then the whole score is set to missing. p-values represent significance of t-test of equality between groups (Male-Female; Rural-Urban; Bottom-Top/Middle wealth tercile; Internet access through home computer/working smartphone (No-Yes)). Poorest and middle/richest terciles refer to the household's position in the 2016 (round 5) wealth distribution.
Results are for the combined Younger Cohort/ Older Cohort sample.

For peer review only

Supplementary Table 26.India: Mean score and rates of general anxiety disorder

	Mean GAD-7 score	CI95%	p-value	Minimal (0-4)	CI95%	p-value	Mild anxiety (5-9)	CI95%	p-value	Moderate anxiety (10-14)	CI95%	p-value	Severe anxiety (>=15)	CI95%	p-value
**Total**	**1.69**	**1.59; 1.78**		**88.94**	**87.68; 90.12**		**9.15**	**8.08; 10.32**		**1.64**	**1.19; 2.20**		**0.27**	**0.11; 0.55**	
Male	1.55	1.43; 1.67	0.003	90.72	89.19; 92.26	0.002	8.04	6.59; 9.48	0.038	1.10	0.54; 1.65	0.022	0.15	-0.06; 0.35	0.210
Female	1.84	1.69; 1.99		86.99	85.13; 88.86		10.38	8.69; 12.06		2.23	1.42; 3.05		0.40	0.05; 0.75	
Rural	1.76	1.65; 1.88	0.014	88.19	86.73; 89.66	0.055	9.56	8.23; 10.90	0.252	1.92	1.30; 2.55	0.071	0.32	0.06; 0.58	0.401
Urban	1.50	1.34; 1.66		90.80	88.73; 92.87		8.13	6.17; 10.09		0.93	0.24; 1.62		0.13	-0.13; 0.39	
Poorest tercile	1.83	1.66; 2.00	0.042	86.48	84.22; 88.74	0.004	11.02	8.95; 13.09	0.018	2.16	1.20; 3.12	0.137	0.34	-0.04; 0.73	0.602
Middle/Richest terciles	1.62	1.50; 1.73		90.18	88.79; 91.58		8.21	6.92; 9.50		1.38	0.83; 1.93		0.23	0.00; 0.45	
No internet	1.80	1.52; 2.08	0.410	87.84	84.11; 91.57	0.521	10.47	6.98; 13.97	0.403	1.69	0.22; 3.16	0.944	0.00	0.00; 0.00	0.345
With internet	1.67	1.57; 1.78		89.08	87.81; 90.35		8.99	7.82; 10.15		1.63	1.12; 2.15		0.30	0.08; 0.52	
N	2622														

Notes: If any missing answers to questions then the whole score is set to missing. p-values represent significance of t-test of equality between groups (Male-Female; Rural-Urban; Bottom-Top/Middle wealth tercile; Internet access through home computer/working smartphone (No-Yes)). Poorest and middle/richest terciles refer to the household's position in the 2016 (round 5) wealth distribution.
Results are for the combined Younger Cohort/ Older Cohort sample.

Supplementary Table 27. India: Mean score and rates of depression

	Mean PHQ-8 score	CI95%	p-value	No significant depressive symptoms(0-4)	CI95%	p-value	Mild depression (5-9)	CI95%	p-value	Moderate depressive symptoms(10-14)	CI95%	p-value	Moderately severe depressive symptoms(15-19)	CI95%	p-value
**Total**	**1.41**	**1.33; 1.5**		**90.08**	**88.88; 91.2**		**8.73**	**7.68; 9.88**		**1.03**	**0.68; 1.49**		**0.15**	**0.04; 0.39**	
Male	1.34	1.22; 1.45	0.067	90.36	88.79; 91.92	0.624	8.84	7.33; 10.34	0.843	0.73	0.28; 1.18	0.113	0.07	-0.07; 0.22	0.276
Female	1.50	1.37; 1.63		89.78	88.11; 91.46		8.62	7.06; 10.17		1.36	0.72; 2.00		0.24	-0.03; 0.51	
Rural	1.47	1.36; 1.58	0.039	89.16	87.75; 90.57	0.012	9.40	8.08; 10.72	0.056	1.28	0.77; 1.79	0.043	0.16	-0.02; 0.34	0.873
Urban	1.27	1.12; 1.42		92.40	90.50; 94.30		7.07	5.23; 8.90		0.40	-0.05; 0.85		0.13	-0.13; 0.39	
Poorest tercile	1.49	1.33; 1.65	0.220	89.20	87.15; 91.26	0.284	9.55	7.60; 11.49	0.296	1.02	0.36; 1.69	0.980	0.23	-0.09; 0.54	0.486
Middle/Richest terciles	1.37	1.27; 1.48		90.53	89.15; 91.90		8.32	7.03; 9.620		1.03	0.56; 1.51		0.11	-0.04; 0.27	
No internet	1.42	1.17; 1.67	0.941	89.86	86.42; 93.31	0.894	8.78	5.55; 12.02	0.974	1.35	0.03; 2.67	0.561	0.00	0.00; 0.00	0.475
With internet	1.41	1.32; 1.50		90.11	88.90; 91.33		8.73	7.58; 9.88		0.99	0.59; 1.39		0.17	0.00; 0.34	
N	2622														

Notes: If any missing answers to questions then the whole score is set to missing. p-values represent significance of t-test of equality between groups (Male-Female; Rural-Urban; Bottom-Top/Middle wealth tercile; Internet access through home computer/working smartphone (No-Yes)). Poorest and middle/richest terciles refer to the household's position in the 2016 (round 5) wealth distribution.
Results are for the combined Younger Cohort/ Older Cohort sample. No severe depressive symptoms were reported.

For peer review only

Supplementary Table 28. Peru: Mean score and rates of general anxiety disorder

	Mean GAD-7 score	CI95%	p-value	Minimal (0-4)	CI95%	p-value	Mild anxiety (5-9)	CI95%	p-value	Moderate anxiety (10-14)	CI95%	p-value	Severe anxiety (>=15)	CI95%	p-value
**Total**	**4.53**	**4.34; 4.72**		**59.14**	**56.88; 61.37**		**27.34**	**25.34; 29.42**		**10.55**	**9.20; 12.02**		**2.97**	**2.25; 3.84**	
Male	3.80	3.55; 4.04	0.000	66.35	63.36; 69.35	0.000	23.72	21.02; 26.42	0.000	7.94	6.23; 9.66	0.000	1.99	1.10; 2.87	0.011
Female	5.29	5.01; 5.57		51.72	48.51; 54.94		31.08	28.10; 34.05		13.23	11.05; 15.41		3.98	2.72; 5.24	
Rural	3.87	3.46; 4.27	0.001	66.29	61.37; 71.21	0.002	25.56	21.02; 30.10	0.402	5.62	3.22; 8.01	0.001	2.53	0.89; 4.16	0.588
Urban	4.69	4.47; 4.90		57.48	55.00; 59.96		27.76	25.51; 30.00		11.69	10.08; 13.30		3.07	2.20; 3.93	
Poorest tercile	4.05	3.74; 4.35	0.001	63.08	59.16; 66.99	0.020	27.01	23.41; 30.61	0.826	8.21	5.98; 10.43	0.026	1.71	0.66; 2.76	0.031
Middle/Richest terciles	4.75	4.51; 4.99		57.37	54.68; 60.06		27.50	25.07; 29.92		11.60	9.86; 13.34		3.53	2.53; 4.54	
No internet	4.96	4.14; 5.78	0.367	48.68	37.37; 60.00	0.058	36.84	25.92; 47.77	0.058	14.47	6.51; 22.44	0.255	0.00	0.00; 0.00	0.120
With internet	4.51	4.32; 4.71		59.58	57.32; 61.84		26.95	24.90; 28.99		10.38	8.97; 11.79		3.09	2.29; 3.89	
N	1887														

Notes: If any missing answers to questions then the whole score is set to missing. p-values represent significance of t-test of equality between groups (Male-Female; Rural-Urban; Bottom-Top/Middle wealth tercile; Internet access through home computer/working smartphone (No-Yes)). Poorest and middle/richest terciles refer to the household's position in the 2016 (round 5) wealth distribution.
Results are for the combined Younger Cohort/ Older Cohort sample.

For peer review only

Supplementary Table 29. Peru: Mean score and rates of depression

	Mean PHQ-8 score	CI 95%	p-val	PHQ (0-4)	CI 95%	p-val	PHQ (5-9)	CI 95%	p-val	PHQ (10-14)	CI 95%	p-val	PHQ (15-19)	CI 95%	p-val	PHQ (>=20)	CI 95%	p-val
**Total**	**3.61**	**3.43; 3.79**		**68.42**	**66.26; 70.51**		**21.94**	**20.09; 23.88**		**7.79**	**6.62; 9.09**		**1.48**	**0.99; 2.14**		**0.37**	**0.15; 0.76**	
Male	3.08	2.85; 3.32	0.000	72.62	69.79; 75.45	0.000	19.75	17.22; 22.27	0.020	6.58	5.01; 8.16	0.047	0.73	0.19; 1.27	0.006	0.31	-0.04; 0.67	0.677
Female	4.15	3.89; 4.41		64.09	61.00; 67.17		24.19	21.44; 26.95		9.03	7.19; 10.88		2.26	1.30; 3.21		0.43	0.01; 0.85	
Rural	3.11	2.73; 3.49	0.008	73.60	69.01; 78.18	0.020	19.38	15.27; 23.50	0.196	6.18	3.67; 8.69	0.208	0.84	-0.11; 1.79	0.267	0.00	0.00; 0.00	0.201
Urban	3.73	3.53; 3.92		67.21	64.86; 69.56		22.53	20.44; 24.63		8.16	6.79; 9.54		1.63	1.00; 2.27		0.46	0.12; 0.80	
Poorest tercile	3.07	2.79; 3.36	0.000	73.85	70.28; 77.41	0.001	19.66	16.43; 22.88	0.109	5.47	3.62; 7.32	0.012	0.68	0.01; 1.35	0.054	0.34	-0.13; 0.82	0.889
Middle/ Richest terciles	3.85	3.63; 4.07		65.98	63.40; 68.55		22.96	20.68; 25.25		8.83	7.29; 10.38		1.84	1.11; 2.57		0.38	0.05; 0.72	
No internet	3.34	2.62; 4.07	0.545	68.42	57.89; 78.95	0.999	25.00	15.19; 34.81	0.511	6.58	0.96; 12.19	0.688	0.00	0.00; 0.00	0.275	0.00	0.00; 0.00	0.587
With internet	3.62	3.44; 3.80		68.42	66.27; 70.56		21.81	19.91; 23.71		7.84	6.60; 9.08		1.55	0.98; 2.11		0.39	0.10; 0.67	
N	1887																	

Notes: If any missing answers to questions then the whole score is set to missing. p-values represent significance of t-test of equality between groups (Male-Female; Rural-Urban; Bottom-Top/Middle wealth tercile; Internet access through home computer/working smartphone (No-Yes)). Poorest and middle/richest terciles refer to the household's position in the 2016 (round 5) wealth distribution.
Results are for the combined Younger Cohort/ Older Cohort sample.

For peer review only

Supplementary Table 30. Vietnam: Mean score and rates of general anxiety disorder

	Mean GAD-7 score	CI95%	p-value	Minimal (0-4)	CI95%	p-value	Mild anxiety (5-9)	CI95%	p-value	Moderate anxiety (10-14)	CI95%	p-value	Severe anxiety (>=15)	CI95%	p-value
**Total**	**1.47**	**1.37; 1.56**		**90.68**	**89.42; 91.84**		**7.93**	**6.85; 9.11**		**1.09**	**0.71; 1.60**		**0.30**	**0.12; 0.63**	
Male	1.31	1.17; 1.44	0.001	92.08	90.49; 93.67	0.025	6.48	5.03; 7.93	0.013	1.17	0.54; 1.80	0.717	0.27	-0.04; 0.58	0.769
Female	1.62	1.48; 1.76		89.37	87.61; 91.12		9.28	7.63; 10.94		1.01	0.44; 1.58		0.34	0.01; 0.67	
Rural	1.47	1.34; 1.60	0.925	90.93	89.33; 92.53	0.652	7.62	6.15; 9.10	0.559	1.04	0.48; 1.61	0.819	0.40	0.05; 0.75	0.362
Urban	1.46	1.32; 1.60		90.38	88.60; 92.17		8.29	6.62; 9.95		1.14	0.50; 1.79		0.19	-0.07; 0.45	
Poorest tercile	1.60	1.42; 1.78	0.045	88.33	86.08; 90.59	0.006	10.00	7.89; 12.11	0.008	1.15	0.40; 1.90	0.830	0.51	0.01; 1.01	0.195
Middle/Richest terciles	1.40	1.29; 1.51		91.89	90.51; 93.26		6.86	5.59; 8.13		1.06	0.54; 1.57		0.20	-0.03; 0.42	
No internet	2.40	1.24; 3.56	0.017	77.14	63.02; 91.26	0.005	17.14	4.47; 29.82	0.042	2.86	-2.75; 8.46	0.310	2.86	-2.75; 8.46	0.006
With internet	1.45	1.36; 1.55		90.89	89.70; 92.08		7.78	6.68; 8.89		1.06	0.64; 1.48		0.27	0.05; 0.48	
N	2296														

Notes: If any missing answers to questions then the whole score is set to missing. p-values represent significance of t-test of equality between groups (Male-Female; Rural-Urban; Bottom-Top/Middle wealth tercile; Internet access through home computer/working smartphone (No-Yes)). Poorest and middle/richest terciles refer to the household's position in the 2016 (round 5) wealth distribution.
Results are for the combined Younger Cohort/ Older Cohort sample.

For peer review only

Supplementary Table 31. Vietnam: Mean score and rates of depression

	Mean PHQ-8 score	CI95%	p-value	No significant depressive symptoms(0-4)	CI95%	p-value	Mild depression (5-9)	CI95%	p-value	Moderate depr-essive symptoms (10-14)	CI95%	p-value	Moderately severe depressive symptoms(15-19)	CI95%	p-value
**Total**	**1.42**	**1.32; 1.52**		**90.51**	**89.23; 91.67**		**7.67**	**6.61; 8.83**		**1.39**	**0.96; 1.96**		**0.44**	**0.21; 0.80**	
Male	1.24	1.11; 1.37	0.000	92.35	90.78; 93.91	0.004	6.12	4.71; 7.53	0.007	1.17	0.54; 1.80	0.376	0.36	0.01; 0.71	0.595
Female	1.59	1.45; 1.74		88.78	86.98; 90.58		9.11	7.47; 10.75		1.60	0.89; 2.32		0.51	0.10; 0.91	
Rural	1.43	1.30; 1.56	0.852	90.61	88.99; 92.23	0.852	7.70	6.22; 9.19	0.939	1.20	0.6; 1.81	0.398	0.48	0.10; 0.87	0.716
Urban	1.41	1.26; 1.56		90.38	88.60; 92.17		7.62	6.01; 9.23		1.62	0.85; 2.38		0.38	0.01; 0.75	
Poorest tercile	1.40	1.23; 1.57	0.757	91.54	89.58; 93.49	0.226	6.28	4.58; 7.99	0.074	1.54	0.67; 2.40	0.671	0.64	0.08; 1.20	0.284
Middle/Richest terciles	1.43	1.31; 1.55		89.97	88.46; 91.49		8.38	6.98; 9.77		1.32	0.74; 1.89		0.33	0.04; 0.62	
No internet	2.20	1.05; 3.35	0.053	85.71	73.95; 97.48	0.330	8.57	-0.84; 17.99	0.839	2.86	-2.75; 8.46	0.457	2.86	-2.75; 8.46	0.028
With internet	1.41	1.31; 1.51		90.58	89.37; 91.78		7.65	6.55; 8.75		1.37	0.89; 1.85		0.40	0.14; 0.66	
N	2296														

Notes: If any missing answers to questions then the whole score is set to missing. p-values represent significance of t-test of equality between groups (Male-Female; Rural-Urban; Bottom-Top/Middle wealth tercile; Internet access through home computer/working smartphone (No-Yes)). Poorest and middle/richest terciles refer to the household's position in the 2016 (round 5) wealth distribution.
Results are for the combined Younger Cohort/ Older Cohort sample. No severe depressive symptoms were reported.

For peer review only

Supplementary Table 32. Correlation between GAD-7 and PHQ-8 scores

	Ethiopia	India	Peru	Vietnam
Correlation coefficient	0.629***	0.610***	0.700***	0.636***

*Note: Bonferroni corrected p-value *** significant at 1%, ** significant at 5%, * significant at 10% for the unconditional correlation between GAD-7 and PHQ-8. Results are for the combined Younger Cohort/ Older Cohort sample.*

Supplementary Table 33. Joint rates

	Ethiopia		India		Peru		Vietnam	
	% of sample	95% CI	% of sample	95% CI	% of sample	95% CI	% of sample	95% CI
Display neither symptoms of anxiety nor depression	75.45	73.58; 77.24	83.87	82.40; 85.26	52.36	50.08; 54.63	85.50	83.99; 86.91
Display symptoms of at least mild anxiety only	9.12	7.94; 10.4	6.22	5.32; 7.21	16.06	14.43; 17.79	5.01	4.15; 5.98
Display symptoms of at least mild depression only	6.69	7.94; 10.4	5.07	5.32; 7.21	6.78	14.43; 17.79	5.18	4.15; 5.98
Display symptoms of at least mild anxiety and at least mild depression	8.75	7.60; 10.01	4.84	4.05; 5.74	24.80	22.87; 26.81	4.31	3.52; 5.22

Note: Results are for the combined Younger Cohort/ Older Cohort sample.

For peer review only

Supplementary Table 34. Correlation between subjective well-being and GAD-7/PHQ-8 scores

	Ethiopia				India				Peru				Vietnam			
	Subjective well-being (phone survey, 2020)		Subjective well-being (difference Round 5 (2016) and phone survey (2020))		Subjective well-being (phone survey, 2020)		Subjective well-being (difference Round 5 (2016) and phone survey (2020))		Subjective well-being (phone survey, 2020)		Subjective well-being (difference Round 5 (2016) and phone survey (2020))		Subjective well-being (phone survey, 2020)		Subjective well-being (difference Round 5 (2016) and phone survey (2020))	
	r	p-value	r	p-value	r	p-value	r	p-value	r	p-value	r	p-value	r	p-value	r	p-value
Mean GAD-7 score	0.140	0.000	0.041	0.054	0.107	0.000	0.064	0.001	0.199	0.000	0.096	0.000	0.253	0.000	0.118	0.000
Mean PHQ-8 score	0.091	0.000	0.024	0.263	0.097	0.000	0.055	0.005	0.186	0.000	0.067	0.004	0.207	0.000	0.119	0.000

Note: Results are for the combined Younger Cohort/ Older Cohort sample. Bonferroni corrected p-value.

Supplementary Table 35. Subjective well-being score Round 5 (2016) / phone survey (2020) by anxiety/depression (Ethiopia & India)

	Ethiopia							India						
	**All**	**at least mild anxiety**	**no anxiety**	**p-value**	**at least mild depression**	**no depression**	**p-value**	**All**	**at least mild anxiety**	**no anxiety**	**p-value**	**at least mild depression**	**no depression**	**p-value**
(1) Subjective well-being, 2016 (Round 5)	5.69	5.43	5.75	0.000	5.61	5.71	0.303	5.05	4.94	5.06	0.178	4.98	5.06	0.381
(2) Subjective well-being, 2020 (Phone survey)	4.73	4.28	4.83	0.000	4.36	4.80	0.000	4.56	4.18	4.61	0.000	4.12	4.61	0.000
Difference in subjective well-being (1)-(2)	0.96	1.15	0.92	0.052	1.26	0.91	0.005	0.49	0.77	0.45	0.009	0.86	0.45	0.001

Note: Results are for the combined Younger Cohort/ Older Cohort sample.

For peer review only

Supplementary Table 36. Subjective well-being score Round 5 (2016) / phone survey (2020) by anxiety/depression (Peru & Vietnam)

	Peru							Vietnam						
	**All**	**at least mild anxiety**	**no anxiety**	**p-value**	**at least mild depression**	**no depression**	**p-value**	**All**	**at least mild anxiety**	**no anxiety**	**p-value**	**at least mild depression**	**no depression**	**p-value**
(1) Subjective well-being, 2016 (Round 5)	6.33	6.20	6.42	0.001	6.14	6.42	0.000	5.87	5.46	5.91	0.000	5.65	5.89	0.024
(2) Subjective well-being, 2020 (Phone survey)	5.81	5.47	6.05	0.000	5.44	5.98	0.000	6.26	5.33	6.36	0.000	5.54	6.34	0.000
Difference in subjective well-being (1)-(2)	0.52	0.74	0.38	0.000	0.70	0.44	0.013	-0.39	0.14	-0.45	0.000	0.11	-0.45	0.000

Note: Results are for the combined Younger Cohort/ Older Cohort sample.

For peer review only

Supplementary figures

Supplement to: Impact of the COVID-19 Pandemic on anxiety and depression symptoms of young people in the Global South: evidence from a four-country cohort study

- Supplementary Figure 1

Supplementary Figure 1. Variables used in the analysis

Variables used in the analysis. Gender refers to male/female. Location refers to rural/urban.

For peer review only

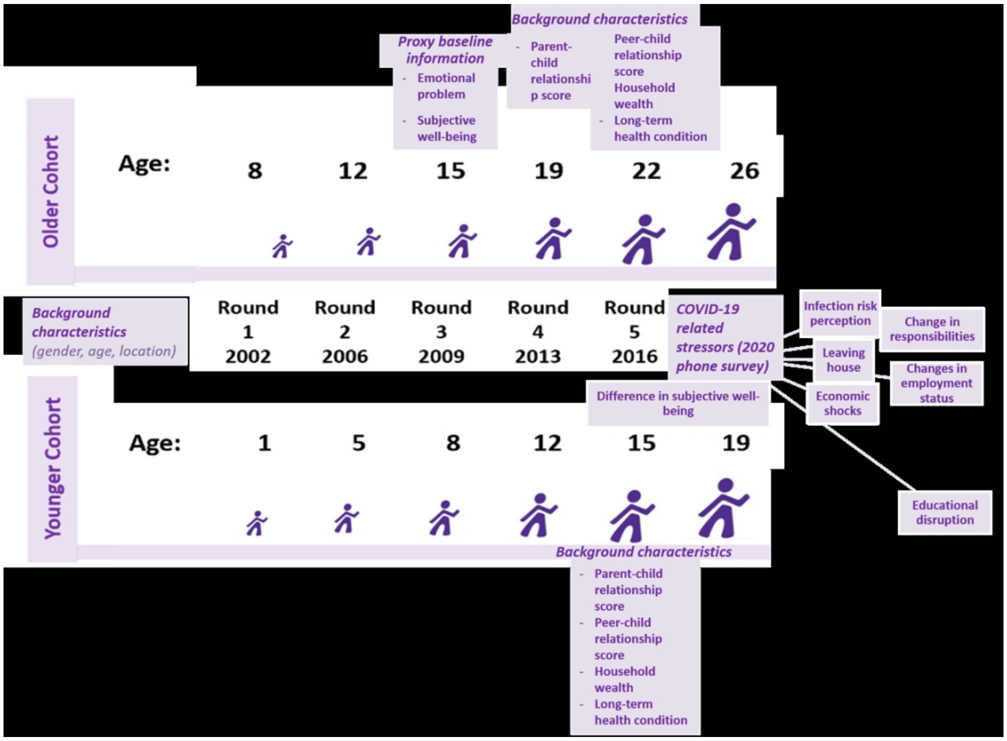
Background characteristics
- Parent-child relationship score
Peer-child relationship score
Household wealth
- Long-term health condition
Proxy baseline information
- Emotional problem
- Subjective well-being
Older Cohort
Age:
8
12
15
19
22
26
Background characteristics (gender, age, location)
Round 1 2002
Round 2 2006
Round 3 2009
Round 4 2013
Round 5 2016
COVID-19 related stressors (2020 phone survey)
Infection risk perception
Change in responsibilities
Leaving house
Changes in employment status
Economic shocks
Educational disruption
Difference in subjective well-being
Younger Cohort
Age:
1
5
8
12
15
19
Background characteristics
- Parent-child relationship score
- Peer-child relationship score
- Household wealth
- Long-term health condition

Research checklist

STROBE Statement for: *Impact of the COVID-19 Pandemic on anxiety and depression symptoms of young people in the Global South: evidence from a four-country cohort study*

	Item No	Recommendation	Reported on page #
**Title and abstract**	1	(*a*) Indicate the study's design with a commonly used term in the title or the abstract	1
		(*b*) Provide in the abstract an informative and balanced summary of what was done and what was found	2
**Introduction**			
Background/rationale	2	Explain the scientific background and rationale for the investigation being reported	3
Objectives	3	State specific objectives, including any prespecified hypotheses	3-4<br>Figure 2
**Methods**			
Study design	4	Present key elements of study design early in the paper	4
Setting	5	Describe the setting, locations, and relevant dates, including periods of recruitment, exposure, follow-up, and data collection	4
Participants	6	(*a*) Give the eligibility criteria, and the sources and methods of selection of participants. Describe methods of follow-up	4
		(*b*) For matched studies, give matching criteria and number of exposed and unexposed	N/A
Variables	7	Clearly define all outcomes, exposures, predictors, potential confounders, and effect modifiers. Give diagnostic criteria, if applicable	4-5
Data sources/ measurement	8*	For each variable of interest, give sources of data and details of methods of assessment (measurement). Describe comparability of assessment methods if there is more than one group	4-5<br>Supplementary Figure 1
Bias	9	Describe any efforts to address potential sources of bias	10, 12
Study size	10	Explain how the study size was arrived at	4
Quantitative variables	11	Explain how quantitative variables were handled in the analyses. If applicable, describe which groupings were chosen and why	4-6
Statistical methods	12	(*a*) Describe all statistical methods, including those used to control for confounding	4, 5, 6
		(*b*) Describe any methods used to examine subgroups and interactions	4, 5, 6
		(*c*) Explain how missing data were addressed	2, 4, 8
		(*d*) If applicable, explain how loss to follow-up was addressed	N/A
		(*e*) Describe any sensitivity analyses	N/A

For peer review only - http://bmjopen.bmj.com/site/about/guidelines.xhtml
For peer review only

**Results**			
Participants	13*	(a) Report numbers of individuals at each stage of study—eg numbers potentially eligible, examined for eligibility, confirmed eligible, included in the study, completing follow-up, and analysed	2, 4<br>Supplementary Tables 1- 3
		(b) Give reasons for non-participation at each stage	2, 4<br>Supplementary Tables 1- 3
		(c) Consider use of a flow diagram	N/A
Descriptive data	14*	(a) Give characteristics of study participants (eg demographic, clinical, social) and information on exposures and potential confounders	4<br>Supplementary Table 5
		(b) Indicate number of participants with missing data for each variable of interest	2, 4<br>Supplementary Tables 1- 3
		(c) Summarise follow-up time (eg, average and total amount)	N/A
Outcome data	15*	Report numbers of outcome events or summary measures over time	4
Main results	16	(*a*) Give unadjusted estimates and, if applicable, confounder-adjusted estimates and their precision (eg, 95% confidence interval). Make clear which confounders were adjusted for and why they were included	6-14
		(*b*) Report category boundaries when continuous variables were categorized	N/A
		(*c*) If relevant, consider translating estimates of relative risk into absolute risk for a meaningful time period	N/A
Other analyses	17	Report other analyses done—eg analyses of subgroups and interactions, and sensitivity analyses	5-6, 9-14<br>Supplementary Tables 6-9, 16-23
**Discussion**			
Key results	18	Summarise key results with reference to study objectives	15
Limitations	19	Discuss limitations of the study, taking into account sources of potential bias or imprecision. Discuss both direction and magnitude of any potential bias	16
Interpretation	20	Give a cautious overall interpretation of results considering objectives, limitations, multiplicity of analyses, results from similar studies, and other relevant evidence	16-17
Generalisability	21	Discuss the generalisability (external validity) of the study results	16
**Other information**			
Funding	22	Give the source of funding and the role of the funders for the present study and, if applicable, for the original study on which the present article is based	18

For peer review only

*Give information separately for exposed and unexposed groups.

Note: An Explanation and Elaboration article discusses each checklist item and gives methodological background and published examples of transparent reporting. The STROBE checklist is best used in conjunction with this article (freely available on the Web sites of PLoS Medicine at http://www.plosmedicine.org/, Annals of Internal Medicine at http://www.annals.org/, and Epidemiology at http://www.epidem.com/). Information on the STROBE Initiative is available at http://www.strobe-statement.org.

For peer review only
